# The Intricate Nonribosomal Assembly of a Potent Antifungal Lipopeptide from the Burkholderia cepacia Complex

**DOI:** 10.1021/jacs.5c04167

**Published:** 2025-06-02

**Authors:** Lei Zhong, Agnes Mühlenweg, Dou Hong, Sarah Yammine, Annette Poch, Dingchang Xu, Yasemin Kirimlioglu, Lisa Großgloß, Malo Boulanger, Franziska Graeger, Maria Seidel, Manuel Gemander, Grit Walther, Sebastian Kemper, Tam Dang, Monique Royer, Andi Mainz, Stéphane Cociancich, Roderich D. Süssmuth

**Affiliations:** 1 Institut für Chemie, 26524 Technische Universität Berlin, Straße des 17. Juni 124, Berlin 10623, Germany; 2 CIRAD, UMR PHIM, Montpellier 34398, France; 3 PHIM, Univ Montpellier, CIRAD, INRAE, Institut Agro, IRD, Montpellier 34398, France; 4 National Reference Center for Invasive Fungal Infections, Leibniz Institute for Natural Product Research and Infection Biology, Hans Knöll Institute, Jena 07745, Germany

## Abstract

The Burkholderia cepacia complex (BCC) is a group of Gram-negative bacteria known for their pathogenicity to patients suffering from cystic fibrosis (CF). The BCC-belonging strain B. pyrrocinia BC11 (formerly *B. cepacia* BC11) produces AFC-BC11, a compound with strong activity against phytopathogenic fungi. In this contribution, we report on the unprecedented *N*-acyl-tetrapeptide structure and antifungal potency of this natural product. We further provide insights into central steps of its biosynthesis mediated by a nonclassical nonribosomal peptide synthesis machinery lacking condensation domains. With the involvement of a sole acyl/peptidyl carrier protein AfcK, an acyltransferase AfcL and coenzyme A, the growing acyl-peptide chain is shuffled between different thioester carriers during the intricate biosynthetic assembly. The knowledge of the AFC-BC11 structure may contribute to the development of antifungals against phytopathogens and, with the *afc* gene cluster being conserved in various *Burkholderia* strains, possibly to an understanding of the human pathogenesis of the BCC.

## Graphical abstract

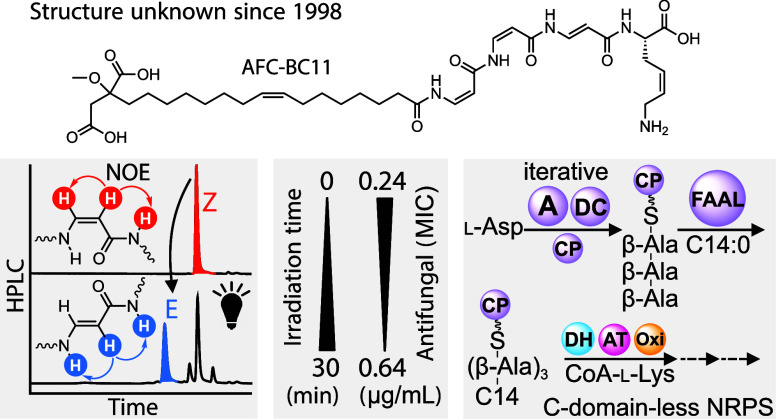

## Introduction

Bacteria of the genus *Burkholderia* have been described as degraders of polychlorinated pollutants but are also connected to severe animal (e.g., glanders) and human diseases (e.g., melioidosis).^1,2^ Patients suffering from cystic fibrosis (CF), a disease with symptoms of thickened mucus particularly in the lungs, commonly are coinfected with opportunistic bacteria from the *Burkholderia cepacia* complex (BCC).^3^ Further research into BCC also considered its use as a biological control agent for fungal infections of plants due to its broad and pronounced antifungal activity.^4^ Previously, the discovery of a compound named AFC-BC11 from *Burkholderia pyrrocinia* BC11 (formerly *B. cepacia* BC11) was reported. The compound displayed strong antifungal activity against *Rhizoctonia solani*, the causative agent of the devastating plant disease damping-off of cotton.^5^ Moreover, the *afc* biosynthetic gene cluster (BGC) and its transcriptional regulator ShvR have been described as virulence factors of *B. cenocepacia*.^6–11^ While various reports emphasize the importance of AFC-BC11 for pathogenesis of CF^9–12^ as well as a potential antifungal agent,^5,8,13^ its structural characterization and biosynthetic assembly remained elusive.

## Results and Discussion

### AFC-BC11 is an Unusual Photosensitive *N*-acyl Tetrapeptide

The originally reported producer strain of AFC-BC11, formerly *B. cepacia* BC11,^5^ is not available in any public collection and therefore was replaced by *B. orbicola* Mc0–3 (formerly *B. cenocepacia* Mc0–3) and *B. puraquae* DSM 103137. Both strains belong to BCC (Table S1) and possess the *afc* biosynthetic gene cluster (BGC). The potential for the biosynthesis of AFC-BC11 is widespread in the BCC, as evidenced by the distribution of the corresponding *afc* BGC among various strains of this group (Tables S2 and S3, Figure S1).

Initial attempts to isolate compound AFC-BC11 from the above-mentioned *Burkholderia* strains were hampered by its rapid and complete decomposition during HPLC purification. An optimized production and isolation protocol (see Supporting Information) with the strict exclusion of light (see below) yielded AFC-BC11 in milligram quantities. HPLC-HR-Orbitrap ESI-MS gave a molecular mass of *m*/*z* 734.3906 for [M + H]^+^, which corresponds to a molecular formula of C_36_H_55_N_5_O_11_ (calculated for C_36_H_56_N_5_O_11_^+^ 734.3971, Δ*m* = – 8.9 ppm; Figure 1a). The ^1^H–^15^N SOFAST-HMQC NMR spectrum (Figures S2–S4) correlates four amide protons (δ_H_ 10.91, 10.99, 10.62, and 7.91 ppm) with their nitrogen neighbors (δ_N_ 105.6, 105.2, 104.1, and 121.6 ppm, respectively), indicating the presence of four peptide bonds in the molecule. Moreover, the analysis of ^1^H–^1^H COSY, ^1^H–^1^H TOCSY and ^1^H–^13^C HSQC/HMBC spectra (Figures S5–S9) revealed four amino acid spin systems, which were assigned to three dehydro-β-alanine (DBA) residues and one γ,δ-dehydro-lysine residue (DHLys) (Figure 1b). The scalar coupling constants between α and β protons of the three DBA residues (^3^ *J*_HαHβ_ 9.1, 9.4, and 13.7 Hz) revealed their *Z*-, *Z*- and *E*-configuration, respectively (Figures 1c and S2). The *Z*-configuration of DHLys was deduced from the coupling constants of Hγ (5.74 ppm,^3^ *J*_HγHδ_ 10.5 Hz) and Hδ (5.55 ppm,^3^ *J*_HδHγ_ 11.1 Hz) (Figure S10). The sequence of the peptide was obtained from inter-residual NOE correlations between NH_i_ and H_αi‑1_ (Figure S11) as (*Z*)-^1^DBA-(*Z*)-^2^DBA-(*E*)-^3^DBA-(*Z*)-^4^DHLys (Figure 1b). Further analysis of COSY and HMBC (Figures S5, S8 and S9) as well as HR-MS data (Figure 1a) revealed an *O*-methylated malic acid-fatty acid (MMFA) moiety featuring a fatty acyl-chain with a double bond between carbons C8 and C9 (Figure 1b). The ^13^C chemical shifts of allylic atoms C7/C10 at 27.2 ppm suggested the *Z*-configuration of that double bond (Figures 1b and S7).^14^ The *N*-acyl connection to the peptide part was established using diagnostic NOE correlations between the amide proton of (*Z*)-^1^DBA and H2/H3 of the fatty acyl-chain (Figures 1b and S11). Marfey analytics, subsequent to the reduction of double bonds and total hydrolysis of AFC-BC11, revealed the l-configuration for DHLys (Figure S12). The MS/MS data are in full accordance with the structure of AFC-BC11 (Figure S13) and of low abundance derivatives thereof (Figures S14 and S15). Overall, AFC-BC11 is an unusual *N*-acyl tetrapeptide comprising a DBA trimer core, which is N- and C-terminally decorated with a distinctive MMFA and DHLys moiety, respectively (Figures 1b and S16, Table S4).

**Figure 1:**
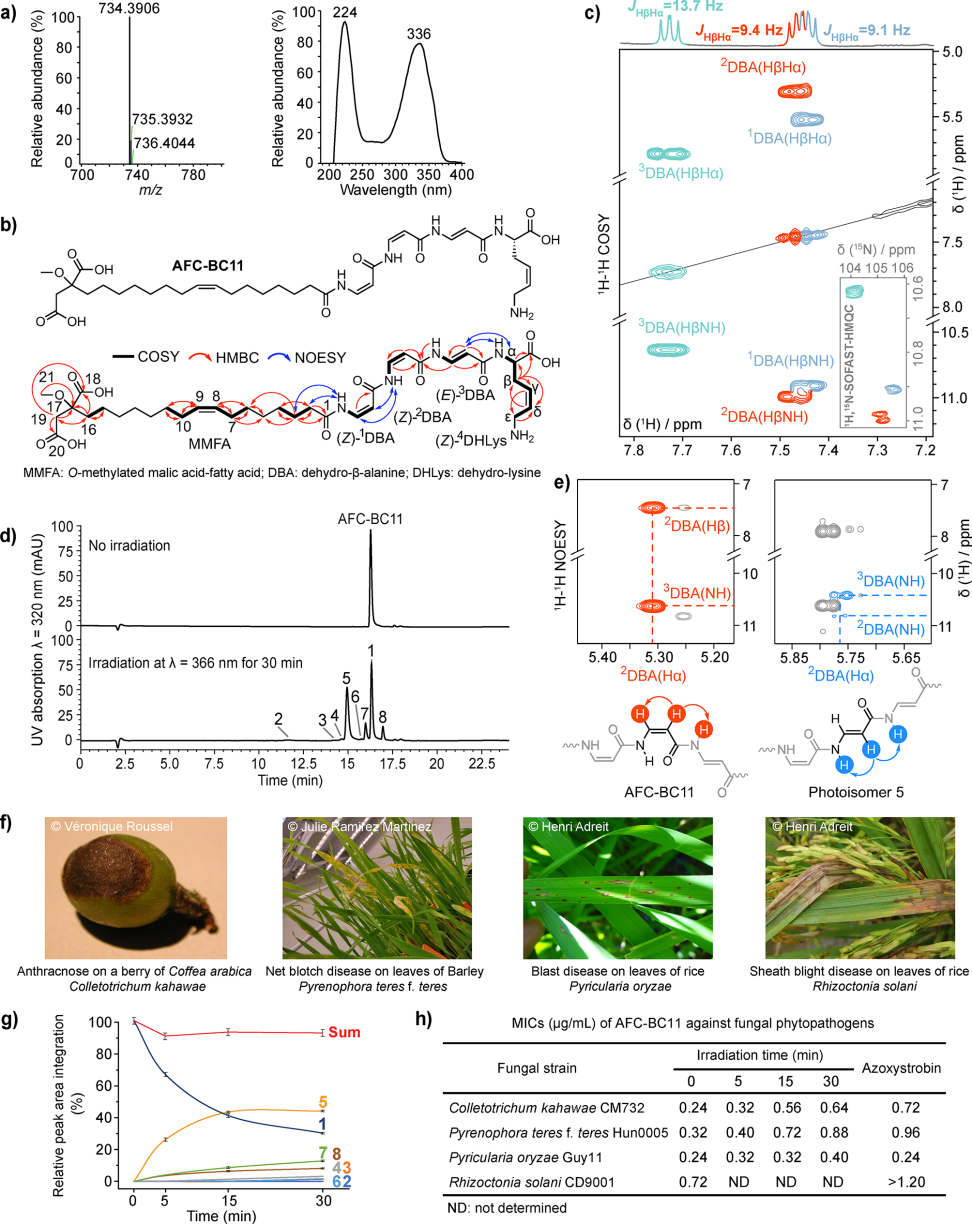
Structure, photoisomerization and antifungal assessment of AFC-BC11. (a) HR-MS and UV spectra, and (b) NMR-spectroscopic correlations for the structure of AFC-BC11. (c) ^1^H–^1^H COSY indicating Hβ-Hα/NH correlations in DBA trimer and ^1^H–^15^N SOFAST-HMQC highlighted. (d) HPLC chromatograms of AFC-BC11 before (top) and after (bottom) photoisomerization, with the parent compound coded as 1 and new photoisomers as 2–8 based on their elution times. (e) *E*-configured ^2^DBA in photoisomer 5 was characterized via diagnostic NOE signals. (f) Plant diseases caused by phytopathogenic fungi inhibited by AFC-BC11. (g) Photoisomerization kinetics of AFC-BC11 at λ = 366 nm for antifungal assays, with HPLC analysis conducted in triplicate and error bars indicating standard deviation. (h) Antifungal assays of AFC-BC11 after irradiation at λ = 366 nm for 0–30 min against phytopathogenic fungi, with azoxystrobin as the positive control. Reprinted with permission from Véronique Roussel, Julie Ramirez Martinez, and Henri Adreit. Copyright 2025.

Importantly, AFC-BC11 remained stable when dissolved in DMSO and rigorously protected from exposure to light (Figure S17). Photoisomerization for 12 h either by daylight or UV-A (366 nm) as well as UV–B (300 nm) or UV–C (254 nm) resulted in the detection of eight (*m*/*z* 734.39 and λ_max_ of 326–336 nm) and 16 isobaric peaks (Figures 1d and S18–S21), respectively, which is reminiscent of recent reports on photoswitchable α, β-peptide foldamers.^15^ NMR analysis of the first major isomerization product (photoisomer 5, Figures 1e and S22–S25) unambiguously identified ^2^DBA to undergo *Z*-to-*E* conversion upon UV irradiation. However, the rapid conversion into a multitude of products including compound degradation impeded the characterization of further isomers.

We observed a strong antifungal activity for pure AFC-BC11 against phytopathogenic fungi, e.g. *Colletotrichum kahawae* (coffee berry disease) and *Pyrenophora teres* f. *teres* (net blotch on barley) (Figure 1f), with minimal inhibitory concentrations (MICs) of 0.24–0.72 μg/mL, which outperforms the reference antifungal azoxystrobin (Figure 1h). Of note, UV irradiation over time diminished the antifungal activity of AFC-BC11 (Figures 1g/h, Table S5), which indicated that the (*Z*, *Z*, *E*)-configuration of the DBA trimer is critical for full antifungal activity. Interestingly, AFC-BC11 did not show activity against human pathogenic fungi, e.g. *Candida albicans* and *Aspergillus fumigatus* (Table S6),^16^ or against Gram-negative and Gram-positive bacteria (Figure S26).

### The *afc* BGC Lacks Classical Features of Nonribosomal Peptide Synthesis

The *afc* biosynthetic gene cluster of *B. orbicola* Mc0–3 comprises 25 genes (Figure 2a and Table S2). Despite AFC-BC11 being a peptide, the gene cluster misses features of a classical modular nonribosomal peptide synthetase (NRPS),^17^ such as condensation (C) domains essential for peptide-bond formation. On the other hand, genes *afcQ* and *afcK* encode for an NRPS-characteristic adenylation (A) domain and a carrier protein (CP), respectively. Based on bioinformatic analysis and AlphaFold2 models (see Supporting Information), further genes have been functionally annotated to encode for a potential fatty acyl-AMP ligase (*afcA*), a citrate synthase (*afcS*), a SAM-dependent *O*-methyltransferase (*afcT*) and various oxidoreductases (*afcC*/*D*/*E*/*F*/*J*/*N*/*U*). We speculated that the dehydrogenase/desaturase genes *afcC*/*D*/*E*/*J*/*N* may catalyze the installation of double bonds in residues MMFA, DBA and DHLys. Hence, apart from the oxidative tailoring, the set of *afc* genes implied an iterative coupling and processing toward a DBA trimer, reminiscent of the iterative amide bond elongation catalyzed by A domains in the biosynthesis of ferrichrome^18^ and streptothricin.^19^ The formation of DBA trimer would be accompanied or followed by activation and transfer of the N-terminal acyl chain and the coupling of l-Lys or l-DHLys, respectively. Accordingly, we expected the sole carrier protein AfcK to play a central role in the storage and shuttling of reaction intermediates (Figure 2b).

**Figure 2:**
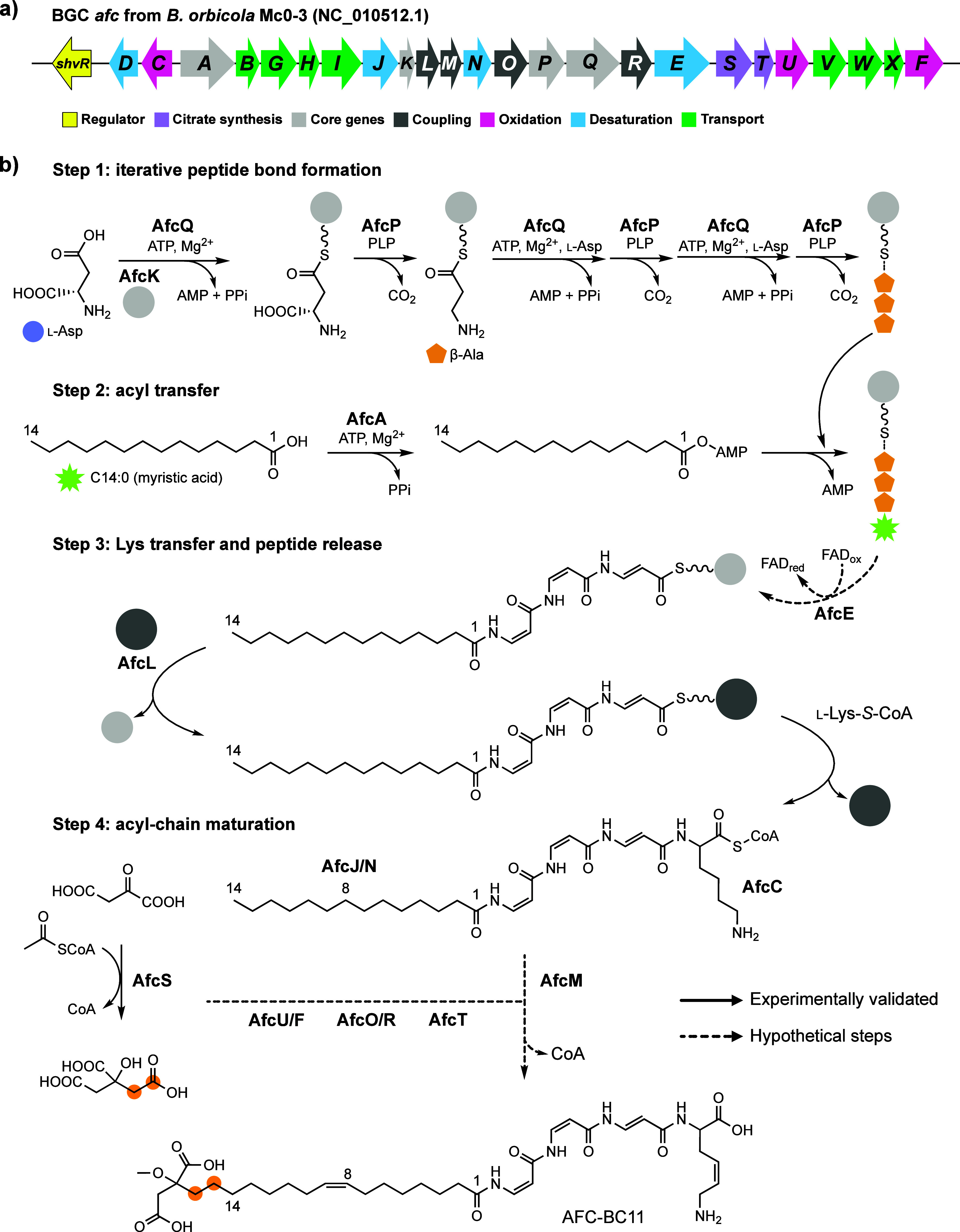
Proposed biosynthesis of AFC-BC11. (a) Arrangement of the *afc* biosynthetic gene cluster from *B. orbicola* Mc0–3. (b) Suggested biosynthetic assembly of AFC-BC11. (AfcK: carrier protein; AfcQ: adenylation domain; AfcP: PLP-dependent decarboxylase; AfcA: fatty acyl-AMP ligase; AfcL: acyltransferase (KAS III-like synthase); AfcC: oxidase; AfcS: citrate synthase; AfcT: SAM-dependent *O*-methyltransferase).

### Iterative Peptide-Bond Formation by the Adenylating Enzyme AfcQ

To foster an understanding of the biosynthetic assembly of AFC-BC11, we embarked on the *in vitro* reconstitution of central biosynthetic steps. Biosynthetic genes were heterologously expressed in *E. coli* and proteins were purified for biochemical assays (Table S7, Figure S27). The investigations started with the VinN-type (vicenistatin biosynthesis) A domain AfcQ (Figure S28),^20^ for which the specificity-conferring code of A domains predicted l-Asp as the substrate (Table S8).^21^ This was experimentally confirmed in a photometric hydroxylamine release assay (Figures 3a and S29a/b),^22^ which also clearly excluded a direct activation of β-Ala (Figure 3a). According to the biosynthetic logic of l-Asp as a precursor of DBA and the homology between AfcQ and VinN, the activation of l-Asp by AfcQ must occur at the side chain carboxylate to establish the β-amino acid scaffold found in AFC-BC11 (Figure 2b, step 1). An AlphaFold2 model of AfcQ combined with molecular docking via AutoDock Vina (see Supporting Information) provides a convincing explanation for its selectivity: the bulky residue Arg311 (Arg331 in VinN^20^) at the entrance of the substrate pocket most likely precludes the conventional accommodation of the substrate’s side chain, but instead allows binding of the α-carboxylate of l-Asp and thus orients the β-carboxylate toward the invariant catalytic residue Lys503 for the adenylation reaction (Figures 3a and S30).

**Figure 3:**
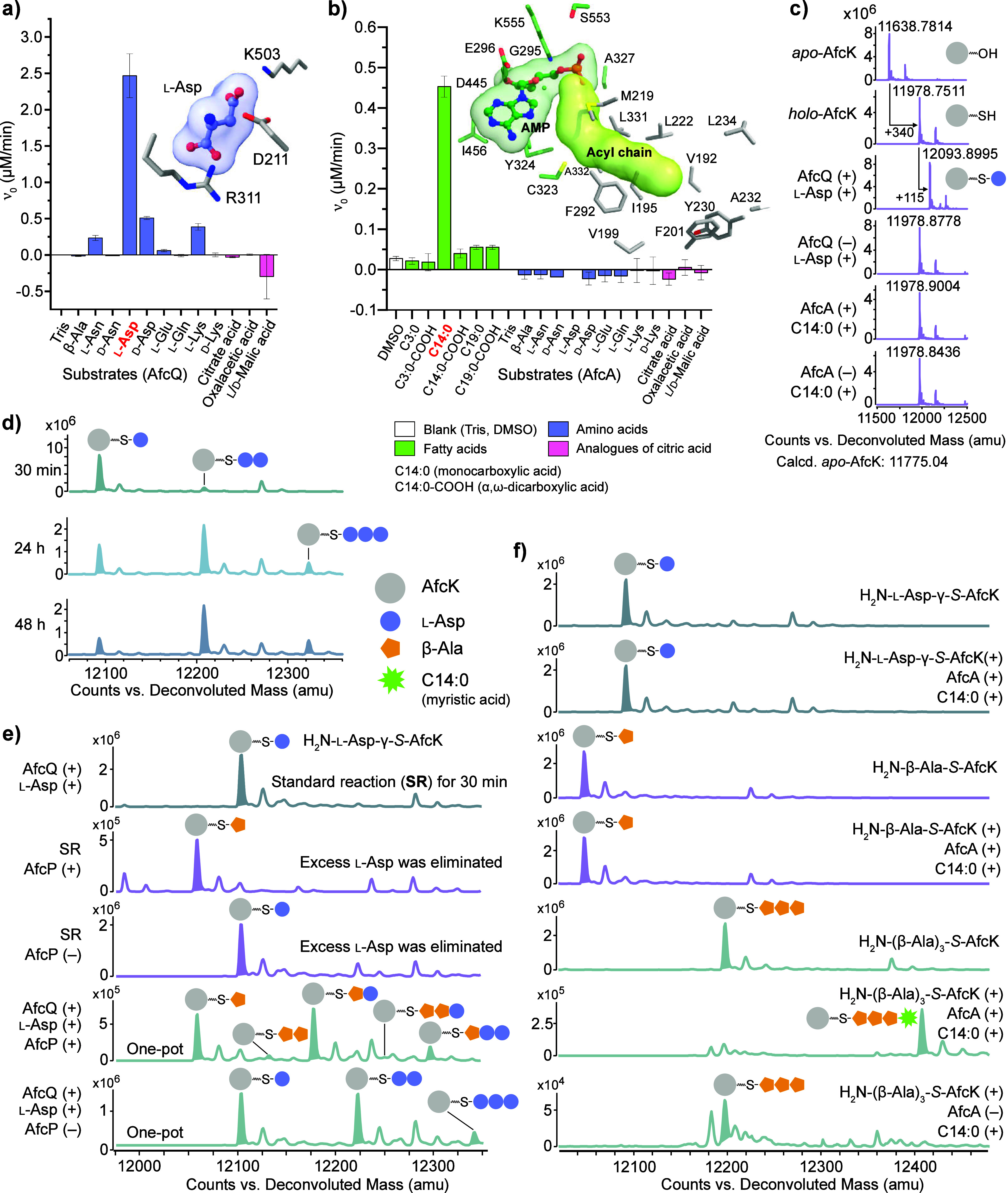
*In vitro* reconstitution of myristoyl-(β-Ala)_3_ assembly. Hydroxylamine release assays identified substrate activation of (a) l-Asp by the A-domain-like protein AfcQ and of (b) myristic acid (tetradecanoic acid, C14:0) by the fatty acyl-AMP ligase (FAAL) AfcA.^22^ The predicted substrate binding pockets with potential key residues are displayed. (c) Loading of the carrier protein AfcK with the prosthetic group and l-Asp substrate. (d) Multiple loading of l-Asp onto AfcK was observed upon longer incubation times. (e) α-decarboxylation by AfcP during AfcQ-catalyzed peptide elongation on AfcK. Single loading of l-Asp was observed in the standard reaction (SR, top). To prevent multiple loading of l-Asp, excess l-Asp was removed before adding AfcP in the second reaction. The fourth system represented a one-pot reaction for multiple l-Asp loading and decarboxylation. The other two reactions served as negative controls, lacking AfcP. (f) Selective transfer of activated C14:0 onto H_2_N-(β-Ala)_3_-*S*-AfcK by AfcA yielding myristoyl-(β-Ala)_3_-*S*-AfcK.

The AfcQ-catalyzed adenylation of l-Asp pointed to a thiotemplated biosynthesis mechanism, as has also been described for the assembly of β-Ala analogs in secondary metabolites, such as fluvirucin^23^ and vicenistatin.^20^ Accordingly, we considered AfcK bearing a conserved Ser40 (Figures S31 and S32) as the sole carrier protein with a key role in AFC-BC11 assembly (Figure 2b, step 1). In a first step, *apo*-AfcK was primed using phosphopantetheinyl transferase (PPTase) Sfp and CoA to install the prosthetic ppant group (Figures 3c and S33). We then incubated *holo*-AfcK with AfcQ and its (co)­substrates l-Asp/ATP, which resulted in successful loading of l-Asp onto the ppant arm of *holo*-AfcK as judged by HPLC-MS (Figures 3c and S33). Importantly, multiple loading events were observed upon longer incubation times (Figures 3d, S34 and S35). This observation demonstrated the basic principle of iterative peptide-bond formation by AfcQ: the thiol group of the ppant arm of *holo*-AfcK as well as the α-amino group of H_2_N-l-Asp-γ-*S*-AfcK are tolerated as the nucleophiles attacking the first and the subsequent H_2_N-l-Asp-γ-*O*-AMP intermediates provided by AfcQ, respectively. This is in contrast to conventional thiotemplated NRPS biosynthesis, in which the second half-reaction of A domains involves loading of the activated carboxylic acid onto the thiol of partner *holo*-CPs, whereas the subsequent peptide bond formation is catalyzed by C domains.

In this respect, we anticipated that in an *in vivo* scenario, H_2_N-l-Asp-γ-*S*-AfcK is first subject to α-decarboxylation by PLP-dependent AfcP (Figures S36–S38), thereby rendering β-Ala with an accessible β-amino group which would act as the acceptor in the next l-Asp-loading step (Figure 2b, step 1).

To this end, we performed a one-pot reaction with AfcQ, *holo*-AfcK and semipure AfcP as well as their (co)­substrates l-Asp, ATP and PLP. Indeed, we observed various species of *holo*-AfcK loaded with β-Ala/l-Asp peptides (Figures 3e and S39–S41) in which l-Asp is expected only in the N-terminal position. Moreover, the loading of subsequent l-Asp onto H_2_N-l-Asp-γ-*S*-AfcK was found to be slower than that onto H_2_N-(β-Ala)_n_-*S*-AfcK (n = 1–3) (Figure S42). Overall, these results confirmed that the decarboxylase AfcP acts on AfcK-loaded l-Asp and that transformation into β-Ala initiates the next l-Asp-loading cycle (Figure 2b, step 1).

### Acyl Transfer is Catalyzed by the Fatty Acyl-AMP Ligase AfcA

Activation of the acyl chain was assigned to AfcA, for which bioinformatic comparison showed similarities with fatty acyl-AMP ligases (FAALs) and fatty acyl-CoA ligases (FACLs) (Figure S43).^24^ This assumption was also supported by an AlphaFold2 model of AfcA, which revealed a hydrophobic substrate tunnel (Figure S44). A substrate screening using the photometric assay^22^ established for AfcQ (Figure 3a) yielded myristic acid (tetradecanoic acid, C14:0) as one of the major substrates, whereas short (C3) to long chain (C19) fatty acids, dicarboxylic (fatty) acids (e.g., tetradecanedioic acid, C14:0-COOH) or any type of amino acid were not activated by AfcA (Figures 3b and S29c). Mass spectrometric analyses for the product of AfcA activation were indicative of myristoyl-*O*-AMP, rather than myristoyl-*S*-CoA (Figure S45), which classified AfcA as FAAL. The subsequent transfer of activated myristic acid to either H_2_N-l-Asp-γ-*S*-AfcK or H_2_N-β-Ala-*S*-AfcK was not successful (Figures 3f, S46 and S47). We speculated that those substrates were not sufficient in length to reach myristoyl-*O*-AMP in the catalytic center of AfcA. Hence, we synthesized H_2_N-(β-Ala)_3_-*S*-CoA (see Supporting Information) and used it for priming of *apo*-AfcK with Sfp. The subsequent incubation of H_2_N-(β-Ala)_3_-*S*-AfcK with AfcA (including substrates myristic acid and ATP) yielded myristoyl-(β-Ala)_3_-*S*-AfcK (Figures 3f and S48) and thus demonstrated the successful acyl transfer to the terminal β-amino group (Figure 2b, step 2). The substrate selectivity for an appropriately extended β-Ala repeat attached to *holo*-AfcK attributed a gate-keeper function to AfcA and rationalized the exclusive presence of a DBA trimer in AFC-BC11.

### Lys Transfer and Peptide Release

The *in vitro* reconstitution of the myristoyl-(β-Ala)_3_ assembly led us to investigate the attachment of l-Lys into the peptide chain. We anticipated the α-amino group of l-Lys as substrate acceptor during the release step from myristoyl-(β-Ala)_3_-*S*-AfcK. Ketoacyl synthase (KAS) III-like proteins AfcL/O/R, which all show a similar fold with a catalytic triad of Cys/His/Asp (AfcL/O) and Thr/His/Glu (AfcR), respectively, were candidate enzymes for this transfer (Figures 4a and S49, Table S9). In particular, AfcL showed similarities to acyltransferase SfaN which has been shown to store and shuttle thioester intermediates in sanglifehrin A biosynthesis.^25^ Incubation of purified AfcL with synthetic H_2_N-(β-Ala)_n_-SNAc (*N*-acetylcysteamine thioester) surrogates (n = 1–4) yielded various peptidyl-*S*-enzyme conjugates, i.e. H_2_N-(β-Ala)_n_-*S*-AfcL with H_2_N-(β-Ala)_3_-SNAc thioester being the preferred loading substrate in a competition experiment (Figures 4b, S50 and S51). This confirmed that AfcL stored the peptidyl chain as a thioester intermediate (Figure 2b, step 3), very likely on its catalytic Cys111 (Figure 4a). In addition to AfcL, we observed that AfcQ and AfcA also tolerated diverse SNAc-based substrates as AfcK mimics albeit with lower selectivity compared to AfcK-loaded substrates (Figures S69–S76, Table S10).

**Figure 4:**
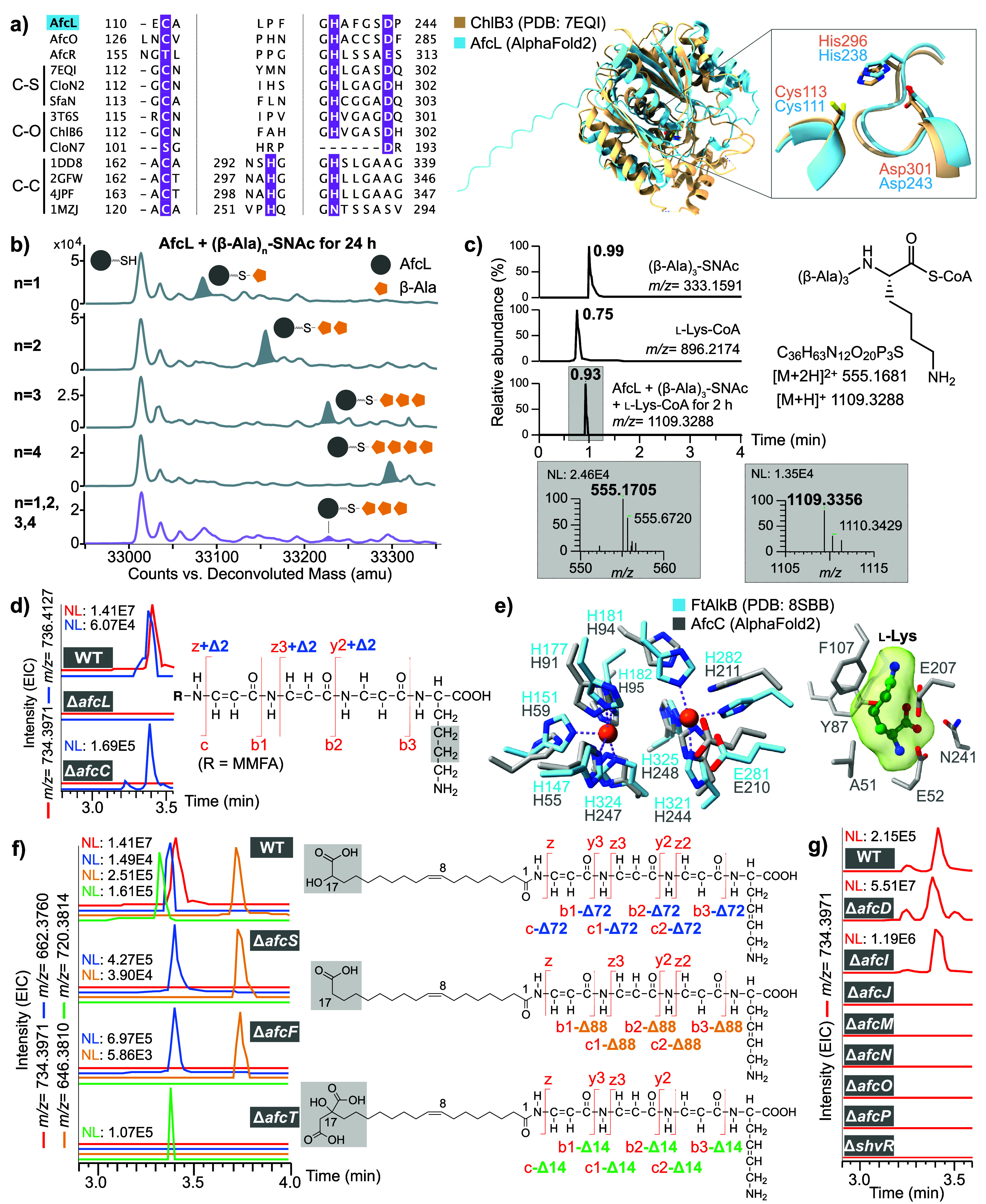
Lys-mediated peptide release and metabolic profiling of targeted mutants. (a) Catalytic triad (Cys/His/Asp) of AfcL aligned with KAS III-like enzymes for C–S, C–O and C–C bond formation (left), and structural superimposition with ChlB3 ^33^ highlighting the conserved triad (right). (b) Incubation of AfcL with synthetic H_2_N-(β-Ala)_n_-SNAc surrogates (*n* = 1–4) yielded H_2_N-(β-Ala)_n_-*S*-AfcL (top panels), with H_2_N-(β-Ala)_3_-SNAc as the preferred substrate in a competition experiment (bottom). (c) Successful release of H_2_N-(β-Ala)_3_ intermediate from AfcL to synthetic H_2_N-l-Lys-*S*-CoA. (d) Metabolic profiling of Δ*afcL* and Δ*afcC*. MS^2^ analysis localized the modification (Δ*m* of +2 Da) in the C-terminal part confirming an unprocessed l-Lys. (e) Structural superimposition of di-iron center in FtAlkB^34^ and AfcC. The predicted l-Lys binding pocket in AfcC from molecular docking is displayed. (f) Metabolic profiling of Δ*afcS*, Δ*afcF*, Δ*afcT*, and derived structures of metabolites. (g) Metabolic profiling of mutants for AFC-BC11 synthesis.

In terms of the release step, l-Lys alone was surprisingly incapable of liberating the H_2_N-(β-Ala)_3_ thioester intermediate from AfcL. Instead, we unequivocally observed a transfer to H_2_N-l-Lys-*S*-CoA (Figures 4c and S52). The formation of H_2_N-(β-Ala)_3_-l-Lys-*S*-CoA (Figure 2b, step 3) thus implied that an additional reaction for final product release would be required, which however could not be assigned to an apparent enzymatic function in the *afc* BGC. Notably, neither H_2_N-(β-Ala)_3_-*S*-AfcL nor H_2_N-(β-Ala)_3_-l-Lys-*S*-CoA were accepted as substrates for myristoyl loading by AfcA (Figures S53 and S54), which strongly suggests that the acyl-transfer step occurs in the preceding AfcK-loaded state (Figure 2b, step 2).

To support results from the *in vitro* reconstitution experiments, we established gene inactivation mutagenesis of strain *B. pyrrocinia* DSM 10685 (see Supporting Information). Hence, the biosynthetic importance of KAS III-like AfcL was confirmed, as the deletion of *afcL* fully abolished the production of AFC-BC11 (Figure 4d). The putative desaturase AfcC was initially predicted to be involved in double-bond formation in MMFA, but instead catalyzed the dehydrogenation of the C-terminal l-Lys (Figure 2b), which was in agreement with its rather negatively charged substrate pocket (Figures 4e and S55a-e). HPLC-MS analysis of extracts from the Δ*afcC* mutant showed a major product mass of *m*/*z* 736.4142 for [M + H]^+^ (calcd. for C_36_H_58_N_5_O_11_^+^ 736.4127, Δ*m* = 2.0 ppm), corresponding to a mass difference Δ*m* of +2 Da compared to the WT strain. Importantly, MS^2^ experiments unambiguously localized this modification to the C-terminal part of AFC-BC11 (Figures 4d and S56) confirming an unprocessed l-Lys. We consider this a late-stage maturation step most probably after release from AfcK, since AfcC appears to be membrane-embedded (Figures S55f/g).

### Enigmatic Acyl-Chain Maturation

While we succeeded in reconstituting the major steps of this unusual peptide assembly, the exact chemistry and time points of MMFA processing remain mostly elusive (Figure 2b, step 4). The activation and transfer of myristic acid by AfcA and not of dicarboxylic acids as mimics of MMFA is a strong argument for late-stage maturation of the assembled lipopeptide. Several gene knockout mutants of *B. pyrrocinia* DSM 10685 revealed partial insights into this acyl chain maturation (Figure 4f).

Gene inactivation of *afcS* with putative citrate synthase function (Figures S57 and S58) yielded two main products with molecular masses of *m*/*z* 662.3758 and 646.3813 for [M + H]^+^ (calcd. for C_33_H_52_N_5_O_9_^+^ 662.3760, Δ*m* = – 0.3 ppm; for C_33_H_52_N_5_O_8_^+^ 646.3810, Δ*m* = 0.5 ppm), respectively. The mass shifts Δ*m* of – 72 Da and – 88 Da correspond to a C_3_H_4_O_2_ and C_3_H_4_O_3_ fragment missing in the acyl part according to MS^2^ spectra, respectively (Figures 3f and S59). Interestingly, the inactivation of the putative FAD-dependent oxidoreductase gene *afcF* led to the same compounds (Figure 4f). This suggested that both enzymes AfcS and AfcF are involved in MMFA synthesis, and more precisely in coupling of a citrate precursor, most likely citryl-CoA, to the fatty acyl tail (Figure 2b, step 4). Reasonable surrogates could be oxaloacetate and succinate (Figure 4f) as suggested previously for bolagladin biosynthesis that also features an MMFA segment and enzyme homologues BolR (AfcS) and BolE (AfcF).^26,27^ As expected, AfcT (homologous to BolS)^26,27^ showed *O*-methyltransferase activity (Figure S60) as evidenced by the deletion mutant Δ*afcT* with a main product of *m*/*z* 720.3897 for [M + H]^+^ (calcd. for C_35_H_54_N_5_O_11_^+^ 720.3815, Δ*m* = 11.4 ppm), indicating the loss of a methyl group (Δ*m* = – 14 Da) in the malic acid portion (Figures 4f and S61).

Finally, deletion mutants of the transcriptional regulator Δ*shvR*, the dehydrogenase- and oxidoreductase-type genes Δ*afcJ* and Δ*afcN*, the UDP-glycosyltransferase-type gene Δ*afcM*, the decarboxylase gene Δ*afcP* as well as the KAS III-like enzyme gene Δ*afcO* were all incapable of AFC-BC11 production (Figure 4g) pointing to their essential biosynthetic function. This result was unexpected for AfcN (Figure 4g), which shares high homology to acyl-ACP desaturases and thus was suspected to install the double bond in MMFA. By contrast, the other putative acyl-ACP desaturase AfcD was revealed to be dispensable as we observed a WT production profile for mutant Δ*afcD*, which was also the case for mutant Δ*afcI* encoding for an outer-membrane protein channel (Figure 4g). It should be noted that AfcI and the other membrane­(-associated) proteins AfcB/C/G/H/V/W/X were not accessible to complementary *in vitro* assays.

## Conclusions

AFC-BC11 is a new antifungal chemotype with an unprecedented peptidomimetic-like/foldamer-like structure.^28^ Characteristic features of this *N*-acyl-tetrapeptide comprise the N-terminal MMFA moiety, the DBA trimer and the C-terminal DHLys. Key for a successful isolation and structure elucidation was the strict avoidance of UV irradiation, which explains previous failures to isolate the mature compound.^5^ We observed the overall trend of AFC-BC11 accumulation in the liquid culture (Figure S62) and expect cell membrane-embedded AFC-BC11 to be less susceptible to photoisomerization than its free form as handled during isolation (Figure S63). Prolonged exposure to light led to isomerization and reduction of the antifungal activity, which is reminiscent of a photoswitch that may be relevant for the interaction of the phytopathogen in a plant environment.^29^

The AFC-BC11 structure as well as the *afc* biosynthetic gene cluster (Figures 2a, Table S2) share certain similarities with the bolagladins from strain *B. gladioli*,^26,27^ which concerns particularly the MMFA moiety. However, the peptide part of the bolagladin biosynthesis basically follows the logic of a classic multimodular NRPS. In contrast, AFC-BC11 is assembled independently of NRPS-characteristic C domains, albeit based on a thiotemplated mechanism with the carrier protein AfcK being the central mediator in an elaborate network of autonomous enzymes. The core of the AFC-BC11 biosynthesis is the iterative activation and loading of l-Asp by AfcQ/AfcK directly followed by α-decarboxylation to β-Ala by AfcP (Figure 2b, step 1). This iterative process involves a noncanonical thiolation-to-amination switch in the second half reaction of A domain AfcQ. In the literature, there are only few examples of A domains capable of utilizing a terminal amine as a flexible extension of the ppant arm to attack an adenylated intermediate. In those cases, the tolerance of A domains toward amines as acceptor moieties inherently leads to an iterative coupling of amino acids such as Gly (ferrichrome)^18^ and β-Lys (streptothricin).^19^ The straightforward *in vitro* reconstitution of myristoyl-(β-Ala)_3_-*S*-AfcK demonstrated that the FAAL AfcA controls the β-alanyl chain length rendering exclusively a DBA trimer in AFC-BC11 (Figure 2b, step 2).

During bolagladin biosynthesis, a single l-Asp residue is converted to DBA, and inactivation of the dehydrogenase BolQ yielded the corresponding β-Ala analog.^26,27^ We expect AfcE, as the designated homologue of BolQ in the *afc* BGC, to be responsible for the successive dehydrogenation of three β-Ala residues into the DBA trimer of AFC-BC11 (Figure 2b). Accordingly, we suggest that myristoyl-(β-Ala)_3_-*S*-AfcK is the substrate for AfcE, which shows traits of an acyl-ACP dehydrogenase with a hydrophobic substrate tunnel incompatible with a charged H_2_N-β-Ala-*S*-AfcK substrate (Figures S64–S67). Moreover, an unprotected enamine as in H_2_N-DBA-*S*-AfcK could readily lead to isomerization to the imine,^30^ followed by hydrolysis and disintegration of the molecule (Figure S68). An additional level of complexity is added by the (*Z*, *Z*, *E*)-configuration of the DBA trimer in AFC-BC11, which might be dictated by steric requirements in the AfcE substrate pocket and the docking mode of myristoyl-(β-Ala)_3_-*S*-AfcK. However, we cannot exclude the involvement of a second dehydrogenase such as AfcJ.

Remarkably, the release of myristoyl-(β-Ala)_3_ from AfcK was pinpointed to the KAS III-like enzyme AfcL which most likely forms a myristoyl-(β-Ala)_3_-*S*-AfcL intermediate and subsequently couples H_2_N-l-Lys-*S*-CoA instead of l-Lys (Figure 2b, step 3). The biosynthetic purpose of employing an activated l-Lys species is currently unclear but may hint at substrate requirements of CoA-dependent enzymes during late-stage maturation. The activation of l-Lys as a CoA derivative could occur via mechanisms which have been previously described for aminoacyl-tRNA synthetases^31^ or aminoacyl-CoA ligases.^32^

It is noteworthy that the transfer step onto AfcL may instead involve myristoyl-(DBA)_3_-*S*-AfcK or even MMFA-(DBA)_3_-*S*-AfcK, as the exact time points of β-alanyl dehydrogenation and acyl-chain maturation have not been the focus of this study. The exact mechanism of coupling a citryl-CoA precursor to the terminal methyl group of a myristoyl intermediate needs further investigation, which would inevitably include the arsenal of membrane­(-associated) proteins AfcB/C/G/H/I/V/W/X. Their presence in the *afc* BGC indicates a dedicated system for recruitment, processing and trafficking of (im)­mature AFC-BC11 species at and across the inner and outer lipid membranes of *Burkholderia*.

In summary, the biosynthesis of AFC-BC11 combines features of NRPS and PKS with noncanonical mechanisms such as peptide-bond formation by an A domain (AfcQ) and by a KAS III-like enzyme (AfcL). Notably, the manifold Afc enzymes are autonomous catalysts and require a central mediator (AfcK) for storage and directional transfer of reaction intermediates. Finally, the knowledge on the structure of AFC-BC11 enables future studies on its function for *Burkholderia* species and its possible role in the pathogenesis of BCC in CF. Since AFC-BC11 has a pronounced antifungal activity against important phytopathogenic fungi, it may serve as a scaffold for synthetic analogs and the development of new antifungal agents.

## Supplementary Material



## Notes

Detailed description of material and methods: general analytical methods; fermentation, extraction, isolation, structure elucidation, and photoisomerization of AFC-BC11; bioactivity testing of AFC-BC11; BLAST, phylogenetic analysis, MSA, protein structure prediction, and docking study of key enzymes; genomic DNA isolation and cloning of plasmids; protein purification; generation of in-frame deletion mutants; *in vitro* assays; chemical synthesis of substrates; supplementary tables including microorganisms, media, buffers, and substrates used in this study; acquisition parameters for NMR measurements; PCR primers used to clone plasmids and constructs; NMR data and MIC values of AFC-BC11; supplementary figures including SDS-PAGE of purified proteins; HPLC chromatograms for monitoring photoisomerization; MS^2^ spectra of AFC-BC11, its congeners and photoisomers; NMR spectra of AFC-BC11, photoisomers, and chemically synthesized substrates; Marfey’s analysis; growth curve of B. puraquae; AlphaFold2 predicted models of key enzymes; MS spectra of *in vitro* assays and metabolic profiling of mutants (PDF)

## Abbreviations

BCC: Burkholderia cepacia complex
CF: Cystic fibrosis
BGC: Biosynthetic gene cluster
DBA: Dehydro-β-alanine
DHLys: Dehydro-lysine
MMFA: *O*-methylated malic acid-fatty acid
MIC: Minimal inhibitory concentration
NRPS: Nonribosomal peptide synthetase
C domain: Condensation
A domain: Adenylation
CP: Carrier protein
FAAL: Fatty acyl-AMP ligase
C14:0: Myristic acid/tetradecanoic acid
C14:0-COOH: Tetradecanedioic acid
KAS: Ketoacyl synthase
