# Supplementary material for: The Intricate Nonribosomal Assembly of a Potent Antifungal Lipopeptide from the Burkholderia cepacia Complex
Source: J Am Chem Soc. 2025 Jun 2;147(24):20725–34. doi: 10.1021/jacs.5c04167 (PMC12186527; doi:10.1021/jacs.5c04167)
Supplement: Supplementary file 1 [file ja5c04167_si_001.pdf]

Supplementary Information

The intricate non-ribosomal assembly of a potent antifungal lipopeptide from the *Burkholderia cepacia* complex

Lei Zhong[1], Agnes Mühlenweg[1], Dou Hong[1], Sarah Yammine[2,3], Annette Poch[1], Dingchang Xu[1], Yasemin Kirimlioglu[1], Lisa Großgloß[1], Malo Boulanger[2,3], Franziska Graeger[1], Maria Seidel[1], Manuel Gemander[1], Grit Walther[4], Sebastian Kemper[1], Tam Dang[1], Monique Royer[2,3], Andi Mainz[1], Stéphane Cociancich[2,3] & Roderich D. Süssmuth[1]*

[1]Institut für Chemie, Technische Universität Berlin, Straße des 17. Juni 124, 10623 Berlin, Germany. [2]CIRAD, UMR PHIM, 34398 Montpellier, France. [3]PHIM, Univ Montpellier, CIRAD, INRAE, Institut Agro, IRD, 34398 Montpellier, France. [4]National Reference Center for Invasive Fungal Infections, Leibniz Institute for Natural Product Research and Infection Biology, Hans Knöll Institute, 07745 Jena, Germany.

Table of contents

List of supplementary tables………………………………………………………………………….. 3
List of supplementary figures…………………………………………………………………………. 4
1. General analytical methods…………………………………………………………………………... 6
2. Fermentation and isolation…………………………………………………………………………. 7
3. Photoisomerization…………………………………………………………………………………. 8
4. Biological testing……………………………………………………………………………………. 8
5. Bioinformatic analysis………………………………………………………………………………. 9
6. Molecular biology……………………………………………………………………………………. 9
7. *In vitro* enzymatic assays………………………………………………………………………… 11
8. Chemical synthesis of enzyme substrates………………………………………………………… 13
References……………………………………………………………………………………………… 103

List of supplementary tables

Supplementary Table 1. List of species belonging to the *Burkholderia cepacia* complex (BCC). 14
Supplementary Table 2. Putative functions of proteins from the *afc* BGC based on BlastP search and comparison to the *bol* BGC. ..15
Supplementary Table 3. Compilation of the occurrence of *afc* BGC in BCC based on BlastN screening for key genes *afcA*, *afcQ* and the entire *afc* BGC. 17
Supplementary Table 4. NMR data of AFC-BC11 (700 MHz, DMSO-d_6, 298K). 33
Supplementary Table 5. MICs (µg/mL) of AFC-BC11 against selected phytopathogenic fungi depending on UV irradiation. 42
Supplementary Table 6. MICs (µg/mL) of AFC-BC11 against selected human pathogenic fungi. 42
Supplementary Table 7. Overview of proteins purified in this study. 43
Supplementary Table 8. Specificity codes of selected adenylation domains involved in the activation of L-Asp or its analogs. 45
Supplementary Table 9. Selected proteins involved in C-C, C-N, C-S, and C-O bond formation. 66
Supplementary Table 10. Observed peptide bond formation catalyzed by AfcQ and AfcA when using SNAc thioesters as acceptor. .91
Supplementary Table 11. Microorganisms used in this study. 92
Supplementary Table 12. Media used in this study. 93
Supplementary Table 13. Buffers used in this study. 93
Supplementary Table 14. Carboxylic acids used in this study for substrate specificity assays. 93
Supplementary Table 15. Acquisition parameters for NMR measurements in this study. 94
Supplementary Table 16. PCR primers used to clone plasmids for heterologous expression. 94
Supplementary Table 17. PCR primers used to clone constructs for in frame deletion mutants. 95

List of supplementary figures

Supplementary Figure 1. The *afc* BGC is conserved in eight BCC species. 19
Supplementary Figure 2. ^{1}H-NMR spectrum of AFC-BC11. 20
Supplementary Figure 3. ^{1}H-^{15}N SOFAST-HMQC spectrum of AFC-BC11 (NS: 512, SW:35 and O1P:117 for F1). 21
Supplementary Figure 4. ^{1}H-^{15}N SOFAST-HMQC spectrum of AFC-BC11 (NS: 1024, SW:35 and O1P:120 for F1). 22
Supplementary Figure 5. ^{1}H-^{1}H COSY spectrum of AFC-BC11. 23
Supplementary Figure 6. ^{1}H-^{1}H TOCSY spectrum of AFC-BC11. 24
Supplementary Figure 7. ^{1}H-^{13}C HSQC spectrum of AFC-BC11. 25
Supplementary Figure 8. ^{1}H-^{13}C HMBC spectrum of AFC-BC11 (SW:236 and O1P:100 for F1). 26
Supplementary Figure 9. ^{1}H-^{13}C HMBC spectrum of AFC-BC11 (SW:19 and O1P:170 for F1). 27
Supplementary Figure 10. ^{1}H-NMR spectra of AFC-BC11 selectively homo-decoupled at 2.50 ppm and 3.43 ppm. 28
Supplementary Figure 11. ^{1}H-^{1}H NOESY spectrum of AFC-BC11. 29
Supplementary Figure 12. Marfey's amino acid analysis. 30
Supplementary Figure 13. ESI (+)-MS/MS of AFC-BC11 acquired by HPLC-ESI-LTQ-Orbitrap XL mass spectrometer. 31
Supplementary Figure 14. Mass screening of the crude extract prepared from the cell pellet of *B. orbicola* Mc0-3. 31
Supplementary Figure 15. ESI (+)-MS/MS of isomers and congeners of AFC-BC11 acquired by LTQ-Orbitrap. 32
Supplementary Figure 16. The structure of AFC-BC11 with atom numbering used in this study. 32
Supplementary Figure 17. AFC-BC11 is stable when stored in the dark. 34
Supplementary Figure 18. Monitoring of the photoisomerization of AFC-BC11 by analytical HPLC. 35
Supplementary Figure 19. Alignment of analytical HPLC chromatograms from Supplementary Figure 18. 36
Supplementary Figure 20. Peak area integration of photoisomers after irradiation by daylight and at λ = 366 nm. 37
Supplementary Figure 21. PDA and MS spectra of AFC-BC11 acquired by LTQ-Orbitrap XL after irradiation by daylight. 38
Supplementary Figure 22. Photoisomer 5 is instable for purification. 39
Supplementary Figure 23. HPLC analysis of the mixture of AFC-BC11 and its main photoisomer 5 for NMR. 39
Supplementary Figure 24. Overlay of NMR spectral sections of AFC-BC11 acquired prior and after irradiation at λ = 366 nm 40
Supplementary Figure 25. ^{1}H-^{1}H NOESY spectrum of AFC-BC11 after irradiation at λ = 366 nm for 45 min. 41
Supplementary Figure 26. MIC assays performed to evaluate biological activities of AFC-BC11. 43
Supplementary Figure 27. Protein purification of N-terminal His_6-tagged Afc proteins. 44
Supplementary Figure 28. Phylogenetic analysis of A domains involved in the bioproduction of β-alanine and its analogs. 45
Supplementary Figure 29. Substrate assays via the continuous photometric hydroxylamine release assay. 46
Supplementary Figure 30. Structural analysis of AfcQ. 47
Supplementary Figure 31. Alignment of AfcK with ACPs and PCPs. 48
Supplementary Figure 32. Phylogenetic analysis of CPs. 49
Supplementary Figure 33. Loading of *holo*-AfcK monitored by ESI (+)-Q-TOF. 50
Supplementary Figure 34. Multiple L-Asp-loading of *holo*-AfcK with single AfcQ incubation monitored by ESI (+)-Q-TOF. 51
Supplementary Figure 35. Multiple L-Asp-loading of *holo*-AfcK with repeated AfcQ incubation monitored by ESI (+)-Q-TOF. 52
Supplementary Figure 36. Phylogenetic tree of decarboxylases. 53
Supplementary Figure 37. Alignment of PLP-dependent decarboxylases. 54
Supplementary Figure 38. Structural analysis of AfcP. 55
Supplementary Figure 39. Decarboxylation assay of mono-loaded H_2N-L-Asp-γ-*S*-AfcK monitored by ESI (+)-Q-TOF. 56
Supplementary Figure 40. One-pot AfcQ/AfcK/AfcP decarboxylation assay monitored by ESI (+)-Q-TOF. 57
Supplementary Figure 41. Control one-pot reaction utilizing denatured AfcP. 58
Supplementary Figure 42. Comparison of the loading efficiency of L-Asp onto H_2N-L-Asp-γ-*S*-AfcK and H_2N-(β-Ala)$_n$-*S*-AfcK 59
Supplementary Figure 43. Alignment of AfcA with selected FAALs and FACLs 60
Supplementary Figure 44. Structural analysis of AfcA.. 61
Supplementary Figure 45. AfcA-catalyzed activation of myristic acid (C14:0) monitored with LTQ-Orbitrap. 62
Supplementary Figure 46. AfcA-catalyzed acyl transfer using H_2N-L-Asp-γ-*S*-AfcK as acceptor. 63
Supplementary Figure 47. AfcA-catalyzed acyl transfer using H_2N-β-Ala-*S*-AfcK as acceptor. 64
Supplementary Figure 48. AfcA-catalyzed acyl transfer using H_2N-(β-Ala)$_3$-*S*-AfcK as acceptor. 65
Supplementary Figure 49. Structural analysis of AfcL/O/R models. 67
Supplementary Figure 50. Loading of AfcL monitored by ESI (+)-Q-TOF. 68
Supplementary Figure 51. Competitive loading of AfcL monitored by ESI (+)-Q-TOF. 69
Supplementary Figure 52. Acyl-release from AfcL monitored by LTQ-Orbitrap XL. 69
Supplementary Figure 53. Loading test of fatty acid onto H_2N-(β-Ala)$_3$-*S*-AfcL monitored by ESI (+)-Q-TOF. 70
Supplementary Figure 54. Loading test of fatty acid onto H_2N-(β-Ala)$_3$-L-Lys-*S*-CoA monitored by LTQ-Orbitrap XL. 70
Supplementary Figure 55. Structural analysis of AfcC model. 71
Supplementary Figure 56. Metabolic profiling acquired with LTQ-Orbitrap XL of WT and Δ*afcC*. 72

Supplementary Figure 57. Alignment of AfcS with selected citrate synthases. 73
Supplementary Figure 58. Structural analysis of AfcS. 74
Supplementary Figure 59. Metabolic profiling of Δ*afcS*. 75
Supplementary Figure 60. Structural analysis of AfcT. 76
Supplementary Figure 61. Metabolic profiling of Δ*afcT*. 76
Supplementary Figure 62. The time course of *B. puraquae* DSM 103137 cell growth. 77
Supplementary Figure 63. AFC-BC11 produced and purified under different conditions. 77
Supplementary Figure 64. Alignment of AfcE, AfcJ, AfcN, and AfcD with selected FAD-dependent dehydrogenases 78
Supplementary Figure 65. Crystal structures of various FAD-dependent dehydrogenases 79
Supplementary Figure 66. Structural analysis of AfcE. 80
Supplementary Figure 67. Structural analysis of AlphaFold2-predicted AfcE model. 81
Supplementary Figure 68. Proposed enamine-imine tautomerization of the unprotected/capped terminal DBA. 82
Supplementary Figure 69. EICs of AfcQ-catalyzed reactions with SNAc thioesters acquired by LTQ-Orbitrap XL. 83
Supplementary Figure 70. EICs of AfcQ-catalyzed reactions with SNAc thioesters acquired by LTQ-Orbitrap XL 84
Supplementary Figure 71. EICs of AfcA-catalyzed reactions with SNAc thioesters acquired by LTQ-Orbitrap XL 85
Supplementary Figure 72. EICs of AfcA-catalyzed reactions with SNAc thioesters acquired by LTQ-Orbitrap XL. 86
Supplementary Figure 73. EICs of AfcA-catalyzed reactions with SNAc thioesters acquired by LTQ-Orbitrap XL. 87
Supplementary Figure 74. EICs of AfcA-catalyzed reactions with SNAc thioesters acquired by LTQ-Orbitrap XL. 88
Supplementary Figure 75. EICs of AfcA-catalyzed reactions with SNAc thioesters acquired by LTQ-Orbitrap XL. 89
Supplementary Figure 76. EICs of AfcA-catalyzed reactions with SNAc thioesters acquired by LTQ-Orbitrap XL. 90
Supplementary Figure 77. ^{1}H-NMR spectrum of synthetic H_2N-L-Asp-SNAc (**1**). 96
Supplementary Figure 78. ^{1}H-NMR spectrum of synthetic H_2N-(L-Asp)$_2$-SNAc (**2**). 96
Supplementary Figure 79. ^{1}H-NMR spectrum of synthetic H_2N-(L-Asp)$_3$-SNAc (**3**). 97
Supplementary Figure 80. ^{1}H-NMR spectrum of synthetic H_2N-(L-Asp)$_4$-SNAc (**4**). 97
Supplementary Figure 81. ^{1}H-NMR spectrum of synthetic H_2N-β-Ala-SNAc (**5**). 98
Supplementary Figure 82. ^{1}H-NMR spectrum of synthetic H_2N-(β-Ala)$_2$-SNAc (**6**). 98
Supplementary Figure 83. ^{1}H-NMR spectrum of synthetic H_2N-(β-Ala)$_3$-SNAc (**7**). 99
Supplementary Figure 84. ^{1}H-NMR spectrum of synthetic H_2N-(β-Ala)$_4$-SNAc (**8**). 99
Supplementary Figure 85. ^{1}H-NMR spectrum of synthetic H_2N-(β-Ala)$_1$-*S*-CoA (**9**). 100
Supplementary Figure 86. ^{1}H-NMR spectrum of synthetic H_2N-(β-Ala)$_2$-*S*-CoA (**10**). 100
Supplementary Figure 87. ^{1}H-NMR spectrum of synthetic H_2N-(β-Ala)$_3$-*S*-CoA (**11**). 101
Supplementary Figure 88. ^{1}H-NMR spectrum of synthetic H_2N-L-Lys-SNAc (**12**). 101
Supplementary Figure 89. ^{1}H-NMR spectrum of synthetic H_2N-L-Lys-*S*-CoA (**13**). 102

1. General analytical methods

1.1. General materials

Water was provided by a MilliQ water purification system (Merck Millipore) and used for the bacterial media, HPLC and LC-MS. HPLC- and MS-grade acetonitrile was purchased from VWR and formic acid from Carl Roth. Marfey's reagents were purchased from Tokyo Chemical Industry. Unless otherwise stated, all other reagents were purchased from Sigma-Aldrich.

1.2. Analytical and preparative high performance liquid chromatography (HPLC)

An Agilent 1100 system consisting of a G1312A binary pump, a G1315D diode array detector (DAD), a G1316A column compartment, a G1329A automatic liquid sampler (ALS) and a G1364C analytical fraction collector (FC) was used for sample analysis. The analytical HPLC chromatograms were acquired and displayed with Agilent ChemStation for LC 3D systems B.03.02 (Agilent Technologies, Waldbronn, Germany). Isolation procedure by preparative HPLC was carried out with an Agilent 1100 system, which consisted of two G1361A preparative pumps, a G2260A preparative ALS, a G1365B multiple wavelength detector (MWD) and a G1364B preparative FC. Alternatively, purification was performed with an Agilent 1260 Infinity II system equipped with a G7161B preparative binary pump, a G7163B preparative column compartment, a G7114A variable wavelength detector (VWD), and a G7158B preparative ALS/FC. The preparative HPLC chromatograms were acquired and displayed with Agilent OpenLab CDS ChemStation Edition C.01.10 (Agilent Technologies, Waldbronn, Germany).

1.3. HPLC-ESI-mass spectrometry (Exactive)

An Exactive hybrid quadrupole-orbitrap (Thermo Fisher Scientific GmbH, Bremen, Germany) coupled with an analytical HPLC 1200 Infinity system (Agilent Technologies, Waldbronn, Germany) was used. An HPLC column (Poroshell 120, EC-C18, 50 × 2.1 mm, 2.7 µm, Agilent Technologies, Waldbronn, Germany) enabled the sample separation and was eluted by a linear gradient using water plus 0.1% (v/v) formic acid as phase A and acetonitrile plus 0.1% (v/v) formic acid as phase B (all mobile phases mentioned later in this study are for this A and B phase). The general method developed was started at 5% B for 1 min, 5-100% B over 9 min, followed by an isocratic gradient of 100% B for 2 min. The column was re-equilibrated with 5% B for an additional 3 min. The injection volume was 2-20 µL and the flow rate was set to 0.3 mL/min. The drawing and ejection speed was both set to 200 µL/min. The ESI source parameters were set as follows: mass range from *m/z* 200 to *m/z* 2,000 (MS: enhanced resolution at 25,000, full, positive). The MS data derived from the Exactive were acquired with Xcalibur 2.1 (Thermo Fisher Scientific GmbH, Bremen, Germany), displayed and analyzed with Freestyle 1.8 SP2 (Thermo Fisher Scientific GmbH, Bremen, Germany).

1.4. HPLC-ESI-mass spectrometry (LTQ-Orbitrap)

An LTQ-Orbitrap XL hybrid ion trap-orbitrap (Thermo Fisher Scientific GmbH, Bremen, Germany) coupled with an analytical HPLC 1290 Infinity system (Agilent Technologies, Waldbronn, Germany) was used. An HPLC column (Poroshell 120, EC-C18, 50 × 2.1 mm, 2.7 µm, Agilent Technologies, Waldbronn, Germany) enabled the sample separation. The general method developed was started from 5-100% B over 6 min, followed by an isocratic gradient of 100% B for 2 min. The column was re-equilibrated with 5% B for an additional 2 min. The injection volume was 2-10 µL and the flow rate was set to 0.5 mL/min. The drawing and ejection speed was set to 100 µL/min and 400 µL/min, respectively. The ESI source parameters were set as follows: product ion spectra were recorded in data-dependent acquisition (DDA) mode with a mass range from *m/z* 180 to *m/z* 2,000 (MS1: FTMS, normal, resolution=60,000, full, positive; MS^2: FTMS, normal, resolution=30,000, positive). An auxiliary gas flow of 10 units, capillary temperature of 270 °C, capillary voltage of 1/- 35 V, sheath gas flow of 45 units, and source voltage of 4,000/5,000 V were used. The parameter for the DDA mode was set as follows: activation type: CID, minimum signal required: 10,000, isolation width: *m/z* 2.00, normalized collision energy: 35.0, default charge state: 2, activation Q: 0.250, and activation time: 30 ms. The dynamic exclusion enabled was set as follows: repeat count: 3, repeat duration: 30 s, exclusion list size: 50, and exclusion duration: 180 s. For MS/MS fragmentation, the two most intensive precursors per MS1 were selected for subsequent collision-induced dissociation (CID). The MS and MS/MS data derived from the LTQ-Orbitrap XL were acquired with Xcalibur 2.2 (Thermo Fisher Scientific GmbH, Bremen, Germany), displayed and analyzed with Freestyle 1.8 SP2 (Thermo Fisher Scientific GmbH, Bremen, Germany). Based on daily measurements using a reference compound, the mass accuracy of the Orbitrap instrument was within 10 ppm in positive ion mode and within 50 ppm in negative ion mode.

1.5. HPLC-ESI-mass spectrometry (Q-TOF)

A 6530 Accurate-Mass Quadrupole Time-of-Flight (Q-TOF) LC/MS (Agilent Technologies, Waldbronn, Germany) was used to determine the accurate masses of proteins purified and to monitor the enzymatic assays. The Q-TOF was attached to an Agilent 1260 Infinity HPLC system and equipped with an HPLC column (Poroshell 120, EC-C4, 50×2.1 mm, 2.7 µm, Agilent Technologies, Waldbronn, Germany). The gradient was started from 5% to 100% B over 20 min, followed with an isocratic gradient of 100% B for 3 min. The column was re-equilibrated with 5% B for an additional 3 min. The injection volume was 2-5 µL and the flow rate was set to 0.5 mL/min. Other parameters were set as follows: positive mode, MS absolute threshold at 200 counts, MS relative threshold at 0.01 %, mass range from *m/z* 200 to *m/z* 3,200, scan rate at 1 spectra/sec, gas temperature to 300 °C, gas flow to 8 L/min, nebulizer to 35 psi, sheath gas temperature to 350 °C, sheath gas flow to 11 L/min, capillary voltage to 3,500 V, nozzle voltage to 1,000 V, fragmentor to 175 V, skimmer to 65 V. The drawing and ejection speed was both set as 200 µL/min. The MS data derived from the Q-TOF were acquired with MassHunter LC/MS Data Acquisition B.06.01 (Agilent Technologies, Waldbronn, Germany), displayed and analyzed with MassHunter Qualitative Analysis B.06.00 (Agilent Technologies, Waldbronn, Germany).

1.6. Nuclear magnetic resonance (NMR) spectroscopy

1D- and 2D-NMR spectra were acquired on a Bruker Avance III 700 MHz spectrometer (700 MHz for ^{1}H, 176 MHz for ^{13}C and 71 MHz for ^{15}N, respectively) with a 5 mm TXI probe (Bruker, Karlsruhe, Germany) at 298 K. ^{1}H-NMR spectra of synthetic SNAc thioesters were

acquired by a Bruker Avance III 500 MHz spectrometer with a 5 mm BBI broadband inverse probe (1H/19F, BB, Z-gradient, ATM) (Bruker, Karlsruhe, Germany) at 298 K. The ^{1}H and ^{13}C NMR chemical shifts were referenced to the solvent peaks at 2.50 ppm (^{1}H) and 39.5 ppm (^{13}C) when DMSO-d_6 was utilized as solvent, and 4.79 ppm (^{1}H) for residual H_2O when D_2O was employed. TopSpin 3.5 (Bruker, Karlsruhe, Germany) was used for data acquisition and TopSpin 4.1.4 (Bruker, Karlsruhe, Germany) for data processing. The acquisition parameters were set as follows in Supplementary Table 15.

2. Fermentation and isolation of AFC-BC11

2.1. Cultivation of *Burkholderia orbicola* Mc0-3 and production of AFC-BC11

B. orbicola Mc0-3 was grown on 52 square Petri dishes (24.5 cm) containing PDA medium at 28 °C for 48 h. The bacteria were pelleted by centrifugation at 3,000 rpm for 45 min. The pellets were frozen in liquid nitrogen and extracted with 20 mL of 80 % acetone in water to recover the soluble part. After evaporation of acetone, the remaining part was washed twice with water and 71 mg of dry crude extract was obtained. Subsequently, 9 mg of crude extract was resuspended in 200 µL of DMSO followed by centrifugation at 15,000 rpm and 20 °C for 10 min (Hermle Z233 M-2 Microliter Centrifuge). The supernatant was fractionated via a Sunshell C18-WP column (ChromaNik Technologies Inc., 100×4.6 mm, 2.6 µm). The gradient started with 35% B for 10 min, 35 to 60% B over 8 min, 60 to 100% B over 2 min, and finished with an isocratic gradient of 100% B for 1 min and 35% B for re-equilibration. The injection volume was 50 µL. The flow rate was 1 mL/min and UV monitoring was at λ = 214, 254, 280, 320 and 360 nm. Fractions were analyzed by LTQ-Orbitrap with the general method for AFC-BC11.

2.2. Purification of AFC-BC11 for structure elucidation

Crude extract (71 mg in total) was resuspended in 7.6 mL of DMSO followed by centrifugation at 4,000 rpm and 20 °C for 10 min (Eppendorf® Centrifuge 5810R). The supernatant obtained was separated via a Grom-Sil 120 ODS-5 ST column (Grace, 250×20 mm, 10 µm). The gradient started with 40% B for 15 min, 40 to 60% B over 5 min, 60 to 100% B over 2 min, and finished with an isocratic gradient of 100% B for 3 min and 40% B for re-equilibration. The flow rate and UV monitoring were set to 20 mL/min and 320 nm, respectively. The injection volume was 700 µL. After evaporation and lyophilization AFC-BC11 (1.8 mg) was obtained and dissolved in 600 µL of DMSO-d_6 for NMR measurements. The parameters for NMR acquisition were set as outlined in Supplementary Table 15. All procedures required protection from light. To minimize light exposure, we pulled down the blinds and turned off the lights in the lab, relying only on the weak illumination for lab work. Additionally, the automatic liquid sampler and fraction collector of the HPLC were shielded from light by an external cabinet. When necessary, round-bottom flasks and vials were covered with aluminum foil.

2.3. Enantiomer analytics of DHLys with Marfey's reagent

Pure AFC-BC11 (0.2 mg) was hydrogenated with Pd/C (0.1 mg, tetrahydrofuran, 16 hours) followed by hydrolysis with stirring in 200 µL of 6 N HCl at 110 °C for 12 h. The lyophilized hydrolysates were resuspended in 50 µL of H_2O and equally divided into two portions A and B. Portion A was treated with 10 µL of 1 M $NaHCO_3$ and L-FDLA (50 µL of a 10 mg/mL solution in acetone), and the mixture was stirred at 37 °C for 1 h. The reaction was quenched with 10 µL of 1 N HCl and diluted with MeOH up to 200 µL.[1] Portion B was treated with D-FDLA, and authentic standards of L-Lys and D-Lys were treated with Marfey's reagents to yield L-FDLA and D-FDLA derivatives following the same protocol. Sample analysis was carried out with LTQ-Orbitrap in negative ionization mode with an optimized gradient as started from 10-65% B over 30 min, 65-100% B over 0.5 min, and finished with 100% B for 3 min and 10% B for re-equilibration.

2.4. Growth curve determination of *B. puraquae* DSM 103137

100 µL of cryo-stock of *B. puraquae* DSM 103137 was inoculated in 100 mL of PDB in a 500 mL Erlenmeyer flask with three baffles. The pre-cultivation was carried out at 160 rpm and 30 °C for 48 h (OD_{600} to 1.8). Subsequently, 2.7 mL, 5.4 mL and 8.1 mL of the pre-culture broth was inoculated in 97.3 mL, 94.6 mL and 91.9 mL of PDB in a 500 mL Erlenmeyer flask with three baffles to get the initial OD_{600} at 0.05, 0.10 and 0.15, respectively. The bacteria were grown at 140 rpm and 30 °C. All cultivation was performed in duplicate. 100 µL of culture each was mixed with 900 µL of PDB (dilution factor 10) for OD_{600} measurements after 3, 6, 9, 12, 15, 18, 21, 27, 30, 33, 36, 48, 54, 60, 72, 78, 84, 96, 102, and 120 h. Diluted culture broth (1 mL) was centrifugated at 15,000 rpm and 4 °C for 10 min (Hermle Z233 M-2 Microliter Centrifuge). The supernatant obtained was prepared for MS analysis with Exactive with the general method developed. Origin 2022 SR1 9.9.0.225 was used to obtain the growth curve and AFC production.

2.5. Optimized production and purification of AFC-BC11

The pre-cultivation of *B. puraquae* DSM 103137 was carried out to reach OD_{600} to 1.1-1.2. Eighteen 2 L Erlenmeyer flasks with three baffles containing 1 L of PDB each were inoculated with 30 mL of the pre-culture broth and the bacteria were grown at 120 rpm and 30 °C for 96 h. The culture broth was centrifugated at 4,000 rpm and 4 °C for 10 min (Beckman Coulter, Avanti J-26 XP with rotor JLA 8.1). The supernatant obtained was extracted with butanol (1:0.5, v/v) twice. For purification, 48.8 g of dry butanol extract was washed with 732 mL of 80% acetone (1:15, m/v), followed by sonication and centrifugation at 4,000 rpm and 4 °C for 10 min (Eppendorf® Centrifuge 5810R). The deep brown supernatant was pooled together. After evaporation of acetone, the solution was frozen at –80 °C and lyophilized for 12 h. The dry crude extract (32.5 g) was resuspended in 162.5 mL of DMSO (1:5, m/v) and followed by sonication and centrifugation at 4,000 rpm and 20 °C for 10 min (Eppendorf® Centrifuge 5810R). The supernatant separation was carried out using an Agilent 5 Prep-C18 column (50×30 mm, 5 µm, Agilent Technologies). The gradient was from 30 to 40% B over 13 min, from 40 to 100% B over 0.1 min, and finished with an isocratic gradient of 100% B for 2 min. The column was re-equilibrated with 30% B for an additional 2 min. The flow rate was set to 20 mL/min and UV absorbance was monitored at λ = 320 nm. The injection volume was 5 mL. All fractions containing AFC-BC11 were pooled and 28.7 mg of pure AFC-BC11 was obtained after evaporation and lyophilization.

3. Photoisomerization

3.1 Photoirradiation experiments

Handheld UV lamps with bulbs emitting wavelength of 366 nm (6 W) and 254 nm (6 W) (UV-Analysenleuchte Typ UVAC-6U, M&S Laborgeräte GmbH), and a 300-nm LED (32 mW) (M300L4, Thorlabs) controlled by a T-Cube LED driver (LEDD1B, Thorlabs) were used. Pure AFC-BC11 (0.1 mg) was dissolved in 1 mL of DMSO. The solution was aliquoted in four vial inserts with 100 µL each (Screw neck vial 702282/Micro-insert 702813, Macherey-Nagel) and irradiated under daylight, 366, 254 and 300 nm at room temperature, respectively. The HPLC chromatograms were recorded after irradiation for 0, 5, 15, 30 min, 1, 2, 5, 7, 9 and 12 hours. Separation of analytes was run on a Fortis C18 column (Fortis Technologies Ltd., 150×4.6 mm, 5 µm) with a linear gradient starting with 35% B for 10 min, 35 to 60% B over 8 min, 60 to 100% B over 2 min, finishing with 100% B for 1 min and 35% B for re-equilibration. The injection volume was 5 µL and the flow rate was 1 mL/min. UV monitoring was at λ = 320 nm. The quantification of photoisomers was done via peak area integration. MS spectra of photoisomers were acquired with LTQ-Orbitrap with an optimized gradient as started from 5-100% B over 20 min, 100% B for 2 min and 5% B for re-equilibration.

3.2 Structure elucidation of the AFC-BC11 photoisomer

Pure AFC-BC11 (1.6 mg) was dissolved in 600 µL of DMSO-d_6 in NMR tube (Norell®, ST500-7) and irradiated at λ = 366 nm and room temperature for 45 min. Irradiated sample (5 µL portion) was analyzed with analytical HPLC as described above. The parameters for NMR measurements were set as outlined in Supplementary Table 15. The NMR data acquired were visualized with CcpNmrAnalysis 3.1.1.[2]

4. Biological testing

4.1. Photoisomerized AFC-BC11 for biological testing

Pure AFC-BC11 (0.8 mg) was dissolved in 8 mL of DMSO. The solution was subjected to continuous irradiation at λ = 366 nm and room temperature for 0, 5, 15 and 30 min. At each time point, 2 mL of the sample solution was transferred into a brown vial and stored at –20 °C in the dark. A 5 µL aliquot of each sample was analyzed using the aforementioned analytical HPLC method. The HPLC analysis was conducted in triplicate and the peak areas acquired at λ = 320 nm were integrated for quantification.

4.2. Antifungal assays with phytopathogenic fungi

The biological assays were conducted on five phytopathogenic fungi (Supplementary Table 11). Non-photoisomerized and photoisomerized (see above; irradiation time 0, 5, 15 and 30 min) AFC-BC11 samples and azoxystrobin (standard control) in a concentration of 100 µg/mL each were diluted with DMSO (AFC-BC11) or MeOH (azoxystrobin) to obtain mother stocks of various concentrations (10 to 40 µg/mL). These mother stocks were further diluted in the final growth PDB media to obtain concentration ranges as follows: for *C. kahawae* CM732 (0.08-0.96 µg/mL), *F. xylarioides* CAB003 (0.4-2.4 µg/mL), for *P. oryzae* Guy11 (0.08-0.96 µg/mL), for *P. teres* f. *teres* Hun0005 (0.08-0.96 µg/mL), and for *R. solani* CD9001 (0.08-1.2 µg/mL). For each fungal strain, a small portion of mycelium was crushed 4 times for 20 seconds in water using a grinder (MP Biomedicals FastPrep-24 homogenizer Classic). The resulting crushed mycelium was diluted in PDB at an OD_{600} = 0.05. The antifungal assay was carried out in a 96-well microplate (Thermo Scientific NunclonTM Delta Surface) where each well contained 200 µL of PDB, 25 µL of the diluted AFC-BC11 sample or control following the range initially prepared and 25 µL of fungal crushed mycelium. The plates were sealed with plastic tape (Parafilm®) and incubated in the dark at 28°C for 3 days (*R. solani*) and 7 days (*C. kahawae*, *F. xylarioides*, *P. oryzae* and *P. teres* f. *teres*), respectively. The solvent control was the inoculation of each fungus in presence of DMSO or MeOH, while the negative control was an empty well containing only PDB. The antifungal effect was initially assessed visually and then by measuring the optical density (λ = 600 nm) in each well using a plate reader (Tecan Spark Machine). MICs were defined as the lowest concentration of AFC-BC11 samples or azoxystrobin to inhibit the growth of the tested fungus. At least three replicates were performed for each sample and each fungus.

4.3. Antifungal assays with human pathogenic fungi

In vitro antifungal susceptibility of pure AFC-BC11 against 9 clinically relevant fungal species representing the main taxa of pathogenic fungi (Supplementary Table 11) was tested in comparison to the approved antifungal drug voriconazole (VCZ; Pfizer Inc., Peapack, NJ, USA) using broth microdilution technique following the European Committee on Antimicrobial Susceptibility Testing (EUCAST) standard methodology for yeasts or filamentous fungi respectively.[3,4] In contrast to the EUCAST protocol, microdilution plates were prepared by twofold serial dilutions of the antifungal agents. Filamentous fungi were grown on malt extract agar (MEA) for 2-7 days at 35 °C and yeasts were cultivated on yeast extract peptone dextrose agar (YPD) for 24 h. Spore or yeast cell suspensions were counted with a hemocytometer. Minimum inhibitory concentrations (MIC) endpoints of filamentous fungi were defined as 100% reduction in growth and were determined visually using a mirror after 48 hours of incubation at 35 °C. Microdilution plates of yeasts were read with a microdilution plate reader (Infinite® M Nano plus, Tecan) and MIC endpoints were defined as the lowest drug concentration giving inhibition of growth of ≥50% of that of the drug-free control. Since AFC-BC11 is light sensitive tests were performed in low light and the inoculated test plates were wrapped in aluminum foil. *A. fumigatus* ATCC 204305 and *Candida parapsilosis* ATCC 22019 were used as reference strains.

4.4. Antibacterial assays

Non-photoisomerized and photoisomerized (irradiation time 0, 5, 15 and 30 min) AFC-BC11 samples in a concentration of 100 µg/mL were diluted with DMSO to generate a solution of 64 µg/mL for each, which was further diluted by serial 1:2 dilution with DMSO, achieving standard stock solutions with a wide concentration range from 64 to 0.125 µg/mL. Broth microdilution assays were performed to determine minimal inhibitory concentration (MIC) values according to the ninth edition of the Approved Standard M07-A9. The test

was carried out for three gram-negative strains and three gram-positive strains (Supplementary Table 11) in 96-well microplates (Polystyrene, F-bottom, 655161, Greiner). 20 µL of cryo-stock of each strain were inoculated in 20 mL of LB followed by an overnight incubation at 200 rpm and 37 °C. The test inoculum was adjusted by the 0.5 McFarland Standard (OD_{625} from 0.08 to 0.10, OD_{625} = 0.10 is equivalent to 1×108 CFU/mL). Within 15 min of preparation, the adjusted inoculum suspension was diluted in MHBII so that each well contained approximately 5 × 10^5 CFU/mL in a final volume of 100 µL. 95 µL of the inoculum were applied per well and 5 µL of diluted standard stock solutions were added to reach final concentrations of 3.2 to 0.00625 µg/mL. One column of each well plate served as a growth control only with 100 µL of the inoculum suspension and another column served as sterility control (100 µL of MHBII). The antimicrobial effect of solvent was tested by adding 5 µL of DMSO to several wells. Ciprofloxacin solution was added in one row as positive control. Each tray was sealed with plastic tape (Parafilm®). Microdilution trays of *E. coli* DSM1116, *E. coli* BW25113, *S. typhimurium* TA100, and *B. subtilis* DSM10 were incubated at 37 °C in the dark for 20 h but trays of *M. luteus* DSM1790 and *M. phlei* DSM750 at 30 °C. Assays were performed in duplicate and trays were subsequently analyzed by naked eye.

5. Bioinformatic analysis

5.1. Basic local alignment search tool (BLAST)

The *afc* gene conservation in *Burkholderia* spp. was analyzed based on BlastN tool screening for the presence of *afcA* (BCENMC03_RS32465), *afcQ* (BCENMC03_RS32525) and *afc* BGC (NC_010512.1, location 545185-572489), respectively. The parameters were set as follows: nucleotide collection (nr/nt) as standard database, organism to *Burkholderia* (taxid:32008) or *Burkholderia cepacia* complex (taxid:87882), program selection to megablast, except threshold to 0.05, word size to 28, max matches in a query range to 0, match/mismatch scores to 1/-2, gap coats as linear and query coverage to 50%. The multiple gene alignment was visualized with Easyfig 2.2.6.[5]

5.2. Phylogenetic analyses

The evolutionary history was inferred using the Neighbor-Joining method.[6] The bootstrap consensus tree inferred from 1,000 replicates was taken to represent the evolutionary history of the taxa analyzed. Branches corresponding to partitions reproduced in less than 50% bootstrap replicates were collapsed. The percentage of replicate trees in which the associated taxa clustered together in the bootstrap test (1,000 replicates) were shown next to the branches.[7] The evolutionary distances were computed using the p-distance method and were in the units of the number of amino acid differences per site.[8] All ambiguous positions were removed for each sequence pair (pairwise deletion option). Evolutionary analyses were conducted in MEGA11.[9,10] The phylogenetic tree was visualized and annotated with an online tool iTOL.[11]

5.3. Multiple sequence alignment (MSA)

The multiple sequence alignment was generated using Clustal Omega, an online tool that utilizes ClustalW with character counts as output format.[12] The default parameters were used for the alignment. The resulting alignment was analyzed and visualized using Jalview.[13] Alternatively, MSA was carried out for the selected acyltransferases using PROMALS3D with the default parameters.[14] To identify homologous proteins, the Foldseek Search Server (database: PDB100 20240101; mode: 3Di/AA) was used, which detected similarity between protein structures by three-dimensional superposition.[15]

5.4. Protein structure prediction

The 3D structures were predicted using AlphaFold2 by means of ColabFold v1.5.2.[16–20] The modes and parameters of prediction used were by default and molecular graphics and analyses were performed with UCSF ChimeraX 1.6.1.[21,22]

5.5. Docking studies on the adenylation domains (AfcQ, FlvN, DptA3) and desaturase AfcC

Protein structures, which have been fetched directly from PDB or predicted using AlphaFold2, were prepared for docking by means of AutoDock Tools 1.5.7.[23] The ligands were prepared with OpenBabel 3.1.1 and AutoDock Tools 1.5.7.[24] The setting up of grid options is described as follows, FlvN (center_x = -0.696, center_y = -3.134, center_z = -2.384, spacing = 1.000, size_x = 40.0, size_y = 40.0, size_z = 40.0), AfcQ (center_x = -0.344, center_y = -1.852, center_z = -2.453, spacing = 1.000, size_x = 15.0, size_y = 15.0, size_z = 15.0), DptA3 (center_x = -1.568, center_y = 0.002, center_z = 3.559, spacing = 1.000, size_x = 10.0, size_y = 10.0, size_z = 10.0), and AfcC (center_x = -4.558, center_y = -0.779, center_z = 0.666, spacing = 0.225, size_x = 40.0, size_y = 40.0, size_z = 40.0). Docking was lunched with AutoDock Vina 1.1.2.[25]

6. Molecular biology

6.1 Genomic DNA isolation and cloning of plasmids

Genomic DNA of *B. orbicola* Mc0-3 was isolated with the NucleoSpin TissueTM Kit (Macherey-Nagel). Genes were amplified by PCR from chromosomal DNA with primers shown in Supplementary Table 16 and cloned via Gibson assembly into expression vector pET28a_TEV (Merck KGaA).[26]

6.2 Purification of Afc proteins

Chemically competent *E. coli* BL21-Gold/DE3 cells transformed with the corresponding vector were spread on LB agar plate containing kanamycin (50 µg/mL) and incubated overnight at 37 °C. A single colony was inoculated into 20 mL of LB supplemented with kanamycin (50 µg/mL) in a 100-mL Erlenmeyer flask without baffles and cultivated overnight at 37 °C and 180 rpm. The resulting culture was inoculated into 1 L of TB with kanamycin (50 µg/mL) in two 2-L Erlenmeyer flasks with three baffles to reach an initial OD_{600} of 0.1 for fermentation at 37 °C and 180 rpm. The expression was induced by adding β-D-1-thiogalactopyranoside (IPTG, 0.2 mM) when OD_{600}

reached 0.8-1.0. The culture was further incubated overnight at 18 °C and 180 rpm. Cells were harvested by centrifugation at 5,000 rcf and 4 °C for 30 min (Beckman Coulter, Avanti J-26 XP with rotor JLA 8.1). The pellet was resuspended in lysis buffer (1:10, w/v). Additionally, $MgCl_2$ (10 mM), DNase (5 µg/mL), lysozyme and benzamidine were added. The cells were lysed using a cell homogenizer at 20 kpsi and 7 °C (Constant Systems Ltd). The lysate was centrifuged at 50,000 rcf and 4 °C for 30 min (Beckman Coulter, Avanti J-26 XP with rotor JA-25.50). The supernatant was passed through a 0.45 µm syringe filter and loaded onto a HisTrap™ HP column (Cytiva, 25×16 mm, 34 µm, 5 mL) using an ÄKTA system (ÄKTApurifier 10, GE Healthcare). The column was eluted by a linear gradient started with 100% wash buffer for 8 min, followed with 3% elution buffer for 4 min and 50% for 4 min, and finished with 100% elution buffer for 8 min. The flow rate was set to 5 mL/min. Fractions of interest were collected to increase protein concentration using centrifugal concentrator (Amicon® Ultra, Merck) at 4,000 rpm and 4 °C (Eppendorf® Centrifuge 5810R). Subsequently, size-exclusion chromatography was performed with a HiLoadTM 16/60 SuperdexTM 200 pg column (Cytiva, 120 mL) to remove residual imidazole and protein contaminations with protein buffer. The chromatograms were recorded with Unicorn v5.20 (ÄKTApurifier 10, GE Healthcare) and the flow rate was set to 1 mL/min. SDS-PAGE (12%) and Coomassie staining was used to identify fractions containing recombinant proteins. These fractions were collected and further concentrated. The final concentration of protein was determined with NanoPhotometer® P 330 (Implen). Protein concentrations were calculated from absorption at λ = 280 nm, using extinction coefficients calculated from their respective amino acid sequences. Aliquots of protein samples were snap-frozen in liquid nitrogen and stored at –80 °C for further applications. The image of the gel was acquired with the software argusX1 v7.9.7 (Biostep) and a scanner system (ViewPix 900 based on Epson scanner technology).

6.3 Purification of Sfp protein

The phosphopantetheinyl transferase Sfp (*Bacillus subtilis* spp. *spizizenii* ATCC 6633) used in this study was purified in-house from pET15b_WT_sfp vector in *E. coli* BL21-Gold/DE3, cultivated in TB supplemented with ampicillin (100 µg/mL). The expression was induced by adding IPTG (1 mM) until OD_{600} reached 0.6. The culture was further incubated overnight at 18 °C and 180 rpm. After cell harvest and disruption, the purification with Ni-NTA affinity chromatography was carried out using the similar methods described above, but with different buffers (Supplementary Table 13).

6.4 Cloning of constructs for in-frame deletion mutants

In-frame deletion mutants of *B. pyrrocinia* DSM 10685 were created by conjugation and following selection of mutants with kanamycin. For this purpose, the kanamycin resistance cassette (Km^R) of vector pET28a_TEV was cloned via Gibson assembly into the mobilizable vector p18mobapra[27] to generate p18mobKm^R. For the cloning of in-frame deletion constructs the upstream and downstream regions of the target *afc* genes were cloned into p18mobKm^R via Gibson assembly. The primers used for this step are shown in Supplementary Table 17. During the ongoing work it was observed that double crossover events in *B. pyrrocinia* DSM 10685, generated with p18mobKm^R based deletion constructs, were too rare, so that it was switched to the pGPI-SceI-based homologous recombination mutagenesis system.[28] For this, the trimethoprim resistance-S1 nuclease cleavage site cassette (Tp^R) of pGPI-SceI (gift from Miguel Valvano; Addgene plasmid # 32060) was amplified by PCR and cloned via Gibson assembly into the p18mobKm^R in-frame deletion constructs used for conjugation.

6.5 Conjugation and generation of in-frame deletion mutants

B. pyrrocinia DSM 10685 was cultivated in 20 mL of LB overnight at 30 °C. The 2,6-diaminopimelic acid (DAP) auxotrophic *E. coli* DSM 113367, carrying the in-frame deletion construct, was grown overnight in 20 mL of LB supplemented with 50 µg/mL trimethoprim (Tp) and 0.3 mM DAP at 37 °C. 1 mL of each of the overnight cultures of *B. pyrrocinia* and *E. coli* donor strain were mixed, centrifuged, washed with 1 mL of LB, centrifuged again and the resulting pellet was plated on LB supplemented with 0.3 mM DAP. After overnight incubation at 30 °C cells were scraped in 2 mL of LB from the plate and dilutions were spread onto LB plates supplemented with 150 µg/mL Tp. After incubation for 72 h at 30 °C, genomic DNA of a trimethoprim-resistant single colony was isolated as described above and checked by PCR for a single crossover event. A correct single crossover mutant was transformed with replicative vector pAH25-SceI, expressing the I-SceI nuclease.[29] The single crossover mutant was cultivated overnight at 30 °C in 20 mL of LB supplemented with 150 µg/mL Tp. The donor strain *E. coli* DSM113367, carrying pAH25-SceI, was grown overnight at 37 °C in 20 mL of LB supplemented with 34 µg/mL chloramphenicol (Cm) and 0.3 mM DAP. Conjugation procedure was carried out as described above. After overnight incubation at 30 °C cells were scraped in 2 mL of LB from plate and 300 µL of scraped cells were plated onto a LB plate supplemented with 100 µg/mL Cm. After incubation for 72 h at 30 °C cells were scraped off again and directly inoculated in 50 mL of M9 supplemented with 100 µg/mL Cm.[30] The expression of the I-SceI nuclease at this experimental stage leads to a higher chance for a double crossover event. After 72 h incubation at 30 °C dilution of cells was plated onto LB supplemented with 100 µg/mL Cm. Resulting colonies were screened according to phenotype Tp^SCm^R and checked by PCR for the double crossover event. Correct mutants were incubated in 50 mL of LB for 48 h at 30 °C to induce the loss of pAH25-SceI. After this, cells were plated onto LB and screened for Tp^SCm^S phenotype. The resulting final in-frame deletion mutants were checked for AFC-BC11 production.

6.6 Metabolic profiling of *afc* gene deletion mutants

Cryo-stocks of the mutant of interest (50 µL) were inoculated in 20 mL of LB in a 100-mL Erlenmeyer flask without baffles. The pre-cultivation was carried out overnight at 160 rpm and 30 °C. 300-mL Erlenmeyer flasks with three baffles containing 50 mL of PDB were inoculated with 500 µL of the pre-culture broth and the bacteria were grown at 160 rpm and 30 °C for 96 h. The culture broth was centrifugated in a 50-mL falcon tube at 4,000 rpm and 4 °C for 10 min (Beckman Coulter, Avanti J-26 XP with rotor JLA 8.1). The supernatant was divided in two 50-mL falcon tubes and each was extracted with 10 mL of butanol, followed with incubation at room temperature for 30 min and centrifugated at 4,000 rpm and 4 °C for 10 min. The supernatants were combined and the sample was dried by Speed Vac (SAVANT SPC131DDA, Thermo Scientific). The pellet was resuspended in 150 µL of DMSO followed by sonication and centrifugation at 4,000 rpm and 20 °C for 10 min. The supernatants were analyzed with an LTQ-Orbitrap mass spectrometer.

7. *In vitro* enzymatic assays

7.1. AfcQ substrate activation assay

Hydroxylamine release assay: the adenylation activity of AfcQ was measured with a continuous assay according to published procedures.[31,32] AfcQ-catalyzed substrate activation was tested in individual incubations of 25 amino acids and 3 citric acid analogues (Supplementary Table 14). The substrates were pre-dissolved in 100 mM Tris-HCl pH 8.0 at a stock concentration of 100 mM. The assay pre-mix with a volume of 95 µL contained: 50 µL of 2× adenylation buffer, 20 µL of 1 mM 2-amino-6-mercapto-7-methylpurine ribonucleoside (MesG, EnzChek™ Pyrophosphate Assay Kit E6645, ThermoFisher), 5 µL of 100 mM ATP, 1 µL of 100 mM tris (2-carboxyethyl) phosphine (TCEP), 7.5 µL of 2 M hydroxylamine, 1 µL of 40 U/mL inorganic pyrophosphatase (IP, Merck), 1 µL of 100 U/mL purine nucleoside phosphorylase (PNP, EnzChek™ Pyrophosphate Assay Kit E6645, ThermoFisher), 0.3 µL of 588.3 µM AfcQ and 9.2 µL of Milli-Q water, and was incubated at room temperature for 15 min. Subsequently, the pre-mix solution was incubated with 5 µL of 100 mM substrate and placed in one well of a 96-well plate. The final concentrations of AfcQ and of the substrate were 2 µM and 5 mM, respectively. The UV absorption was monitored by a multimode microplate reader (Tecan Infinite M200, Männedorf, Switzerland). The parameters were set as follows: temperature at 22 °C, shaking (duration: 15 s, amplitude: 2 mm, mode: orbital), wait time to 5 s, kinetic cycle (duration: 30 min, wait time: 1 s), absorbance (wavelength: 360 nm, number of flashes: 5, settle time: 10 ms). The data were acquired with i-controlTM 1.6 microplate reader. The pathlength of each well was 0.2972 cm and the extinction coefficient of MesG was set to 11,000 $M^{-1}cm^{-1}$. Reactions with addition of 5 µL of 100 mM Tris-HCl pH 8.0 were used as controls. All experiments were measured in triplicate.

Determination of kinetic parameters of AfcQ (k_{cat} and K_m): reactions, containing 2 µM AfcQ, L-Asp (0, 5, 12.5, 25, 50, 100, 250, 500, 1,000, 2,000 and 5,000 µM), 0.2 mM MesG, 5 mM ATP, 1 mM TCEP, 150 mM hydroxylamine, 0.04 U IP, and 0.10 U PNP, were prepared in 50 µL of adenylation buffer 2x. Kinetic parameters were deduced by non-linear regression analysis based on Michaelis–Menten kinetics using the program Origin 2022 SR1 9.9.0.225. All reactions were performed in triplicate.

7.2. AfcA substrate activation assay

Hydroxylamine release assay: 62 carboxylic acids, including 34 fatty acids, 25 amino acid, and 3 citric acid analogues, were tested (Supplementary Table 14). The stock solutions of hydrophobic fatty acids (chain length C3-C19) were prepared in DMSO at a stock concentration of 1 mM. The final concentration of AfcA and the substrate were 2 µM and 5 mM (50 µM for hydrophobic fatty acids), respectively. Reactions added with 5 µL of 100 mM Tris-HCl pH 8.0 or 5 µL of DMSO were performed as controls. All assays were done in triplicate under the same conditions as for AfcQ.

Mass spectrometric detection of myristoyl-*O*-AMP formation: a 100-µL reaction mixture, containing 2 µM AfcA, 100 µM myristic acid (C14:0), 5 mM CoA, and 5 mM ATP, was incubated in 50 µL of adenylation buffer 2x at 30 °C for 1 h. AfcA was deactivated at 95 °C for 5 min as a negative control. The reactions were quenched by adding 100 µL of MeOH and then centrifugated at 4 °C and 14,000 rpm for 5 min (Eppendorf® Centrifuge 5415R). The supernatant obtained was processed for LTQ-Orbitrap measurements with the general method developed but in negative ionization mode.[33]

7.3. Preparation of *holo*-AfcK

Apo-AfcK was converted into the *holo*-form by Sfp-mediated transfer of 4'-phosphopantetheine from coenzyme A.[34,35] A 100-µL reaction mixture, comprising 200 µM *apo*-AfcK, 10 µM Sfp, and 1 mM CoA (coenzyme A, trilithium salt, 94%, Calbiochem®, Sigma-Aldrich), was incubated in HEPES buffer at 30 °C for 1 h. Subsequently, excess CoA was eliminated through serial dilutions using adenylation buffer 1x and 3K MWCO centrifugal concentrators (Amicon® Ultra - 0.5 mL Centrifugal filters, Merck Millipore) at 14,000 rpm and 4 °C for 10 min (Eppendorf® Centrifuge 5415R). This dilution procedure was repeated three times, resulting in a final volume of 150 µL (dilution factor 1.5). Phosphopantetheinylation was verified by ESI-Q-TOF-MS analysis.

7.4. AfcQ-catalyzed mono-loading onto *holo*-AfcK with L-Asp

A 100-µL reaction mixture containing 120 µM *holo*-AfcK, 2 µM AfcQ, 5 mM L-Asp, and 5 mM ATP was incubated in 50 µL of adenylation buffer 2x at 30 °C for 30 min.[34,35] Alternatively, a 100 µL reaction mixture with 20 µM *holo*-AfcK, 2 µM AfcA, 50 µM myristic acid (C14:0), and 5 mM ATP was prepared. AfcQ and AfcA were deactivated at 95 °C for 5 min as negative controls. Successful loading of substrate was verified by ESI-Q-TOF-MS analysis.

7.5. AfcQ-catalyzed iterative loading onto *holo*-AfcK with L-Asp

A 700-µL reaction mixture containing 120 µM *holo*-AfcK, 2 µM AfcQ, 5 mM L-Asp, and 5 mM ATP was incubated at 30 °C for 30 min. 100 µL of sample was then used as a reference (vial A). Subsequently, the excess L-Asp and ATP were removed from the remaining 600-µL mixture with 4-mL 3K MWCO centrifugal concentrators (Amicon®, Merck Millipore) at 4,000 rpm and 4 °C for 25 min (Eppendorf® Centrifuge 5810R). Four additional reaction systems were prepared as follows, vial B: 100 µL of the reaction mixture, vial C: addition of 5 µL of 100 mM ATP, vial D: addition of 5 µL of 100 mM L-Asp, vial E: addition of 5 µL of 100 mM ATP and 5 µL of 100 mM L-Asp. Vials A-E were incubated at 30 °C for 24 h. In addition, 0.34 µL of 588.3 µM AfcQ was added to the remaining mixture in vial A followed by incubation at 30 °C for an additional 30 min. Successful loading of substrate was verified by ESI-Q-TOF-MS analysis.

7.6. Decarboxylation assays with AfcP

H_2N-L-Asp-γ-*S*-AfcK as substrate: loading of isotopically labelled L-Asp($^{13}C_4$) on *holo*-AfcK was performed as described above. Subsequently, the excess L-Asp($^{13}C_4$) and ATP were removed with 0.5-mL 3K MWCO centrifugal concentrators. A 50-µL reaction mixture containing 64 µM L-Asp($^{13}C_4$)-loaded AfcK, 0.5 mM PLP, and 5 µM AfcP was incubated at 30 °C. Subsequently, the

decarboxylation was verified by HPLC-ESI-Q-TOF measurements after incubation for 1 h and 24 h. For one-pot reaction: a 100-µL reaction mixture containing 107 µM *holo*-AfcK, 5 mM ATP, 5 mM L-Asp($^{13}C_4$), 0.5 mM PLP, 5 µM AfcP, and 2 µM AfcQ was incubated at 30 °C. Deactivated AfcP was generated by heating at 95 °C for 5 min as a negative control. Q-TOF measurements were performed after different incubation time (1, 5, 10, 20, 40 min, 1, 2, and 24 h).

7.7. AfcQ-catalyzed loading onto H_2N-L-Asp-γ-*S*-AfcK and H_2N-(β-Ala)$_n$-*S*-AfcK with L-Asp

Apo-AfcK was converted into the *holo*-form as described above in 7.3. Subsequently, a 100-µL reaction mixture, comprising 107 µM *holo*-AfcK, 5 mM ATP, 5 mM L-Asp, and 2 µM AfcQ, was incubated in adenylation buffer at 30 °C. Meanwhile, direct enzymatic transfer of aminoacyl-phosphopantetheine to *apo*-AfcK was proceeded by incubation of 200 µM *apo*-AfcK, 10 µM Sfp, and 1 mM H_2N-(β-Ala)$_n$-*S*-CoA (n=1, 2, and 3, **9-11**) at 30°C for 1 h in adenylation buffer. Subsequently, the excess H_2N-(β-Ala)$_n$-*S*-CoA was removed using 3K MWCO centrifugal concentrators. Afterwards, 107 µM H_2N-(β-Ala)$_n$-*S*-AfcK, 5 mM ATP, 5 mM L-Asp, and 2 µM AfcQ were incubated at 30°C. The subsequent loading was verified by Q-TOF measurements after different incubation time (1, 3, 5, and 24 h).

7.8. AfcQ-catalyzed loading onto SNAc thioester substrates

Peptide bond formation catalyzed by AfcQ was evaluated with eight SNAc acceptor substrates: H_2N-(L-Asp)$_n$-SNAc (n=1, 2, 3, and 4, **1-4**) and H_2N-(β-Ala)$_n$-SNAc (n=1, 2, 3, and 4, **5-8**). A 100-µL reaction mixture containing 4 µM AfcQ, 100 µM L-Asp($^{13}C_4$) or L-Asp, 5 mM SNAc thioesters (**1-8**), and 5 mM ATP was incubated in 50 µL of adenylation buffer 2x at 30 °C for 1 h. AfcQ was deactivated at 95 °C for 5 min as negative control. Reactions were quenched by addition of an equal volume of MeOH and centrifuged at 14,000 rpm and 4 °C for 5 min (Eppendorf® Centrifuge 5415R). The supernatant was subjected to LTQ-Orbitrap measurements with the general method developed.

7.9. AfcA-catalyzed acyl-transfer onto SNAc thioester substrates

A 100-µL reaction mixture containing individual SNAc thioester (**1-8,** 5 mM), 4 µM AfcA, 100 µM fatty acid, and 5 mM ATP was incubated in 50 µL of adenylation buffer 2x. The reactions were carried out under the same conditions as for AfcQ. The tested fatty acids were C3:0, C3:0-COOH, C13:0, C13:0-COOH, C14:0, C14:0-COOH, C19:0, and C19:0-COOH.

7.10. AfcA-catalyzed acyl-transfer onto H_2N-(β-Ala)$_3$-*S*-AfcK

H_2N-L-Asp-γ-*S*-AfcK as acceptor: *apo*-AfcK was converted into H_2N-L-Asp-γ-*S*-AfcK via a two-step reaction with Sfp and AfcQ under conditions outlined above. Subsequently, 100 µL of the reaction mixture underwent incubation with 10 µL of 1 mM myristic acid (C14:0) and 1 µL of 419.2 µM AfcA at 30 °C for 1 h.

H_2N-β-Ala-*S*-AfcK as acceptor: the reaction was conducted as a one-pot reaction, comprising 200 µM *apo*-AfcK, 10 µM Sfp, 1 mM CoA, and 5 mM H_2N-β-Ala-SNAc thioester (**5**), and was incubated at 30°C for 1 h to generate H_2N-β-Ala-*S*-AfcK. Subsequently, the excess CoA and H_2N-β-Ala-SNAc were eliminated using 3K MWCO centrifugal concentrators. Following this, a 50-µL reaction mixture containing H_2N-β-Ala-*S*-AfcK, 4 µM AfcA, 100 µM myristic acid (C14:0), and 5 mM ATP was incubated at 30°C for an additional 1 h.

H_2N-(β-Ala)$_3$-*S*-AfcK as acceptor: direct enzymatic transfer of aminoacyl-phosphopantetheine to *apo*-AfcK proceeded by incubation of 200 µM *apo*-AfcK, 10 µM Sfp, and 1 mM H_2N-(β-Ala)$_3$-*S*-CoA (**11**) at 30°C for 1 h. Subsequently, the excess H_2N-(β-Ala)$_3$-*S*-CoA was removed using 3K MWCO centrifugal concentrators. Afterwards, 107 µM H_2N-(β-Ala)$_3$-*S*-AfcK, 4 µM AfcA, 100 µM myristic acid (C14:0), and 5 mM ATP were incubated at 30°C for an additional 1 h. AfcA was deactivated at 95°C for 5 min as negative controls. The acyl-transfer was verified by HPLC-ESI-Q-TOF measurements.

7.11. Acyl-transfer onto AfcL

A 100-µL reaction mixture containing individual 500 µM thioester mimic (**5-8**, **12**, and **13**) and 20 µM AfcL was incubated in 50 µL of adenylation buffer 2x at 4 °C. The successful acyl-transfer was verified by HPLC-ESI-Q-TOF measurements after incubation for 1 h and overnight. Alternatively, 125 µM each of thioester mimics (**5-8**) were mixed with 20 µM AfcL to test competitive binding to AfcL.

To test the peptide release from AfcL: a 100-µL reaction mixture containing 500 µM H_2N-(β-Ala)$_3$-SNAc (**7**), 500 µM H_2N-L-Lys-*S*-CoA (**13**) (or L-Lys, D-Lys) and 20 µM AfcL was incubated in 50 µL of adenylation buffer 2x at 4 °C for 2 h and overnight. Protein buffer (general) was used as negative control for AfcL. Subsequentially, the reaction was quenched by adding an equal volume of MeOH, followed by centrifugation at 14,000 rpm and 4 °C for 20 min (Eppendorf® Centrifuge 5415R) using 0.5-mL 3K MWCO centrifugal concentrators (Amicon® Ultra - 0.5 mL Centrifugal filters, Merck Millipore) to remove the protein. The resulting samples were analyzed by LTQ-Orbitrap using the general method developed.

To test the loading of myristic acid onto H_2N-(β-Ala)$_3$-AfcL by means of AfcA: a 200-µL reaction mixture containing 1 mM H_2N-(β-Ala)$_3$-SNAc (**7**) and 36 µM AfcL was incubated in 100 µL of adenylation buffer 2x at 4 °C for overnight. The excess H_2N-(β-Ala)$_3$-SNAc (**7**) was removed with 0.5-mL 3K MWCO centrifugal concentrators and 150 µL of concentrated reaction mixture was obtained. Subsequently, a 100-µL reaction mixture containing 500 µM ATP, 200 µM myristic acid (C14:0), 10 µM AfcA and 61.5 µL of the concentrated reaction mixture was further incubated at 4 °C for 2 h and for overnight. Protein buffer (general) was used as negative control for AfcA. Samples were analyzed by HPLC-ESI-Q-TOF measurements.

To test the loading of myristic acid onto H_2N-(β-Ala)$_3$-L-Lys-*S*-CoA by means of AfcA: a 100-µL reaction mixture containing 500 µM ATP, 125 µM H_2N-(β-Ala)$_3$-SNAc (**7**), 200 µM myristic acid (C14:0), 10 µM AfcA, 25 µM AfcL and 1 mM H_2N-L-Lys-*S*-CoA (**13**) was

incubated in 50 µL of adenylation buffer 2x at 4 °C for 2 h and overnight. MQ-water was used as negative control for H_2N-L-Lys-*S*-CoA. The acyl-transfer onto H_2N-(β-Ala)$_3$-L-Lys-*S*-CoA was verified by LTQ-Orbitrap as described above.

8. Chemical synthesis of enzyme substrates

8.1. Synthesis of Boc-(L-Asp-O*t*Bu)$_n$-OH and Boc-(β-Ala)$_n$-OH by solid phase peptide synthesis (SPPS)

To 1 g of previously swollen resin in a 20 mL syringe was added a solution of 0.600 mmol of Fmoc-L-Asp-O*t*Bu (CAS: 129460-09-0, or Fmoc-β-Ala-OH, CAS: 35737-10-1), 0.22 mL of *N*,*N*-diisopropylethylamine (DIPEA) in 3 mL of dichloromethane (DCM). The resin was shaken for 2 h and then the liquid was filtered off, and the resin was washed with 10 mL of *N*,*N*-dimethylformamide (DMF) and 10 mL of DCM. Subsequently, 10 mL of a DCM:MeOH:DIPEA (80:15:5) solution was added and the resin was shaken for an additional 20 min. Fmoc-protecting group was removed by adding 15 mL of 20% piperidine in DMF. The resin was shaken for 30 min and then washed with DMF (10 mL) and DCM (10 mL) and was ready for the next coupling. A solution of Fmoc-L-Asp-O*t*Bu (or Fmoc-β-Ala-OH, 1.20 mmol), hexafluorophosphate benzotriazole tetramethyl uronium salt (HBTU, 1.18 mmol), DIPEA (500 µL) in 6 mL of DMF was added. After shaking for 90 min, the liquid was filtered off and the resin was washed with DMF (10 mL) and DCM (10 mL). The steps for Fmoc-deprotection and coupling were repeated until getting the wanted peptide sequence. To release the peptides from resin, a solution of 25% hexafluoroisopropanol (HFIP) in DCM (15 mL) was poured over the resin and the mixture was shaken for 2 h. The resin was filtered off and washed with DCM (10 mL) for 3 times. The combined filtrate was concentrated and purified with HPLC.[36,37]

8.2. Synthesis of H_2N-(L-Asp)$_n$-SNAc (n=1, 2, 3, and 4, 1-4) and H_2N-(β-Ala)$_n$-SNAc (n=1, 2, 3, and 4, 5-8)

To a solution of corresponding carboxylic acid synthesized in 6.1 (1.00 eq., 0.500 mmol) in DCM (2 mL) was added 4-dimethylaminopyridine (DMAP, 20.0 mol%, 0.100 mmol, 11.2 mg), 1-ethyl-3-(3-dimethylaminopropyl) carbodiimide hydrochloride (EDCI·HCl, 1.20 eq., 0.600 mmol, 115 mg), and *N*-acetylcysteamine (SNAc, 1.20 eq., 0.600 mmol, 71.5 mg) at 0 °C. The resulting mixture was allowed to warm to room temperature and stirred for 16 h. The reaction was quenched by addition of sat. aq. NH_4Cl, then extracted with DCM. The combined organic layers were dried over Na_2SO_4, filtered, and concentrated under reduced pressure. The residue was purified by flash column chromatography on silica gel (cyclohexane:ethyl acetate 4:1) to give protected SNAc derivatives.[38] Boc-protecting group was removed by dissolving the sample in 50% TFA/DCM solution. After stirring for 4 h, the mixture was concentrated under reduced pressure to give the desired product without any further purification.[39] In case of using Boc-L-Asp-O*t*Bu, the O*t*Bu-protecting group was removed by dissolving it in DCM (2 mL) and adding 2 mL of aq. phosphoric acid (85 wt%). The mixture was stirred at room temperature for 6 h. Then, 5 mL of water was added, and the mixture was stirred for an additional 30 min. The resulting suspension was filtered and the solid was washed with DCM and water to give the desired product without any further purification.[40] Thioesters were obtained in 76% yield (**1**), 63% yield (**2**), 43% yield (**3**), 38% yield (**4**), 84% yield (**5**), 82% yield (**6**), 73% yield (**7**), 43% yield (**8**), respectively.

8.3. Synthesis of H_2N-(β-Ala)$_n$-*S*-CoA (n=1, 2, and 3, 9-11)

Boc-(β-Ala)$_n$-OH (2.00 eq., 0.0800 mmol, n=1: 15.1 mg; n=2: 20.8 mg; n=3: 26.5 mg), CoA (1.00 eq., 40.0 µmol, 30.7 mg), benzotriazol-1-yloxytripyrrolidinophosphonium hexafluorophosphate (PyBOP, 2.00 eq., 80.0 µmol, 41.6 mg) and potassium carbonate (4.00 eq., 0.160 mmol, 22.1 mg) were added to 2 mL of a tetrahydrofuran water solution (1:1). The mixture was stirred for 3 h at room temperature. The mixture was purified with HPLC (a linear gradient of 10% to 60% acetonitrile over 20 min in water with 0.1% TFA) to give Boc-(β-Ala)$_n$-*S*-CoA as a white amorphous powder (n=1: 10.6 mg, 28% yield; n=2: 14.7 mg, 36% yield; n=3: 15.6 mg, 36% yield). Boc-(β-Ala)$_n$-*S*-CoA was then dissolved in 2 mL of a tetrahydrofuran water solution (1:1), and TFA (1 mL) was added dropwise at 0 °C. The reaction was allowed to warm to room temperature and stirred for 2 h. The mixture was purified with HPLC (a linear gradient of 5% to 20% acetonitrile over 20 min in water with 0.1% TFA) to give H_2N-(β-Ala)$_n$-*S*-CoA as a white amorphous powder (n=1: 8.00 mg, 84% yield; n=2: 3.60 mg, 27% yield; n=3: 5.50 mg, 39% yield). Spectral data are in accordance with literature values.[41] CoA substrates were obtained in 24% yield (**9**), 10% yield (**10**), and 14% yield (**11**), respectively.

8.4. Synthesis of H_2N-L-Lys-SNAc (12)

H_2N-L-Lys-SNAc was synthesized according to the same procedure described in section 6.2 with Boc-L-Lys(Boc)-OH (1.00 eq., 0.500 mmol, 173 mg), 4-dimethylaminopyridine (DMAP, 20.0 mol%, 0.100 mmol, 11.2 mg), 1-ethyl-3-(3-dimethylaminopropyl) carbodiimide hydrochloride (EDC·HCl, 1.20 eq., 0.600 mmol, 115 mg), and *N*-acetylcysteamine (SNAc, 1.20 eq., 0.600 mmol, 71.5 mg). H_2N-L-Lys-SNAc was obtained in 84% yield.

8.5. Synthesis of H_2N-L-Lys-*S*-CoA (13)

Boc-L-Lys(Boc)-OH (3.00 eq., 0.150 mmol, 52.0 mg), CoA (1.00 eq., 50.0 µmol, 45.1 mg), benzotriazol-1-yloxytripyrrolidinophosphonium hexafluorophosphate (2.00 eq., 0.100 mmol, 52.0 mg) and potassium carbonate (4.00 eq., 0.200 mmol, 27.6 mg) were added to 2 mL of a tetrahydrofuran water solution (1:1). The mixture was stirred for 3 h at room temperature. The mixture was purified with HPLC (a linear gradient of 10% to 60% acetonitrile over 20 min in water with 0.1% TFA) to give Boc- L-Lys(Boc)-*S*-CoA as a white amorphous powder (21.9 mg, 40% yield). Boc-L-Lys(Boc)-*S*-CoA was then dissolved in 2 mL of a tetrahydrofuran water solution (1:1), and TFA (1 mL) was added dropwise at 0 °C. The reaction was allowed to warm to room temperature and stirred for 2 h. The mixture was purified with HPLC (a linear gradient of 5% to 20% acetonitrile over 20 min in water with 0.1% TFA) to give H_2N-L-Lys-*S*-CoA as a white amorphous powder (4.89 mg, 27% yield).

Supplementary Table 1. List of species belonging to the *Burkholderia cepacia* complex (BCC).

BCC species	Reference	BCC species	Reference
B. cepacia	(Vandamme et al. 1997)	*B. metallica*	(Depoorter et al. 2016)
B. multivorans	(Vandamme et al. 1997)	*B. arboris*	(Depoorter et al. 2016)
B. cenocepacia	(Vandamme et al. 1997; Vandamme et al. 2003)	*B. contaminans*	(Depoorter et al. 2016)
B. stabilis	(Vandamme et al. 1997; Vandamme 2000)	*B. lata*	(Depoorter et al. 2016)
B. vietnamiensis	(Vandamme et al. 1997; Monique Gillis 1995)	*B. latens*	(Depoorter et al. 2016)
B. dolosa	(Coenye, LiPuma, et al. 2001; Vermis et al. 2004)	*B. pseudomultivorans*	(Depoorter et al. 2016)
B. ambifaria	(Coenye, Mahenthiralingam, et al. 2001)	*B. diffusa*	(Depoorter et al. 2016)
B. anthina	(Vandamme et al. 2002)	*B. paludis*	(Ong et al. 2016)
B. pyrrocinia	(Vandamme et al. 2002)	*B. catarinensis*	(Bach et al. 2017)
B. ubonensis	(Yabuuchi et al. 2000)	*B. alpina*	(Weber and King 2017)
B. stagnalis	(De Smet et al. 2015)	*B. puraquae*	(Martina et al. 2018)
B. territorii	(De Smet et al. 2015)	*B. aenigmatica*	(Depoorter et al. 2020)
B. seminalis	(Depoorter et al. 2016)	*B. orbicola*	(LM Morales et el. 2022)

Supplementary Table 2. Putative functions of proteins from the *afc* BGC based on BlastP search and comparison to the *bol* BGC.

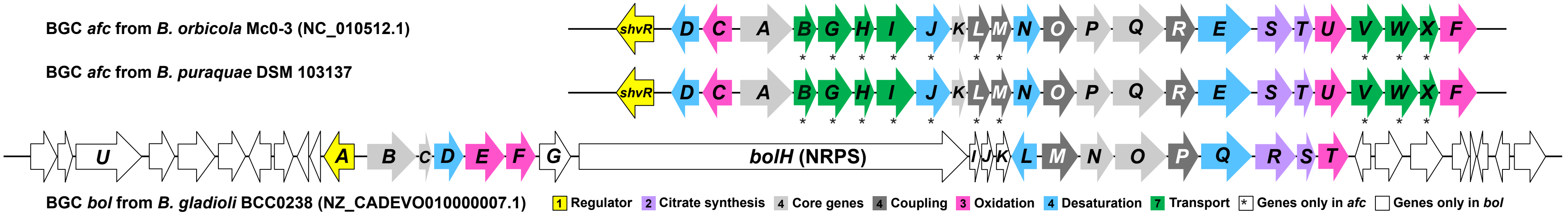

Nr.	Gene[a]	Putatively encoded protein (locus tag, CDS)	Length (aa)	Protein hit[b] (gene, locus tag, CDS)	Length (aa)	Identity/Similarity (%)	Comments
1	*shvR*	LysR substrate-binding domain-containing protein (BCENMC03_RS32450, WP_226247193.1)	321	LysR substrate-binding domain-containing protein (*bolA*, H9D26_RS17460, WP_105850350.1)	329	58/70	Transcriptional regulator, LysR family
2	*afcD*	Acyl-ACP desaturase/dehydrogenase (BCENMC03_RS32455, WP_041492293.1)	286	Ferritin-like domain-containing protein (*bolL*, H9D26_RS17515, WP_126241845.1)	280	91/95	$\Delta^{6/9}$ fatty acid desaturase
3	*afcC*	Fatty acid desaturase family protein (BCENMC03_RS32460, WP_011694954.1)	315	Fatty acid desaturase family protein (*bolF*, H9D26_RS17485, WP_036055811.1)	343	23/40	
4	*afcA*	Fatty acyl-AMP ligase/FAAL (BCENMC03_RS32465, WP_226247195.1)	587	AMP-binding protein (*bolB*, H9D26_RS17465, WP_241238501.1)	577	52/66	Fatty acid activation
5	*afcB*	ABC transporter (membrane-associated ATPase) (BCENMC03_RS32470, WP_012337003.1)	236	-	-	-	Lipid A exporter, phosphatidylcholine exporter, lipoprotein transporter, peptide transport, multidrug efflux
6	*afcG*	ABC transporter (transmembrane component) (BCENMC03_RS32475, WP_006481180.1)	405	-	-	-	Lipoprotein exporter, permease, macrolide exporter
7	*afcH*	Outer membrane lipoprotein-sorting protein (BCENMC03_RS32480, WP_012337004.1)	250	-	-	-	Lipoprotein carrier/trafficking, translocation of complex lipids
8	*afcI*	Outer membrane carboxylate channel (BCENMC03_RS32485, WP_226247197.1)	420	-	-	-	Uptake of nutrients and negatively charged acids
9	*afcJ*	Acyl-CoA/ACP dehydrogenase/oxidase (FAD as cofactor) (BCENMC03_RS32490, WP_012337006.1)	361	-	-	-	Glutaryl-CoA dehydrogenases, acyl-beta-oxidation, double bond into acyl chains (tetramer)
10	*afcK*	Acyl carrier protein/Peptidyl-carrier protein (BCENMC03_RS32495, WP_006481176.1)	84	Acyl carrier protein (*bolC*, H9D26_RS17470, WP_013690725.1)	82	67/82	ACP/PCP
11	*afcL*	3-Oxoacyl-ACP synthase III/type-III PKS (BCENMC03_RS32500, WP_012337007.1)	291	-	-	-	C-C coupling (Claisen), condensing acyl-CoA with acyl-ACP for the initiation of fatty acid//polyketide synthesis
12	*afcM*	UDP-Glycosyltransferase (BCENMC03_RS32505, WP_012337008.1)	209	-	-	-	

13	*afcN*	Ferritin-like domain-containing protein/Acyl-ACP desaturase (BCENMC03_RS32510, WP_012337009.1)	327	Ferritin-like domain-containing protein (*bolD*, H9D26_RS17475, WP_105850352.1)	329	72/84	Ribonucleotide reductase; needs electrons from NADPH-ferredoxin reductase; aldehyde decarbonylase; non-heme di-iron protein; p-aminobenzoate N-Oxygenase AurF
14	*afcO*	3-Oxoacyl-ACP synthase III/type-III PKS (BCENMC03_RS32515, WP_012337010.1)	327	3-Oxoacyl-ACP synthetase (*bolM*, H9D26_RS17520, WP_126241404.1)	376	62/69	C-C coupling (Claisen), homologous to starter PKS that accept starter acyl-ACP (over CoA substrates)
15	*afcP*	PLP-dependent decarboxylase (BCENMC03_RS32520, WP_012337011.1)	391	PLP-dependent decarboxylase (*bolN*, H9D26_RS17525, WP_126241405.1)	398	73/80	
16	*afcQ*	Acyl-CoA ligase (AMP-forming) (BCENMC03_RS32525, WP_012337012.1)	527	Acyl-CoA ligase (AMP-forming) (*bolO*, H9D26_RS17530, WP_105852536.1)	527	75/84	
17	*afcR*	3-Oxoacyl-ACP synthase III/type-III PKS (BCENMC03_RS32530, WP_012337013.1)	364	Hypothetical protein (*bolP*, H9D26_RS17535, WP_241238502.1)	371	73/81	C-C coupling (Claisen), homologous to starter PKS that accept starter acyl-ACP (over CoA substrates)
18	*afcE*	Acyl-CoA dehydrogenase (FAD as cofactor) (BCENMC03_RS32535, WP_041492342.1)	616	Acyl-CoA dehydrogenase (*bolQ*, H9D26_RS17540, WP_126241406.1)	615	81/89	Double bond in dehydro-β-Ala (L-prolyl-PCP dehydrogenase, homodimer)
19	*afcS*	Citrate synthase/2-Methylcitrate synthase (BCENMC03_RS32540, WP_012337015.1)	414	Citrate synthase (*bolR*, H9D26_RS17545, WP_105857830.1)	453	73/80	Citrate synthesis
20	*afcT*	SAM-dependent O-methyltransferase (BCENMC03_RS32545, WP_011549202.1)	220	Class I SAM-dependent methyltransferase (*bolS*, H9D26_RS17550, WP_013690740.1)	220	85/91	Methylation of citrate
21	*afcU*	TauD/TfdA family dioxygenase (BCENMC03_RS32550, WP_012337016.1)	357	TauD/TfdA family dioxygenase (*bolT*, H9D26_RS17555, WP_036055807.1)	357	78/84	Fe(II)- and α-ketoglutarate-dependent oxygenase, L-arginine β-hydroxylase/desaturase VioC, stereoinversion of C5 in carbapenem via radical (tetramer)
22	*afcV*	ABC transporter (membrane-associated ATPase) (BCENMC03_RS32555, WP_041492343.1)	337	-	-	-	Lipid translocator across membrane
23	*afcW*	ABC transporter (transmembrane component) (BCENMC03_RS32560, WP_012337018.1)	359	-	-	-	Lipid trafficking
24	*afcX*	Permease (BCENMC03_RS32565, WP_012337019.1)	180	-	-	-	Transporter/Channel
25	*afcF*	FAD-binding oxidoreductase (BCENMC03_RS32570, WP_012337020.1)	472	FAD-binding oxidoreductase (*bolE*, H9D26_RS17480, WP_105850353.1)	480	55/60	D-2-hydroxyglutarate dehydrogenase, LDH, Cytokinin oxidase, hydroxylase/monooxygenase

[a] Annotation of *afc* gene cluster from *B. orbicola* Mc0-3 (NC_010512.1). [b] Annotation of *bol* gene cluster from *B. gladioli* BCC0238 (NZ_CADEVO010000007.1).[42,43]

Supplementary Table 3. Compilation of the occurrence of *afc* BGC in BCC based on BlastN screening for key genes *afcA*, *afcQ* and the entire *afc* BGC[a].

Nr.	Strain	Accession (location)	*afcA*	*afcQ*	*afc* BGC
			Cover/Identity	Cover/Identity	Cover/Identity
1	*B. ambifaria* AMMD	CP009800.1 (1248023-1275388)	98/88.73	100/90.21	98/90.40
2	*B. ambifaria* AU0212	CP111125.1 (689358-716723)	98/88.73	100/90.21	98/90.40
3	*B. ambifaria* B21-006	CP086299.1 (824455-851802)	98/89.76	100/90.09	98/90.53
4	*B. ambifaria* B21-008	CP086293.1 (796857-824210)	98/89.06	100/90.40	98/90.49
5	*B. ambifaria* CEP0996	CP113270.1 (N.A.)[b]	98/89.47	100/90.03	98/90.51
6	*B. ambifaria* FDAARGOS_1027	CP066040.1 (679128-706493)	98/88.73	100/90.21	98/90.40
7	*B. ambifaria* HSJ1	CP113276.1 (N.A.)	98/88.73	100/90.21	98/90.40
8	*B. ambifaria* HSJ1v	CP113273.1 (N.A.)	98/88.73	100/90.21	98/90.40
9	*B. ambifaria* Q53	CP092845.1 (136911-164260)	98/89.25	100/90.09	98/90.56
10	*B. arboris* MEC_B345	CP101526.1 (410024-437293)	100/92.69	99/93.00	100/92.54
11	*B. cenocepacia* GIMC4560:Bcn122	CP020601.1 (215371-242609)	100/95.58	100/96.53	96/96.03
12	*B. cenocepacia* H111	HG938372.1 (231018-258259)	100/95.98	100/96.78	100/95.45
13	*B. cenocepacia* HI2424	CP000460.1 (145158-172487)	100/98.47	100/97.68	100/97.92
14	*B. cenocepacia* J2315	AM747722.1 (217322-244566)	100/96.03	100/96.84	100/95.54
15	*B. cenocepacia* 2008Ycf657	CP090762.1 (759010-786268)	100/95.86	100/96.78	100/95.57
16	*B. cenocepacia* 2009Ycf95III	CP090759.1 (758949-786207)	100/95.86	100/96.78	100/95.57
17	*B. cenocepacia* 2019Y71443953II	CP090675.1 (759007-786265)	100/95.86	100/96.78	100/95.57
18	*B. cenocepacia* 842	CP015035.1 (859122-886377)	100/96.20	100/96.59	100/95.53
19	*B. cenocepacia* 895	CP015037.1 (19448-46703)	100/96.09	100/96.21	100/95.55
20	*B. cenocepacia* C6433	CP098498.1 (32206-59137)	100/95.69	100/96.72	96/96.33
21	*B. cenocepacia* CMCC(B)23006	CP091014.1 (626891-654217)	100/98.30	100/98.05	100/97.82
22	*B. cenocepacia* CR318	CP017240.1 (896053-923382)	100/98.47	100/97.68	100/97.92
23	*B. cenocepacia* FDAARGOS_720	CP050980.1 (3264383-3291651)	100/98.47	100/97.68	99/97.84
24	*B. cenocepacia* FDAARGOS_734	CP054819.1 (221786-249030)	100/96.03	100/96.84	100/95.54
25	*B. cenocepacia* IST439	LR798195.1 (N.A.)	100/95.63	100/96.59	100/95.51
26	*B. cenocepacia* K56-2	ALJA02000017.1 (460263-487509)	100/98.64	100/98.86	100/95.53
27	*B. cenocepacia* MSMB384WGS	CP013451.1 (652316-679574)	100/95.46	100/96.97	100/95.49
28	*B. cenocepacia* NML110041	CP102477.1 (609900-637145)	100/96.03	100/96.84	100/95.54
29	*B. cenocepacia* PC184 Mulks	CP021068.1 (632142-659461)	100/97.96	100/97.61	100/97.83
30	*B. cenocepacia* PS27	CP060041.1 (398867-426190)	100/97.96	100/97.36	100/97.79
31	*B. cenocepacia* R-12632	FR989821.1 (298053-325365)	100/94.61	100/94.47	99/95.42
32	*B. cenocepacia* ST32	CP011919.1 (699550-726808)	100/95.75	100/96.28	100/95.54
33	*B. cenocepacia* toggle2	CP073670.1 (18817-46074)	100/95.92	100/96.59	100/95.53
34	*B. cenocepacia* toggle3	CP073703.1 (232575-259832)	100/95.92	100/96.59	100/95.54
35	*B. cenocepacia* toggle4	CP073675.1 (727951-755209)	100/95.98	100/96.53	100/95.62
36	*B. cenocepacia* VC12308	CP019673.1 (728940-756195)	100/96.03	100/96.53	100/95.52
37	*B. cenocepacia* VC1254	CP019675.1 (917457-944714)	100/95.75	100/96.28	100/95.55

38	*B. cenocepacia* VC12802	CP019669.1 (850163-877464)	100/97.62	100/97.62	100/97.71
39	*B. cenocepacia* VC2307	CP019667.1 (733654-760912)	100/95.86	100/96.28	100/95.51
40	*B. cenocepacia* VC7848	CP019668.1 (4344704-4371987)	100/98.02	100/97.16	100/97.87
41	*B. cenocepacia* YG-3	CP034546.1 (1317027-1344540)	100/94.10	100/94.20	99/95.12
42	*B. cepacia* ATCC 25416	CP034555.1 (223553-250933)	100/91.78	100/92.75	100/91.87
43	*B. cepacia* MINF_4A-sc-2280433	LR890525.1 (N.A.)	100/95.46	100/96.78	100/95.60
44	*B. cepacia* JBK9	CP013732.1 (405328-432611)	100/92.74	99/92.93	99/93.02
45	*B. cepacia* 2011Ycf427V	CP090739.1 (978720-1006128)	100/91.67	100/91.09	100/91.66
46	*B. cepacia* 2011Ycf793I	CP090731.1 (353288-380696)	100/91.67	100/91.09	100/91.66
47	*B. cepacia* 2017Y70952565VI	CP090699.1 (4617731-4645139)	100/91.67	100/91.09	100/91.66
48	*B. cepacia* 39628	CP032010.1 (403059-430469)	100/91.50	100/91.67	100/91.72
49	*B. cepacia* BC16	CP045237.1 (1027512-1054909)	100/91.55	100/91.83	100/91.65
50	*B. cepacia* BRDJ	CP095496.1 (N.A.)	100/91.84	100/92.63	100/91.84
51	*B. cepacia* CMCC(B)23005	CP090610.1 (104437-131834)	100/91.84	100/91.21	100/91.74
52	*B. cepacia* DDS 7H-2	CP007785.1 (332750-360005)	100/95.69	100/97.16	100/95.57
53	*B. cepacia* FDAARGOS_345	CP022082.2 (927371-954751)	100/91.78	100/92.75	100/91.87
54	*B. cepacia* FDAARGOS_388	CP023520.1 (481179-508559)	100/91.78	100/92.75	100/91.87
55	*B. cepacia* N3009-2YT	CP102331.1 (N.A.)	100/91.89	100/91.83	100/91.65
56	*B. contaminans* CH-1	AP018359.1 (429548-456937)	100/91.84	100/92.18	100/91.47
57	*B. contaminans* B17-01563-1	CP092853.1 (420703-448092)	100/91.84	100/92.18	100/91.47
58	*B. contaminans* DM32	CP102484.1 (204642-232020)	100/91.17	100/91.41	100/91.34
59	*B. contaminans* FL-1-2-30-S1-D0	CP013392.1 (457710-485099)	100/91.73	100/91.36	100/91.45
60	*B. contaminans* MS14	CP009745.1 (926750-954145)	100/91.76	100/91.48	100/91.42
61	*B. contaminans* NML151013	CP102464.1 (290767-318162)	100/91.44	100/91.93	99/91.86
62	*B. contaminans* NML151067	CP102469.1 (357188-384583)	100/91.44	100/91.93	99/91.86
63	*B. contaminans* SK875	CP028809.1 (847464-874853)	100/91.84	100/92.18	100/91.47
64	*B. contaminans* toggle1	CP073664.1 (1069619-1097005)	100/91.84	100/92.18	100/91.46
65	*B. contaminans* XL73	CP046607.1 (1227616-1255005)	100/91.84	100/92.18	100/91.47
66	*B. contaminans* ZCC	CP042166.1 (1227532-1254921)	100/91.84	100/92.18	100/91.47
67	*B. lata* 383	CP000150.1 (1019324-1046703)	100/91.16	100/91.48	100/91.28
68	*B. lata* FL-7-5-30-S1-D0	CP013405.1 (403707-431102)	100/91.44	100/91.73	100/91.91
69	*B. metallica* FL-6-5-30-S1-D7	CP013402.1 (716561-743920)	100/92.19	100/91.24	98/92.28
70	*B. pyrrocinia* DSM 10685	CP011505.1 (813232-840517)	100/91.91	100/91.48	98/92.51

[a] *afcA* (BCENMC03_RS32465), *afcQ* (BCENMC03_RS32525) and the complete *afc* gene cluster (NC_010512.1, location 545185-572489) from *B. orbicola* Mc0-3 (NC_010512.1) as reference. [b] Not annotated.

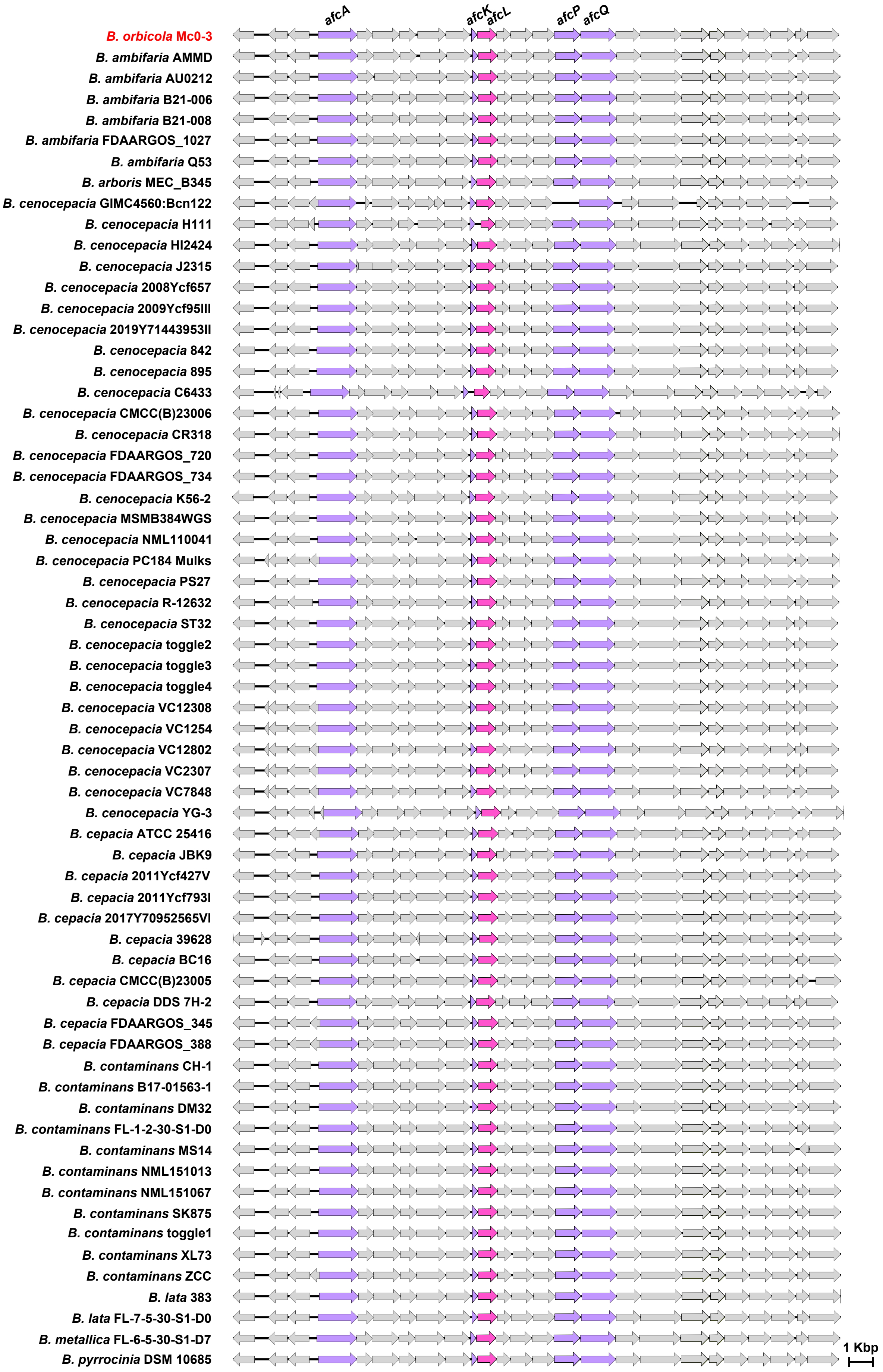

Supplementary Figure 1. The *afc* BGC is conserved in eight BCC species (*B. ambifaria*, *B. arboris*, *B. cenocepacia*, *B. cepacia*, *B. contaminans*, *B. lata*, *B. metallica*, and *B. pyrrocinia*). The gene clusters listed were extracted from BCC strains using the annotation in Supplementary Table 3.

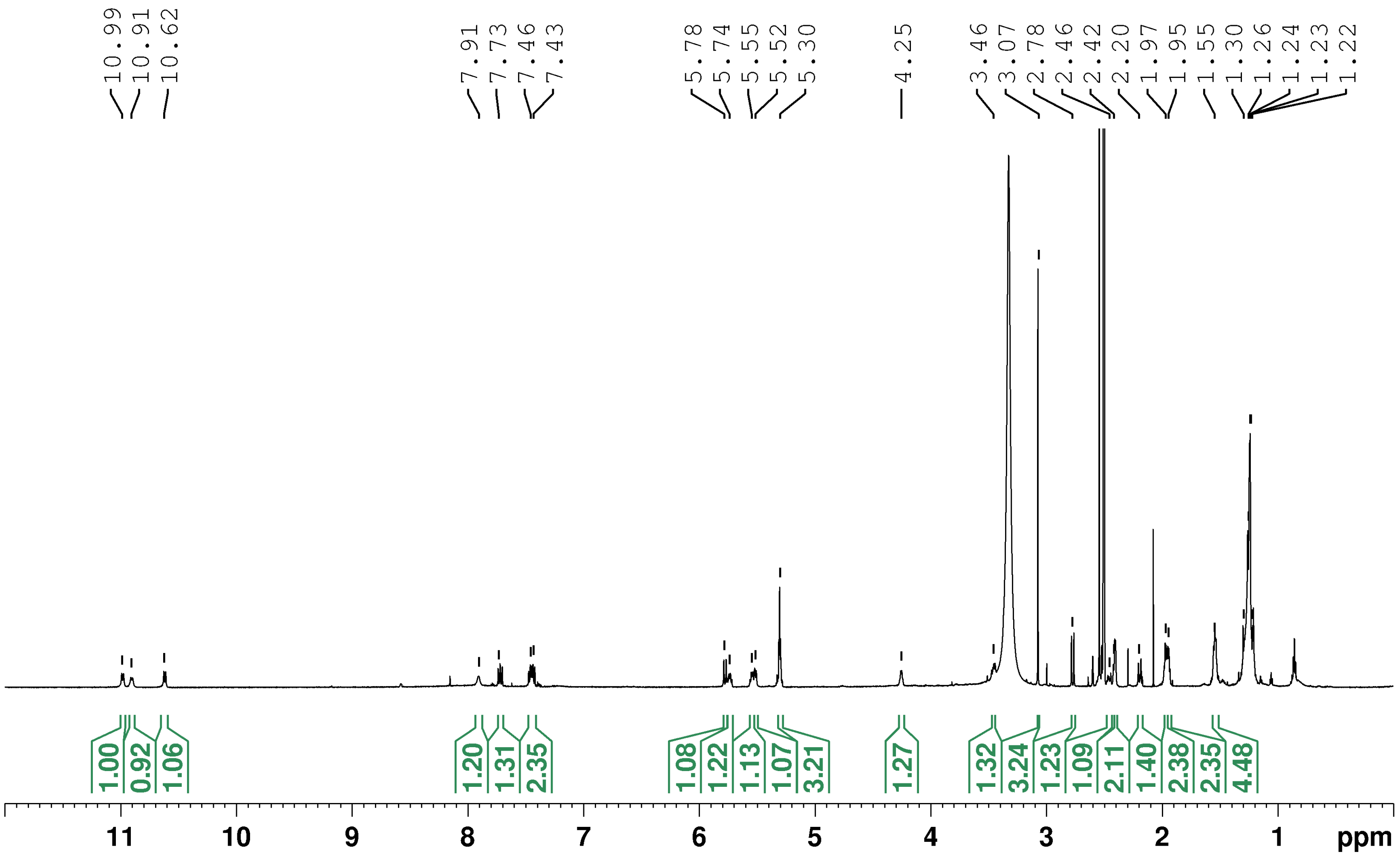

Supplementary Figure 2. ^{1}H-NMR spectrum of AFC-BC11 (DMSO-d_6, 298 K).

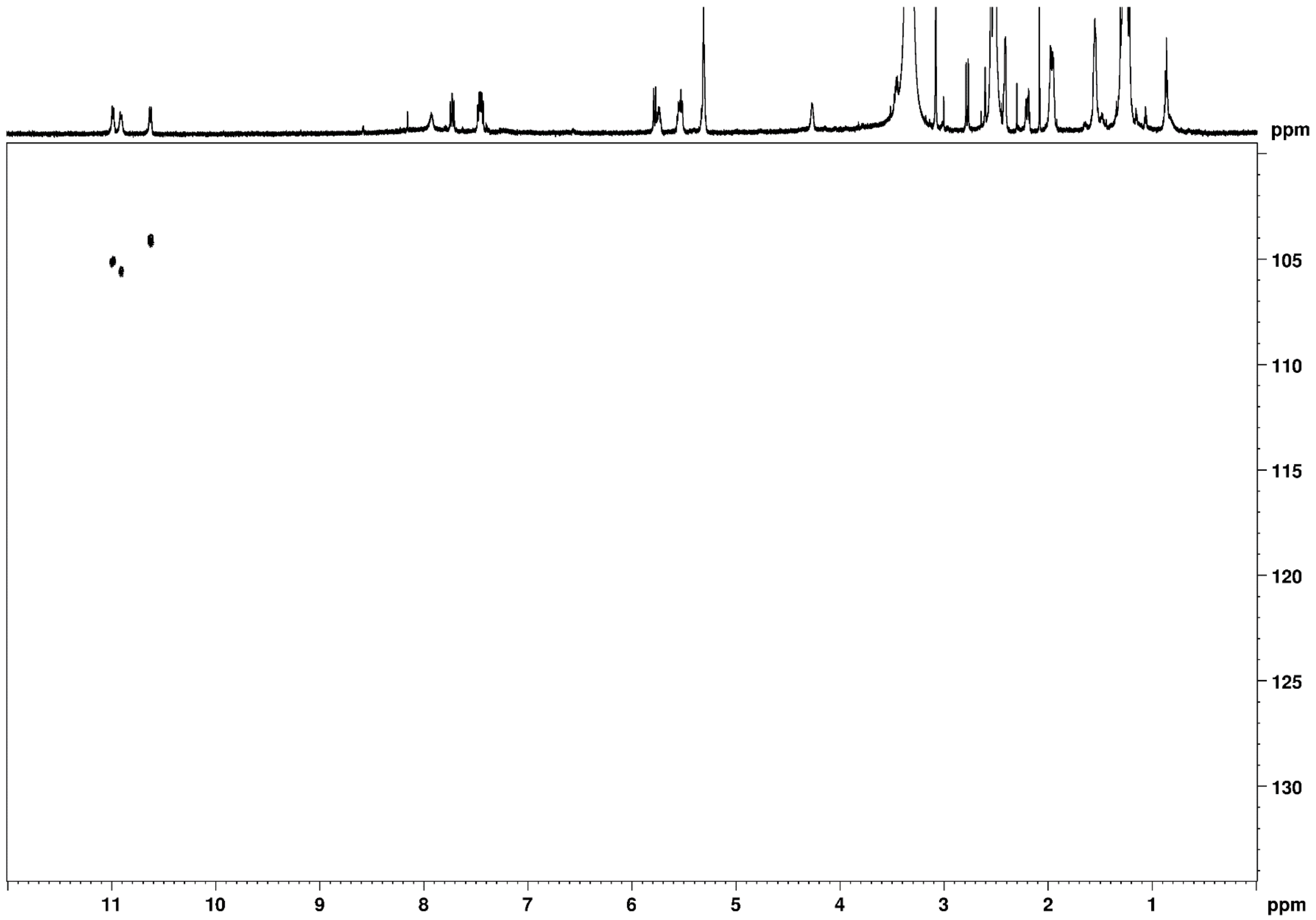

Supplementary Figure 3. ^{1}H-^{15}N SOFAST-HMQC spectrum of AFC-BC11 (NS: 512, SW:35 and O1P:117 for F1) applying ^{1}H excitation at 10.8 ppm with a bandwidth of 3 ppm (DMSO-d_6, 298 K).

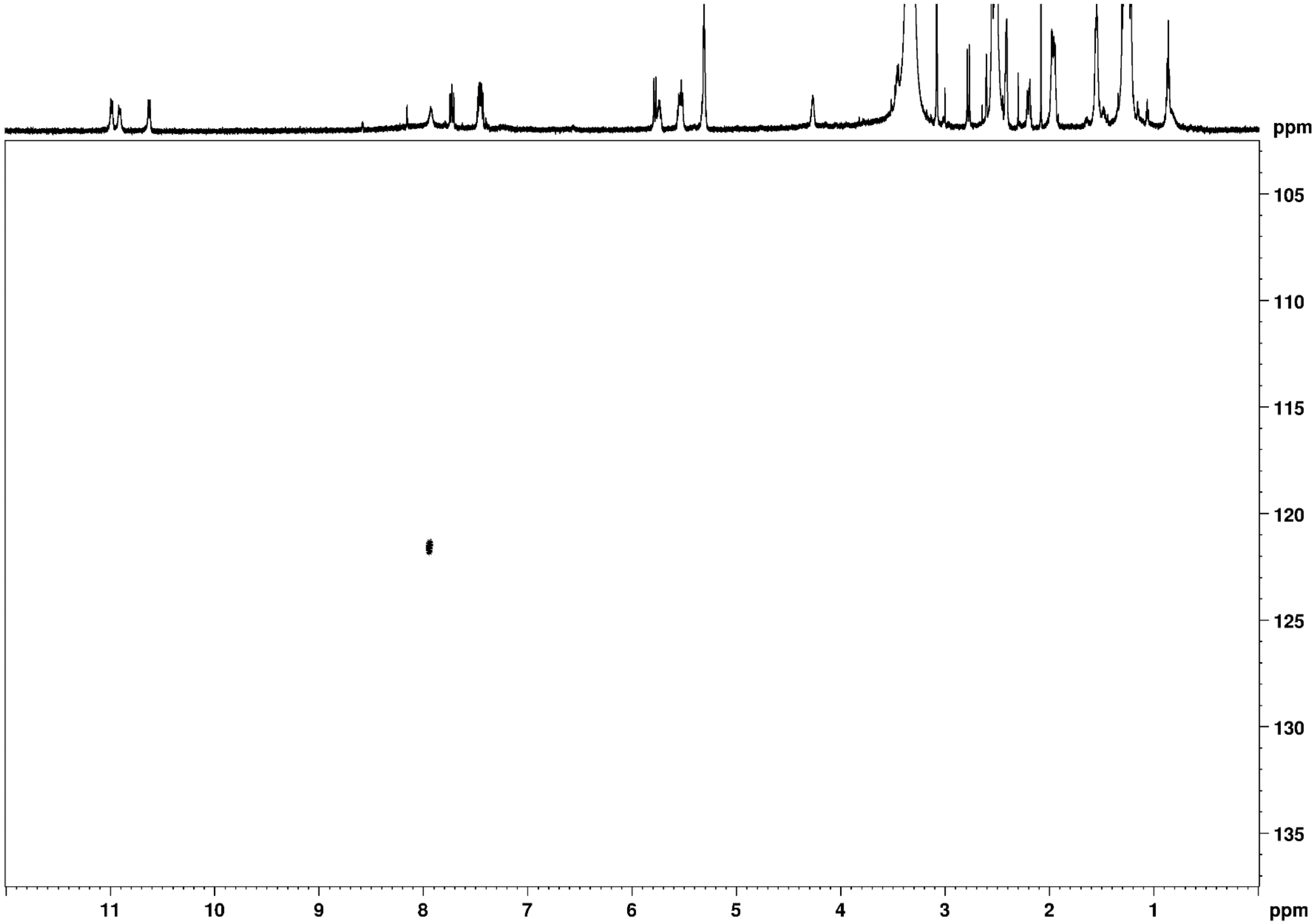

Supplementary Figure 4. ^{1}H-^{15}N SOFAST-HMQC spectrum of AFC-BC11 (NS: 1024, SW:35 and O1P:120 for F1) applying ^{1}H excitation at 7.95 ppm with a bandwidth of 2 ppm (DMSO-d_6, 298 K).

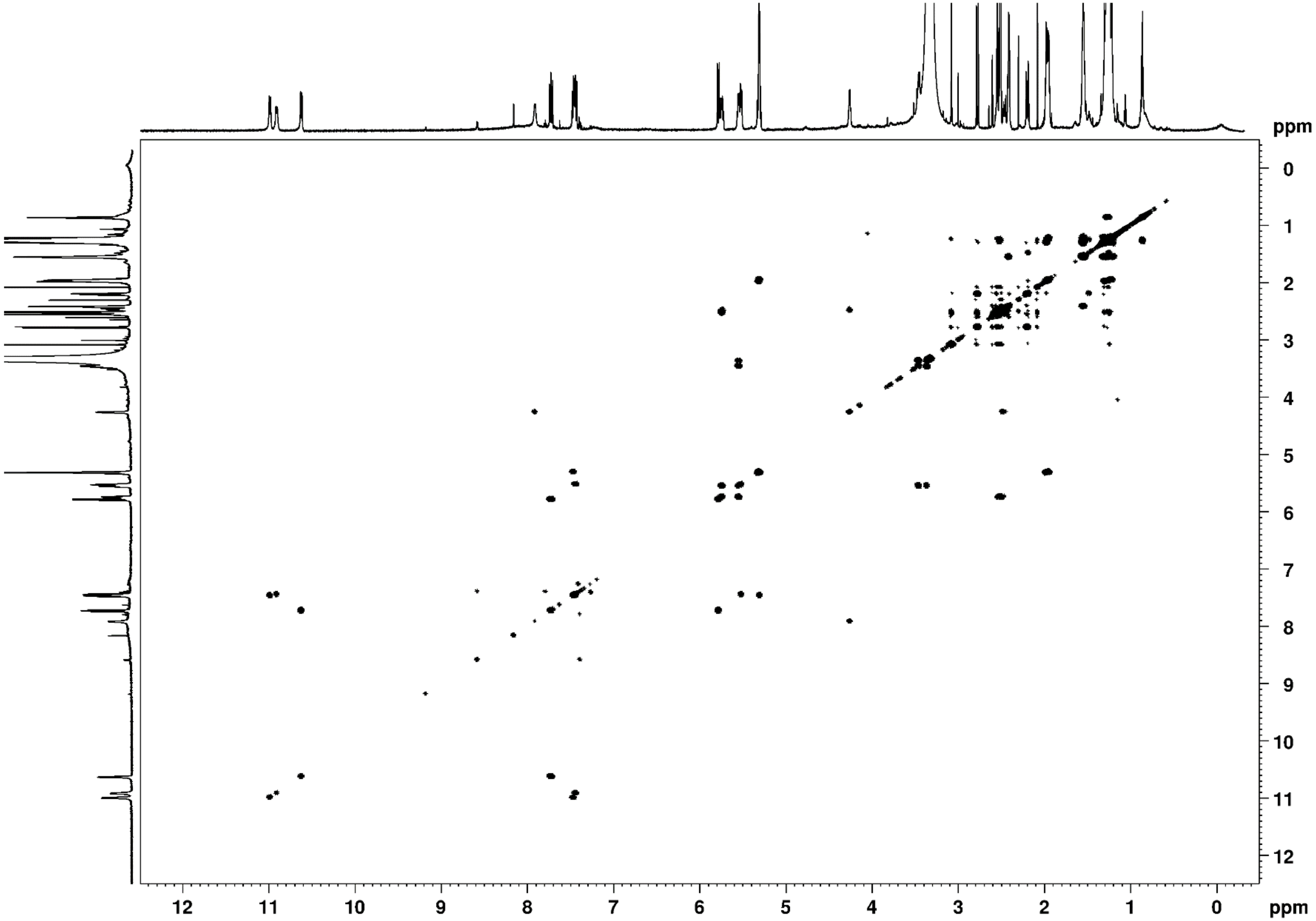

Supplementary Figure 5. ^{1}H-^{1}H COSY spectrum of AFC-BC11 (DMSO-d_6, 298 K).

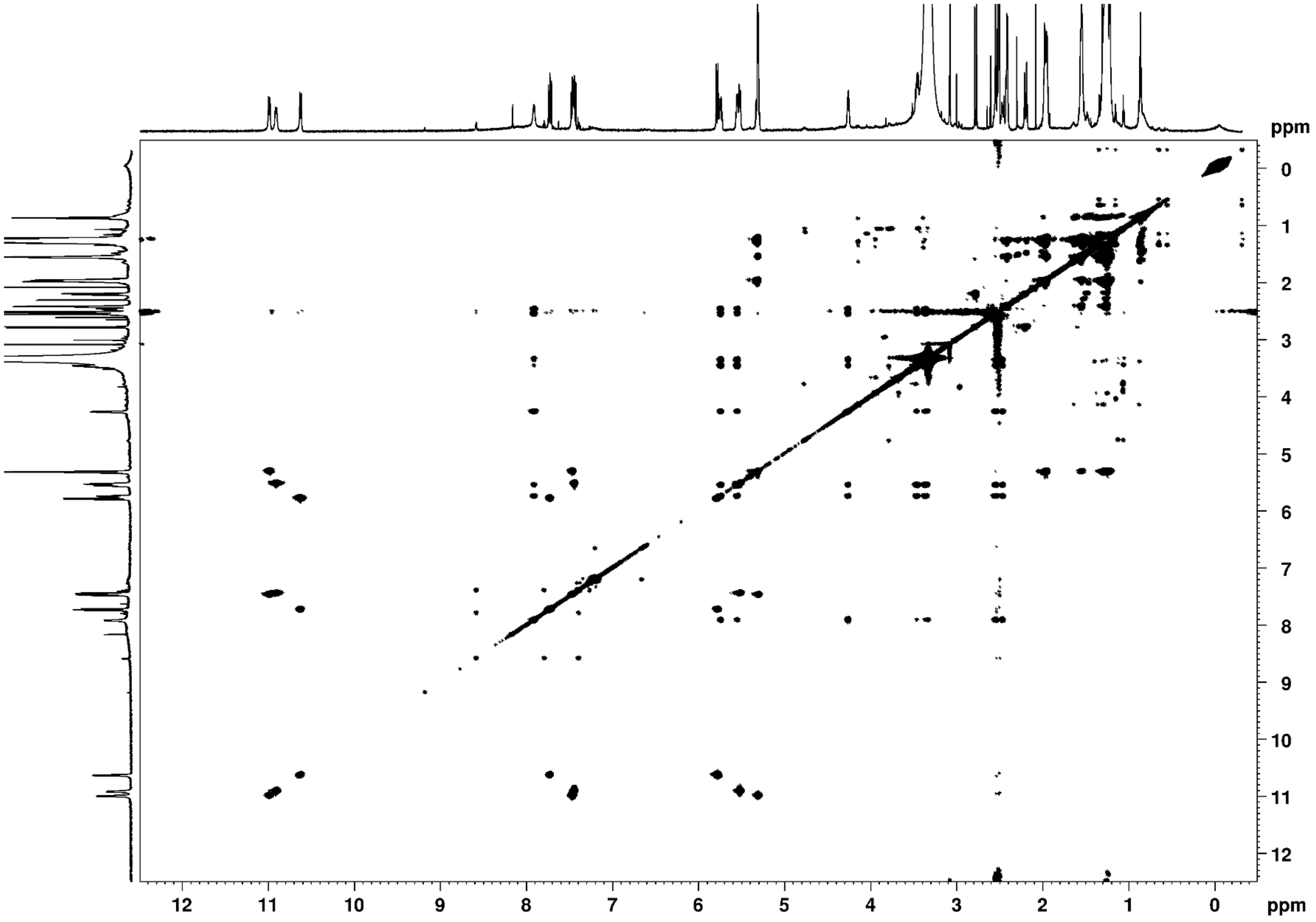

Supplementary Figure 6. ^1H-^1H TOCSY spectrum of AFC-BC11 (DMSO-d_6, 298 K).

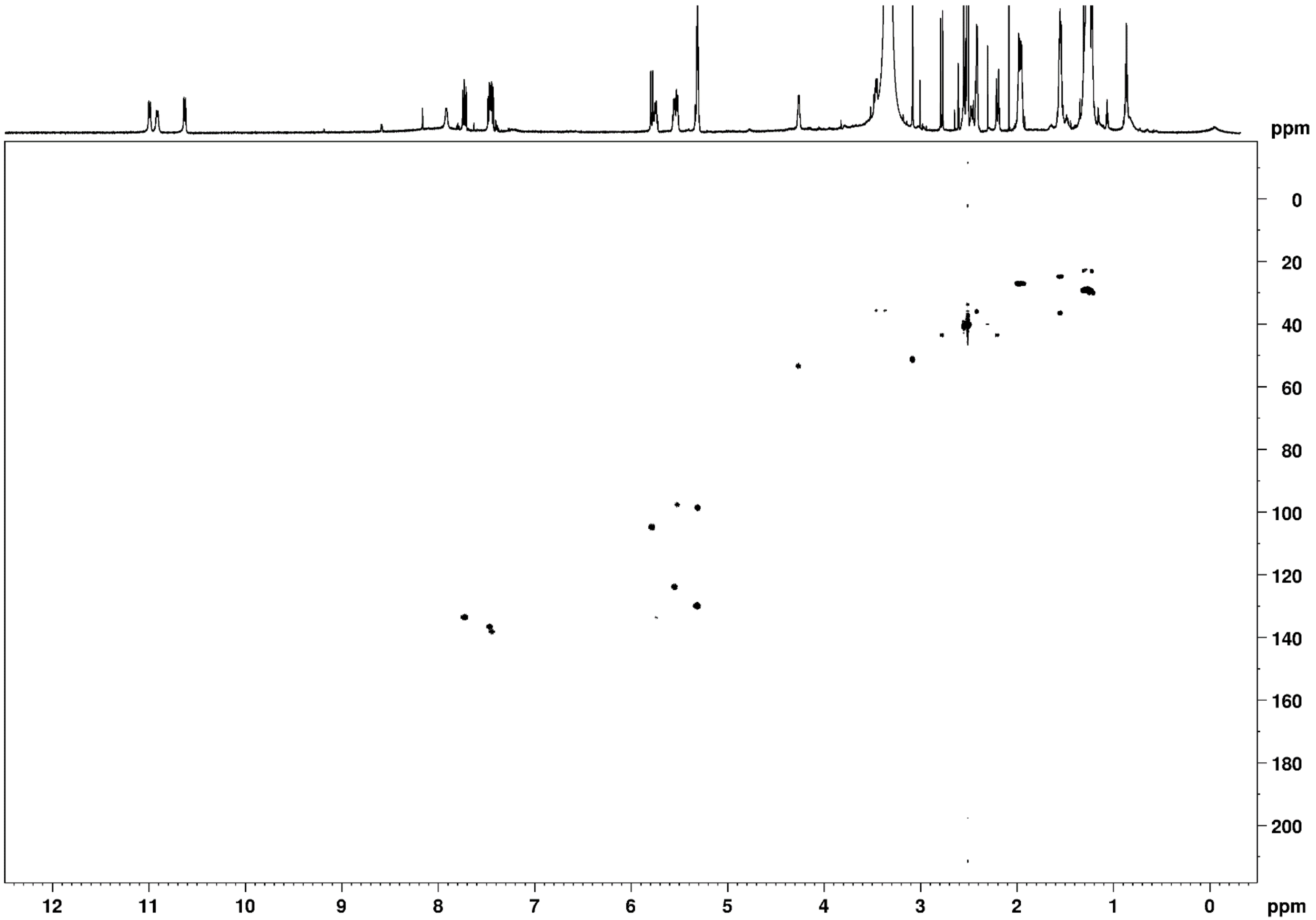

Supplementary Figure 7. ^{1}H-^{13}C HSQC spectrum of AFC-BC11 (DMSO-d_6, 298 K).

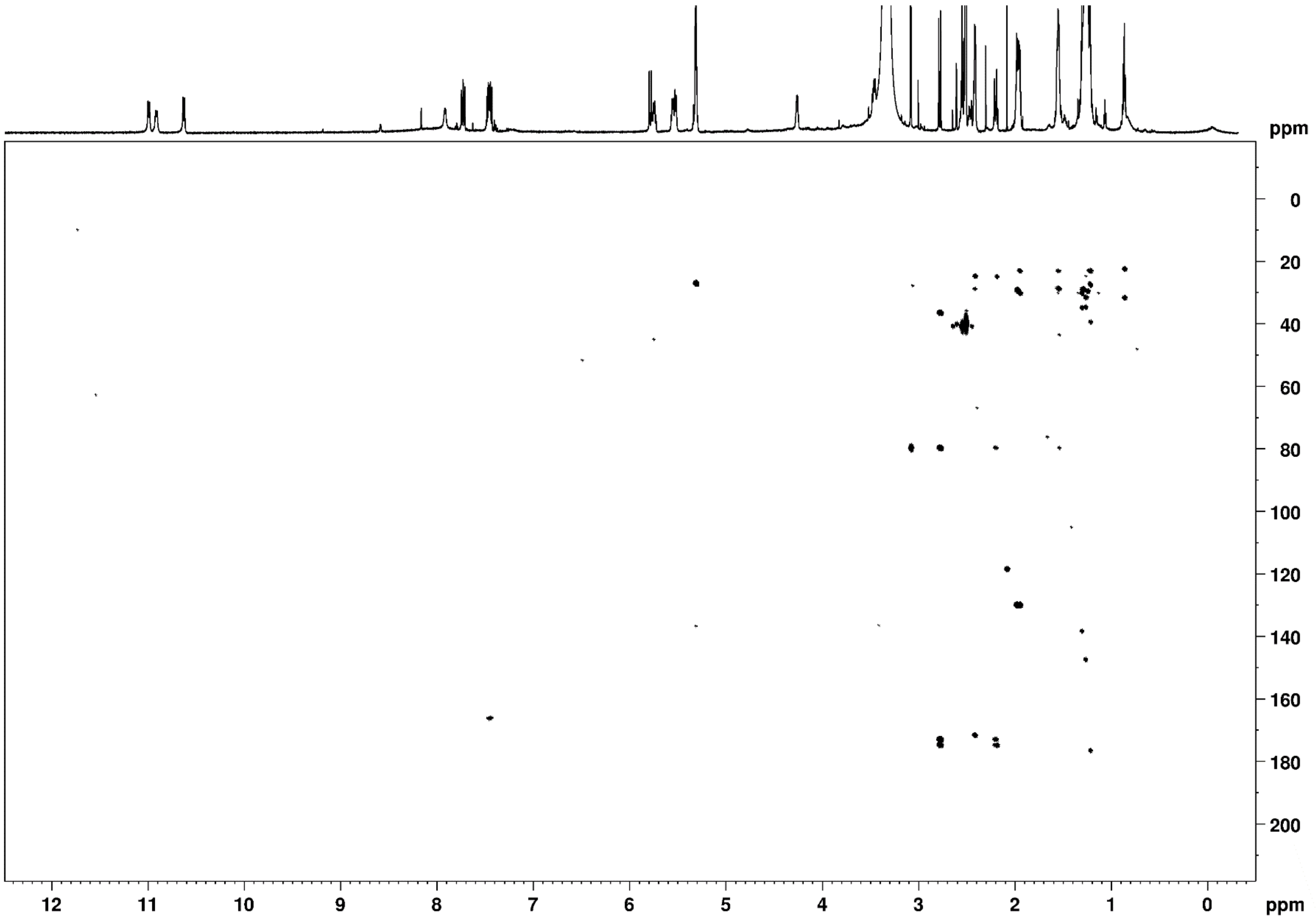

Supplementary Figure 8. ^{1}H-^{13}C HMBC spectrum of AFC-BC11 (SW:236 and O1P:100 for F1) (DMSO-d_6, 298 K).

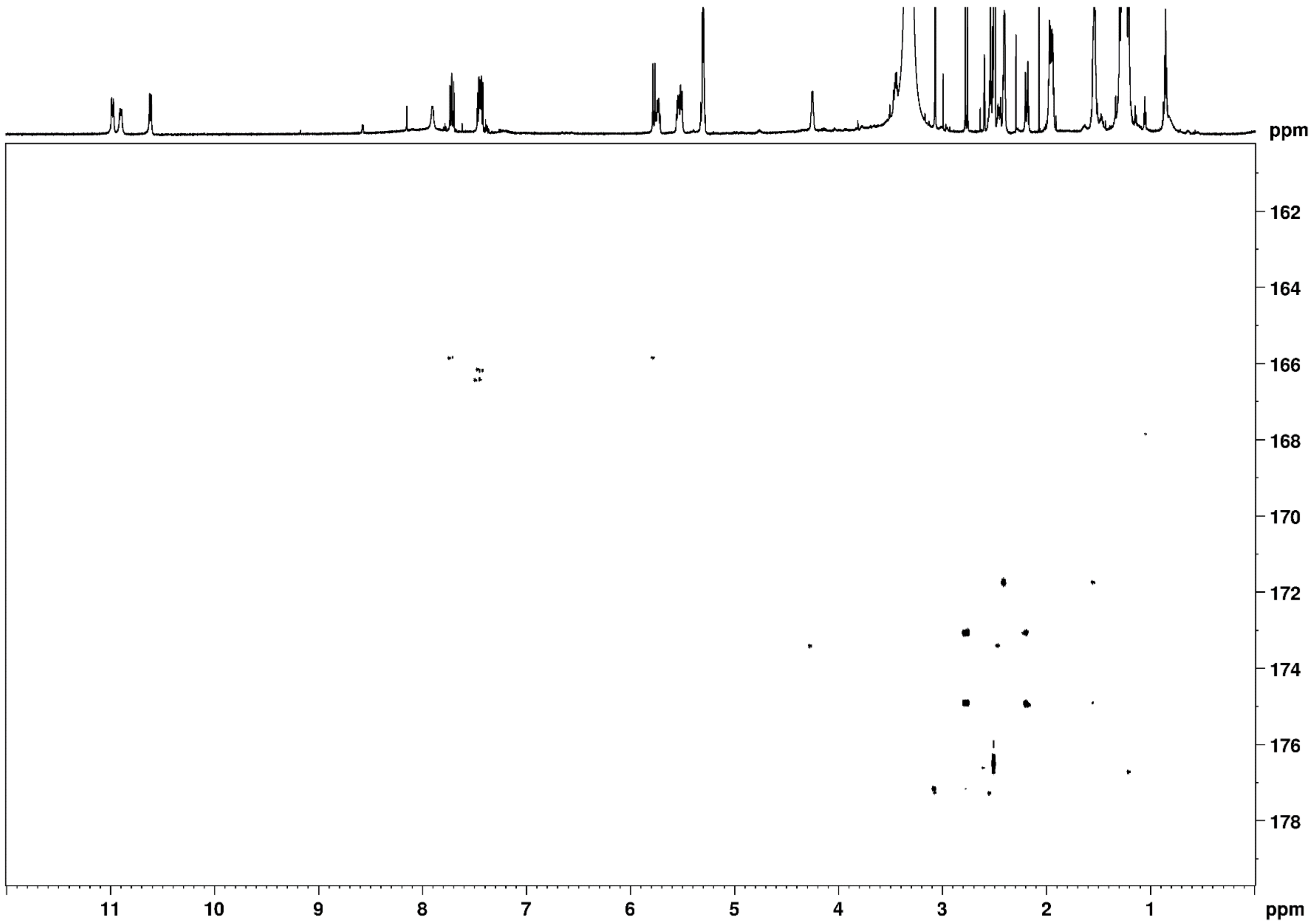

Supplementary Figure 9. ^{1}H-^{13}C HMBC spectrum of AFC-BC11 (SW:19 and O1P:170 for F1) (DMSO-d_6, 298 K).

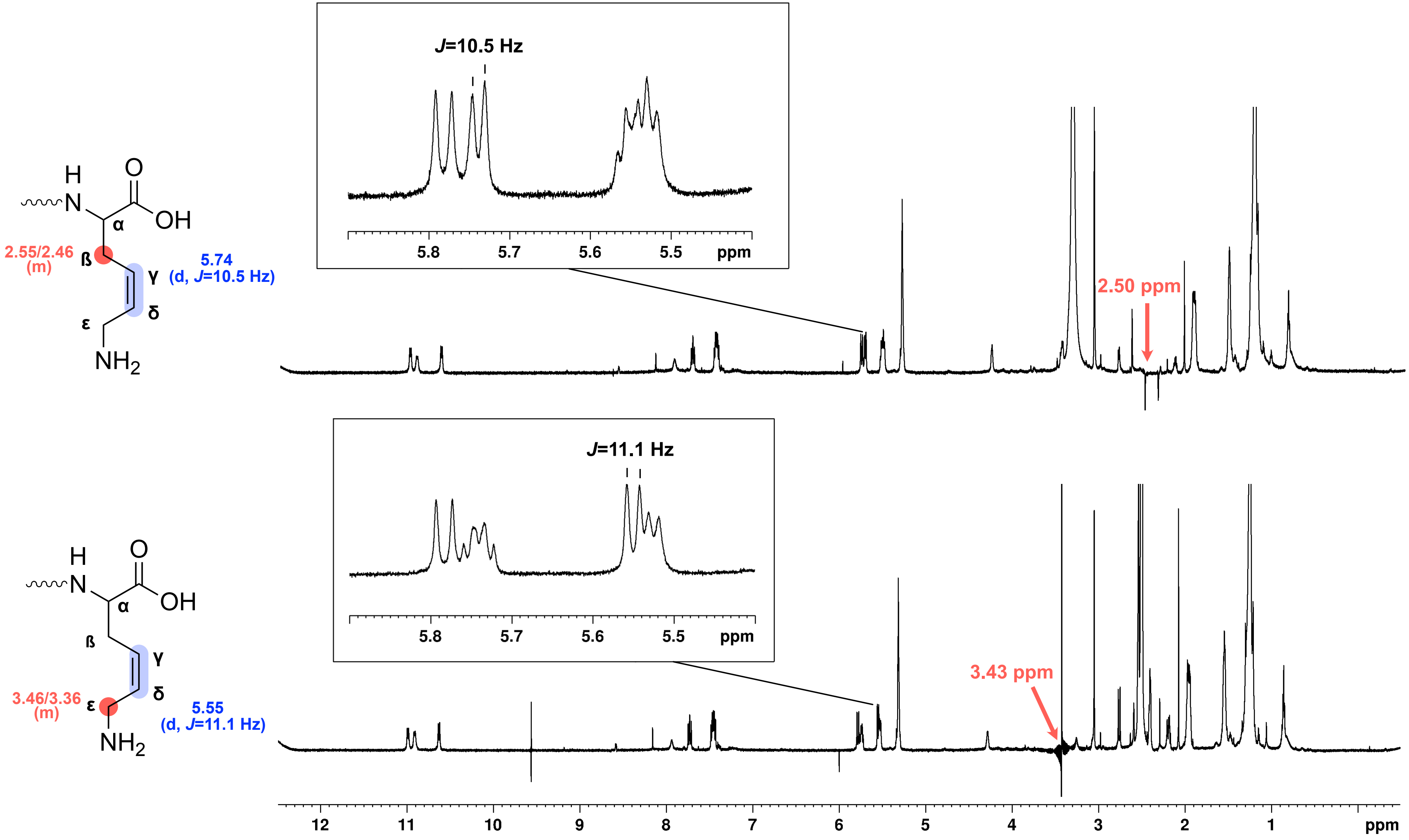

Supplementary Figure 10. ^{1}H-NMR spectra of AFC-BC11 selectively homo-decoupled at 2.50 ppm (***top***) and 3.43 ppm (***bottom***). The coupling constant 10.5 Hz observed for Hγ (5.74 ppm) and 11.1 Hz for Hδ (5.55 ppm) with homo-decoupling indicated a *Z*-configurated double bound in DHLys moiety (DMSO-*d*$_6$, 298 K).

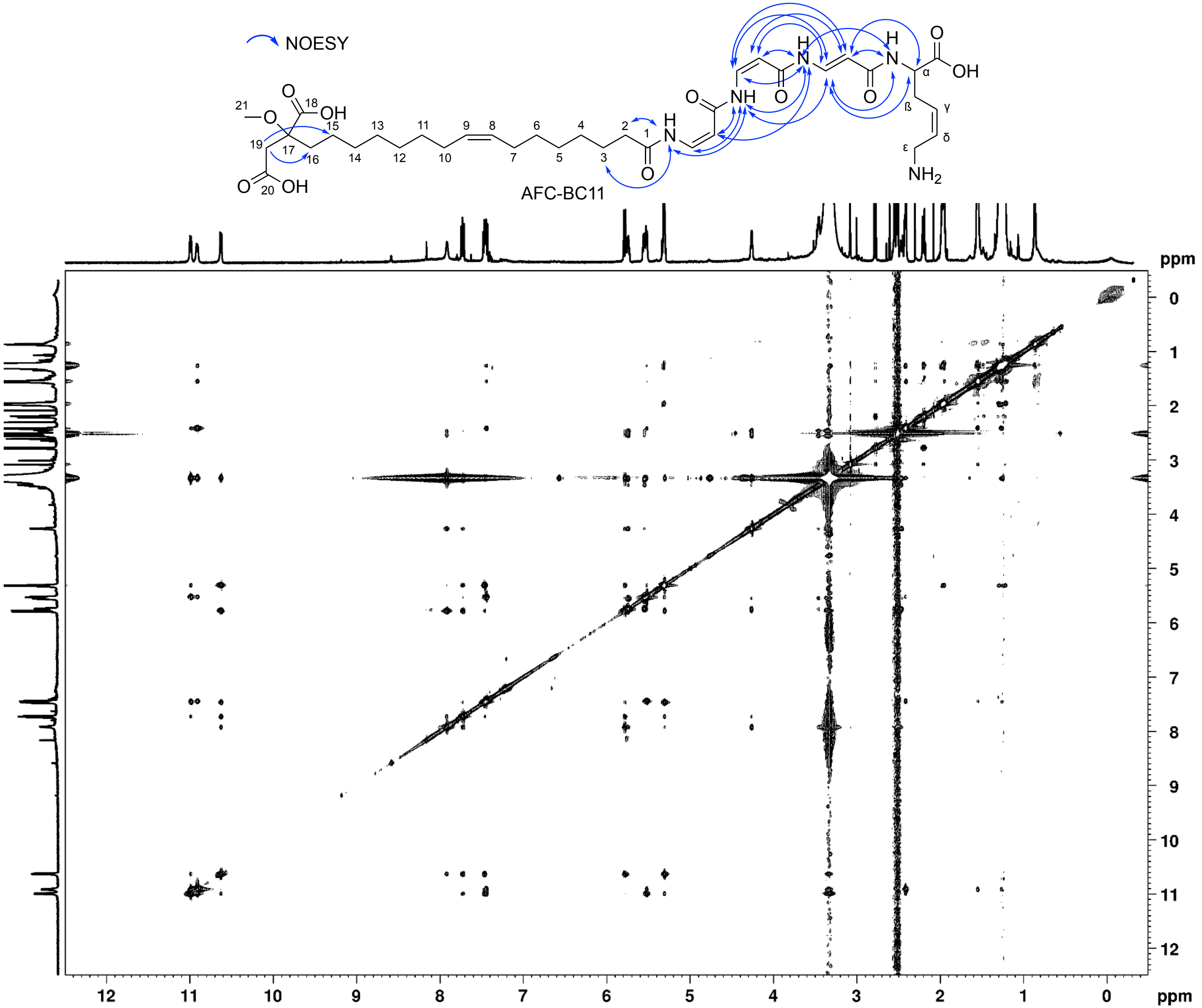

Supplementary Figure 11. ^{1}H-^{1}H NOESY spectrum of AFC-BC11. Key NOE correlations are highlighted in the structure of AFC-BC11 (***top***) (DMSO-d_6, 298 K).

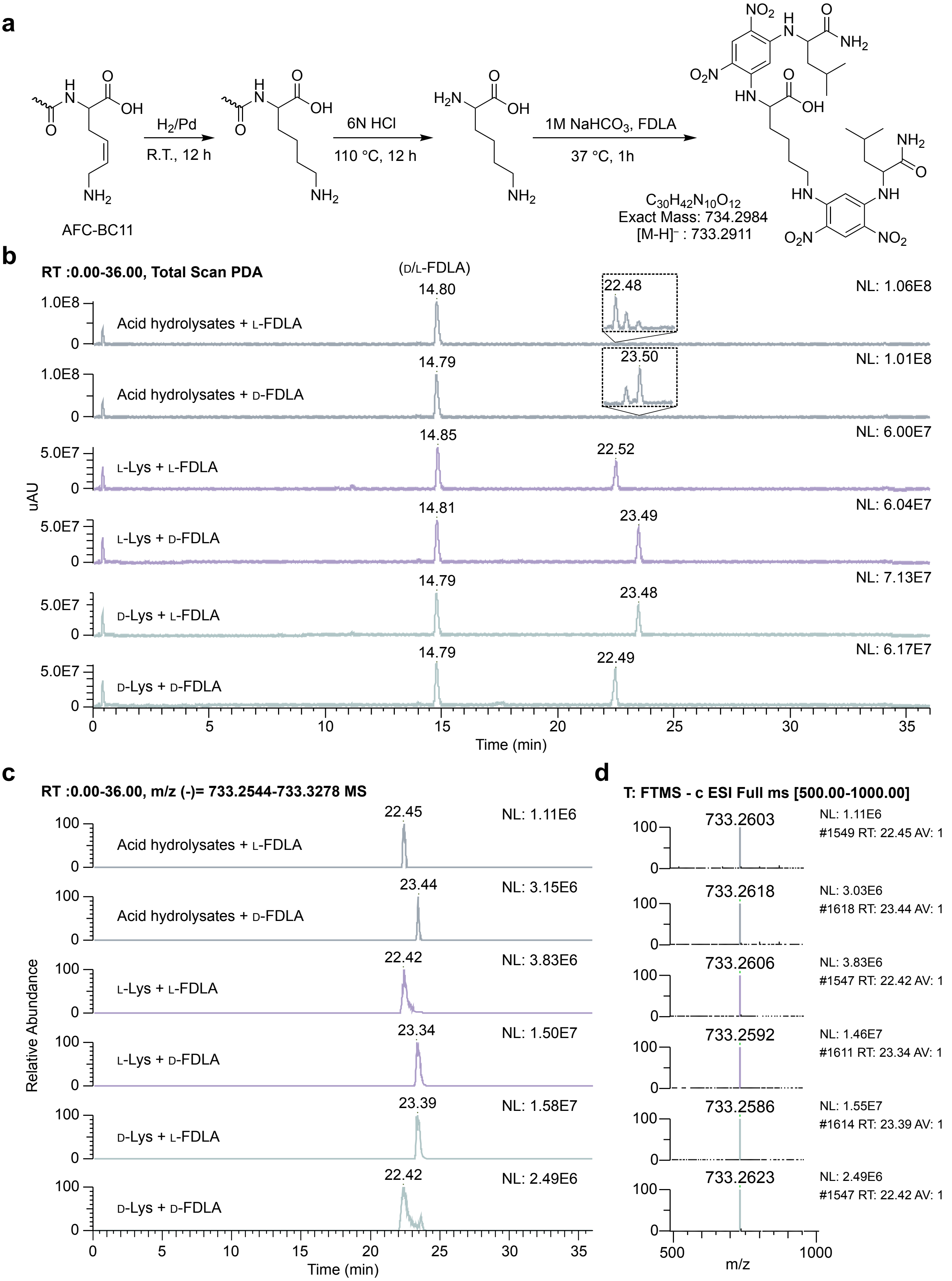

Supplementary Figure 12. Marfey's amino acid analysis. **a**) Reaction schema to obtain FDLA derivatives of lysine from AFC-BC11. **b**) PDA chromatograms. **c**) EIC (-) at *m/z* 733.2911 and **d**) MS spectra of FDLA derivatives of lysine acquired by HPLC-ESI-LTQ-Orbitrap XL mass spectrometer. The calculated mass accuracy for the theoretical $[M-H]^-$ at *m/z* 733.2911 ranged from -39.3 to -44.3 ppm, which falls within the 50 ppm mass accuracy threshold of the Orbitrap instrument in negative ion mode, as determined by daily measurements using a reference compound.

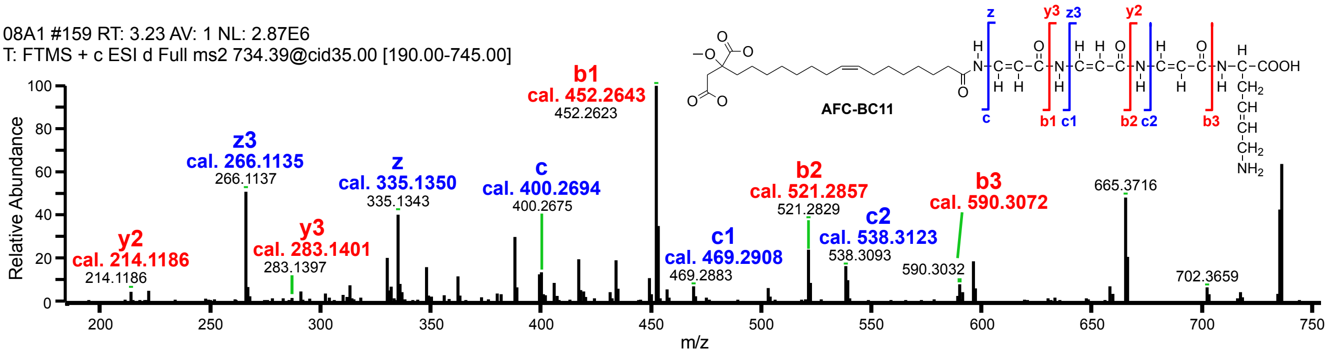

Supplementary Figure 13. ESI (+)-MS/MS of AFC-BC11 acquired by HPLC-ESI-LTQ-Orbitrap XL mass spectrometer.

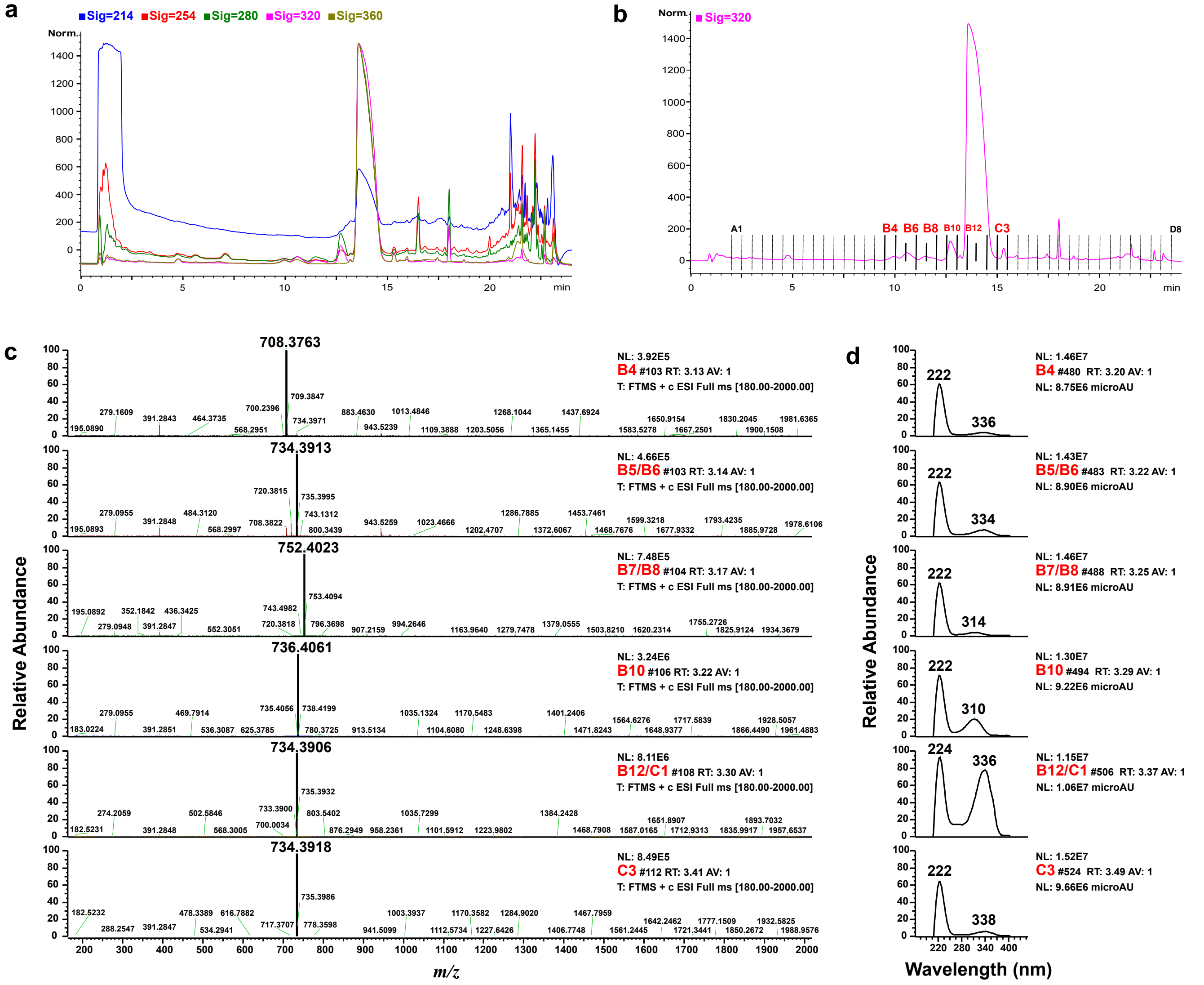

Supplementary Figure 14. Mass screening of the crude extract prepared from the cell pellet of *B. orbicola* Mc0-3. **a**) Analytical HPLC fractionation monitored at wavelengths of λ = 214, 254, 280, 320, and 360 nm. **b**) Time-based fractionation (time slices at 0.5 min) at the wavelength of λ = 320 nm. 44 fractions eluted from 2 to 24 min (A1 to D8) were collected for MS measurements. **c**) Analysis of HRMS profiles acquired by LTQ-Orbitrap XL indicated a set of structurally related $[M+H]^+$ ions. **d**) UV absorption spectra of the corresponding compounds detected.

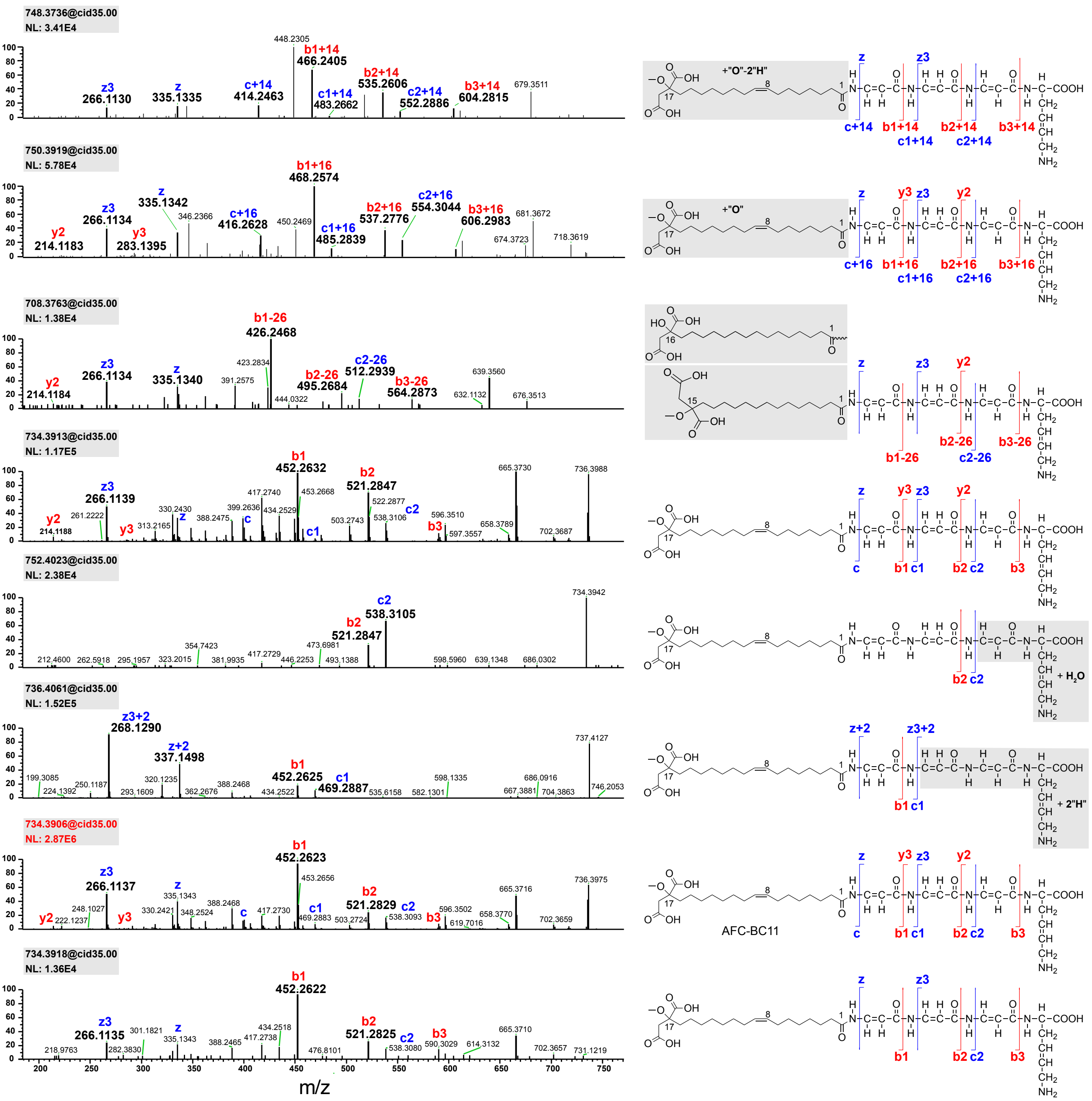

Supplementary Figure 15. ESI (+)-MS/MS of isomers and congeners of AFC-BC11 acquired by LTQ-Orbitrap. Their corresponding linear structures predicted based on MS^2 are shown on the right. The predominant fragments of AFC-BC11 were used as reference and the variable parts are shown in the grey boxes.

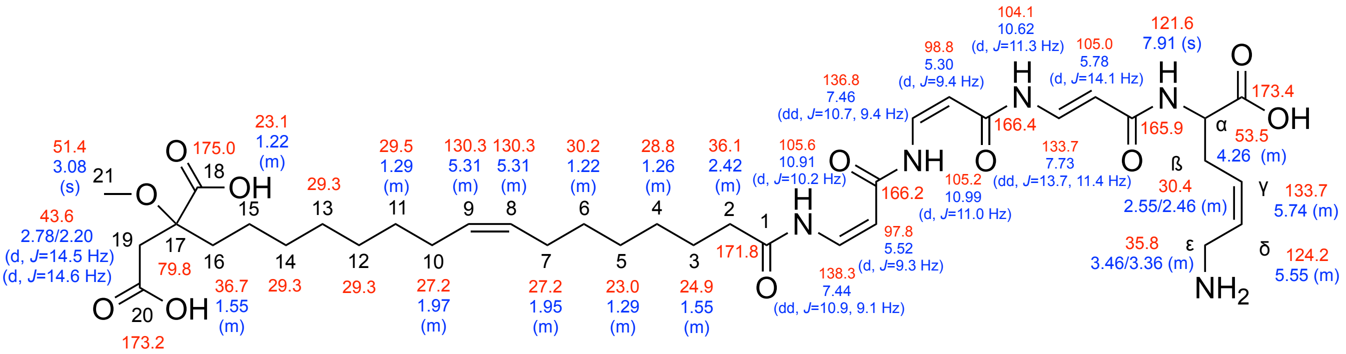

Supplementary Figure 16. The structure of AFC-BC11 with atom numbering used in this study. ^{1}H (***blue***) and ^{13}C/^{15}N chemical shifts (***red***) are given in ppm. Scalar coupling information is also indicated in parenthesis if available.

Supplementary Table 4. NMR data of AFC-BC11 (700 MHz, DMSO-d_6, 298K).

**Position**		**δ (^{1}H) in ppm (multiplicity, J_{HH})**	**δ (^{13}C) in ppm**	**δ (^{15}N) in ppm**	**Intergral (^{1}H)**	**HMBC (^{1}H -^{13}C)**
**(*Z*)-^{1}DBA**	CO	/	166.2	/	/	/
	α	5.52 (d, *J*=9.3 Hz)	97.8	/	1.07	/
	β	7.44 (dd, *J*=10.9, 9.1 Hz)	138.3	/	2.35 (ol)[a]	(*Z*)-^{1}DBA (CO)
	NH	10.91 (d, *J*=10.2 Hz)	/	105.6	0.92	/
**(*Z*)-^{2}DBA**	CO	/	166.4	/	/	/
	α	5.30 (d, *J*=9.4 Hz)	98.8	/	3.21 (ol)	(*Z*)-^{2}DBA (CO, $C_β$)
	β	7.46 (dd, *J*=10.7, 9.4 Hz)	136.8	/	2.35 (ol)	(*Z*)-^{2}DBA (CO)
	NH	10.99 (d, *J*=11.0 Hz)	/	105.2	1.00	/
**(*E*)-^{3}DBA**	CO	/	165.9	/	/	/
	α	5.78 (d, *J*=14.1 Hz)	105.0	/	1.08	(*E*)-^{3}DBA (CO, $C_β$)
	β	7.73 (dd, *J*=13.7, 11.4 Hz)	133.7	/	1.31	(*Z*)-^{2}DBA (CO), (*E*)-^{3}DBA (CO)
	NH	10.62 (d, *J*=11.3 Hz)	/	104.1	1.06	(*Z*)-^{2}DBA (CO)
**(*Z*)-^{4}DHLys**	CO	/	173.4	/	/	/
	α	4.26 (m)	53.5	/	1.27	(*Z*)-^{4}DHLys (CO)
	β	2.55 (m)/2.46(m)	30.4	/	ol/1.09	(*Z*)-^{4}DHLys (CO, $C_α$, $C_δ$, $C_γ$)
	γ	5.74 (m, *J*=11.0 Hz)	133.7	/	1.22	(*Z*)-^{4}DHLys ($C_ε$)
	δ	5.55 (m, *J*=11.0 Hz)	124.2	/	1.13	(*Z*)-^{4}DHLys ($C_β$)
	ε	3.46 (m)/3.36(m)	35.8	/	1.32/ol	(*Z*)-^{4}DHLys ($C_δ$)
	NH	7.91 (br)	/	121.6	1.20	/
**MMFA**[b]	1	/	171.8	/	/	/
	2	2.42 (m)	36.1	/	2.11	MMFA (C_1, C_3, C_4)
	3	1.55 (m)	24.9	/	4.48 (ol)	MMFA (C_1, C_2, C_4, C_5, C_6)
	4	1.26 (m)	28.8	/	ol	MMFA (C_2, C_3)
	5	1.30 (m)	23.0	/	ol	MMFA (C_6)
	6	1.22 (m)	30.2	/	ol	MMFA (C_5, C_7, C_8)
	7	1.95 (m)	27.2	/	2.35	MMFA (C_5, C_6, C_8)
	8	5.31 (m)	130.3	/	3.21 (ol)	MMFA (C_6, C_7)
	9	5.31 (m)	130.3	/	3.21 (ol)	MMFA (C_{10}, C_{11})
	10	1.97 (m)	27.2	/	2.38	MMFA (C_9, C_{11})
	11	1.29 (m)	29.5	/	ol	MMFA (C_9, C_{10})
	12	~1.25	29.3-30.0	/	ol	/
	13	~1.25	29.3-30.0	/	ol	/
	14	~1.25	29.3-30.0	/	ol	/
	15	1.23 (m)	23.1	/	ol	MMFA (C_{16})
	16	1.55 (m)	36.7	/	4.48 (ol)	MMFA (C_{15}, C_{17}, C_{18}, C_{19})
	17	/	79.8	/	/	/
	18	/	175.0	/	/	/
	19	2.78 (d, *J*=14.5 Hz)/2.20 (d, *J*=14.6 Hz)	43.6	/	1.23/1.40	MMFA (C_{16}, C_{17}, C_{18}, C_{20})
	20	/	173.2	/	/	/
	21	3.08 (s)	51.4	/	3.24	MMFA (C_{17})

[a] Overlapped. [b] *O*-methylated malic acid-fatty acid.

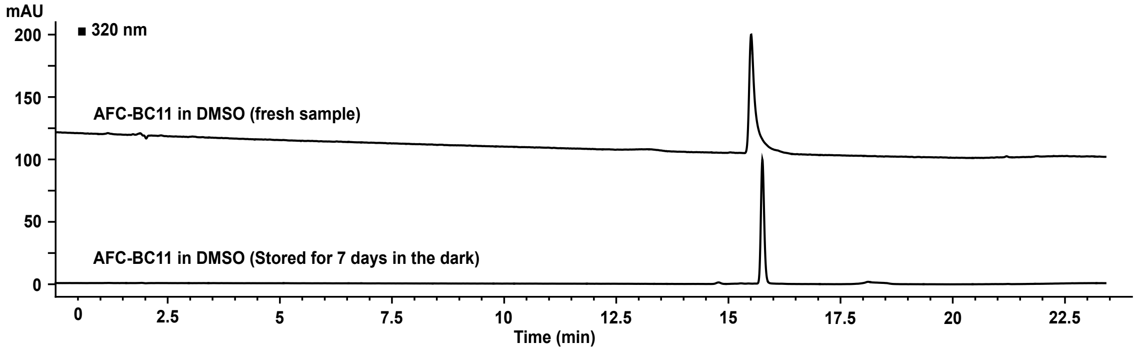

Supplementary Figure 17. AFC-BC11 is stable when stored in the dark. Analytical HPLC chromatograms of the pure AFC-BC11, which have been acquired directly after being dissolved in DMSO (***top***) and stored for 7 days in the dark (***bottom***).

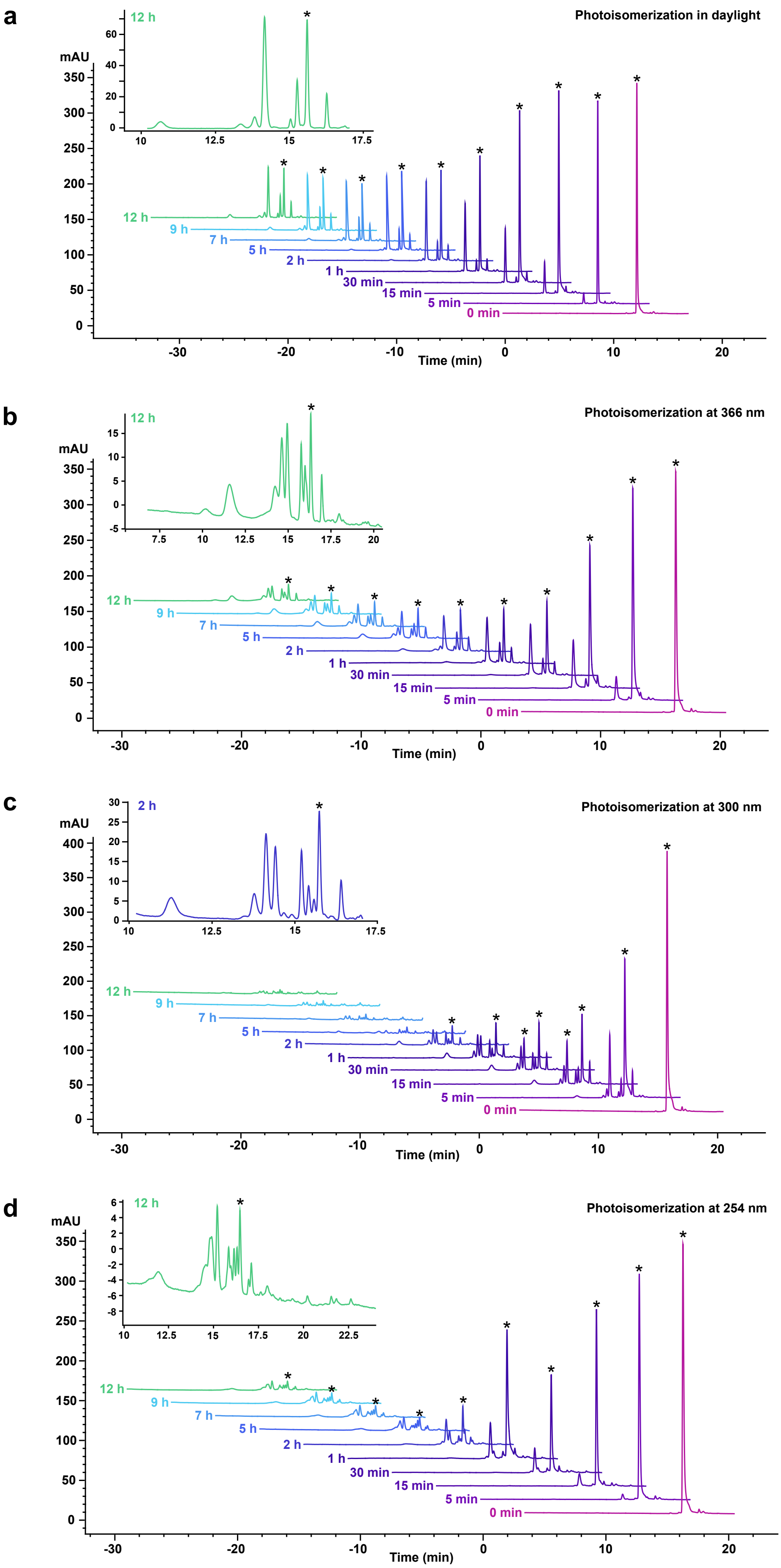

Supplementary Figure 18. Monitoring of the photoisomerization of AFC-BC11 (***asterisk***) by analytical HPLC at the wavelength of λ = 320 nm. **a**) Irradiation by daylight, **b**) at λ = 366 nm (UV-A), **c**) at λ = 300 nm (UV-B), **d**) at λ = 254 nm (UV-C). Zoomed-in image of analytical HPLC chromatograms acquired after irradiation for 12 h (for 2 h at λ =300 nm) are shown on the top left each.

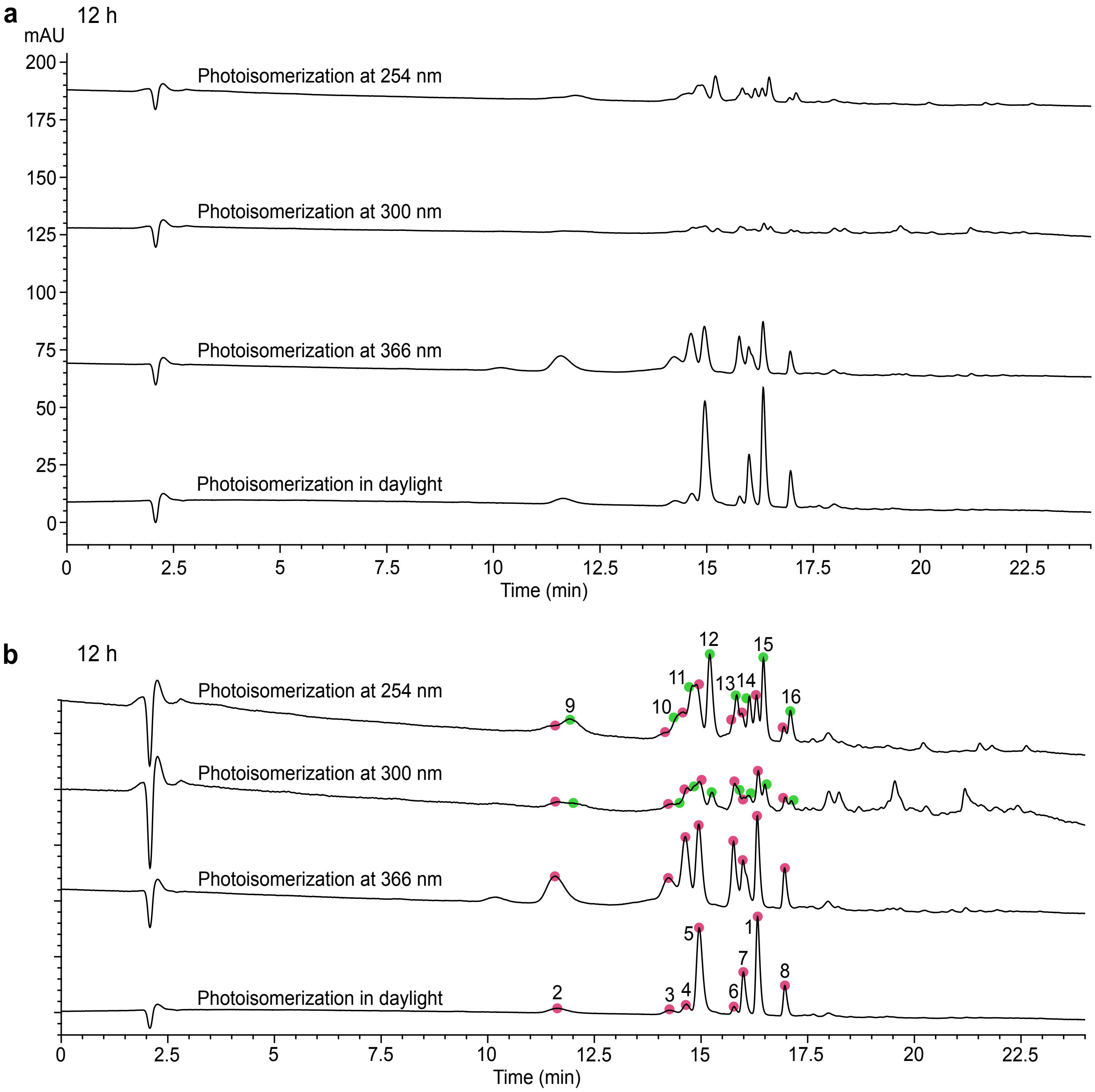

Supplementary Figure 19. Alignment of analytical HPLC chromatograms from Supplementary Figure 18. **a**) Alignment of the absolute UV absorption spectra at the wavelength of λ =320 nm. **b**) Alignment of the corresponding spectra in full scale. The photoisomers occurring after irradiation by daylight and at λ = 366 nm were colored in red and numbered from 1 to 8. Peak 1 is AFC-BC11. Additional photoisomers occurring after irradiation at λ =300 nm and λ =254 nm were colored in green and numbered from 9 to 16.

a

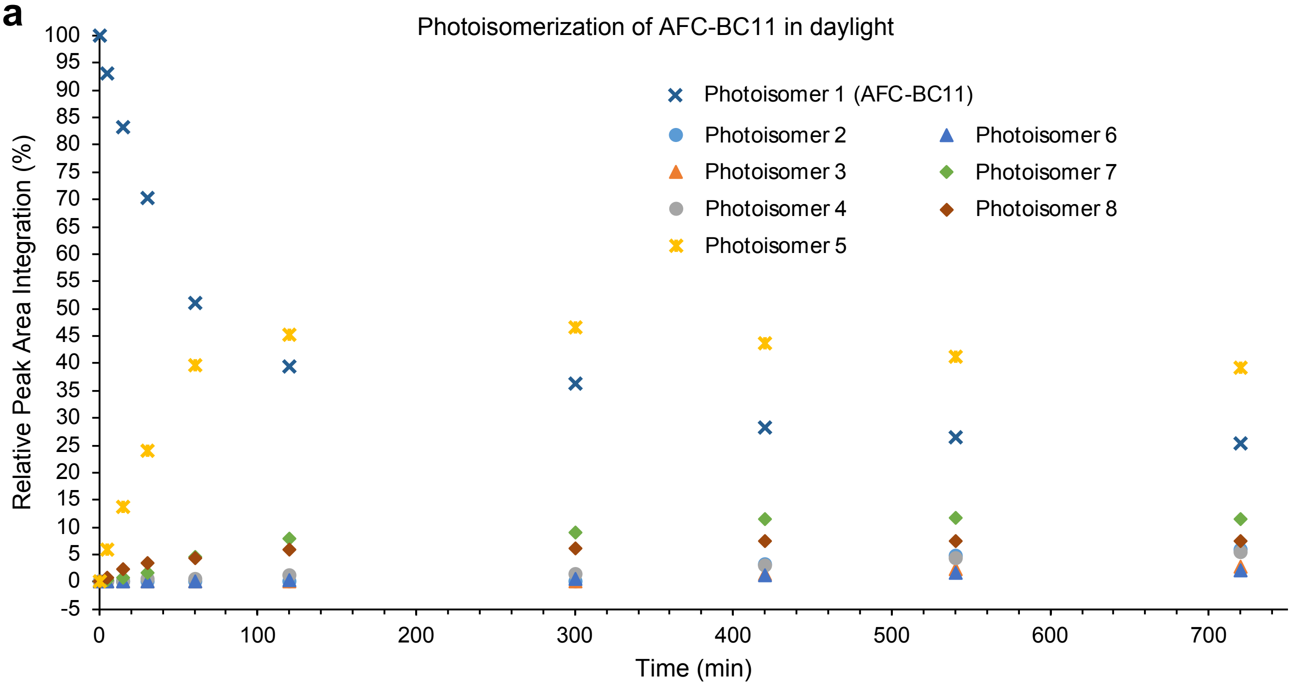

b

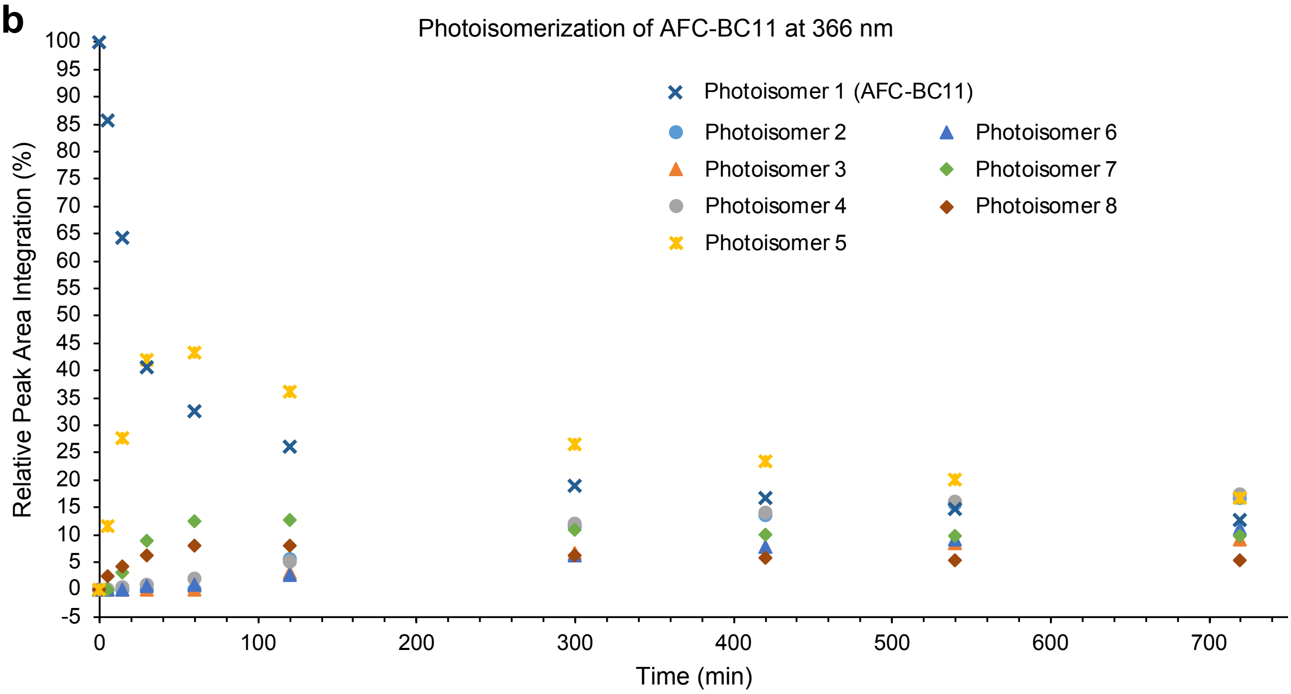

Supplementary Figure 20. Peak area integration of photoisomers after irradiation for 0, 5, 15, 30 min, 1, 2, 5, 7, 9, and 12 h by daylight **a**) and at λ = 366 nm **b**). The calculation was based on Supplementary Figure 18a/b as the compounds were found to be effectively degraded upon irradiation at λ = 300 nm and λ = 254 nm (Supplementary Figure 18c/d). As observed, after 2 h of exposure to daylight, the relative integration of the main photoisomer 5 (45.3%) exceeded that of AFC-BC11 (photoisomer 1, 39.5%) (***top***). This trend was even more pronounced when AFC-BC11 was subjected to λ = 366 nm irradiation, with photoisomer 5 and 1 reaching integration levels of 42.0% and 40.8%, respectively, after 30 minutes of exposure (***bottom***).

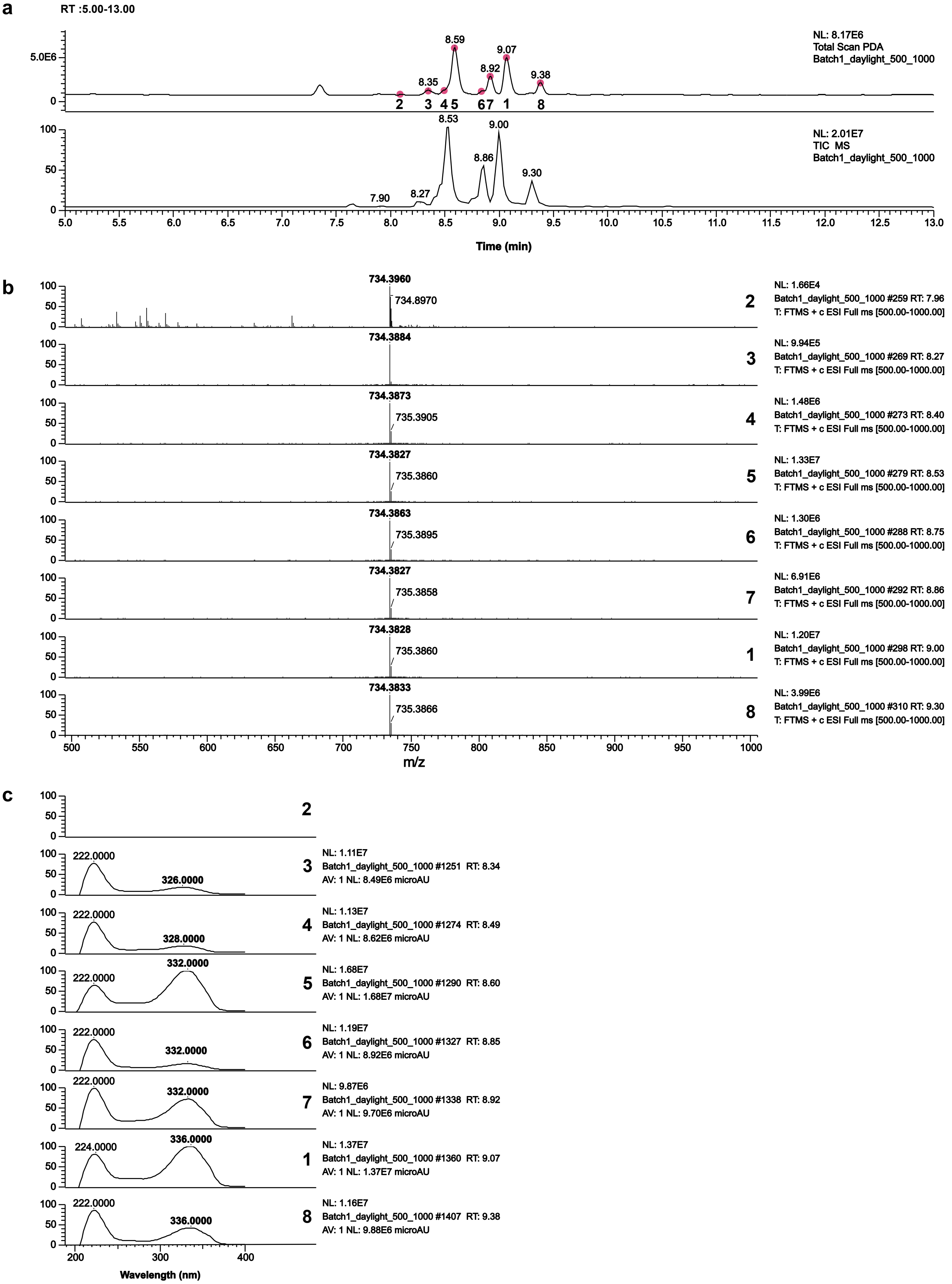

Supplementary Figure 21. PDA and MS spectra of AFC-BC11 acquired by LTQ-Orbitrap XL after irradiation by daylight for 12 h. **a**) Total scan PDA and TIC. The corresponding eight photoisomers were numbered from 1 to 8 as described in Supplementary Figure 19b. Peak 1 is AFC-BC11. *m/z* **b**) and UV absorption spectra **c**) detected for eight photoisomers. UV spectra was not acquired for peak 2 because of the low concentration.

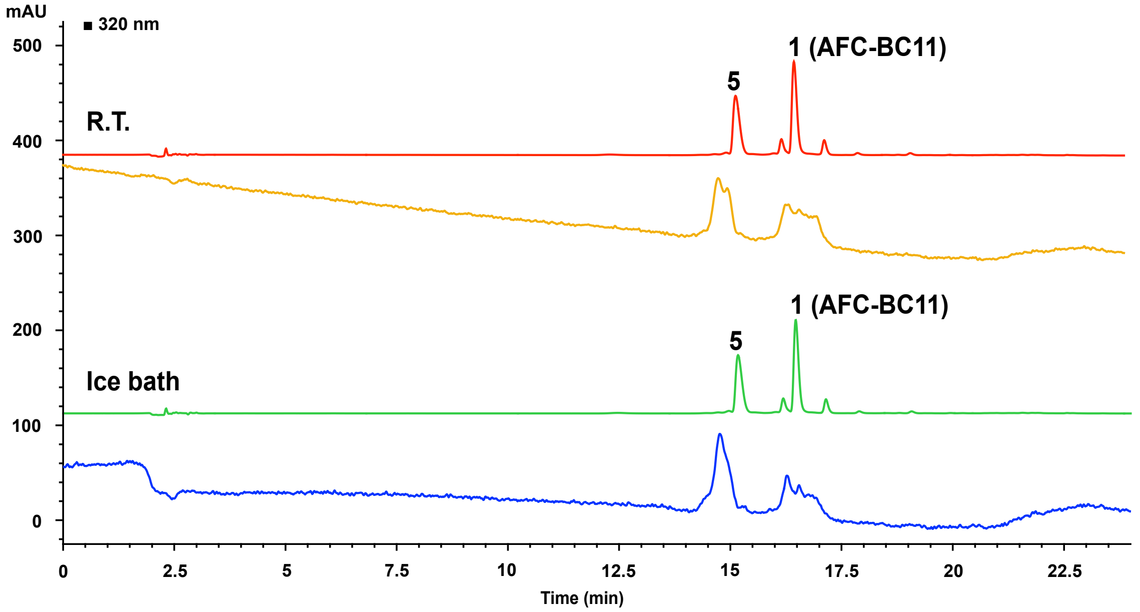

Supplementary Figure 22. Photoisomer 5 is instable for purification. Analytical HPLC chromatograms of the pure AFC-BC11 after irradiation at λ = 366 nm for 45 min (***red***) and of photoisomer 5 collected and directly reinjected (***orange***), which were acquired at room temperature. The analysis procedure was repeated under identical conditions, employing mobile phases that had been pre-cooled in an ice bath (***green*** and ***blue***). The initial purification attempts of the main photoisomer 5 proved its greater inherent instability in comparison to the precursor compound despite employing light-protected and low-temperature conditions.

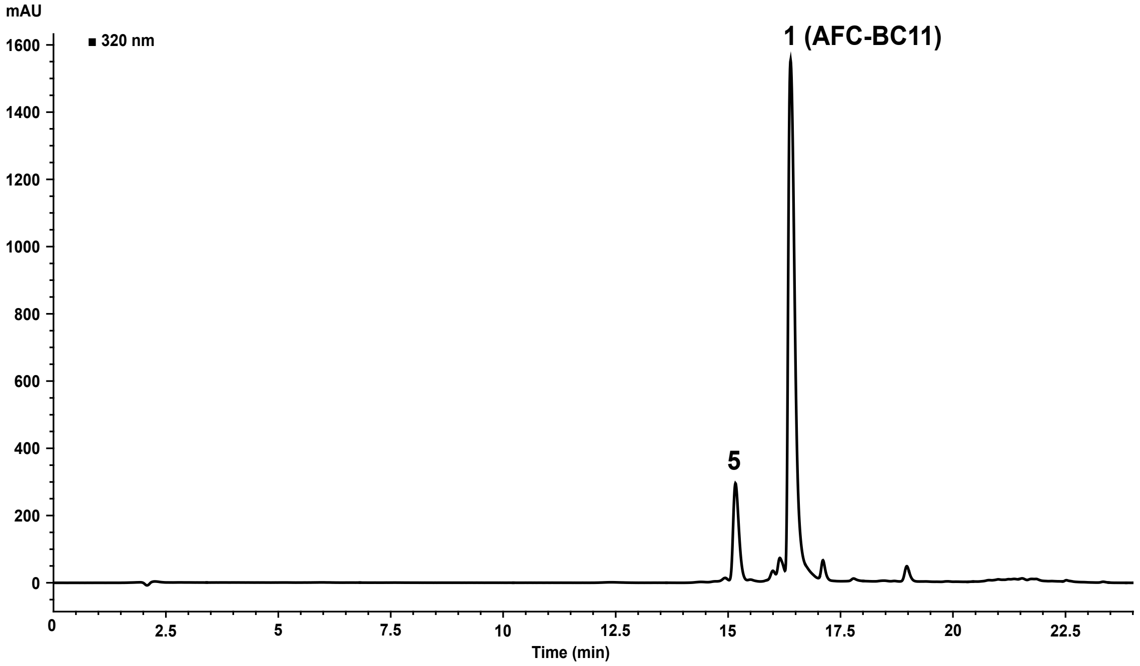

Supplementary Figure 23. HPLC analysis of the mixture of AFC-BC11 and its main photoisomer 5 for NMR. Analytical HPLC chromatogram of the pure AFC-BC11, which has been irradiated at λ = 366 nm for 45 min, followed by NMR measurements. For sample preparation, 1.6 mg of pure AFC-BC11 was dissolved in 600 µL of DMSO-d_6 and the injection volume was 5 µL.

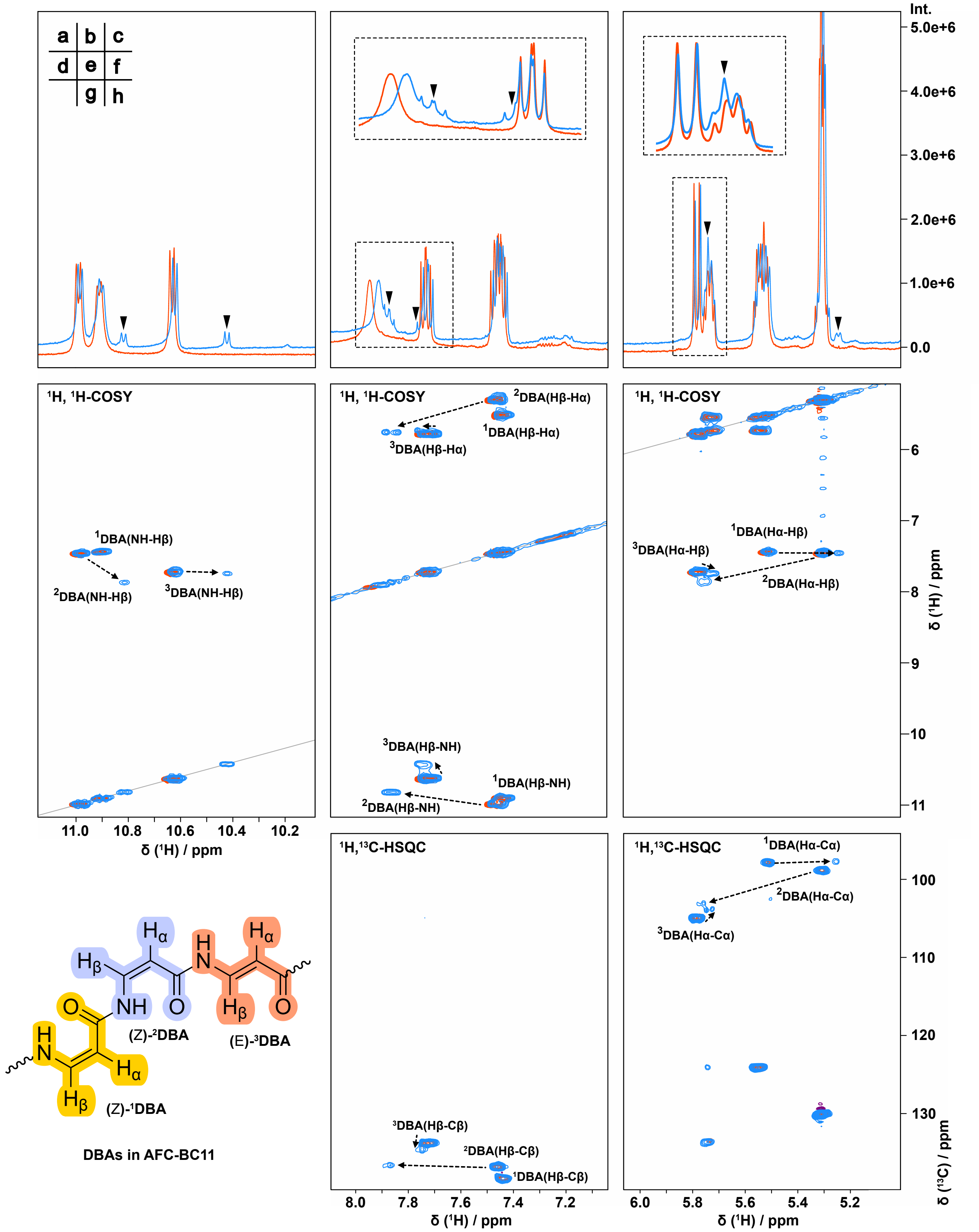

Supplementary Figure 24. Overlay of NMR spectral sections of AFC-BC11 acquired prior (***red***) and after irradiation at λ = 366 nm for 45 min (***blue***). **a**)/**b**)/**c**) Overlay of ^{1}H-NMR spectra with chemical shift ranges at 10.08-11.14 ppm, 7.04-8.10 ppm, and 5.01-6.07 ppm, respectively. Zoomed-in images of peaks were shown on the top in the dashed boxes. The new peaks are highlighted with black arrows. **d**)/**e**)/**f**) Overlay of ^{1}H-^{1}H COSY sections with the corresponding chemical shift ranges. **g**)/**h**) Overlay of ^{1}H-^{13}C HSQC sections with the corresponding chemical shift ranges. Cross peaks belonging to DBAs are annotated. Chemical shift changes are represented by the dashed arrows from AFC-BC11 to the new photoisomer (DMSO-d_6, 298 K).

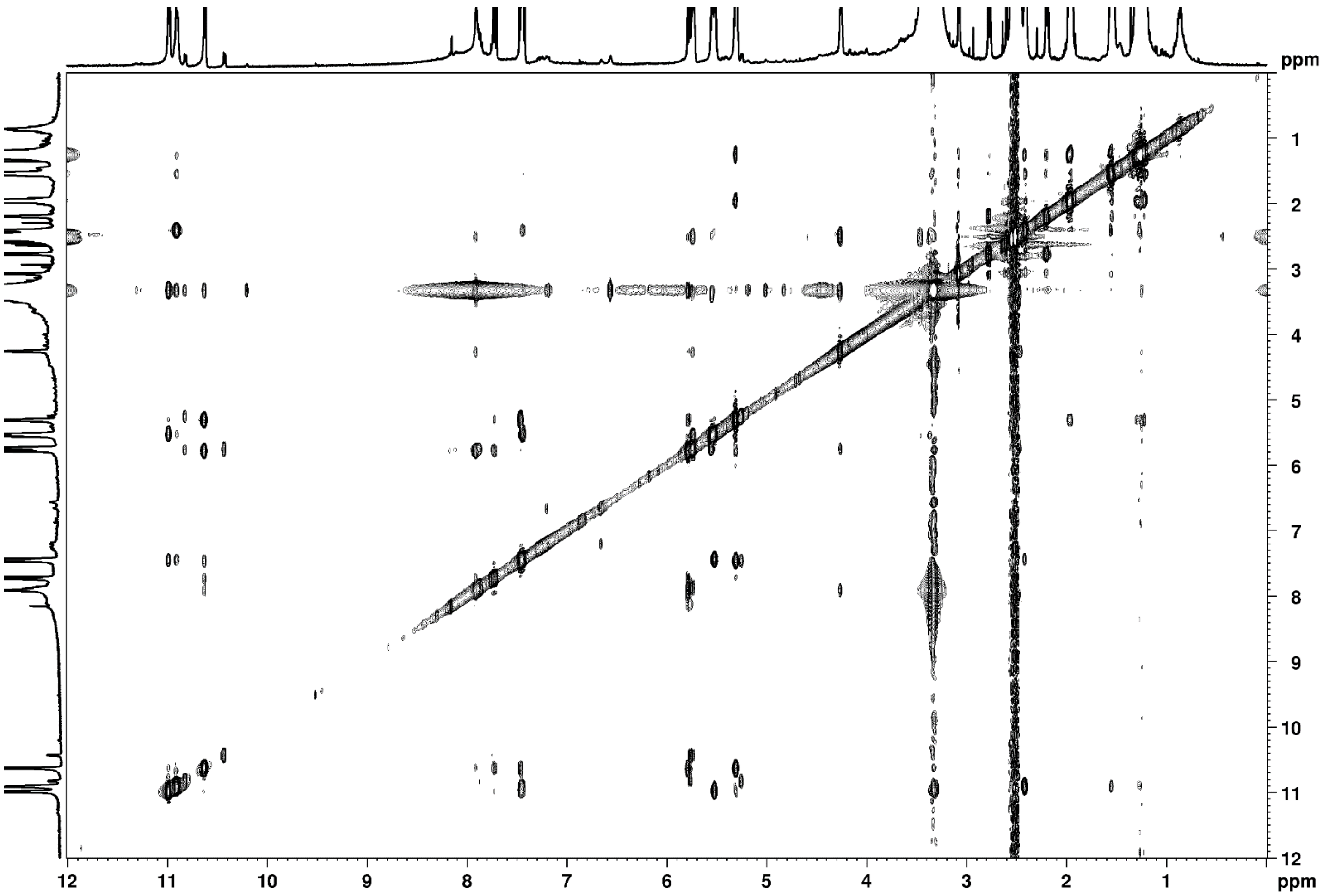

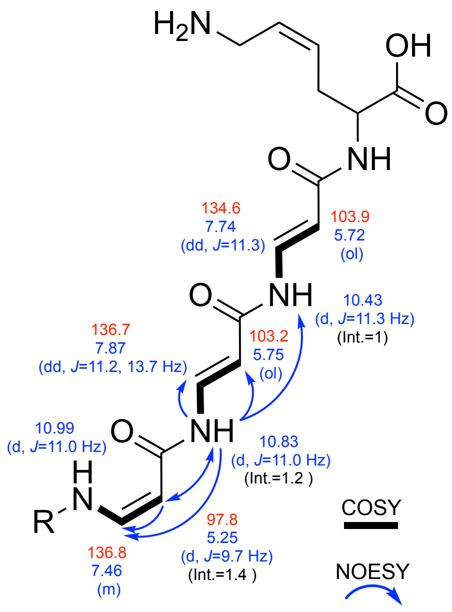

Photoisomer 5

Supplementary Figure 25. ^{1}H-^{1}H NOESY spectrum of AFC-BC11 after irradiation at λ = 366 nm for 45 min. The structure of the main photoisomer 5 with key COSY and NOE correlations, and with annotations of the chemical shifts in DBAs (DMSO-d_6, 298 K). (R=MMFA, *O*-methylated malic acid-fatty acid)

Supplementary Table 5. MICs (µg/mL) of AFC-BC11 against selected phytopathogenic fungi depending on UV irradiation.

Fungal strain	Replicate	Irradiation time at λ = 366 nm (min)				Azoxystrobin[a]
		0	5	15	30	
***Colletotrichum kahawae* CM732**	1	0.24	0.32	0.56	0.64	0.64
	2	0.24	0.32	0.56	0.64	0.72
	3	0.24	0.32	0.56	0.64	0.72
	**Proposed MIC[b]**	**0.24**	**0.32**	**0.56**	**0.64**	**0.72**
***Pyrenophora teres* f. *teres* Hun0005**	1	0.32	0.40	0.72	0.88	0.96
	2	0.32	0.40	0.72	0.88	0.96
	3	0.32	0.40	0.72	0.88	0.96
	**Proposed MIC**	**0.32**	**0.40**	**0.72**	**0.88**	**0.96**
***Fusarium xylarioides* CAB003**	1	> 2.40	> 2.40	> 2.40	> 2.40	2.40
	2	> 2.40	> 2.40	> 2.40	> 2.40	2.40
	3	> 2.40	> 2.40	> 2.40	> 2.40	2.40
	**Proposed MIC**	**> 2.40**	**> 2.40**	**> 2.40**	**> 2.40**	**2.40**
***Pyricularia* (*Magnaporthe*) *oryzae* Guy11**	1	0.24	0.32	0.32	0.40	0.24
	2	0.24	0.32	0.32	0.40	0.24
	3	0.24	0.32	0.32	0.40	0.24
	**Proposed MIC**	**0.24**	**0.32**	**0.32**	**0.40**	**0.24**
***Rhizoctonia solani* CD9001**	1	0.64	ND[c]	ND	ND	> 1.20
	2	0.64	ND	ND	ND	> 1.20
	3	0.72	ND	ND	ND	> 1.20
	**Proposed MIC**	**0.72**	**ND**	**ND**	**ND**	**> 1.20**

[a] As positive control. [b] The highest values were selected as MICs. [c] Not determined.

Supplementary Table 6. MICs (µg/mL) of AFC-BC11 against selected human pathogenic fungi.

Fungal strain	AFC-BC11	Voriconazole[a]
Aspergillus fumigatus ATCC 205304	> 64	0.5
Candida albicans NRZ-2024-0602	> 64	0.25
Candida glabrata NRZ-2024-0605	> 64	0.125
Candida krusei NRZ-2024-0601	> 64	2
Candida parapsilosis ATCC 22019	> 64	0.03
Fusarium solani NRZ-2024-0590	> 64	> 8
Lichtheimia corymbifera NRZ-2024-0649	> 64	> 8
Rhizomucor pusillus NRZ-2024-0631	> 64	4
Scedosporium apiospermum NRZ-2024-0680	> 64	1

[a] As positive control.

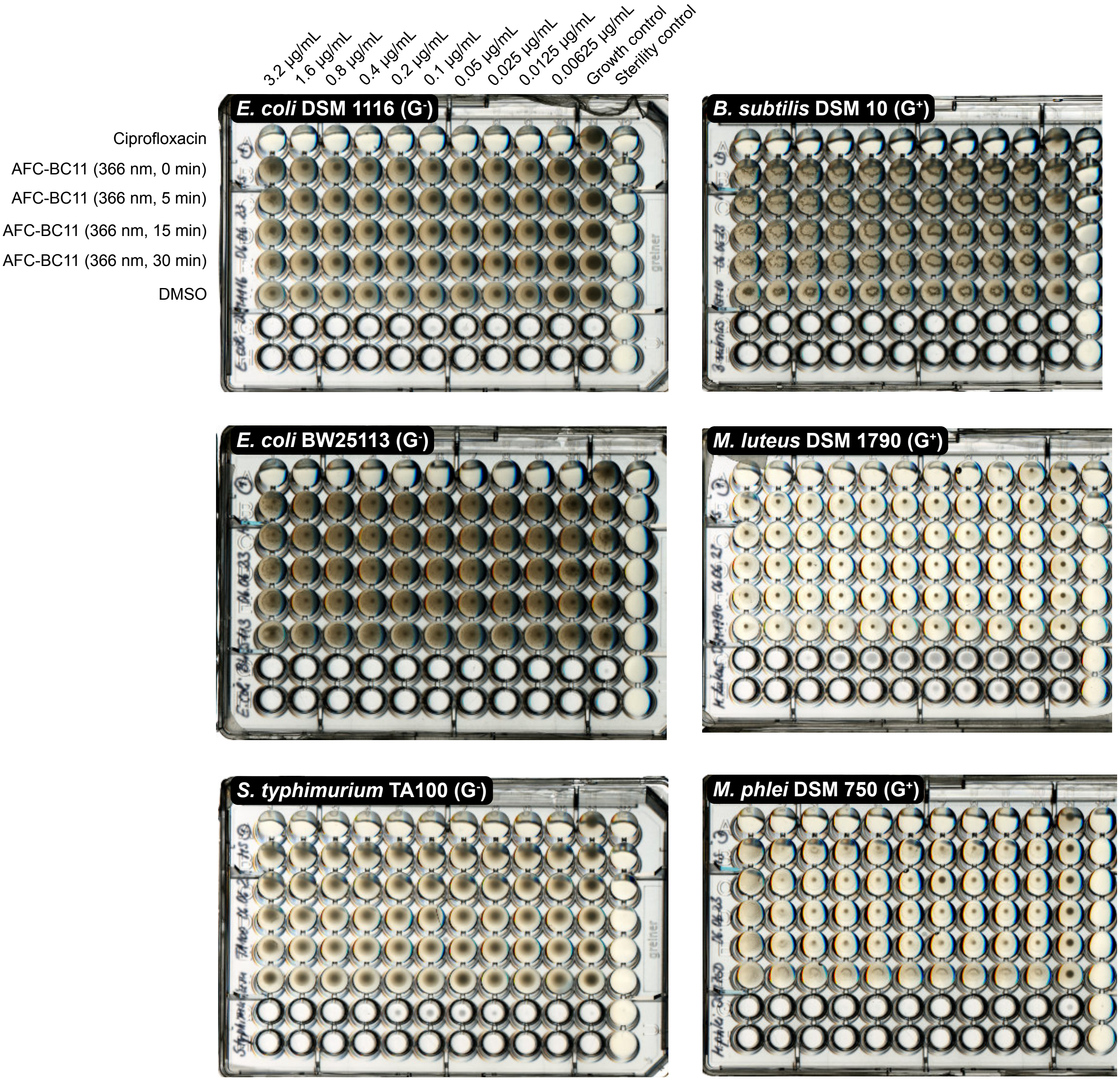

Supplementary Figure 26. MIC assays performed to evaluate the impact of photoisomerization on biological activities of AFC-BC11 against gram-negative and gram-positive strains. Broth microdilution assays were performed in duplicate.

Supplementary Table 7. Overview of proteins purified in this study.

Protein	Construct	MW (Da)[a]	Ext. coefficient	Conc. (mg/mL)	Conc. (µM)	Yield (mg/L)
AfcQ	pET28_mc03_afcQ	58809.90	51715	34.6	588.3	57.1
AfcA	pET28_mc03_afcA	65130.98	48985	27.3	419.2	14.3
AfcK	pET28_mc03_afcK	11775.04	4470	21.1	1791.9	26.4
AfcP	pET28_mc03_afcP	44254.14	34755	15.1	341.0	5.0
AfcL	pET28_mc03_afcL	33146.55	42775	2.9	87.5	2.6
Sfp	pET15b_WT_sfp	28777.53	30620	4.7	163.3	10.6

[a] Theoretical protein mass including the N-terminal His_6-tag (MW: 2635.77 Da).

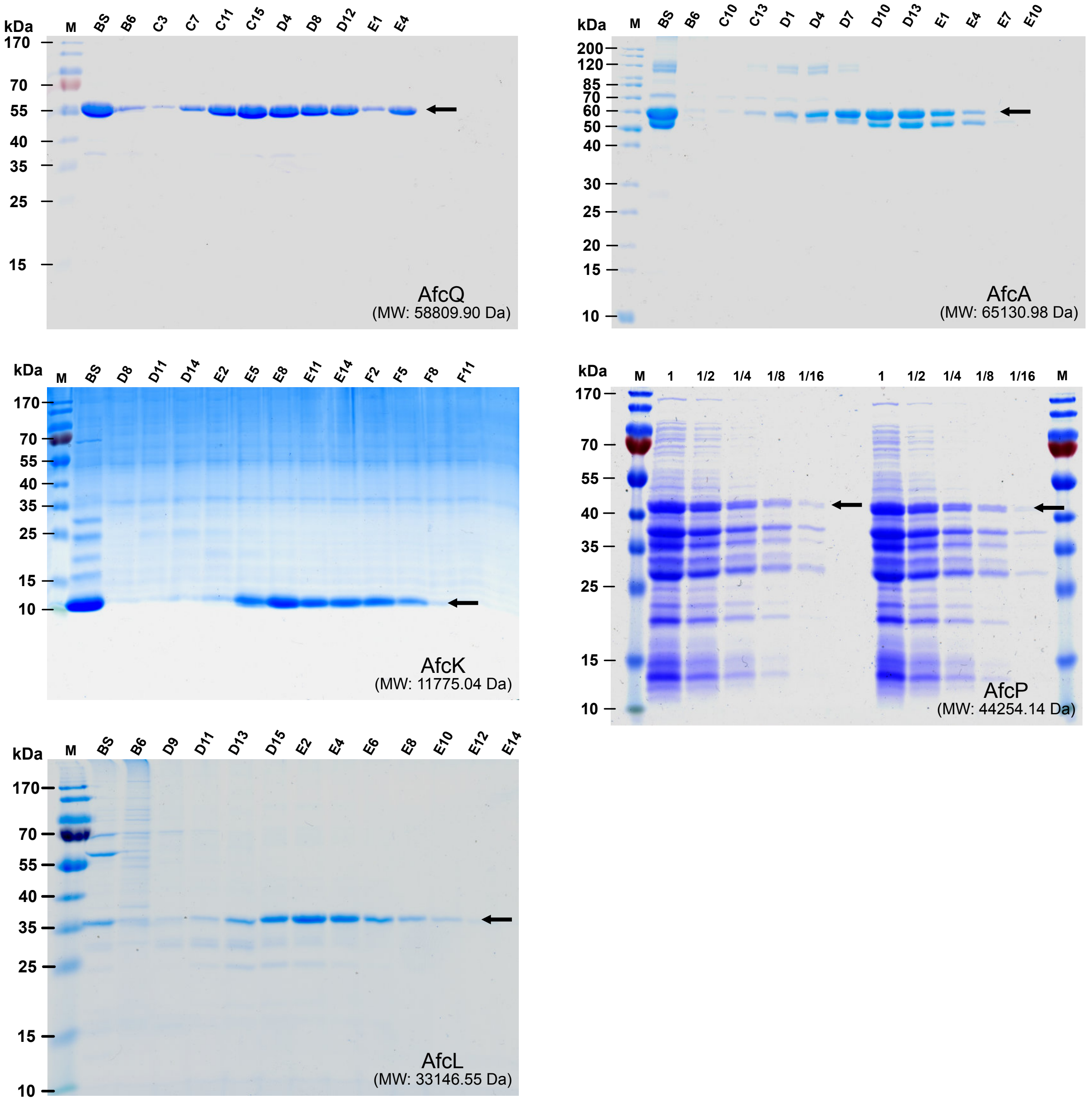

Supplementary Figure 27. Protein purification of N-terminal His$_6$-tagged Afc proteins. SDS-PAGE of fractions eluted with HiLoad^{TM} 16/60 Superdex^{TM} 200 pg size exclusion column (SEC). Fractions containing the aimed protein were pooled for further concentration. SDS-PAGE of AfcP showed the concentrated protein sample with two-fold serial dilution. The bands of His$_6$-tagged Afc proteins are highlighted with arrows. Despite intensive optimization, AfcA and AfcP yielded only semi-pure samples due to partial degradation. The abbreviations are described as follows: marker (M), concentrated fractions before SEC (BS).

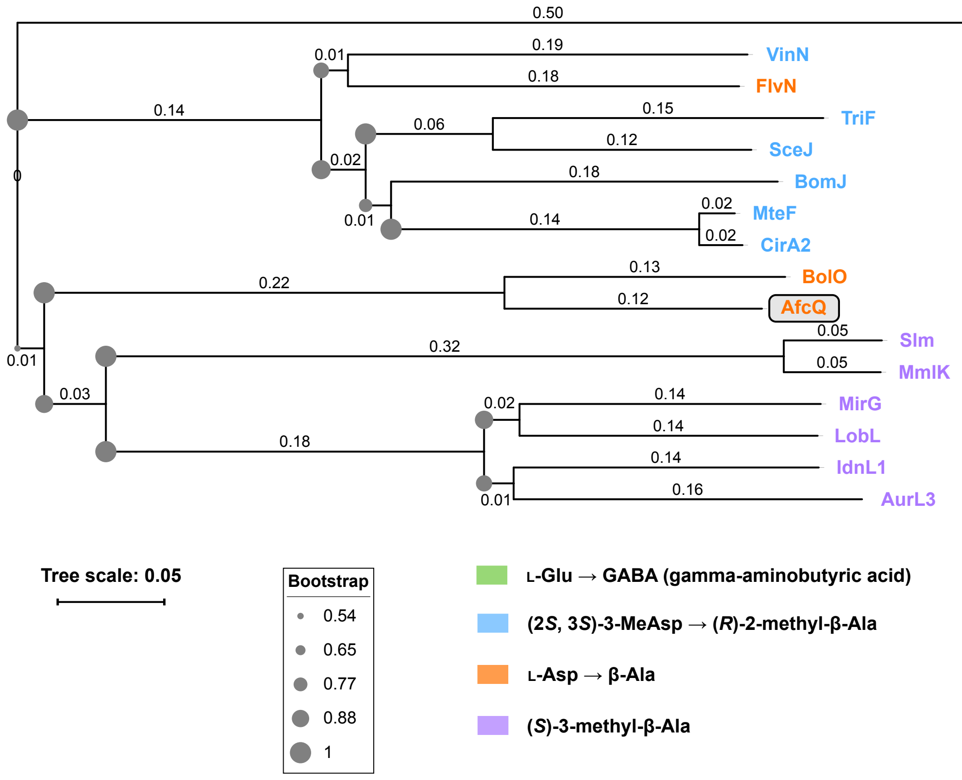

Supplementary Figure 28. Phylogenetic analysis of adenylation (A) domains involved in the bioproduction of β-alanine and its analogs. Based on their native substrates, the 16 A domains are categorized into four subgroups L-Glu, (2*S*, 3*S*)-3-MeAsp, L-Asp, and (*S*)-3-methyl-β-Ala, which are color-coded in green, blue, orange, and lilac, respectively, whereas FlvN is an exception to this classification as FlvN utilizes L-Asp as its native substrate. A domains aligned here were extracted from biosynthetic gene clusters (BGCs) involved in the biosynthesis of butirosin (BtrJ_BAE07074.1),[44] vicenistatin (VinN_BAD08371.1),[45,46] tripartilactam (TriF_QGA70083.1),[47] sceliphrolactam (SceJ_ANH11405.1),[48] bombyxamycin (BomJ_QBL56195.1),[49] macrotermycin (MteF_OAP25805.1),[50] ciromicin (CirA2_UKD51468.1),[51] fluvirucin B_2 (FlvN_BAV56017.1),[52] bolagladin (BolO_QNH85847.1),[42,43] salinilactam (Slm_ABP55216.1),[53] micromonolactam (MmlK_AGI61651.1),[54] mirilactam (MirG_WP_015801512.1),[55,56] lobosamide (LobL_ALA09365.1),[55] incednine (IdnL1_BAP34707.1),[57] and auroramycin (AurL3_AWR88409.1).[58]

Supplementary Table 8. Specificity codes of selected adenylation domains involved in the activation of L-Asp or its analogs.

A domain	Substrate	Adenylation	Natural product	A1	A2	A3	A4	A5	A6	A7	A8	A9	A10
GrsA[a] [b]	L-Phe	α-COOH	Gramicidin S	D^{235}	A^{236}	W^{239}	T^{278}	I^{299}	A^{301}	A^{322}	I^{330}	C^{331}	K^{517}
**AfcQ[c]**	**L-Asp**	**β-COOH**	**AFC-BC11**	$\mathbf{D^{211}}$	$\mathbf{A^{212}}$	$\mathbf{S^{215}}$	$\mathbf{T^{251}}$	$\mathbf{A^{277}}$	$\mathbf{T^{279}}$	$\mathbf{M^{303}}$	$\mathbf{F^{310}}$	$\mathbf{R^{311}}$	$\mathbf{K^{503}}$
FlvN[c]	L-Asp	β-COOH	Fluvirucin B_2	D^{230}	Y^{231}	Y^{234}	P^{270}	T^{297}	T^{299}	M^{323}	K^{330}	R^{331}	K^{510}
DptA3[c]	L-Asp	α-COOH	Daptomycin	D^{194}	L^{195}	T^{198}	K^{228}	L^{251}	G^{253}	A^{277}	V^{285}	C^{287}	K^{476}
IdnL1[b]	(S)-3-methyl-β-Ala	β-COOH	Incednine	D^{216}	F^{217}	L^{220}	N^{259}	L^{287}	S^{289}	C^{313}	V^{320}	A^{321}	K^{514}
VinN[b]	(2S, 3S)-3-MeAsp	β-COOH	Vicenistatin	D^{230}	F^{231}	Y^{234}	P^{270}	T^{297}	S^{299}	M^{323}	K^{330}	R^{331}	K^{510}
SlgN1[b]	(2S, 3S)-3-MeAsp	α-COOH	Streptolydigin	D^{263}	A^{264}	L^{267}	Q^{305}	I^{329}	G^{331}	G^{355}	G^{363}	F^{364}	K^{556}

[a] GrsA as the reference for the specificity-conferring codes (A1-A10). [b] Crystal structures from PDB, GrsA (1AMU),[59,60] IdnL1 (5JJQ),[57,61] VinN (3WV5),[45,62] and SlgN1 (4GR5).[63,64] [c] Structures were predicted by AlphaFold2 and the substrates were modelled by AutoDock Vina, AfcQ (WP_012337012.1), FlvN (BAV56017.1),[52,65] and DptA3 (AHX36919.1).[66] The residues from A2-A9 involved in the interaction and recognition of side chains are color-coded in blue (hydrophilic) and pink (hydrophobic).

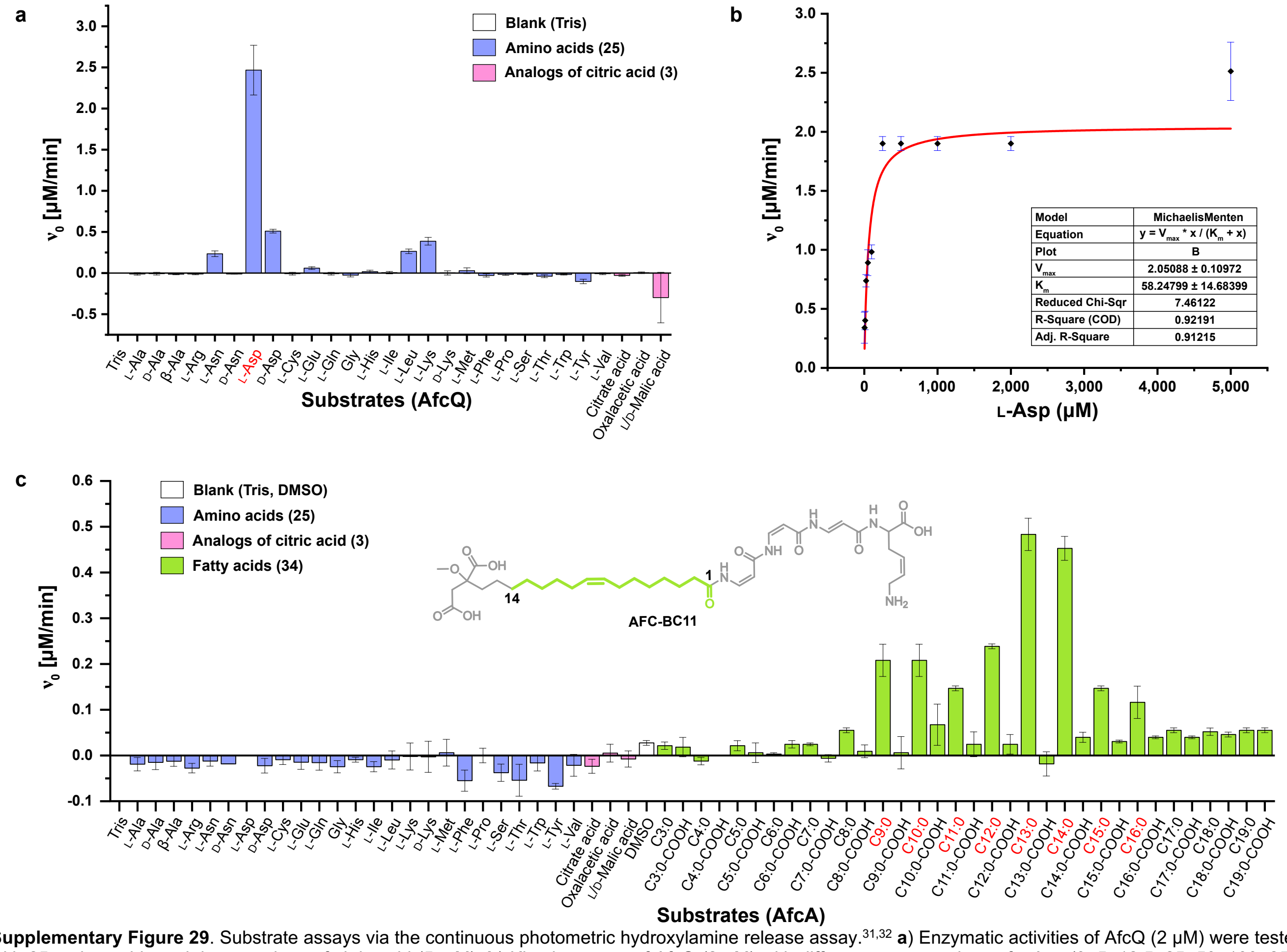

Supplementary Figure 29. Substrate assays via the continuous photometric hydroxylamine release assay.[31,32] **a**) Enzymatic activities of AfcQ (2 µM) were tested with 25 amino acids and three analogs of citric acid (5 mM). **b**) Kinetic assays of AfcQ (2 µM) with different concentrations of L-Asp (0, 5, 12.5, 25, 50, 100, 250, 500, 1,000, 2,000 and 5,000 µM). The inset shows the kinetic parameters from non-linear regression. **c**) Enzymatic activities of AfcA (2 µM) were tested with 25 amino acids and three analogs of citric acid (5 mM), as well as 34 fatty acids (50 µM). All reactions were performed in triplicate and error bars indicate the standard errors.

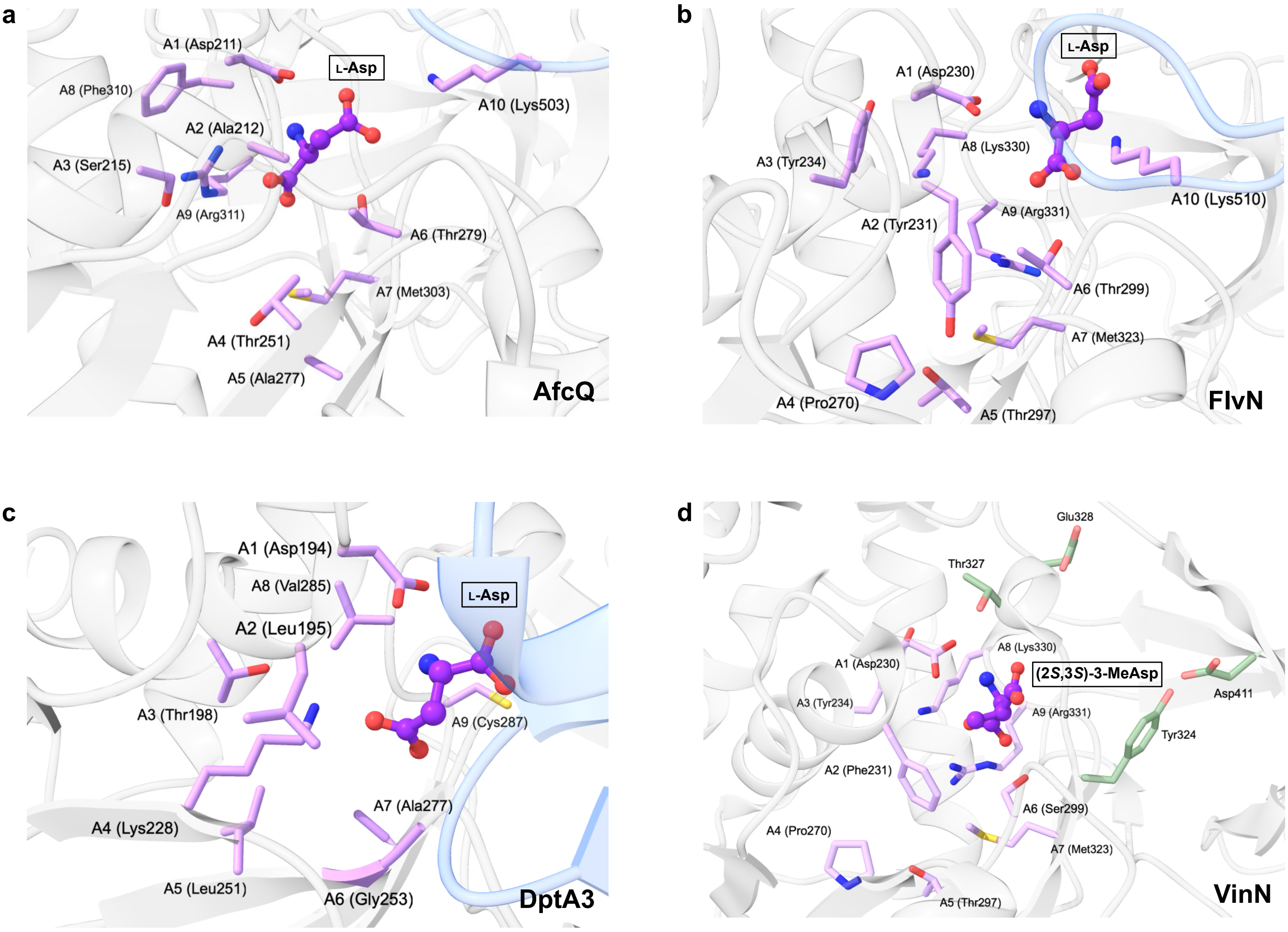

Supplementary Figure 30. Structural analysis of AfcQ. Native substrate L-Asp (***ball stick***) was modelled into the AlphaFold2 predicted protein structures (***cartoon***)[16–20] of **a**) AfcQ (WP_012337012.1), **b**) FlvN (BAV56017.1),[52,65] and **c**) DptA3 (AHX36919.1).[66] **d**) The binding pocket of (2*S*, 3*S*)-3-MeAsp in VinN (PDB: 3WV5).[45,62] The residues (***stick***, ***pink***) indicate the specificity-conferring codes in adenylation domains. Meanwhile, the residues (***stick***, ***green***) indicate the ATP binding sites.

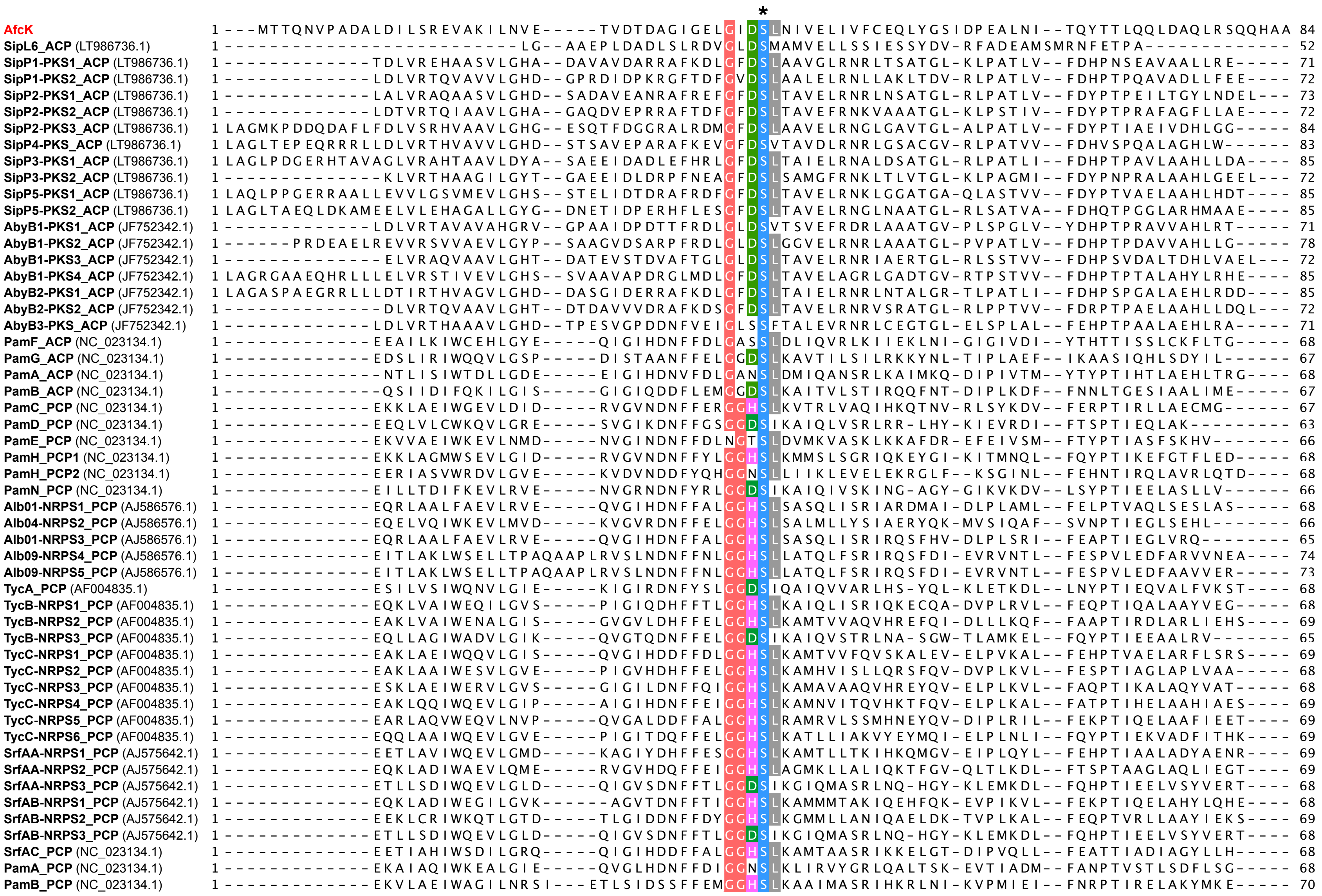

Supplementary Figure 31. Alignment of AfcK with ACPs and PCPs revealed the conserved core motif G_(D/H)SL with the Ser residue (***asterisk***) for phosphopantetheinylation. Notably, the presence of GG residues (***pink***) reflected the enhanced mobility in PCPs.[67] ACPs and PCPs aligned here were extracted from BGCs involved in the biosynthesis of sipanmycin,[68] abyssomicin,[69] paenilamicin[70,71], albicidin,[72] tyrocidine,[67,73] and surfactin.[74]

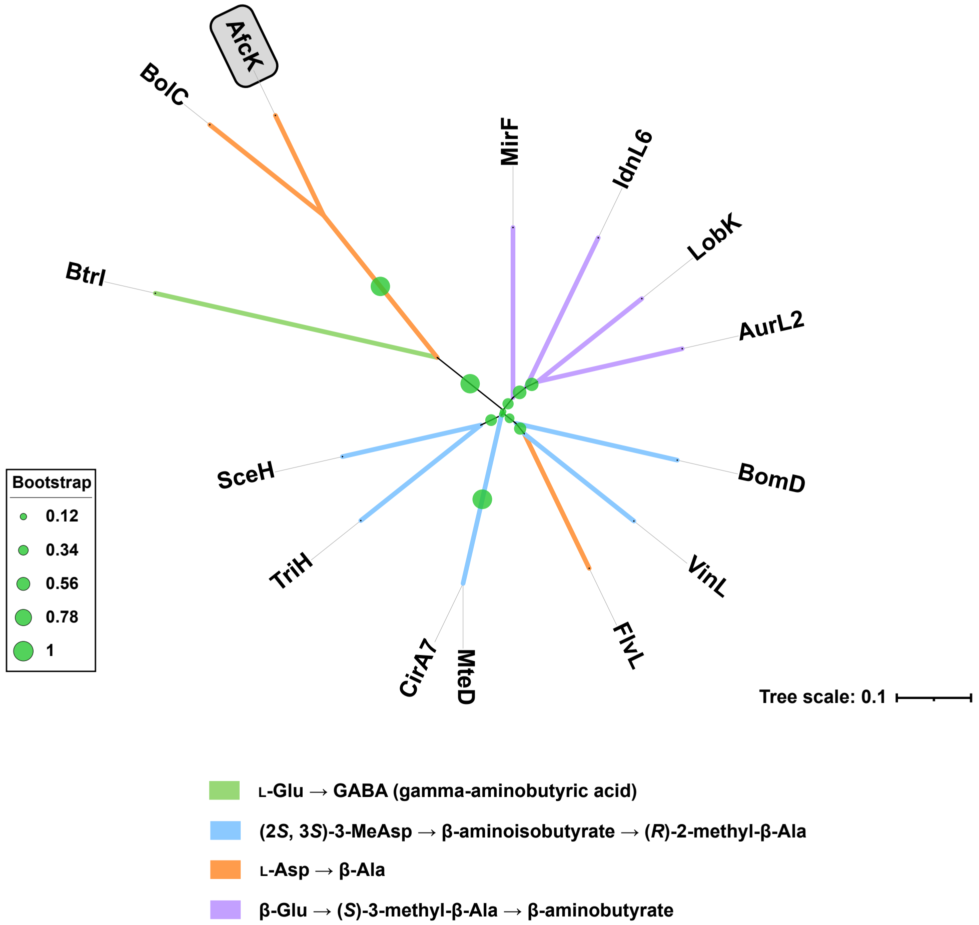

Supplementary Figure 32. Phylogenetic analysis of CPs involved in the biosynthesis of natural products containing β-amino acids and its analogs, which indicated the convergent evolution of AfcK and AfcQ. Based on native substrates of the corresponding partner A domains (Supplementary Figure 28), the 14 CPs are categorized into four subgroups L-Glu (***green***), 3-MeAsp (***blue***), L-Asp (***orange***), and 3-methyl-β-Ala (***lilac***), whereas FlvL, being loaded with L-Asp, is an exception to this classification. It is evident that AfcK and BolC are closely related to BtrI. BtrI is known to participate in the biosynthesis of butirosin. (Accession numbers: BolC_WP_013690725.1,[42,43] FlvL_BAV56000.1,[52] BtrI_BAB18048.1,[44] AurL2_AWR88408.1,[58] IdnL6_BAP34717.1,[57] LobK_ALA09364.1,[55] MirF_WP_015801511.1,[55,56] BomD_QBL56185.1,[49] CirA7_UKD51470.1,[51] MteD_OAP25803.1,[50] SceH_ANH11403.1,[48] TriH_QGA70085.1,[47] VinL_BAD08369.1[45,46])

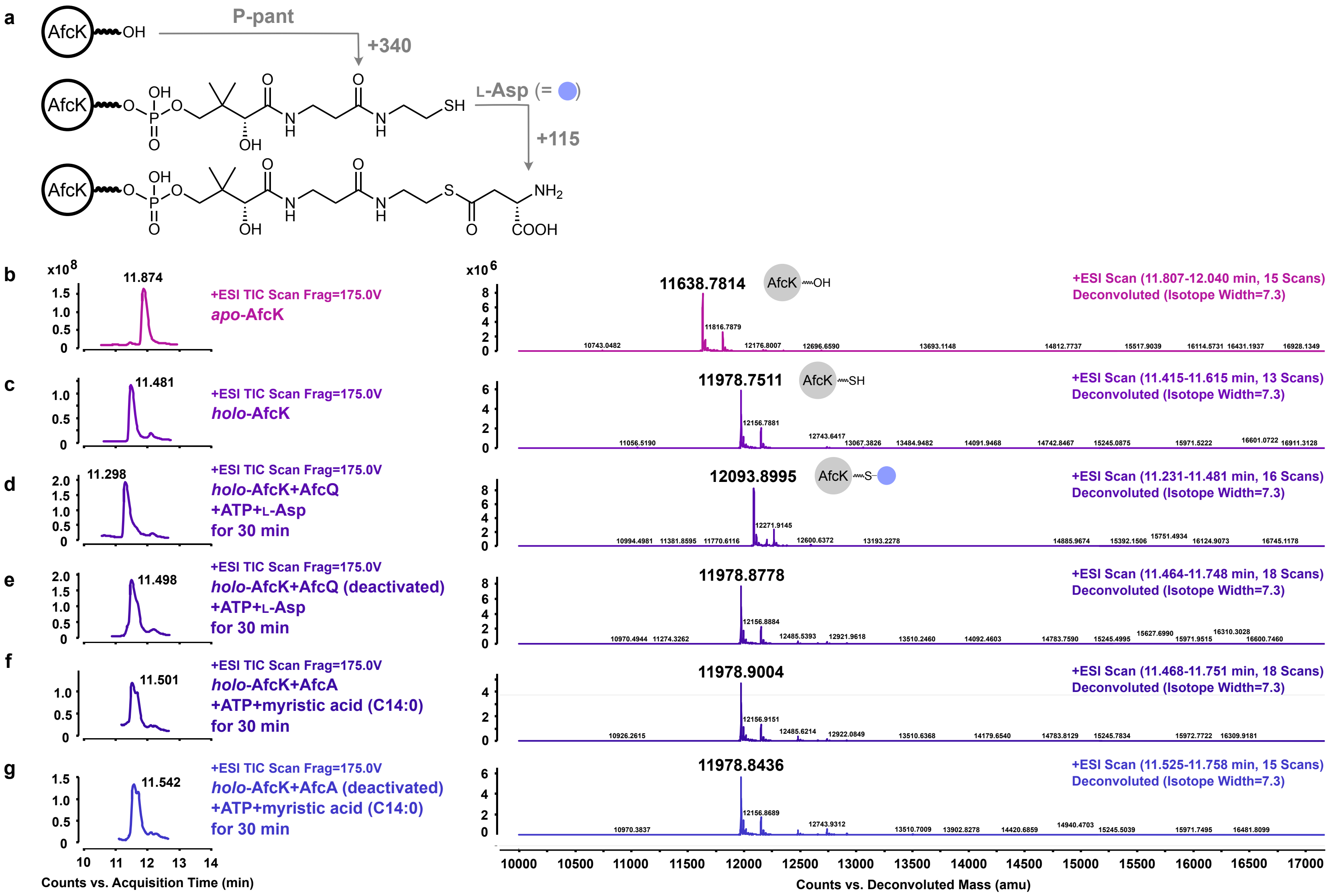

Supplementary Figure 33. Loading of *holo*-AfcK monitored by ESI (+)-Q-TOF. **a**) Reaction scheme of the conversion from *apo*-AfcK to *holo*-AfcK and the loading of L-Asp. Mass shift of 340 amu between *apo*- and *holo*-AfcK was annotated as the loading of ppant arm. The loading of an L-Asp to ppant arm was confirmed by the detection of the mass addition of 115 amu. ESI (+)-Q-TOF of AfcK loading, from **b**)-**g**): *apo*-AfcK, *holo*-AfcK, *holo*-AfcK/L-Asp/AfcQ, denatured AfcQ (95 °C for 5 min) as a negative control, *holo*-AfcK/myristic acid (C14:0)/AfcA, denatured AfcA (95 °C for 5 min) as a negative control.

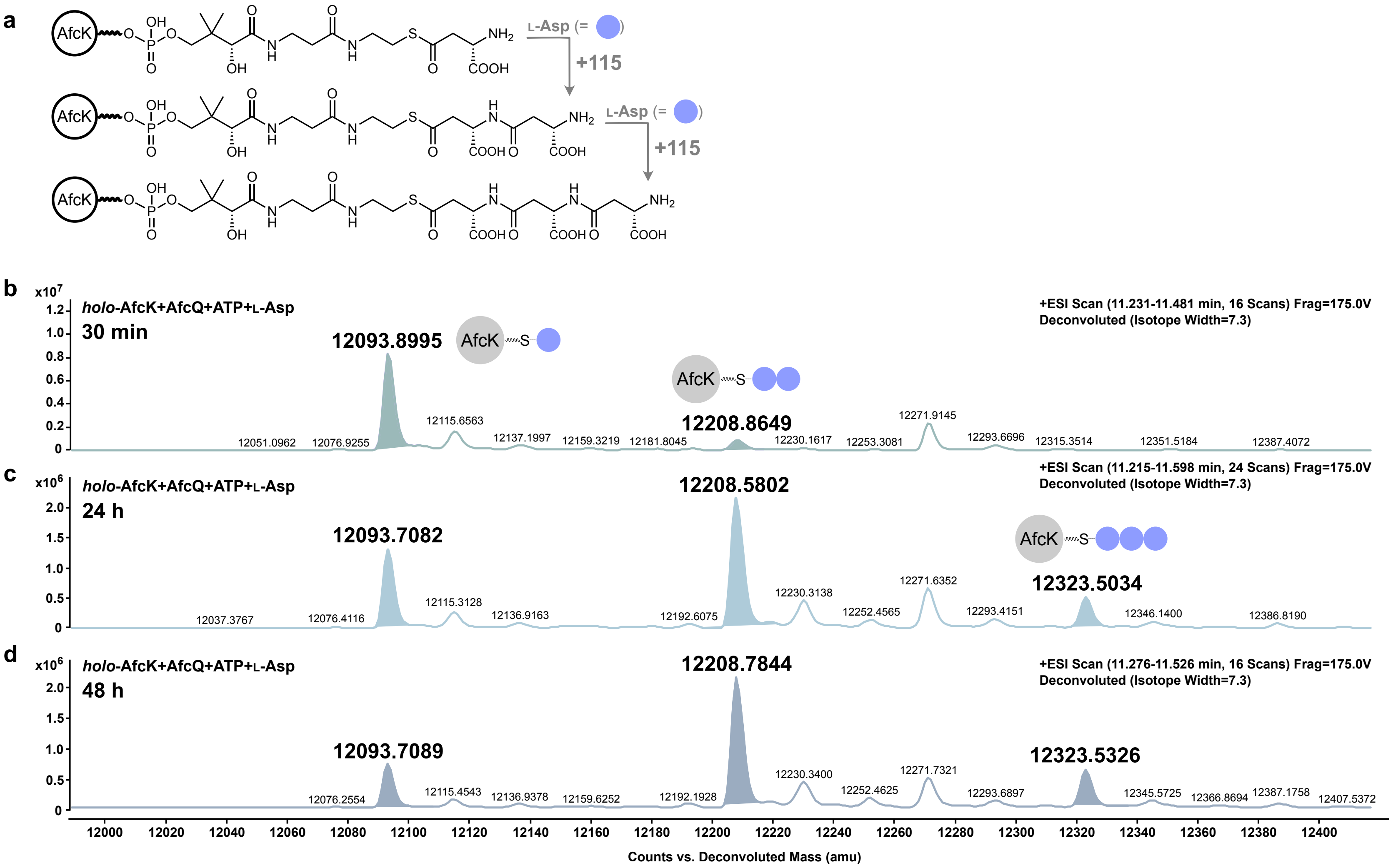

Supplementary Figure 34. Multiple L-Asp-loading of *holo*-AfcK with single AfcQ incubation monitored by ESI (+)-Q-TOF. **a**) Reaction scheme. **b**)-**d**): For longer incubation times (>6 h) the loading of the second and the third L-Asp to the ppant arm was confirmed by the detection of the mass difference of an additional 115 amu from 12,093 to 12,208 amu, and from 12,208 to 12,323 amu, respectively.

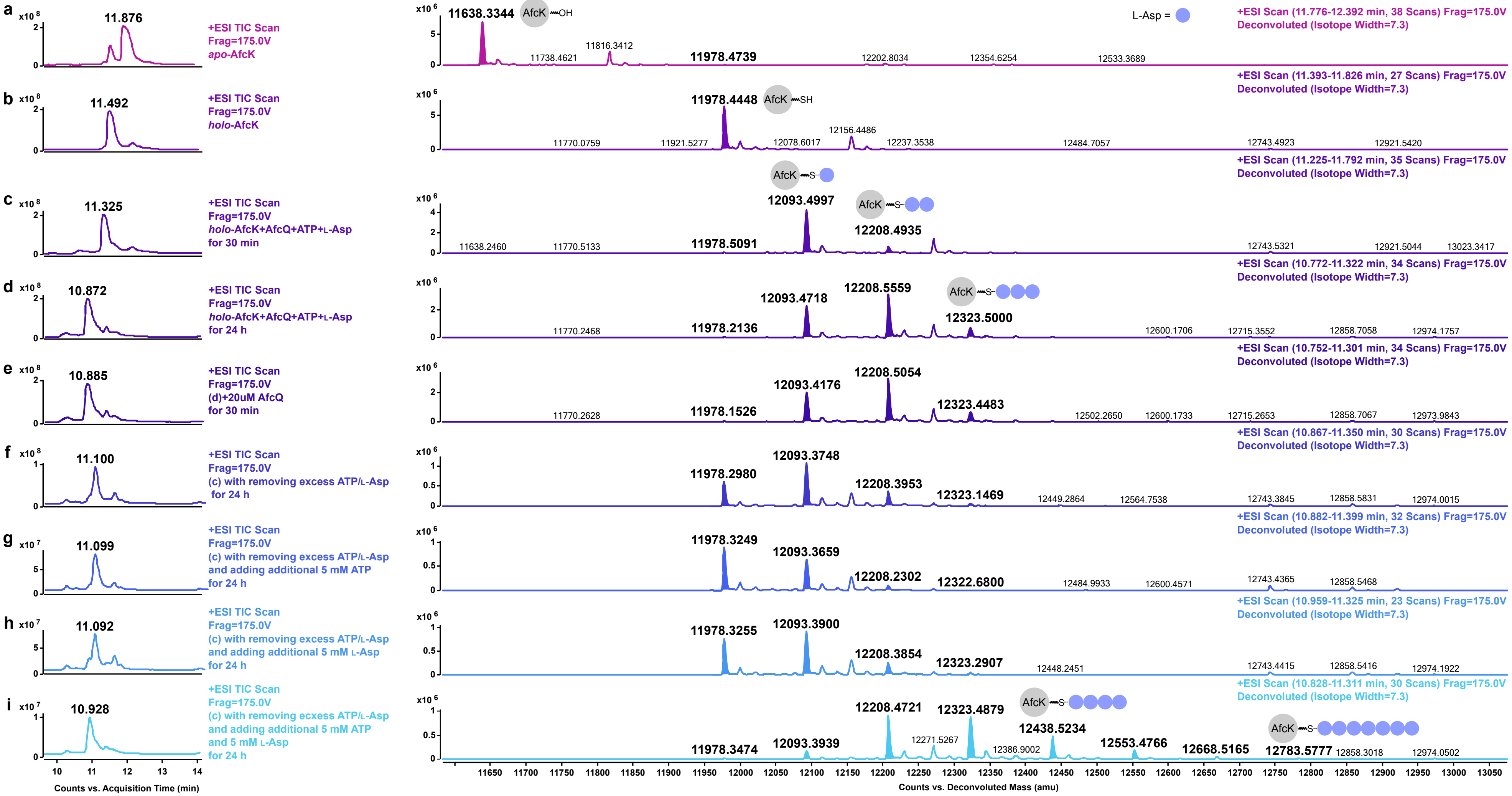

Supplementary Figure 35. Multiple L-Asp-loading of *holo*-AfcK with repeated AfcQ incubation monitored by ESI (+)-Q-TOF. **a**) *apo*-AfcK. **b**) *holo*-AfcK. **c**) The reaction system, containing *holo*-AfcK, AfcQ, ATP, and L-Asp, was incubated at 30 °C for 30 min. The loading of two L-Asp residues was detected. **d**) Detection of three L-Asp residues after incubation for 24 h. **e**) 20 μM AfcQ was added to d) after 24 h followed by incubation for an additional 30 min. The excess ATP and L-Asp were removed from c) followed by **f**) incubation for 24 h, **g**) adding 5 mM ATP and incubation for 24 h, **h**) adding 5 mM L-Asp and incubation for 24 h, **i**) adding 5 mM ATP/5 mM L-Asp and incubation for 24 h. The loading of seven L-Asp residues was detected.

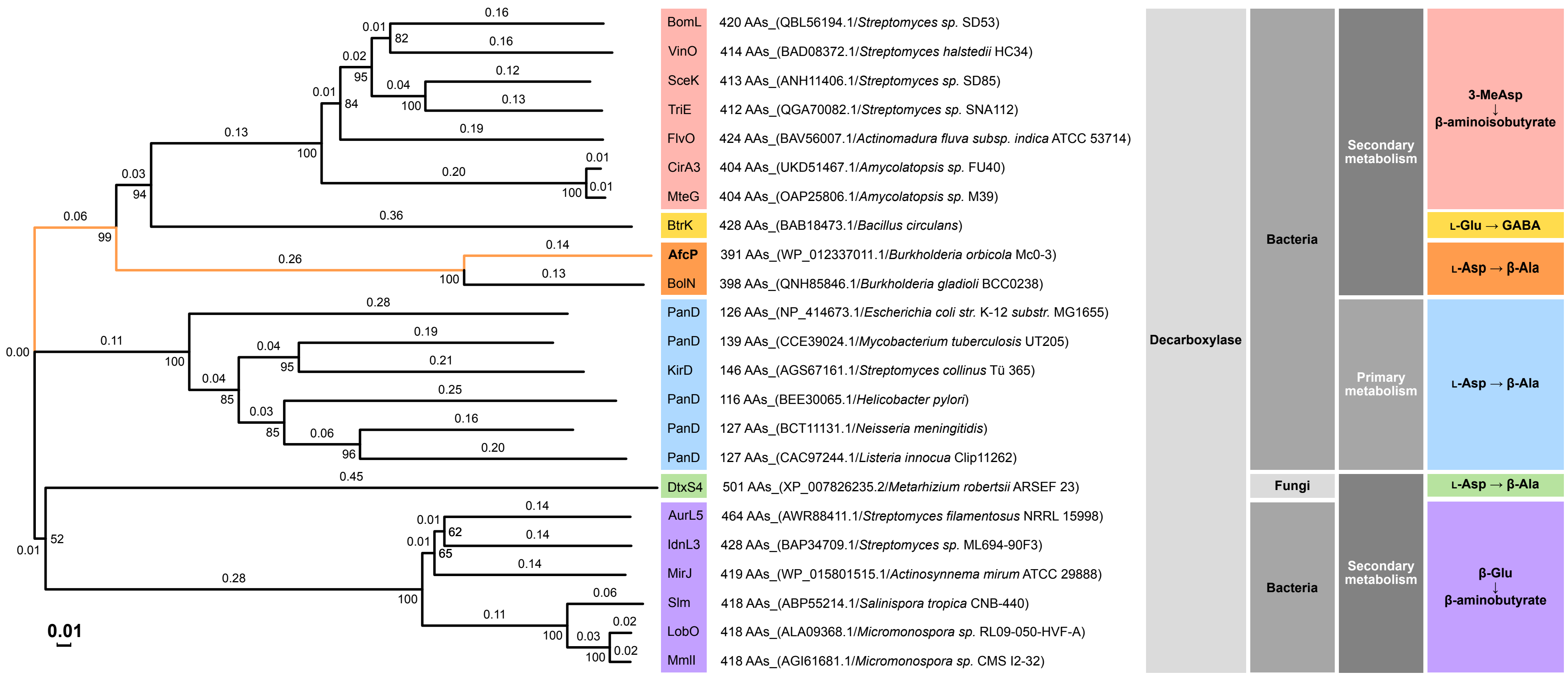

Supplementary Figure 36. Phylogenetic tree of decarboxylases involved in the biosynthesis of natural products containing β-amino acid or its analogs. Based on the origin (bacteria/fungi), metabolomic pathways (primary/secondary), and native substrates (3-MeAsp/L-Glu/L-Asp/β-Glu), the 23 decarboxylases were categorized into six subgroups, which are color-coded in blue, green, orange, pink, yellow, and lilac, respectively. However, KirD and FlvO are two exceptions to this classification. KirD is involved in the biosynthesis of a secondary metabolite known as kirromycin, whereas FlvO utilizes L-Asp as substrate. It is evident that AfcP and BolN are closely related to BtrK in terms of their phylogenetic relationship. Decarboxylases aligned here were extracted from BGCs involved in the biosynthesis of bombyxamycin,[49] vicenistatin,[45,46] sceliphrolactam,[48] tripartilactam,[47] fluvirucin B_2,[52] ciromicin,[51] macrotermycin,[50] butirosin,[44] bolagladin,[42,43] pantothenate,[75,76] kirromycin,[77] destruxin,[78] auroramycin,[58] incednine,[57] mirilactam,[55,56] salinilactam,[53] lobosamide[55], micromonolactam.[54]

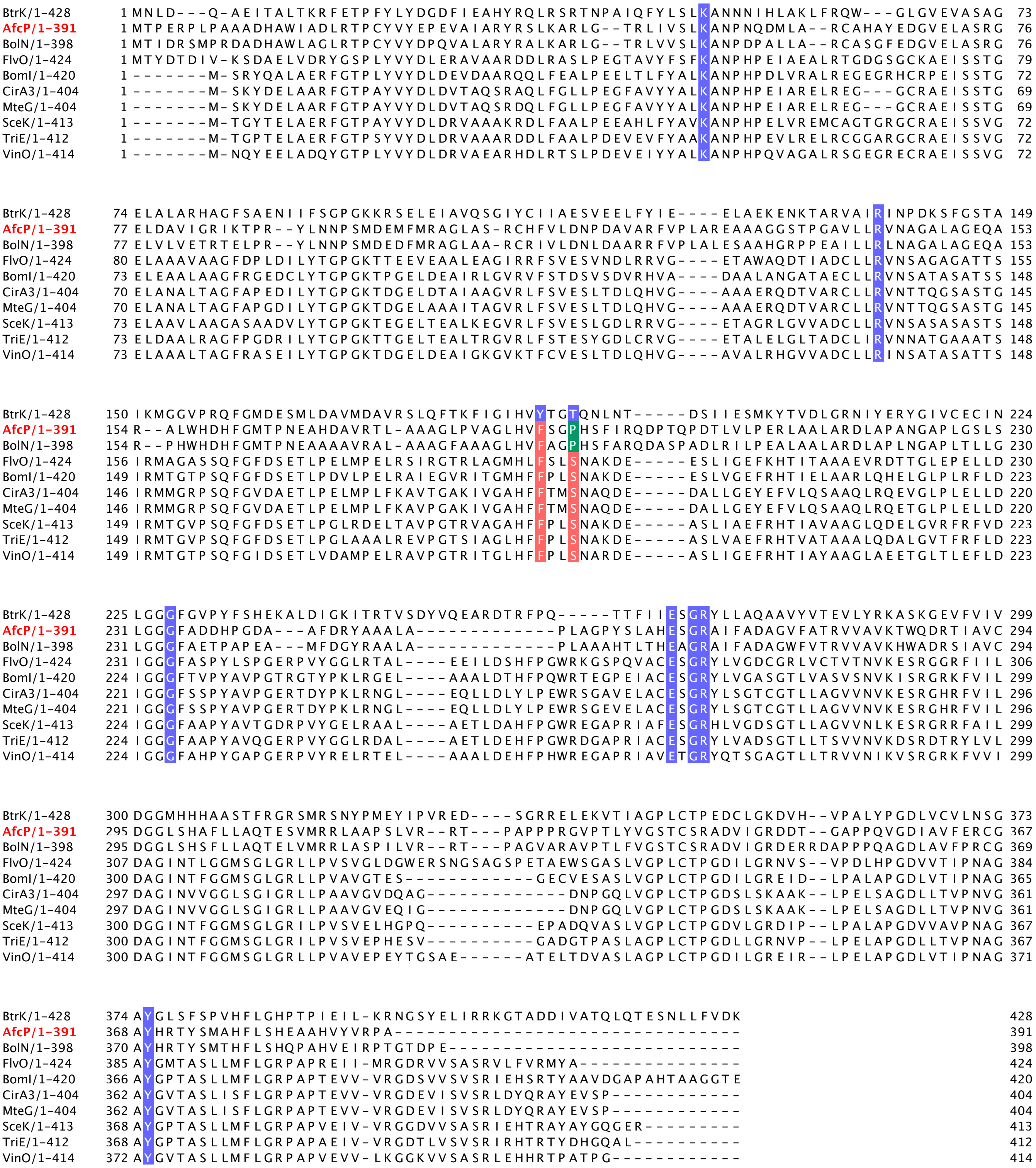

Supplementary Figure 37. Alignment of PLP-dependent decarboxylases. Biosynthesis of β-Ala and its derivatives (β-aminoisobutyrate and GABA) involves multiple PLP-dependent decarboxylases, and their alignment reveals conserved PLP binding sites (***blue***).[79] Notably, while BtrK is often used as the reference, other PLP-dependent decarboxylases feature a replacement of the classical Tyr187 with phenylalanine (***pink***). Despite this substitution, the π-π stacking interaction between the PLP pyridine ring and the phenyl side chain remains. Moreover, Thr190 in BtrK is substituted for proline in AfcP and BolN (***green***). Decarboxylases aligned here were extracted from BGCs involved in the biosynthesis of butirosin,[44] bolagladin,[42,43] fluvirucin B_2,[52] bombyxamycin,[49] ciromicin,[51] macrotermycin,[50] sceliphrolactam,[48] tripartilactam,[47] and vicenistatin.[45,46]

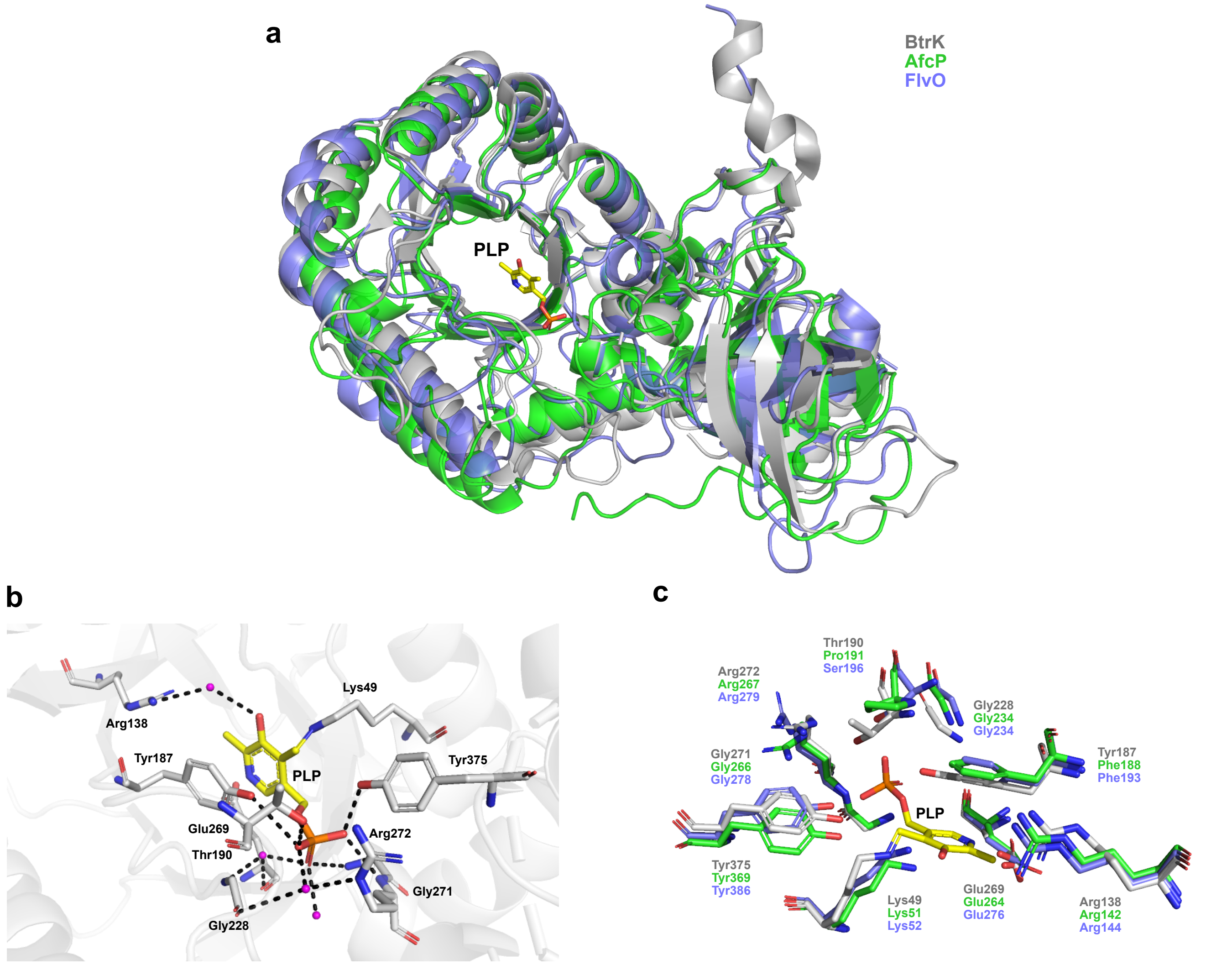

Supplementary Figure 38. Structural analysis of AfcP. **a**) Alignmnent of decarboxylases BtrK (PDB: 7RU7),[79] AfcP (AlphaFold2),[16–20] and FlvO (AlphaFold2).[16–20] The co-factor PLP is shown as stick model. **b**) The binding sites of PLP (***yellow***) in BtrK. **c**) Alignment of the binding pockets of PLP (***yellow***) in BtrK (***gray***), AfcP (***green***), and FlvO (***blue***) by superimposing the crystal structure of BtrK and the AlphaFold2-predicted structures. Lys51 of AfcP represents the expected site of PLP attachment as Schiff base.

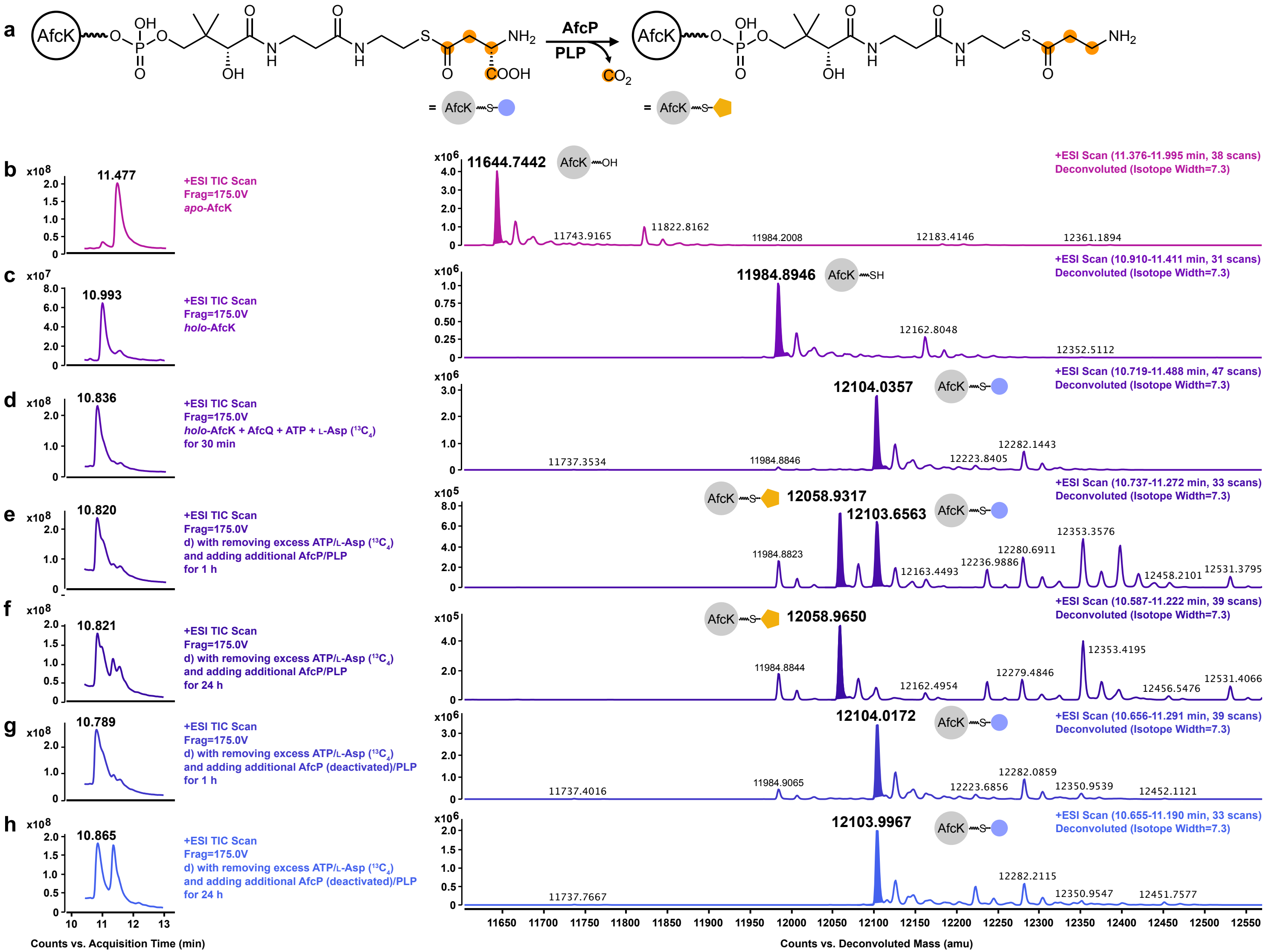

Supplementary Figure 39. Decarboxylation assay of mono-loaded H_2N-L-Asp-γ-*S*-AfcK monitored by ESI (+)-Q-TOF. **a**) Reaction scheme and L-Asp ($^{13}C_4$) as substrate (***orange***). **b**) *apo*-AfcK. **c**) *holo*-AfcK. **d**) mono-loaded H_2N-L-Asp-γ-*S*-AfcK. The excess ATP and L-Asp were removed from d) followed by adding AfcP/PLP and incubation at 30 °C for **e**) 1 hour or **f**) 24 hours. Denatured AfcP was used as negative controls in **g**) and **h**).

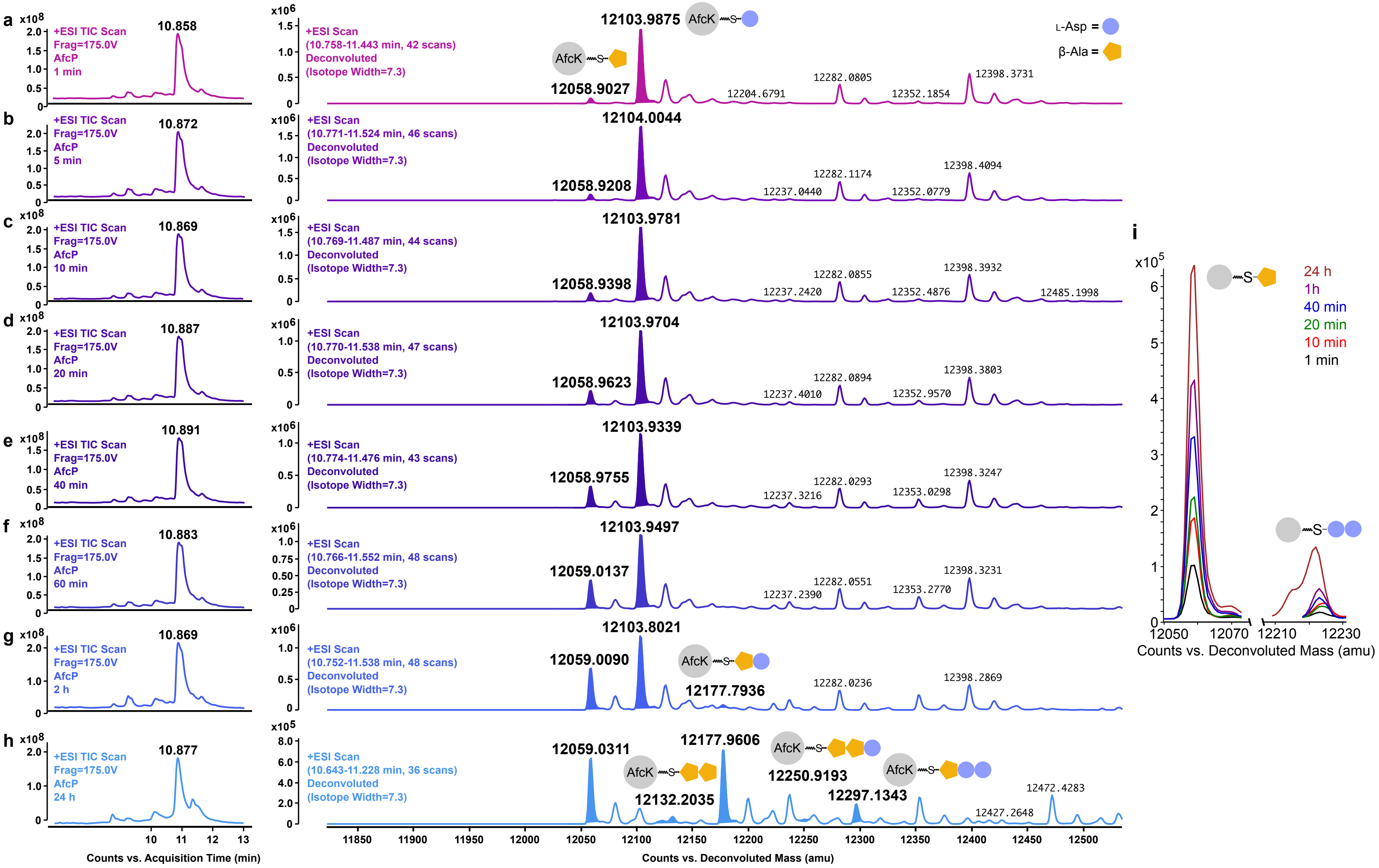

Supplementary Figure 40. One-pot AfcQ/AfcK/AfcP decarboxylation assay monitored by ESI (+)-Q-TOF. Reaction mixture containing *holo*-AfcK, AfcQ, ATP, L-Asp ($^{13}C_4$), AfcP, and PLP was incubated at 30 °C for **a**) 1 min, **b**) 5 min, **c**) 10 min, **d**) 20 min, **e**) 40 min, **f**) 60 min, **g**) 2 hours, and **h**) 24 hours. **i**) Alignment of peaks corresponding to H_2N-β-Ala-*S*-AfcK and H_2N-(L-Asp)$_2$-*S*-AfcK for continuous monitoring from 1 min to 24 hours.

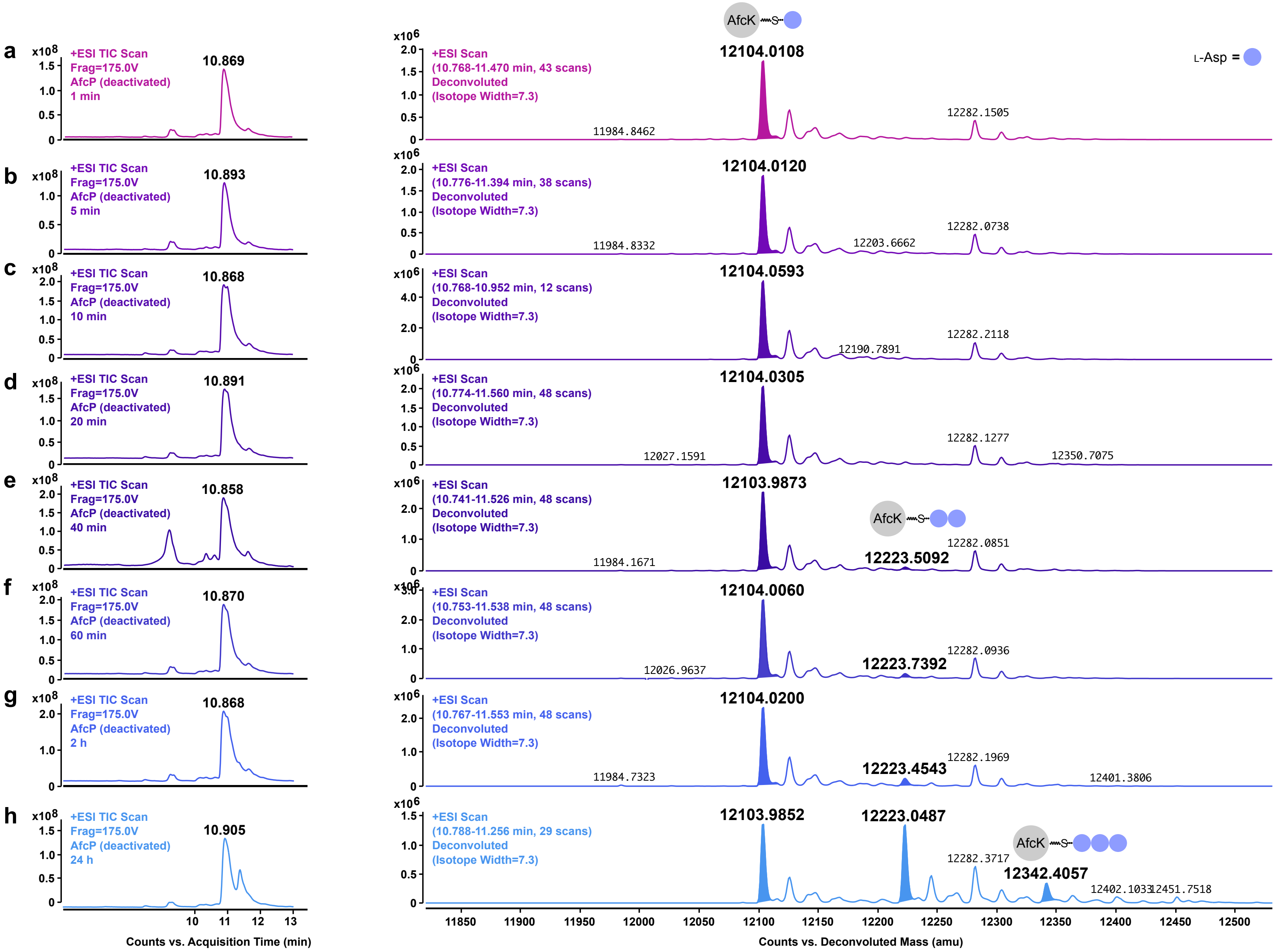

Supplementary Figure 41. Control one-pot reaction utilizing denatured AfcP. Reaction mixture containing *holo*-AfcK, AfcQ, ATP, L-Asp ($^{13}C_4$), denatured AfcP, and PLP was incubated at 30 °C for **a**) 1 min, **b**) 5 min, **c**) 10 min, **d**) 20 min, **e**) 40 min, **f**) 60 min, **g**) 2 hours, and **h**) 24 hours.

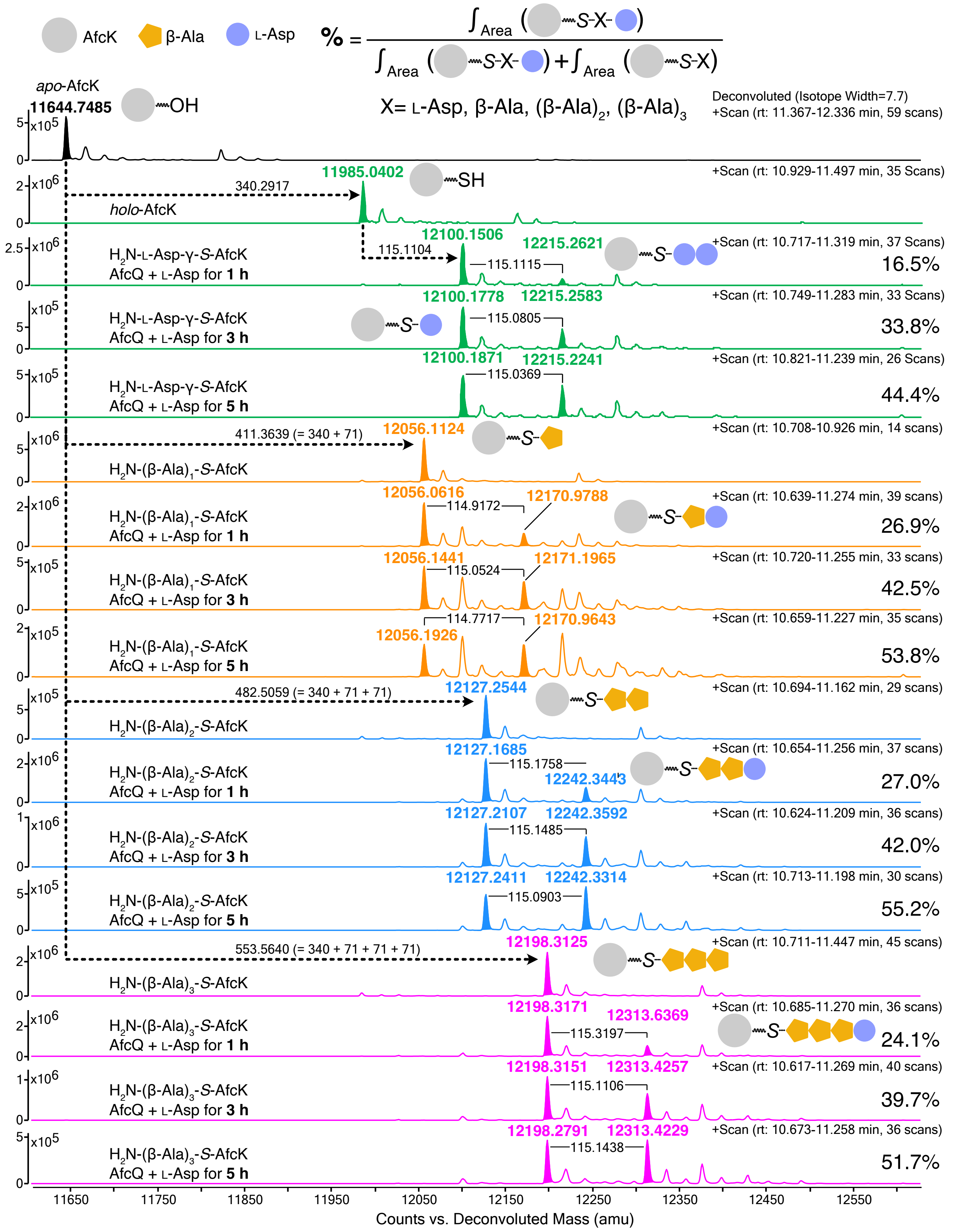

Supplementary Figure 42. Comparison of the loading efficiency of L-Asp onto H_2N-L-Asp-γ-*S*-AfcK and H_2N-(β-Ala)$_n$-*S*-AfcK (n=1,2, and 3) as acceptors. The calculated percentage is used to reflect the loading efficiency of the subsequent L-Asp.

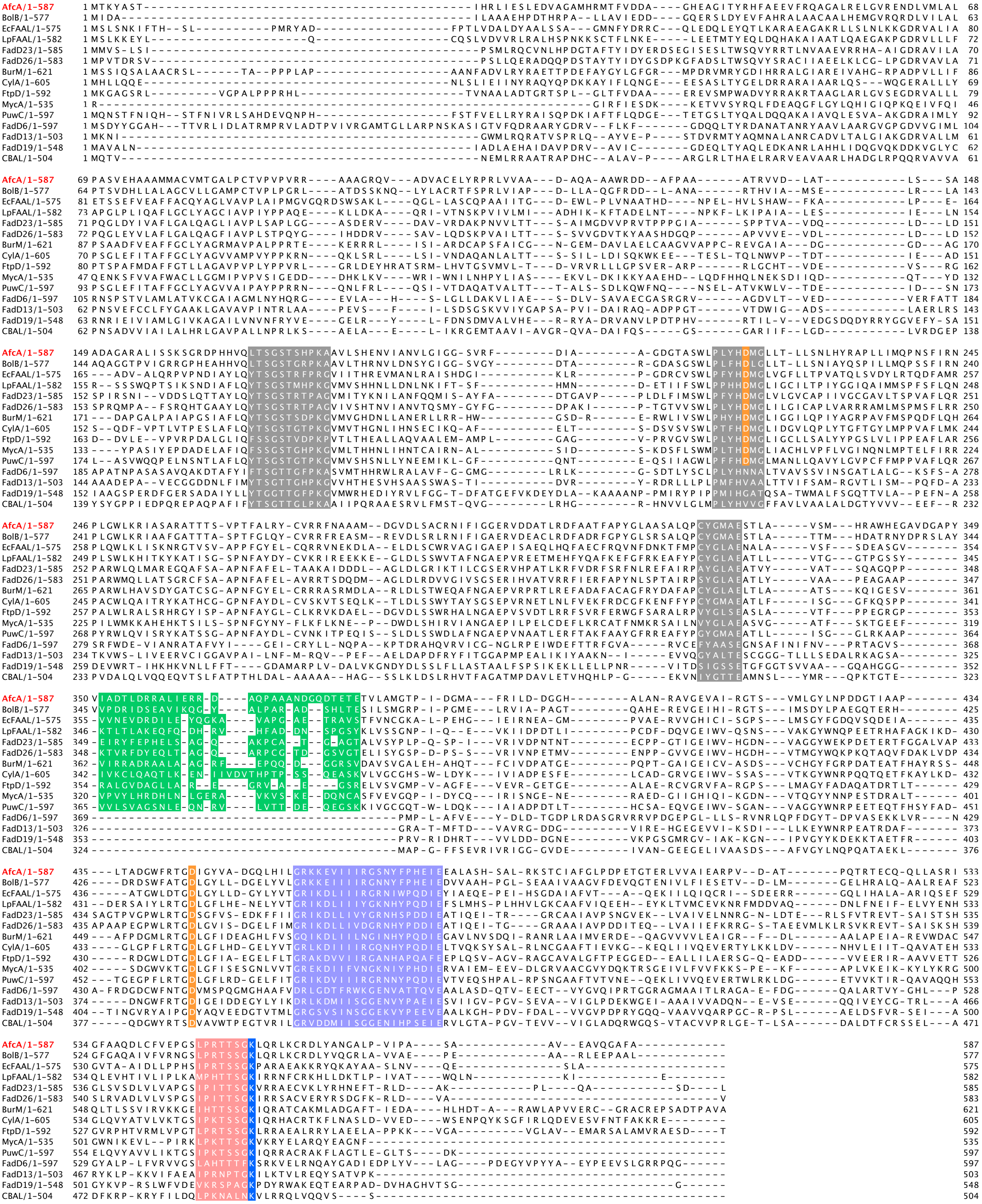

Supplementary Figure 43. Alignment of AfcA with selected FAALs and FACLs revealed the conserved gate motifs (***gray***), insertion motif (***green***), hinge region (***light blue***), and C-terminal loop (***pink***). The conserved Asp and Lys residues are highlighted in orange and blue, respectively. The FAALs aligned here are BolB (WP_241238501.1),[42,43] EcFAAL (PDB: 3PBK),[80] LpFAAL (PDB: 3KXW),[80] FadD23 (PDB: 8IQU),[81] FadD26 (CCP45733.1),[82] BurM (WP_004188299.1),[33] CylA (AFV96135.1),[83] FtpD (WP_011556561.1),[84] MycA (WP_003238152.1),[85] and PuwC (AIW82280.1).[86] The FACLs selected are FadD6 (NP_215722.1), FadD13 (NP_217605.1), FadD19 (YP_177983.1), and CBAL (PDB: 1T5D).[87,88]

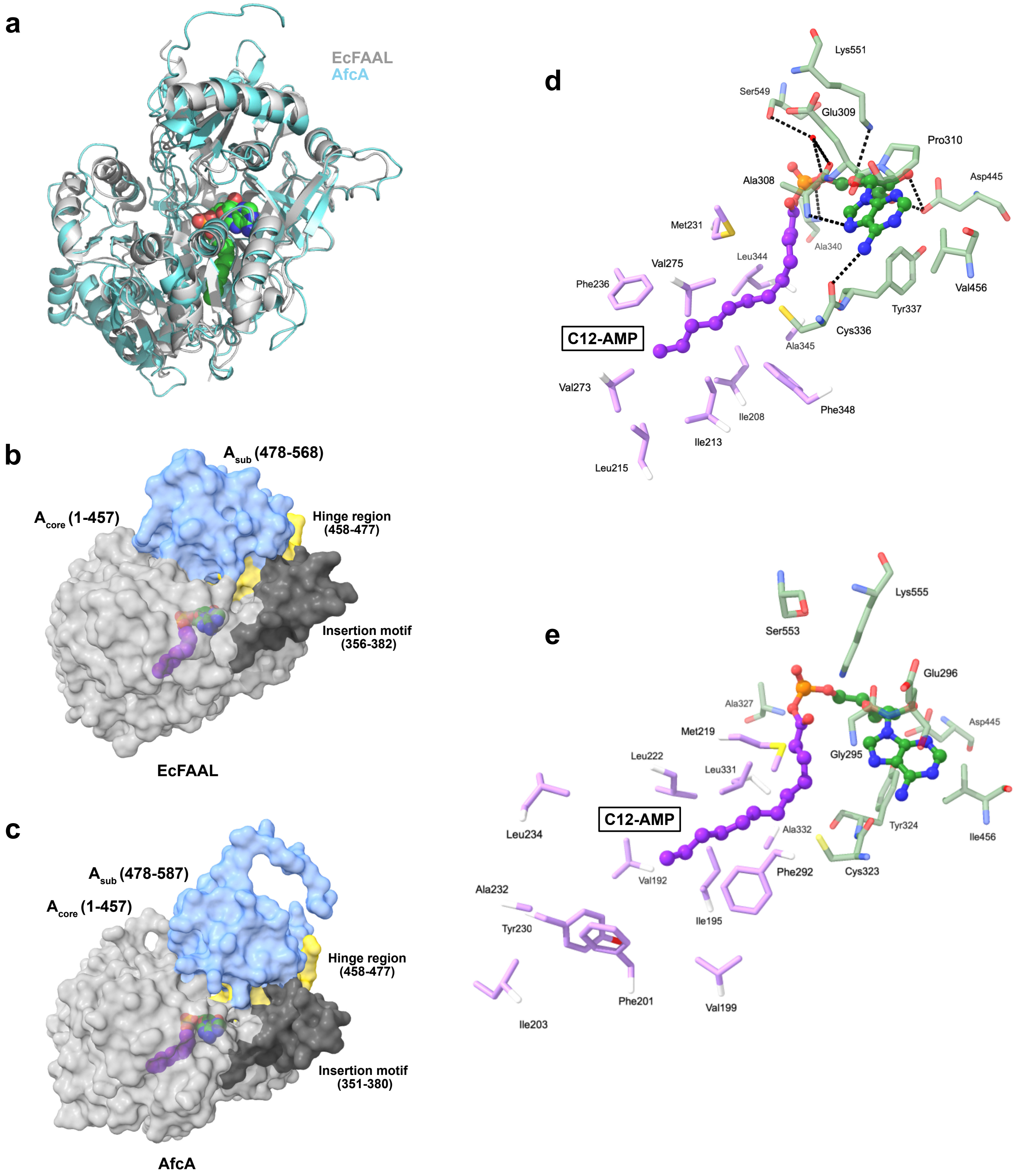

Supplementary Figure 44. Structural analysis of AfcA. **a**) Alignment of AlphaFold2-predicted AfcA[16–20] and EcFAAL (PDB: 3PBK) combined with dodecanoyl-AMP intermediate (***sphere***).[80] **b**) **c**) the crystal structure of EcFAAL and the predicted structure of AfcA, respectively. The N-terminal A_{core}, C-terminal A_{sub}, the hinge region, and the insertion motif are highlighted in gray, blue, yellow, and dark gray, respectively. **d**) The binding sites of dodecanoyl-AMP (***ball stick***) in EcFAAL. The residues (***stick***, ***pink***) indicate the critical sites involved in the formation of a hydrophobic pocket for the acyl chain. Meanwhile, the residues (***stick***, ***green***) indicate the AMP binding sites. **e**) The predicted binding pocket of adenylate intermediate in AfcA by superimposing the crystal structure of EcFAAL and the predicted structure of AfcA.

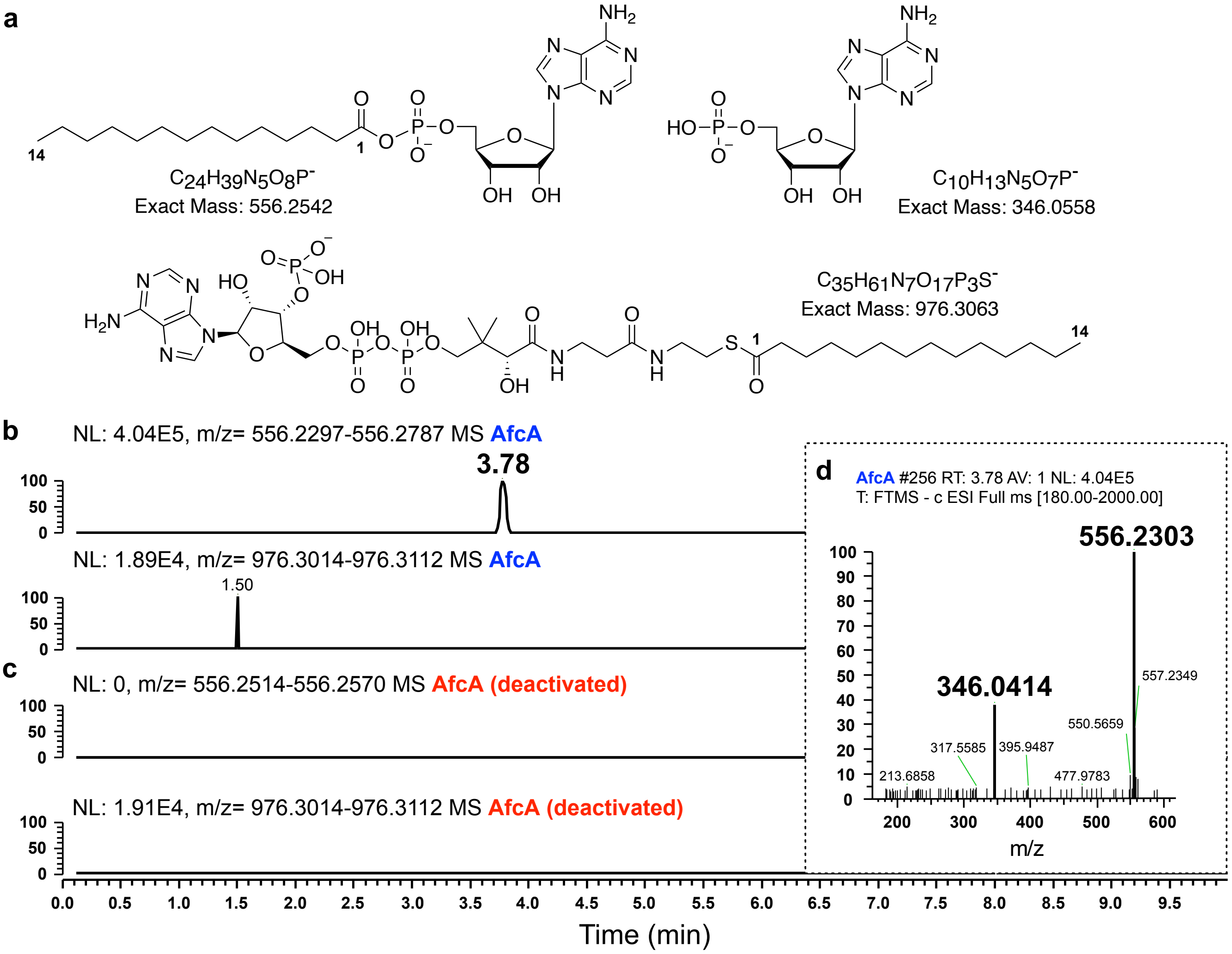

Supplementary Figure 45. AfcA-catalyzed activation of myristic acid (C14:0) monitored with LTQ-Orbitrap. **a**) The structures, molecular formulas, and theoretical molecular masses of [M-H]$^-$ ions of expected reaction products: CH_3-$(CH_2)_{12}$-CO-AMP, AMP, and CH_3-$(CH_2)_{12}$-CO-CoA. **b**) EIC of CH_3-$(CH_2)_{12}$-CO-AMP and CH_3-$(CH_2)_{12}$-CO-CoA from reaction system containing active AfcA. Adenylated myristic acid (C14:0) with retention time at 3.78 min was observed. **c**) Negative control using denatured AfcA. **d**) ESI (-)-MS spectrum of adenylated myristic acid (C14:0) acquired from b). The calculated mass accuracy for the theoretical [M-H]$^-$ at *m/z* 556.2542 and *m/z* 346.0558 was -43.0 and -41.6 ppm, respectively, which falls within the 50 ppm mass accuracy threshold of the Orbitrap instrument in negative ion mode, as determined by daily measurements using a reference compound.

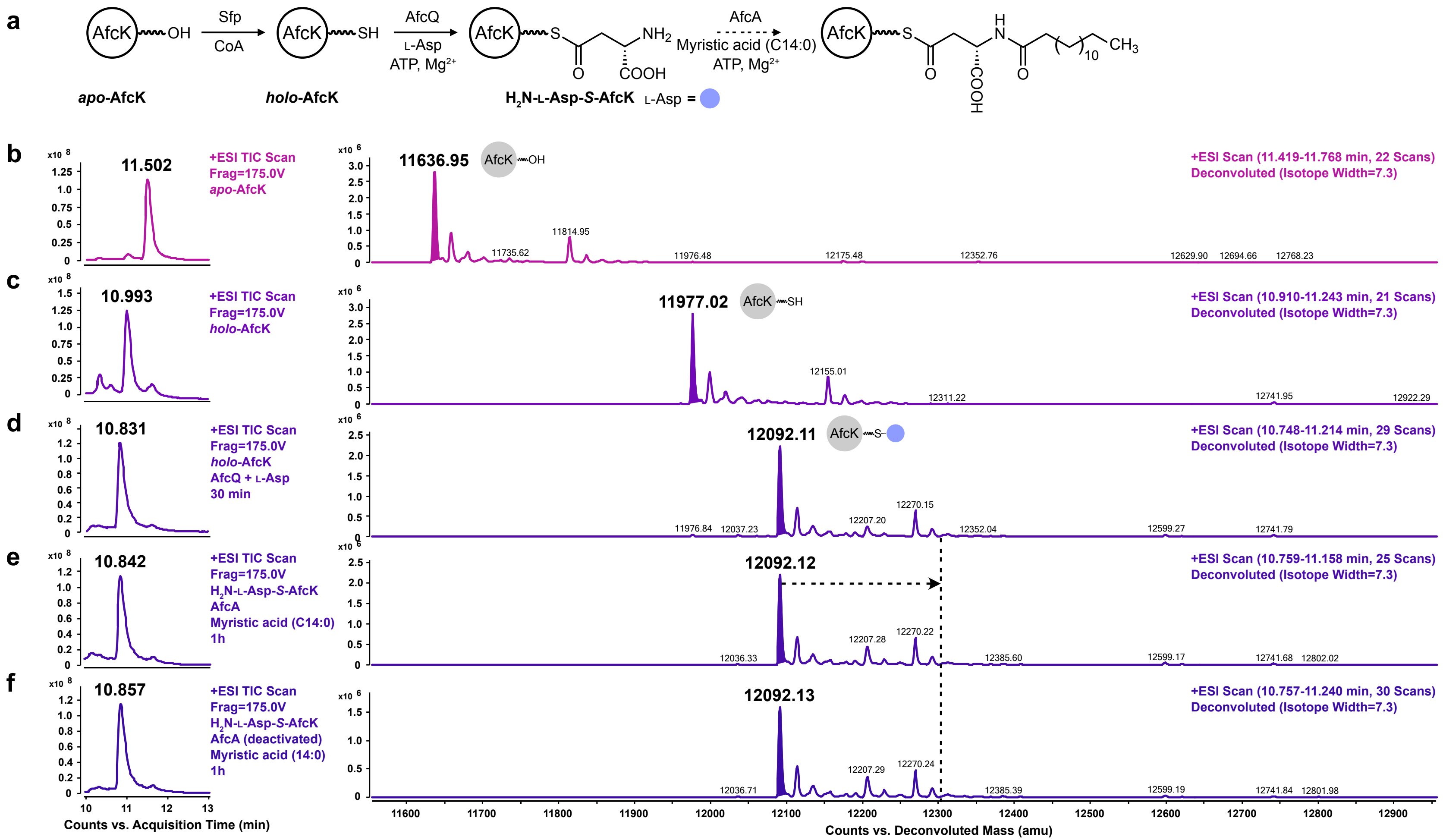

Supplementary Figure 46. AfcA-catalyzed acyl transfer using myristic acid (C14:0) as substrate and H_2N-L-Asp-γ-*S*-AfcK as acceptor. **a**) Reaction scheme. ESI (+)-Q-TOF of AfcK loading subsequent reactions (from **b** to **f:** *apo*-AfcK, *holo*-AfcK, L-Asp loaded *holo*-AfcK, reaction with AfcA, denatured AfcA as a negative control). The expected mass of the product myristic-L-Asp-γ-*S*-AfcK (***dashed line***) was not observed.

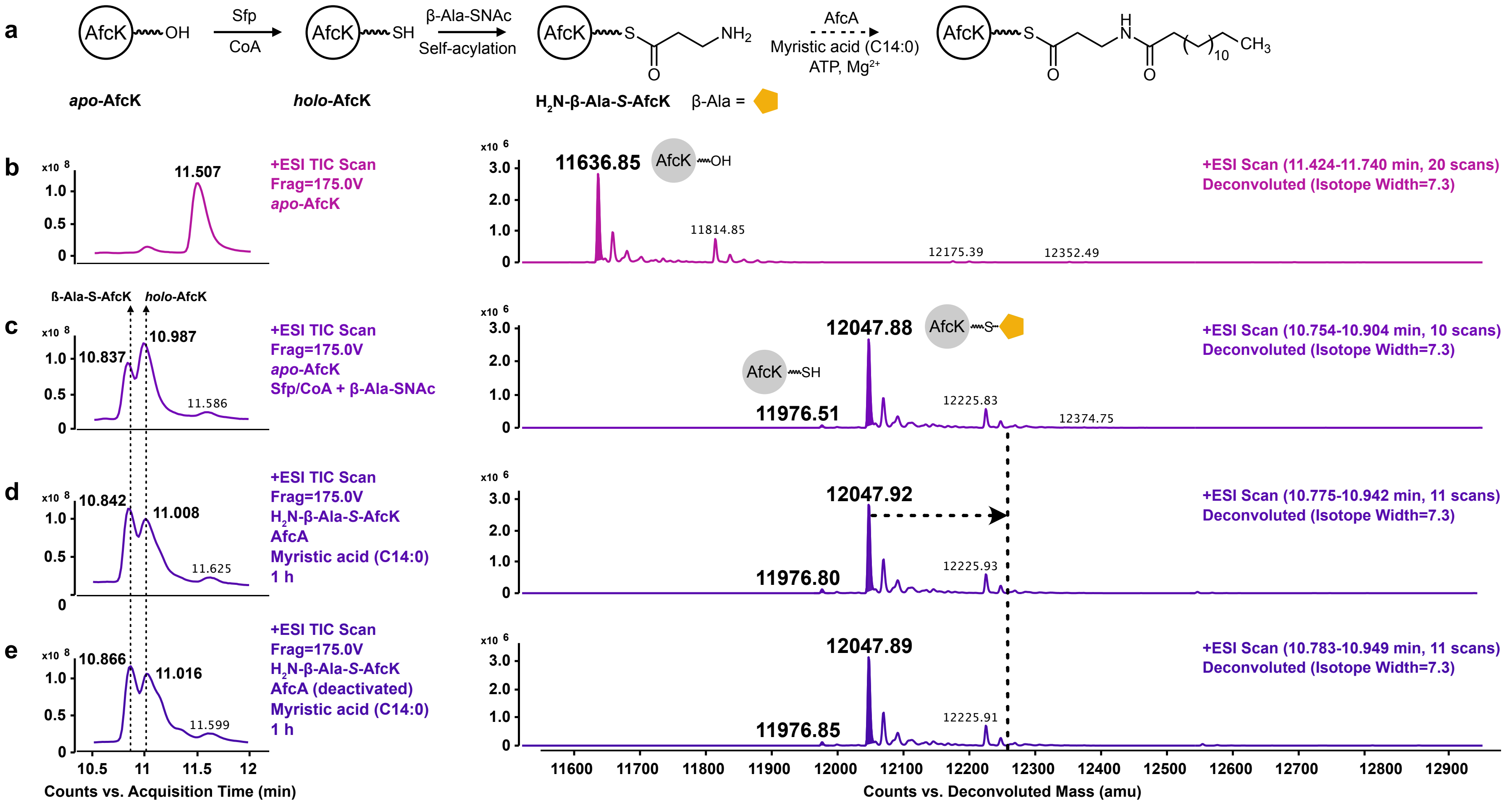

Supplementary Figure 47. AfcA-catalyzed acyl transfer using myristic acid (C14:0) as substrate and H_2N-β-Ala-*S*-AfcK as acceptor. **a**) Reaction scheme. ESI (+)-Q-TOF of AfcK loading subsequent reactions (from **b** to **e:** *apo*-AfcK, β-Ala loaded AfcK, reaction with AfcA, denatured AfcA as a negative control). The expected mass of the product H_2N-β-Ala-*S*-AfcK (***dashed line***) was not observed.

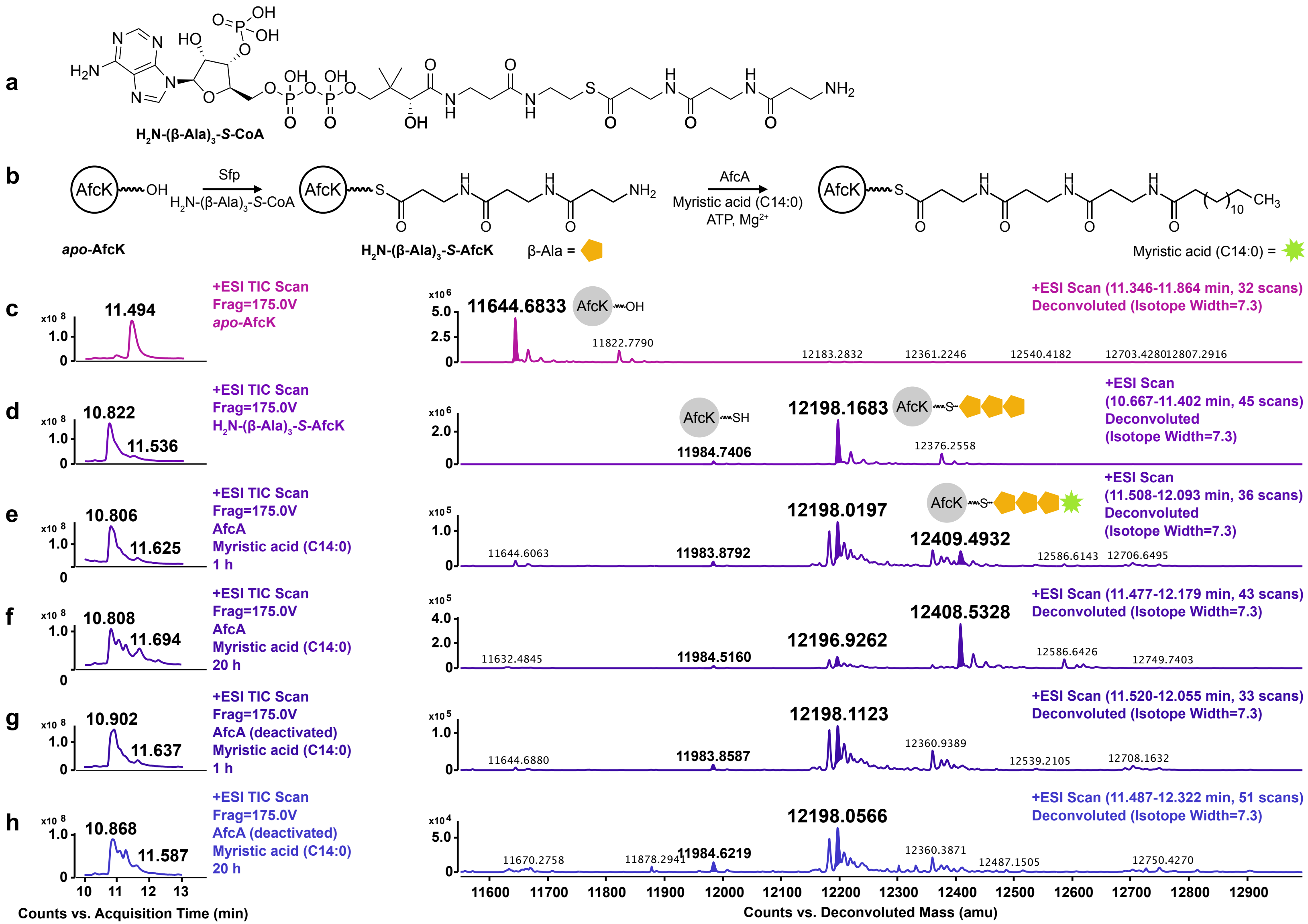

Supplementary Figure 48. AfcA-catalyzed acyl transfer using myristic acid (C14:0) as substrate and H_2N-(β-Ala)$_3$-*S*-AfcK as acceptor. **a**) Structure of H_2N-(β-Ala)$_3$-*S*-CoA. **b**) Reaction scheme. ESI (+)-Q-TOF of AfcK loading (from **c** to **f**: *apo*-AfcK, H_2N-(β-Ala)$_3$-*S*-AfcK, reaction with AfcA for 1 hour, reaction with AfcA for 20 hours). Denatured AfcA was used as negative controls in **g**) and **h**). The loading of myristic acid (C14:0) was observed.

Supplementary Table 9. Selected proteins involved in C-C, C-N, C-S, and C-O bond formation.

Bond	Protein	Nature product	PDB	Function	Donor	Intermediate	Acceptor	Product	Catalytic triad		
C-C	FabB[89]	Fatty acid	1DD8	β-ketoacyl-ACP synthase I (KAS I)	ACP_x-*S*-X	FabB-*S*-X	ACP_Y-*S*-Y	ACP_Y-*S*-Y-X	C^{163}	H^{298}	H^{333}
	FabF[90]	Fatty acid	2GFW	β-ketoacyl-ACP synthase II (KAS II)	ACP_x-*S*-X	FabF-*S*-X	ACP_Y-*S*-Y	ACP_Y-*S*-Y-X	C^{163}	H^{303}	H^{340}
	FabF[91]	Fatty acid	4JPF	β-ketoacyl-ACP synthase II (KAS II)	ACP_x-*S*-X	FabF-*S*-X	ACP_Y-*S*-Y	ACP_Y-*S*-Y-X	C^{164}	H^{304}	H^{341}
	FabH[92]	Fatty acid	1U6S	β-ketoacyl-ACP synthase III (KAS III)	CoA-*S*-X	FabH-*S*-X	ACP_Y-*S*-Y	ACP_Y-*S*-Y-X	C^{112}	H^{244}	H^{274}
	ZhuH[93]	Fatty acid	1MZJ	β-ketoacyl-ACP synthase III (KAS III)	CoA-*S*-X	ZhuH-*S*-X	ACP_Y-*S*-Y	ACP_Y-*S*-Y-X	C^{121}	H^{257}	N^{288}
	DpsC[94]	Daunorubicin	5WGC	KAS III-like	CoA-*S*-X	DpsC-*S*-X	ACP_Y-*S*-Y	ACP_Y-*S*-Y-X	S^{118}	H^{198}	H^{297}
C-N	*holo*-AB3403[95]	Tyrocidine	4ZXI	Condensation domain in NRPS	PCP_x-*S*-X	/	PCP_Y-*S*-Y	PCP_Y-*S*-Y-X	H^{144}	H^{145}	D^{149}
	CtaG[41]	Closthioamide	/	Thiotemplated acyltransferase	PCP_x-*S*-X	CtaG-*S*-X	PCP_Y-*S*-Y	PCP_Y-*S*-Y-X	C^{11}	H^{128}	D^{144}
	AdmF[96]	Andrimid	/	Thiotemplated acyltransferase	ACP_x-*S*-X	AdmF-*S*-X	PCP_Y-*S*-Y	PCP_Y-*S*-Y-X	C^{90}	H^{126}	D^{143}
	BtrH[97]	Butirosin	/	ACP-aminoglycoside acyltransferase (product release)	ACP_x-*S*-X	BtrH-*S*-X	Y-NH_2	Y-X	C^{13}	H^{122}	D^{140}
	PamI[71]	Paenilamicin	/	BtrH-like (product release)	PCP_x-*S*-X	PamI-*S*-X	Y-NH_2	Y-X	C^{14}	H^{122}	D^{142}
	PnaB[98]	Phosphonoalamide	/	ATP-grasp ligase	X-COOH	X-P	Y-NH_2	Y-X	/	/	/
	PnaC[98]	Phosphonoalamide	/	ATP-grasp ligase	X-COOH	X-P	Y-NH_2	Y-X	/	/	/
	AutR[99]	Autucedine	/	ATP-grasp ligase	X-COOH	X-P	PCP-SH	PCP-*S*-X	/	/	/
C-S	ChlB3[100,101]	Chlorothricin	7EQI	KAS III-like	ACP_1-*S*-X	ChlB3-*S*-X	ACP_2	ACP_2-*S*-X	C^{113}	H^{296}	D^{301}
	CloN2[102]	Clorobiocin	/	Acyltransferase	ACP_1-*S*-X	CloN2-*S*-X	ACP_2	ACP_2-*S*-X	C^{113}	H^{296}	D^{301}
	CmaE[103]	Coronamic acid	/	Acyltransferase	ACP_1-*S*-X	CmaE-*S*-X	ACP_2	ACP_2-*S*-X	C^{105}	H^{255}	D^{260}
	SfaN[104]	Sanglifehrin	/	KAS III-like	ACP_1-*S*-X	SfaN-*S*-X	ACP_2	ACP_2-*S*-X	C^{114}	H^{297}	D^{302}
C-O	CerJ[105]	Cervimycin	3T6S	KAS III-like/O-malonyl transferase (product release)	CoA-*S*-X	CerJ-*S*-X	Y-OH	Y-X	C^{116}	H^{295}	D^{300}
	ChlB6[101]	Chlorothricin	/	KAS III-like (product release)	ACP-*S*-X	ChlB6-*S*-X	Y-OH	Y-X	C^{113}	H^{296}	D^{301}
	CloN7[102]	Clorobiocin	/	KAS III-like (product release)	ACP-*S*-X	CloN7-*S*-X	Y-OH	Y-X	S^{101}	/	D^{192}
This study	AfcL	AFC-BC11	/	KAS III-like	/	/	/	/	C^{111}	H^{238}	D^{243}
	AfcO	AFC-BC11	/	KAS III-like	/	/	/	/	C^{128}	H^{279}	D^{284}
	AfcR	AFC-BC11	/	KAS III-like	/	/	/	/	T^{157}	H^{307}	E^{312}

a

```
        AfcL   110  --ECAGWGYVLRRALATAAET---        ---AALPFF-----PATSRRALLASVGKT---PLLPDGHGVFGHAFGSDPWLSVL  249
        AfcO   126  -LNCVSVAAGIETVRALMRRHP--        --LVLPHNA-----DLPGWNALCRAMRVPAE-RLYADNIHARGHACCSDFPINLA  290
        AfcR   155  -NGTLGWYSALMLLDGLLLDE---        --WCVPPGF-----DPGFAARVADAASIP---VAARIQHDGSGHLSSAESAAALI  318
      | 7EQI   112  --GCNGMFSALELAAAHLRAGP--        --RVAYMNF----SREIVEQRCMAALGLPMS-ASTWEFGRKLGHLGASDQVVALD  307
 C–S  | CloN2  112  --GCNGTFSALELAVPYLRSAP--        --TVVIHSM-----PRQAAASYLKILGFSLE-SSTWEFSRTTGHLGAGDHLAALH  307
      | SfaN   113  --GCASAVTAFQQIAGIFATRP--        --RFLFLND-----NQDSLADVAKAVGVPLD-RTNAELAKDLGHCGGADQLICLD  308
      | 3T6S   115  --RCNGGMGAIELAGAYLGSGIG-        --HAVIPVS-----RRGTGHELHDLLGLPDE-RTSWAYGRTTGHVGAGDQYAGLA  306
 C–O  | ChlB6  112  --GCNGMLAALEMAAGWLTLRG--        --KVLFAHVG----GAQTDAIVMQQLGLPLA-KSTWDHGRSIGHVGASDHVYDLE  307
      | CloN7  101  ---SGGAVTALVLAAQHPE-----        ----LHRPA----QQPPEPNSFTDPED-------VWTIL----------DRLFHHT  198
      | 1DD8   162  --ACATSAHCIGNAVEQIQLG---   294  --YLNSHGTSTPVGDVKELAAIREVFGDKS--PAISATKAMTGHSLGAAGVQEAI  344
 C–C  | 2GFW   162  --ACTSGVHNIGHAARIIAYG---   299  --YVNAHGTSTPAGDKAEAQAVKTIFGEAASRVLVSSTKSMTGHLLGAAGAVESI  351
      | 4JPF   163  --ACTTGTHSIGMAARNIAYG---   300  --YINAHGTSTPAGDIAEIAAVKSVFGEHAHALSMSSTKSMTGHLLGAAGAVEAI  352
      | 1MZJ   120  --ACAGFCHALSIAADAVESG---   253  --AFVPHQA-----NLRIIDVLVDRLGVPEH-VVVSRDAEDTGNTSSASVALALD  299
```

b

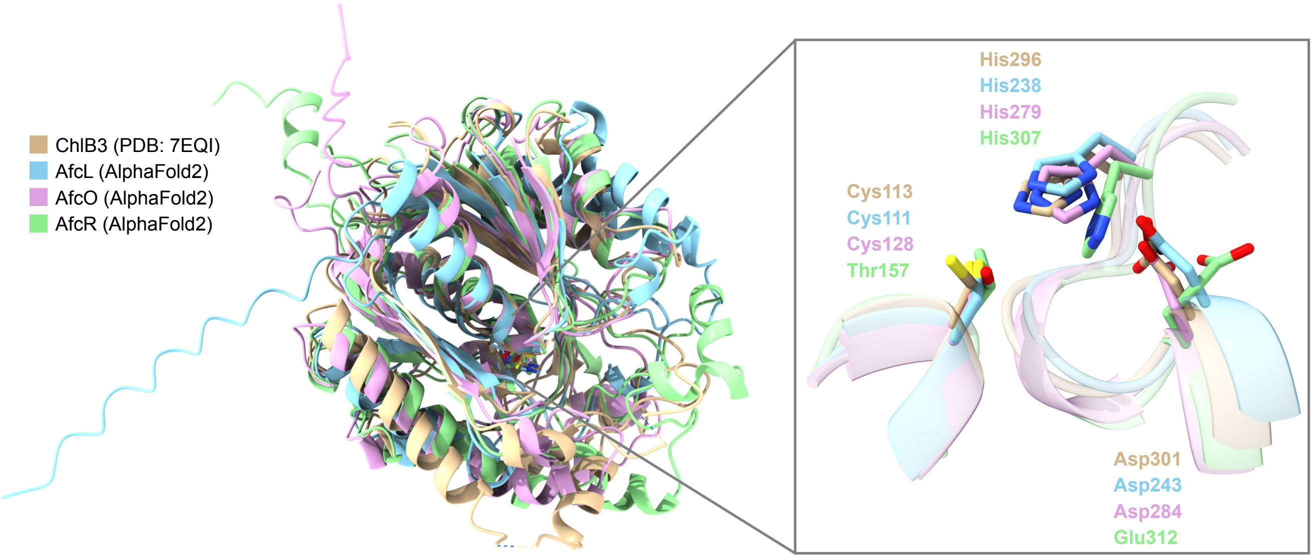

Supplementary Figure 49. Structural analysis of AfcL/O/R models. **a**) Multiple sequence alignment (MSA) of AfcL, AfcO and AfcR with selected acyltransferases from Supplementary Table 9. The conserved catalytic triads are highlighted in violet. **b**) 3D structure superposition of ChlB3 (***tan***, PDB:7EQI)[100,101] and AlphaFold2-predicted structures of AfcL (***sky blue***), AfcO (***lilac***) and AfcR (***green***).[16–20] The catalytic triads are shown in the zoomed view on the right.

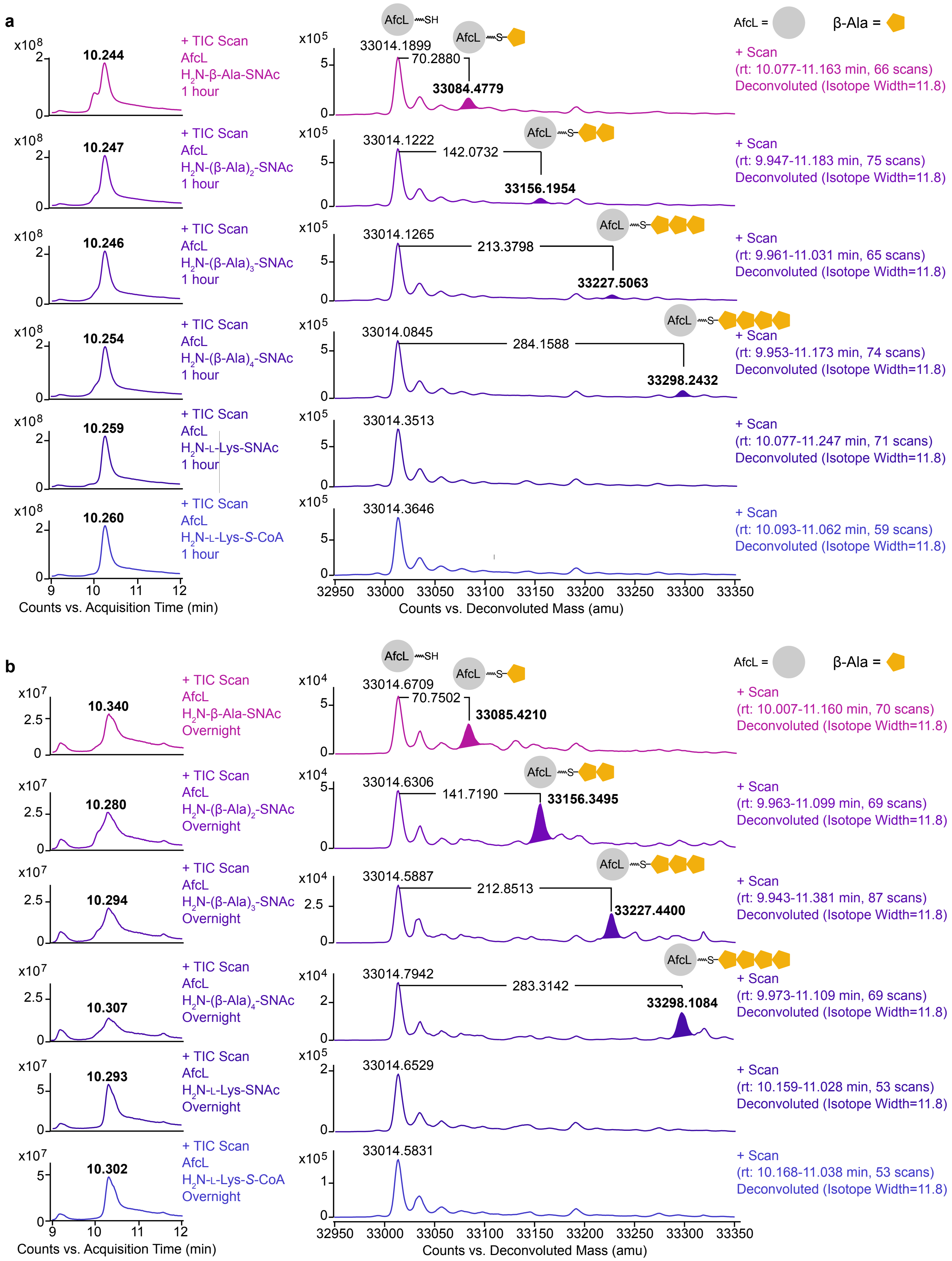

Supplementary Figure 50. Loading of AfcL monitored by ESI (+)-Q-TOF. **a**) 1-h incubation of AfcL with substrates (from top to bottom: H2N-β-Ala-SNAc, H2N-(β-Ala)2-SNAc, H2N-(β-Ala)3-SNAc, H2N-(β-Ala)4-SNAc, H2N-L-Lys-SNAc, or H2N-L-Lys-*S*-CoA). The successful loading was confirmed by the detection of the mass difference of an additional 71, 142, 213, and 284 amu. **b**) Overnight incubation.

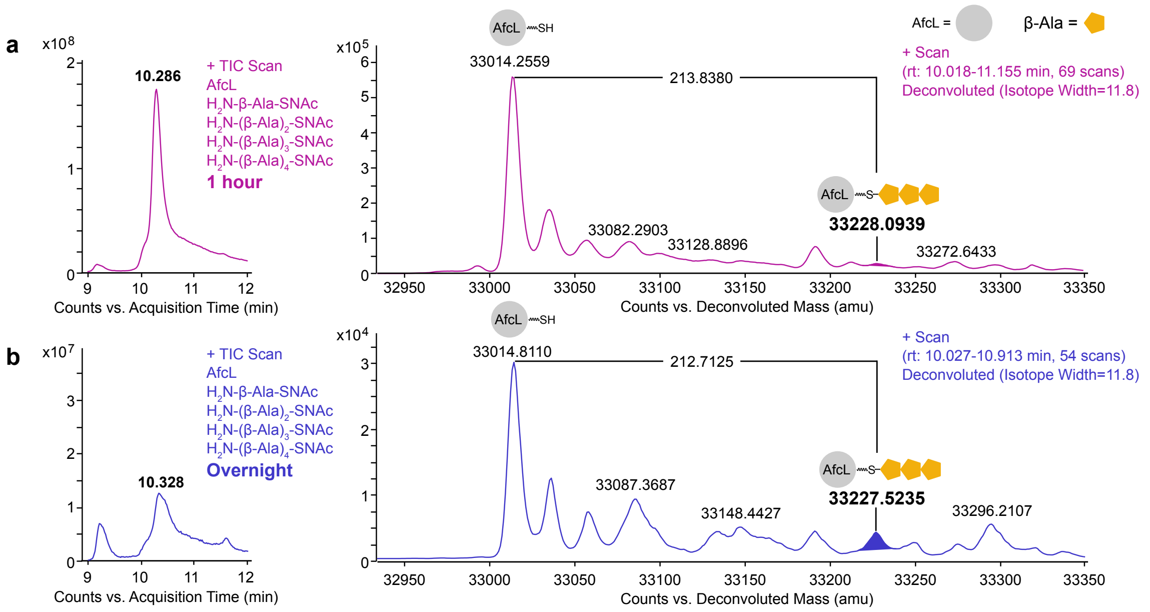

Supplementary Figure 51. Competitive loading of AfcL monitored by ESI (+)-Q-TOF. **a**) 1-h incubation of AfcL together with the mixture of four thioester mimics H_2N-β-Ala-SNAc, H_2N-(β-Ala)$_2$-SNAc, H_2N-(β-Ala)$_3$-SNAc, and H_2N-(β-Ala)$_4$-SNAc. Only the mass difference of an additional 213 amu was detected indicating the successful loading of (β-Ala)$_3$. **b**) Overnight incubation.

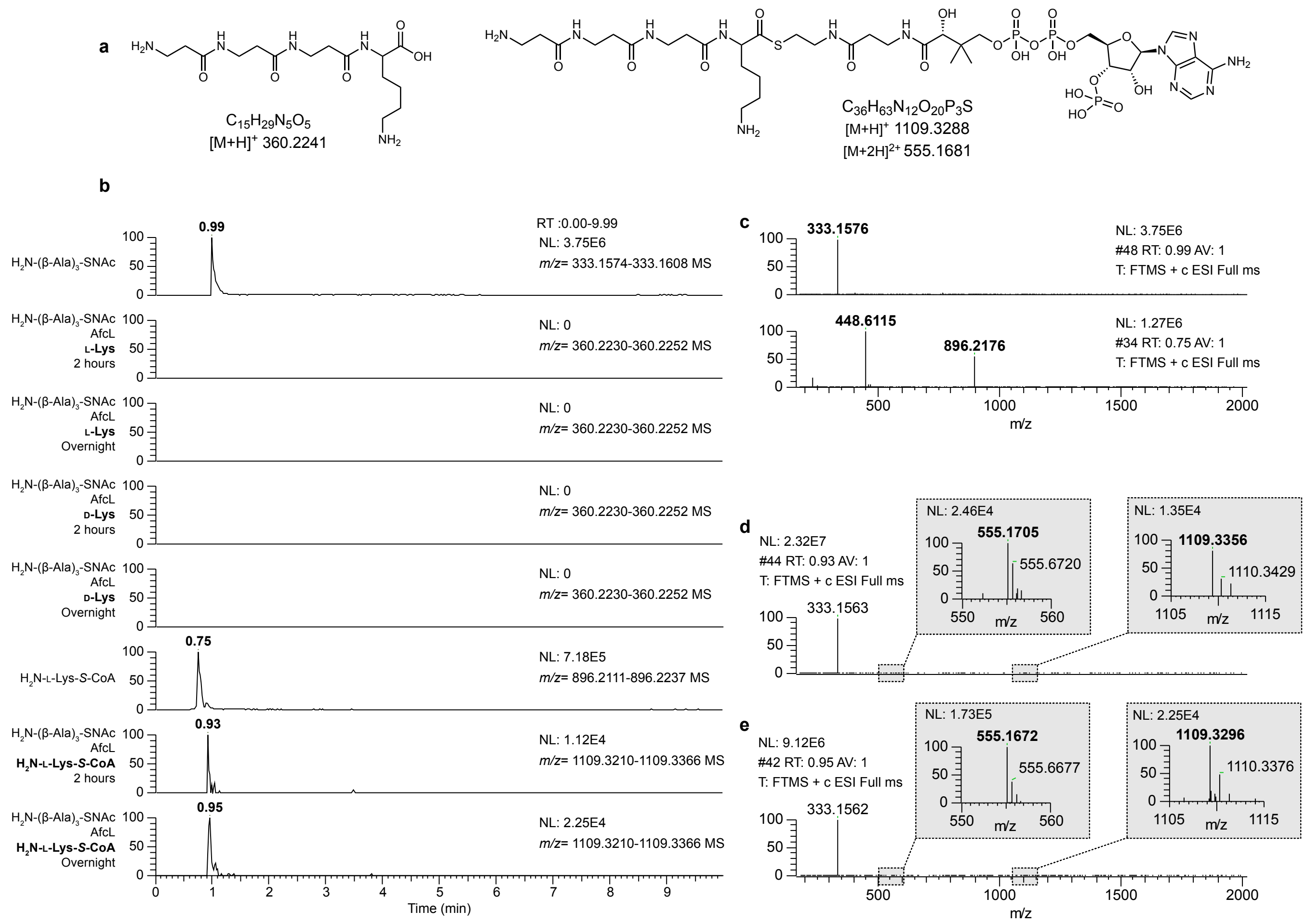

Supplementary Figure 52. Acyl-release from AfcL monitored by LTQ-Orbitrap XL. **a**) Potential products H_2N-(β-Ala)$_3$-Lys and H_2N-(β-Ala)$_3$-Lys-*S*-CoA. **b**) EIC of starting materials and expected products under different reaction conditions (from top to bottom: H_2N-(β-Ala)$_3$-SNAc, incubation with L-Lys for 2 hours and overnight, incubation with D-Lys for 2 hours and overnight, H_2N-L-Lys-*S*-CoA, incubation with H_2N-L-Lys-*S*-CoA for 2 hours and overnight. **c**) MS spectra of the starting materials H_2N-(β-Ala)$_3$-SNAc and H_2N-L-Lys-*S*-CoA from b) with t_R at 0.99 and 0.75 min, respectively. The production of H_2N-(β-Ala)$_3$-Lys-*S*-CoA (*m/z* 555, *m/z* 1109) was detected when incubated with H_2N-L-Lys-*S*-CoA for 2 hours **d**) and overnight **e**). The MS spectra were acquired from b) with t_R at 0.93 and 0.95 min, respectively.

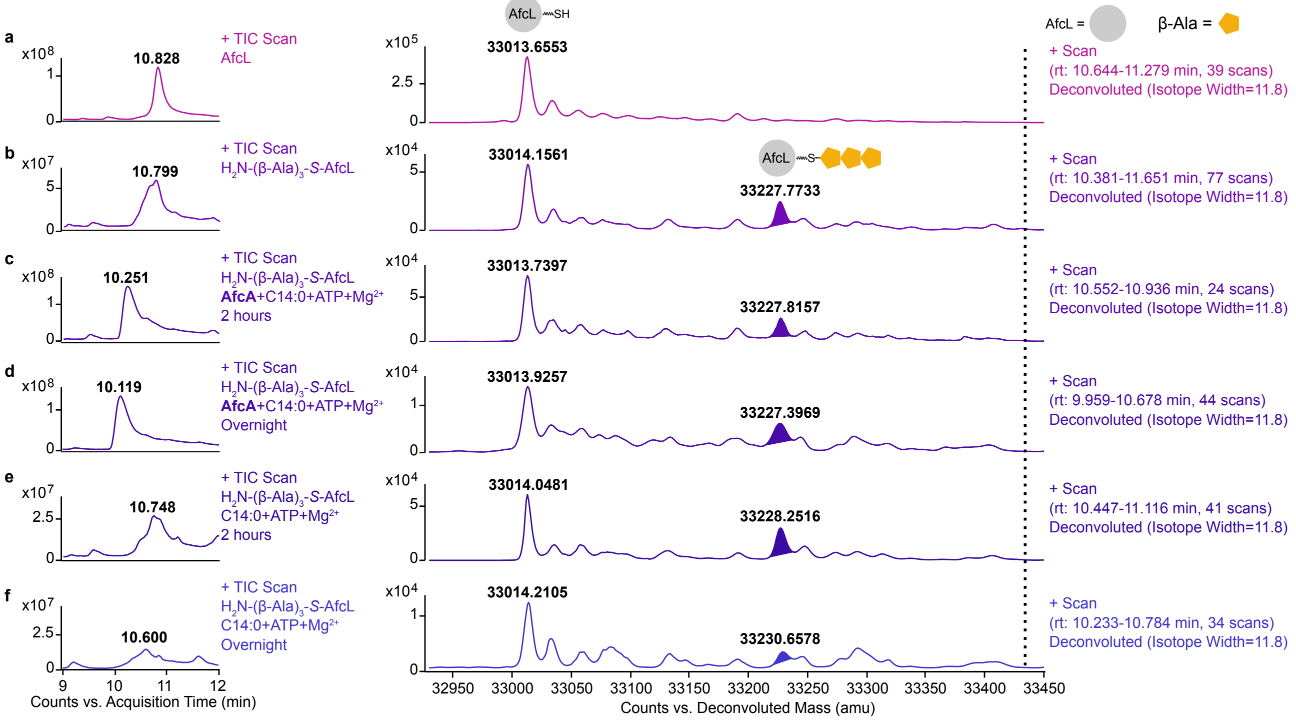

Supplementary Figure 53. Loading test of fatty acid onto H_2N-(β-Ala)$_3$-*S*-AfcL monitored by ESI (+)-Q-TOF. **a**) AfcL as the reference. **b**) H_2N-(β-Ala)$_3$-*S*-AfcL obtained after incubation of H_2N-(β-Ala)$_3$-SNAc and AfcL for overnight and the excess H_2N-(β-Ala)$_3$-SNAc was removed through serial dilution and centrifugation processes. Incubation of H_2N-(β-Ala)$_3$-*S*-AfcL with AfcA, myristic acid (C14:0), ATP and Mg^{2+} for **c**) 2 hours and **d**) overnight, and the control reactions without AfcA for **e**) 2 hours and **f**) overnight. The expected mass of myristoyl-(β-Ala)$_3$-*S*-AfcL is indicated with the dashed line.

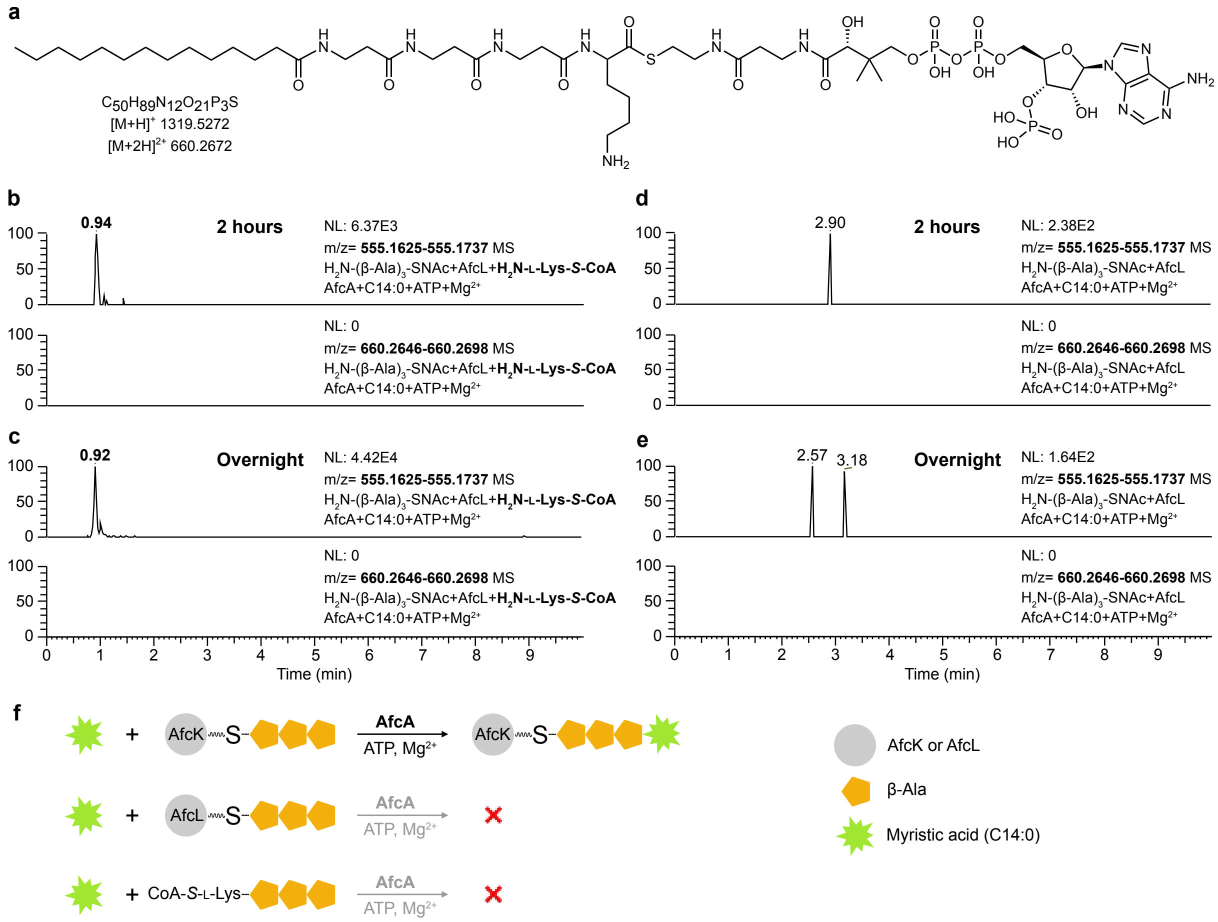

Supplementary Figure 54. Loading test of fatty acid onto H_2N-(β-Ala)$_3$-L-Lys-*S*-CoA monitored by LTQ-Orbitrap XL. **a**) Expected product myristoyl-(β-Ala)$_3$-L-Lys-*S*-CoA. EIC of H_2N-(β-Ala)$_3$-L-Lys-*S*-CoA (*m/z* at 555.1681) and myristoyl-(β-Ala)$_3$-L-Lys-*S*-CoA (*m/z* at 660.2672) after incubation of H_2N-(β-Ala)$_3$-SNAc, AfcL, H_2N-L-Lys-*S*-CoA, AfcA, myristic acid (C14:0), ATP and Mg^{2+} for 2 hours **b**) and overnight **c**), and the control reactions without H_2N-L-Lys-*S*-CoA for **d**) 2 hours and **e**) overnight. **f**) Acyl-transfer model catalyzed by AfcA exclusively accepting H_2N-(β-Ala)$_3$-*S*-AfcK as substrate.

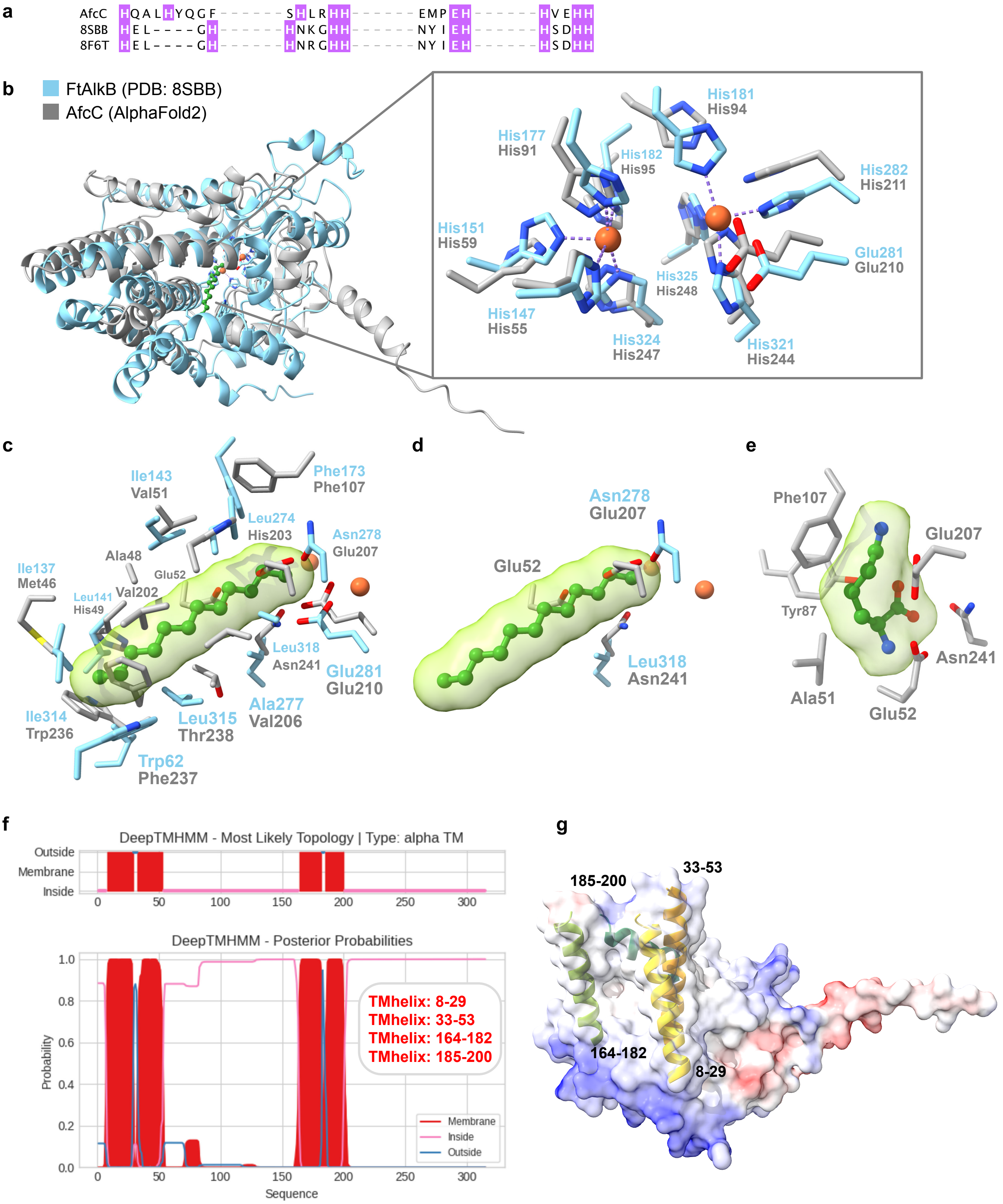

Supplementary Figure 55. Structural analysis of AfcC model. **a**) Alignment of conserved histidine motifs in AfcC, FtAlkB (PDB: 8SBB) and AlkB (PDB: 8F6T).[106,107] **b**) Protein structure superpositions of FtAlkB (***sky blue***) and AlphaFold2-predicted AfcC (***dark gray***)[16–20] combined with dodecane (***ball stick***, ***green***) and irons (***ball***, ***red***). The conserved binding pockets for irons are shown in the zoomed view on the right. **c**) The residues indicate the critical sites involved in the formation of a hydrophobic pocket for dodecane in FtAlkB (***stick***, ***sky blue***) and the predicted hydrophobic residues in AfcC in dark gray by superimposing the crystal structure of FtAlkB and the predicted structure of AfcC. **d**) The hydrophilic residues (Glu52 and Asn241) on the bottom of the pocket in AfcC are highlighted. **e**) The predicted binding model of L-Lys (***ball stick***, ***green***) in AfcC, which was modelled by AutoDock Vina. f) Four transmembrane (TM) helixes in AfcC were predicted using DeepTMHMM.[108] g) The predicted transmembrane helixes are highlighted in the electrostatic surface of AfcC, TMhelix: 8-29 (***yellow***), TMhelix: 33-53 (***orange***), TMhelix: 164-182 (***green***) and TMhelix: 185-200 (***deepgreen***). (Electrostatic surface: coloring ranging from red for negative potential through white to blue for positive potential).

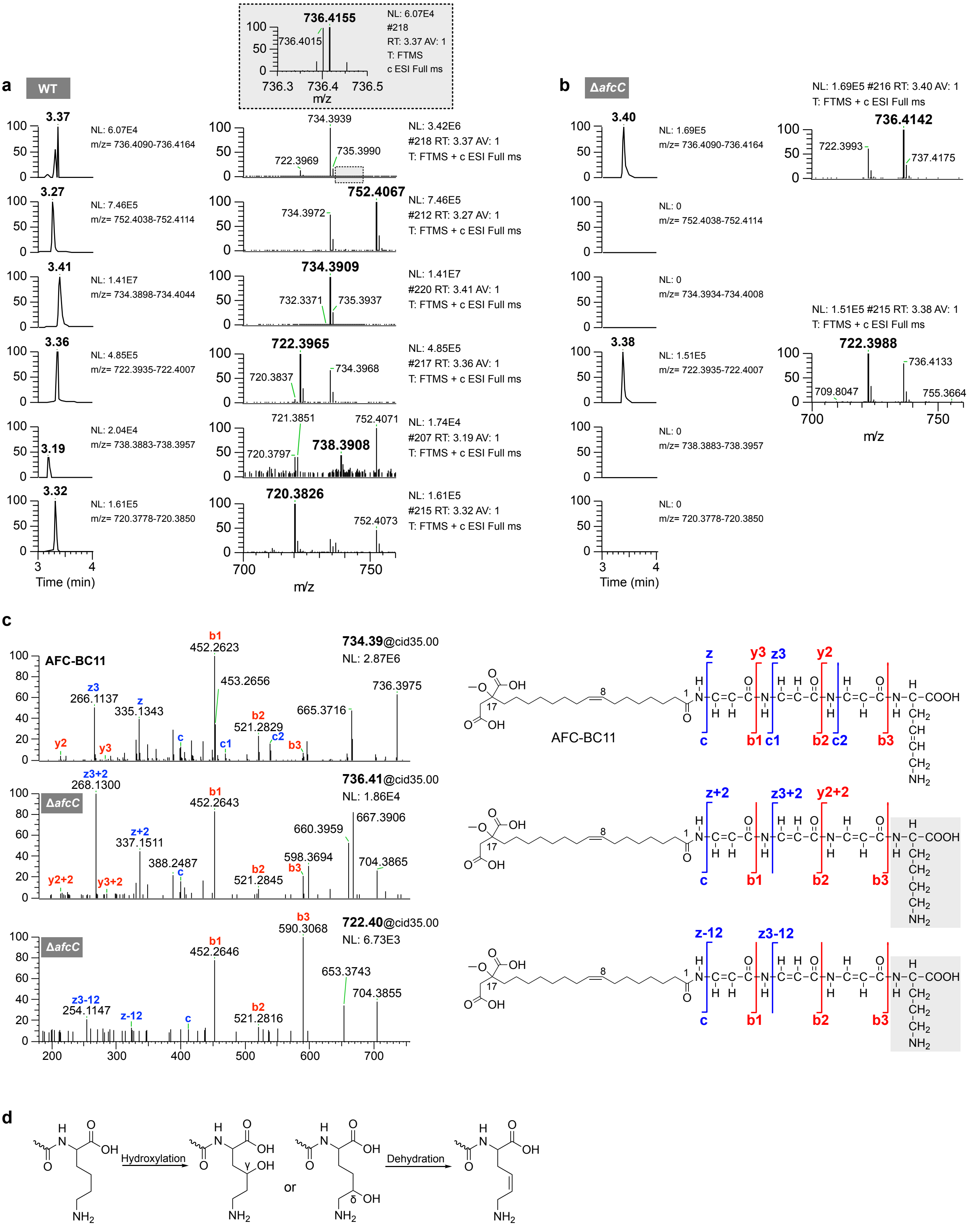

Supplementary Figure 56. Metabolic profiling acquired with LTQ-Orbitrap XL of **a**) WT (*B. pyrrocinia* DSM 10685) and **b**) Δ*afcC*. The production of AFC-BC11 (*m/z* 734) and its congeners (*m/z* 752, *m/z* 738, *m/z* 720) was completely abolished in Δ*afcC*. Only two congeners were still observed from Δ*afcC* (*m/z* 736 and *m/z* 722). **c**) MS/MS spectra of *m/z* 734 observed from WT (as reference), of *m/z* 736 and *m/z* 722 from Δ*afcC*, which identified two families of AFC-BC11-like metabolites with either Lysine (*m/z* 736, *m/z* 752, *m/z* 734) or Ornithine at the C-terminus (*m/z* 722, *m/z* 738, *m/z* 720), respectively. **d**) Proposed formation of the double bond in DHLys by initial hydroxylation and subsequent dehydration.

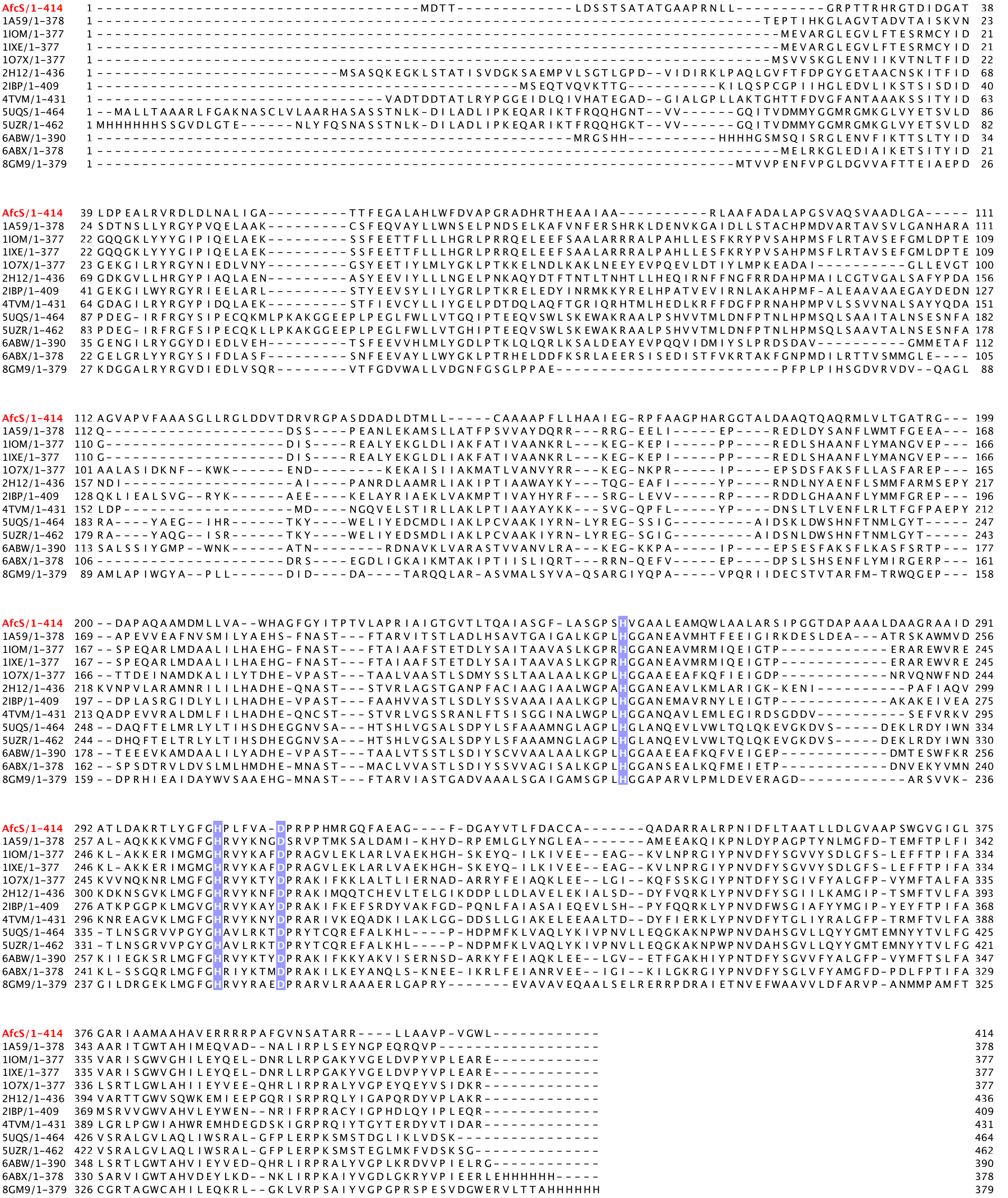

Supplementary Figure 57. Alignment of AfcS with selected citrate synthases revealed the conserved catalytic triad (H253, H305, D311) (***cyan***) in AfcS. The citrate synthases aligned here are 1A59,[109] 1IOM,[110] 1IXE,[110] 1O7X,[111] 2H12,[112] 2IBP,[113] 4TVM,[114] 5UQS,[115] 5UZR,[115] 6ABW,[116] 6ABX,[117] and 8GM9.[118]

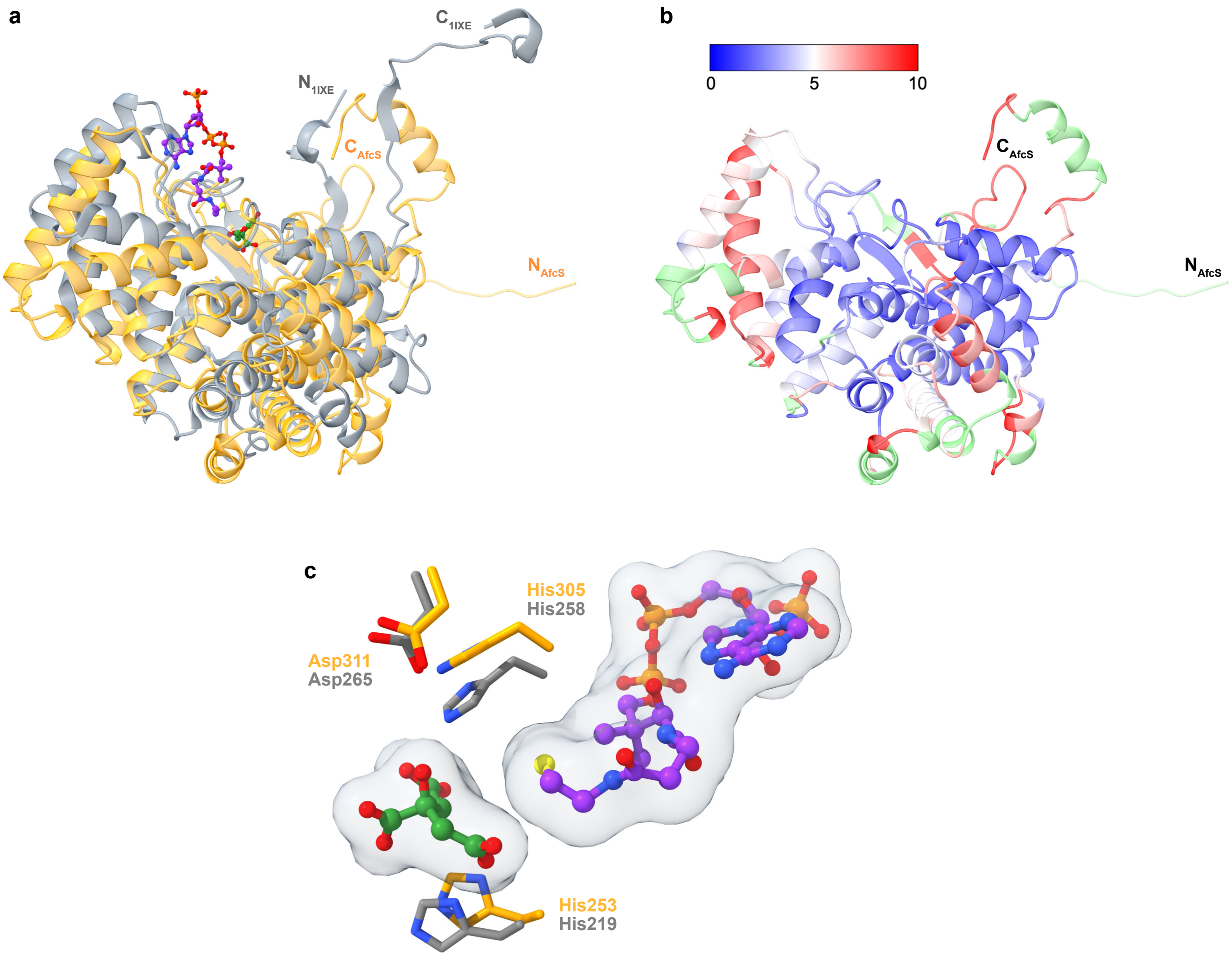

Supplementary Figure 58. Structural analysis of AfcS. **a**) Comparative alignment of AfcS (AlphaFold2, ***orange***)[16–20] with the citrate synthase (PDB: 1IXE, ***gray***).[110] The CoA (***purple***) and the citrate acid (***green***) are illustrated as a ball-and-stick model. **b**) The root mean square deviation (RMSD) map highlights the structural distinctions between AfcS and 1IXE. **c**) Alignment of the catalytic triad in AfcS (***orange***) and in 1IXE (***gray***) by superimposing their structures.

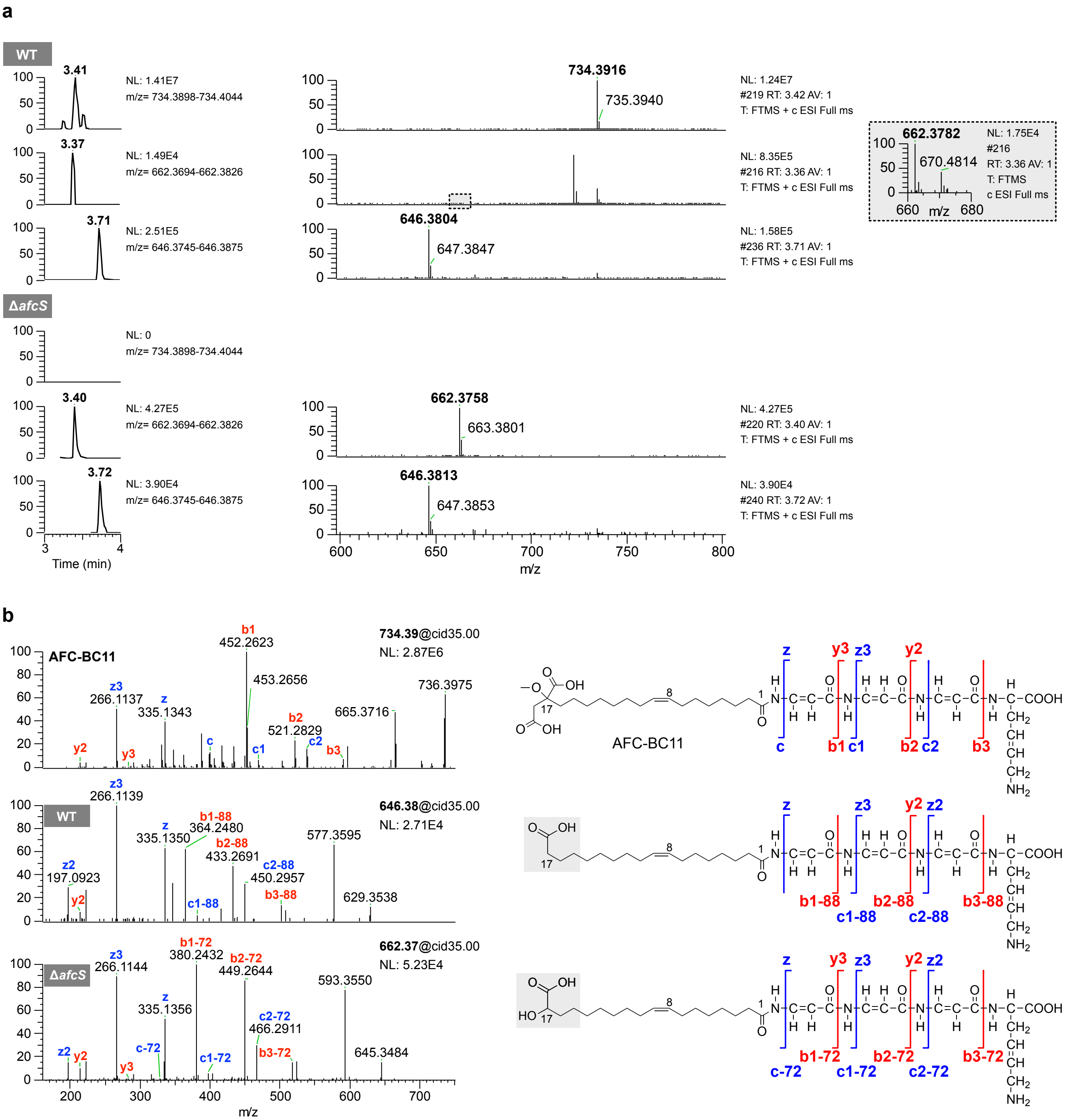

Supplementary Figure 59. Metabolic profiling of Δ*afcS*. **a**) Metabolic profiling acquired with LTQ-Orbitrap XL of WT (*B. pyrrocinia* DSM 10685, ***top***) and Δ*afcS* (***bottom***). The production of AFC-BC11 (*m/z* 734) was completely abolished in Δ*afcS*, and *m/z* 662 and *m/z* 646 became the main metabolites in Δ*afcS*. **b**) MS/MS spectra (*left*) of *m/z* 734 observed from WT (as reference), of *m/z* 646 (WT), and *m/z* 662 (Δ*afcS*) suggesting a conserved peptide part (***right***).

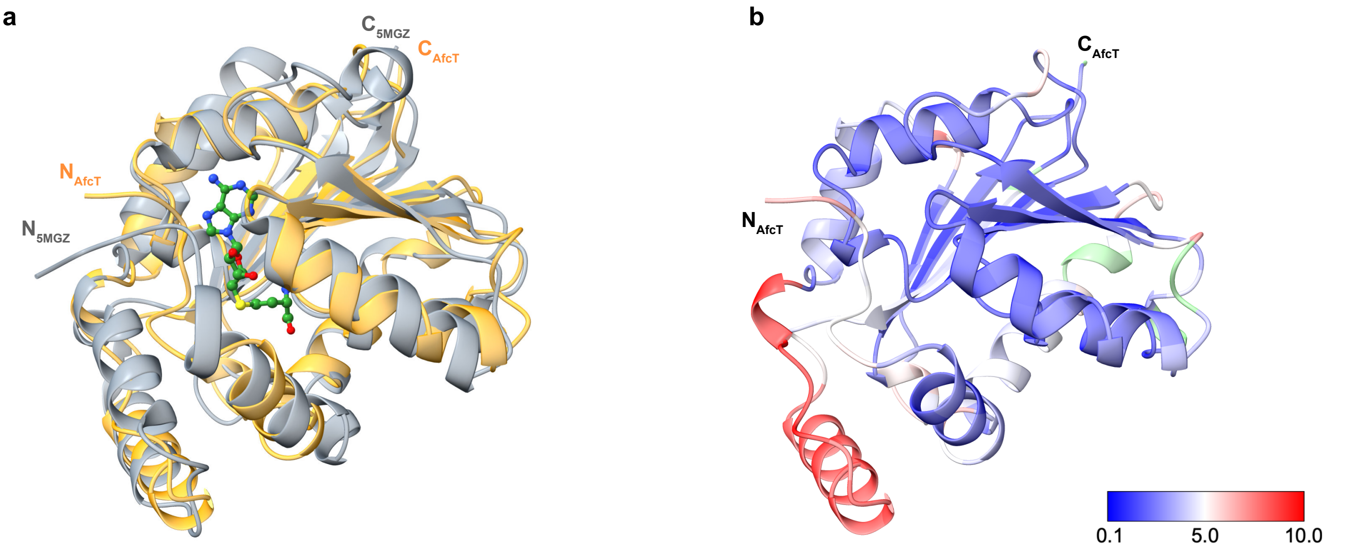

Supplementary Figure 60. Structural analysis of AfcT. **a**) Comparative alignment of AfcT (AlphaFold2, ***orange***)[16–20] with the 8-demethylnovbiocic acid methyltransferase (PDB: 5MGZ, ***gray***).[119] The SAH (***green***) is illustrated as a ball-and-stick model. **b**) The root mean square deviation (RMSD) map highlights the structural distinctions between AfcT and 5MGZ.

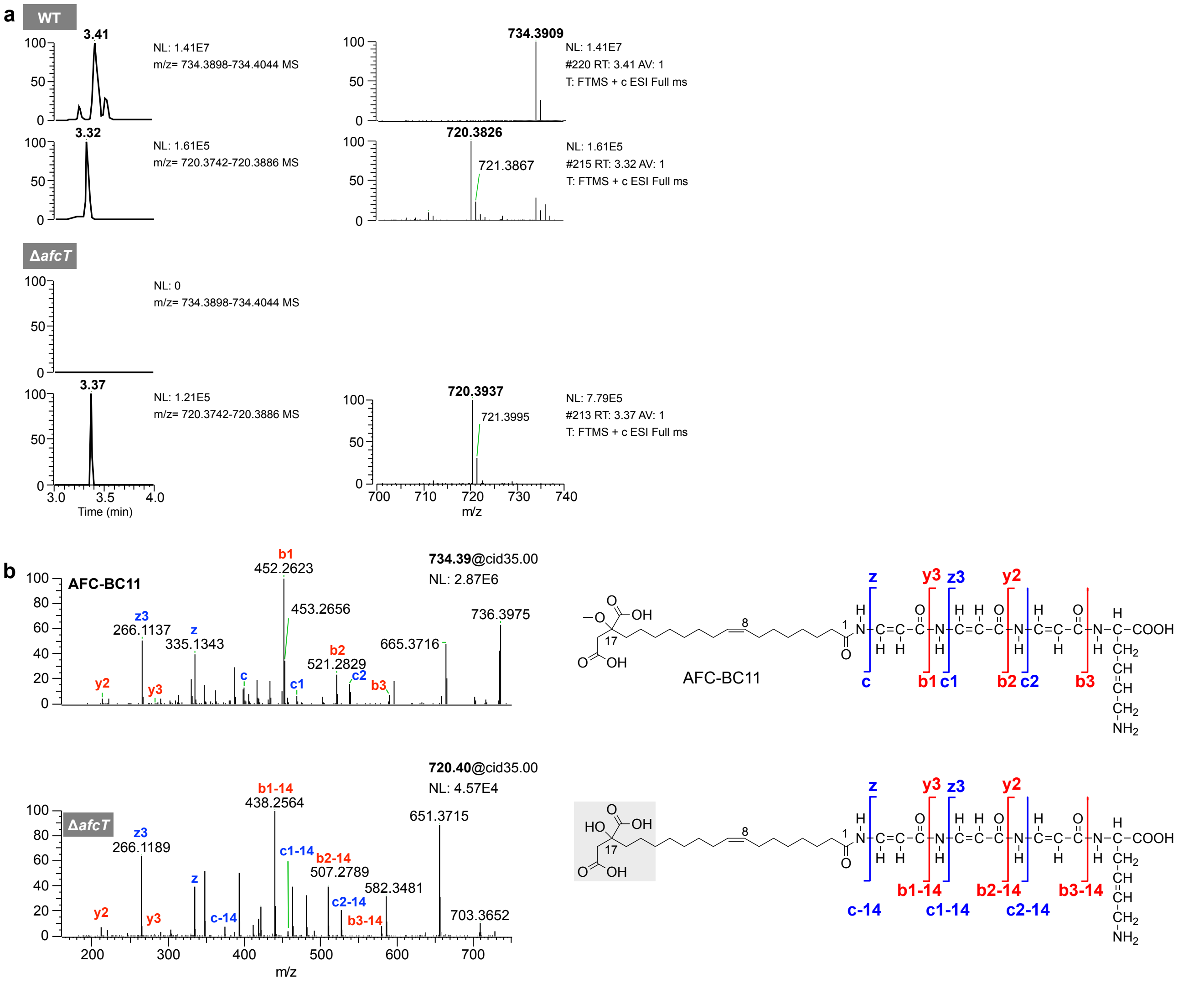

Supplementary Figure 61. Metabolic profiling of Δ*afcT*. **a**) Metabolic profiling acquired with LTQ-Orbitrap XL of WT (*B. pyrrocinia* DSM 10685, ***top***) and Δ*afcT* (***bottom***). The production of AFC-BC11 (*m/z* 734) was completely abolished in Δ*afcT*. And *m/z* 720 became the main metabolites in Δ*afcT*. **b**) MS/MS spectra of *m/z* 734 observed from WT (as reference) and *m/z* 720 (Δ*afcT*).

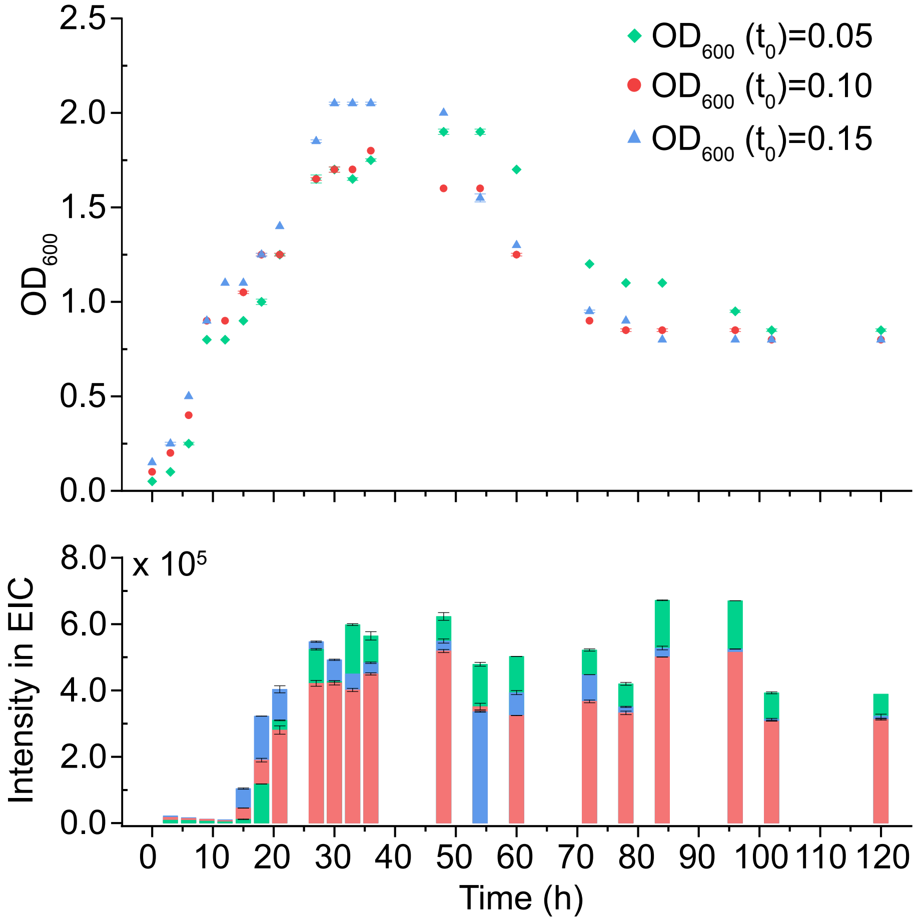

Supplementary Figure 62. The time course of *B. puraquae* DSM 103137 cell growth, with initial OD_{600} values of 0.05 (***green***), 0.10 (***pink***), and 0.15 (***blue***) respectively (***top***). The abundance of AFC-BC11 produced was determined from the intensity in EIC (***bars***) acquired using LC-HRMS (Exactive, ***bottom***). Sample preparation was carried out at specific time points: 3, 6, 9, 12, 15, 18, 21, 27, 30, 33, 36, 48, 54, 60, 72, 78, 84, 96, 102, and 120 h. All experiments were performed in duplicate and error bars indicate the standard deviation.

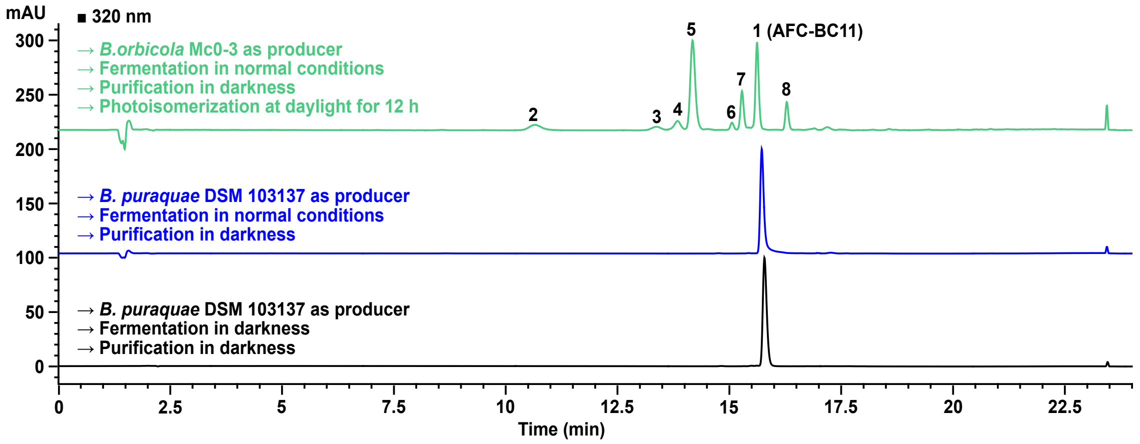

Supplementary Figure 63. AFC-BC11 produced and purified under different conditions (see legend). The parent compound was coded as number 1 and mixture of new photoisomers as 2-8 in the analytical HPLC chromatograms.

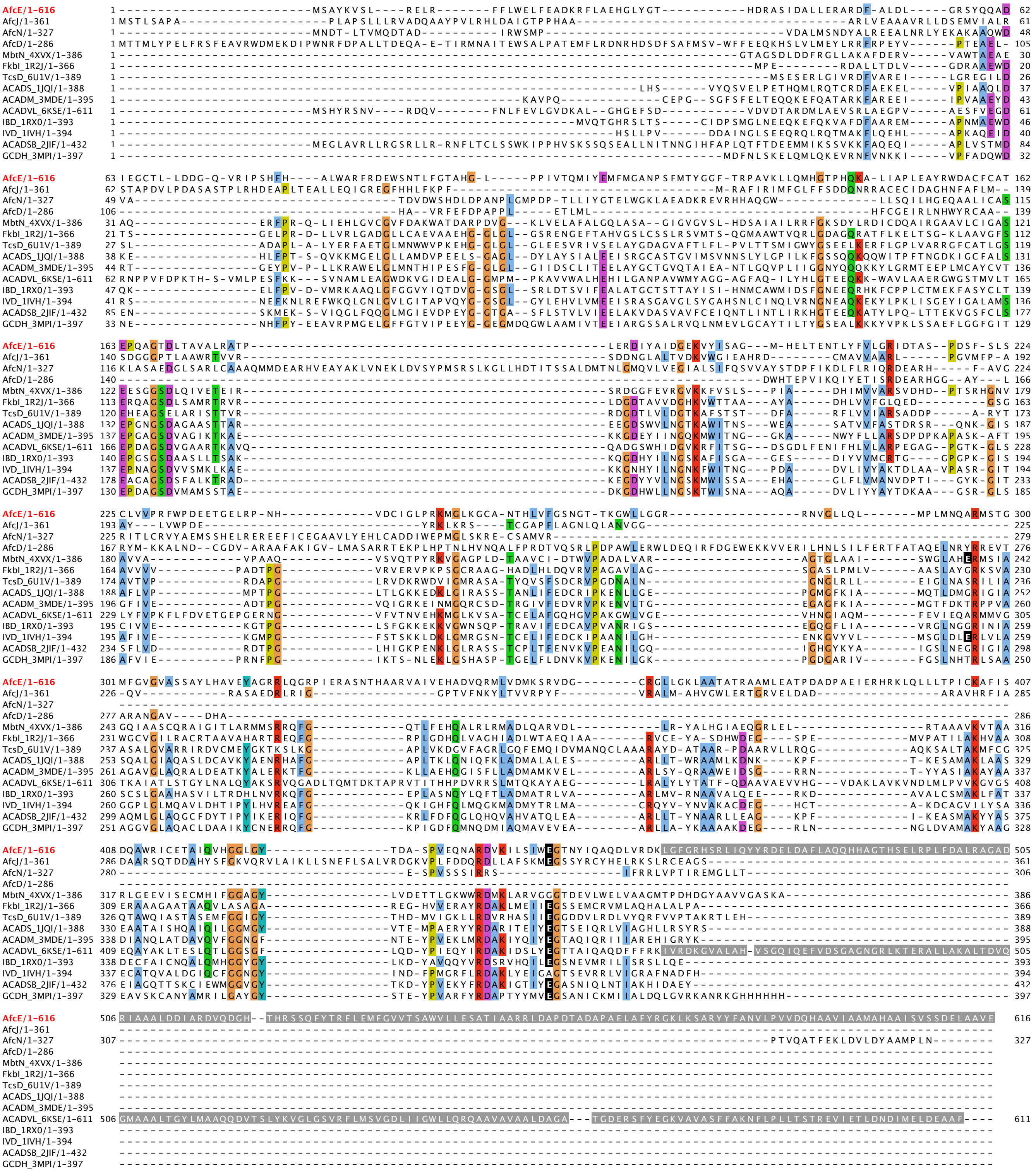

Supplementary Figure 64. Alignment of AfcE, AfcJ, AfcN, and AfcD with selected FAD-dependent dehydrogenases revealed the conserved catalytic glutamic acid residue (***black***). AfcE and ACADVL feature an extra C-terminal sequence (***gray***). The dehydrogenases aligned here are MbtN (PDB:4XVX),[120,121] FkbI (PDB: 1R2J),[122] TcsD (PDB: 6U1V),[123] ACADS (PDB: 1JQI),[124,125] ACADM (PDB: 3MDE),[126,127] ACADVL (PDB: 6KSE),[128,129] IBD (PDB: 1RX0),[130] IVD (1IVH),[131] ACADSB (PDB: 2JIF),[132] and GCDH (PDB: 3MPI).[133,134]

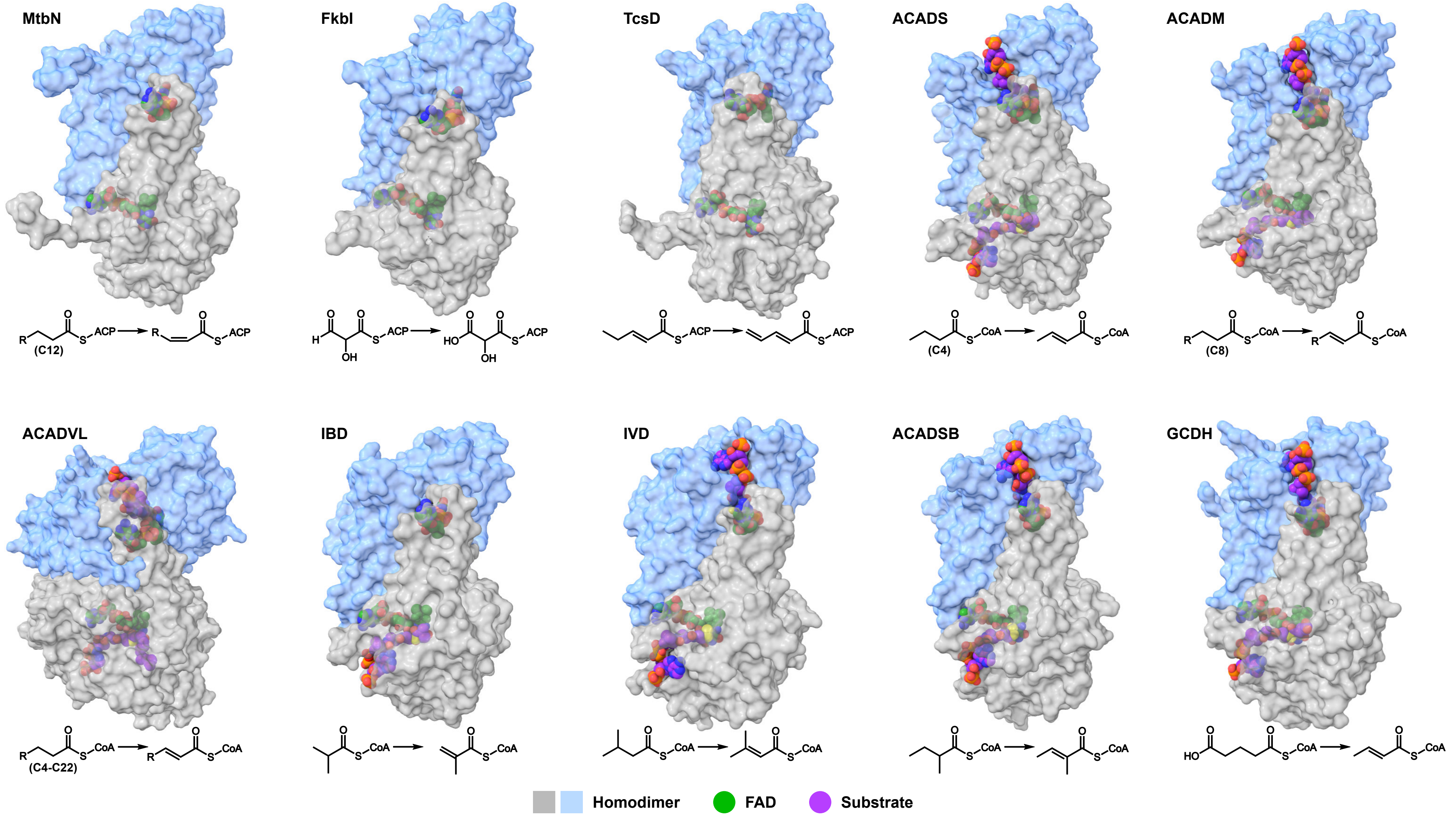

Supplementary Figure 65. Crystal structures of various FAD-dependent dehydrogenases alongside their respective catalytic reactions. The homodimer surfaces are depicted in shades of blue and gray. The FAD cofactors are represented as sphere models (***green***), while the substrates are displayed in purple. The dehydrogenases aligned here are MbtN (PDB:4XVX),[120,121] FkbI (PDB: 1R2J),[122] TcsD (PDB: 6U1V),[123] ACADS (PDB: 1JQI),[124,125] ACADM (PDB: 3MDE),[126,127] ACADVL (PDB: 6KSE),[128,129] IBD (PDB: 1RX0),[130] IVD (1IVH),[131] ACADSB (PDB: 2JIF),[132] and GCDH (PDB: 3MPI).[133,134]

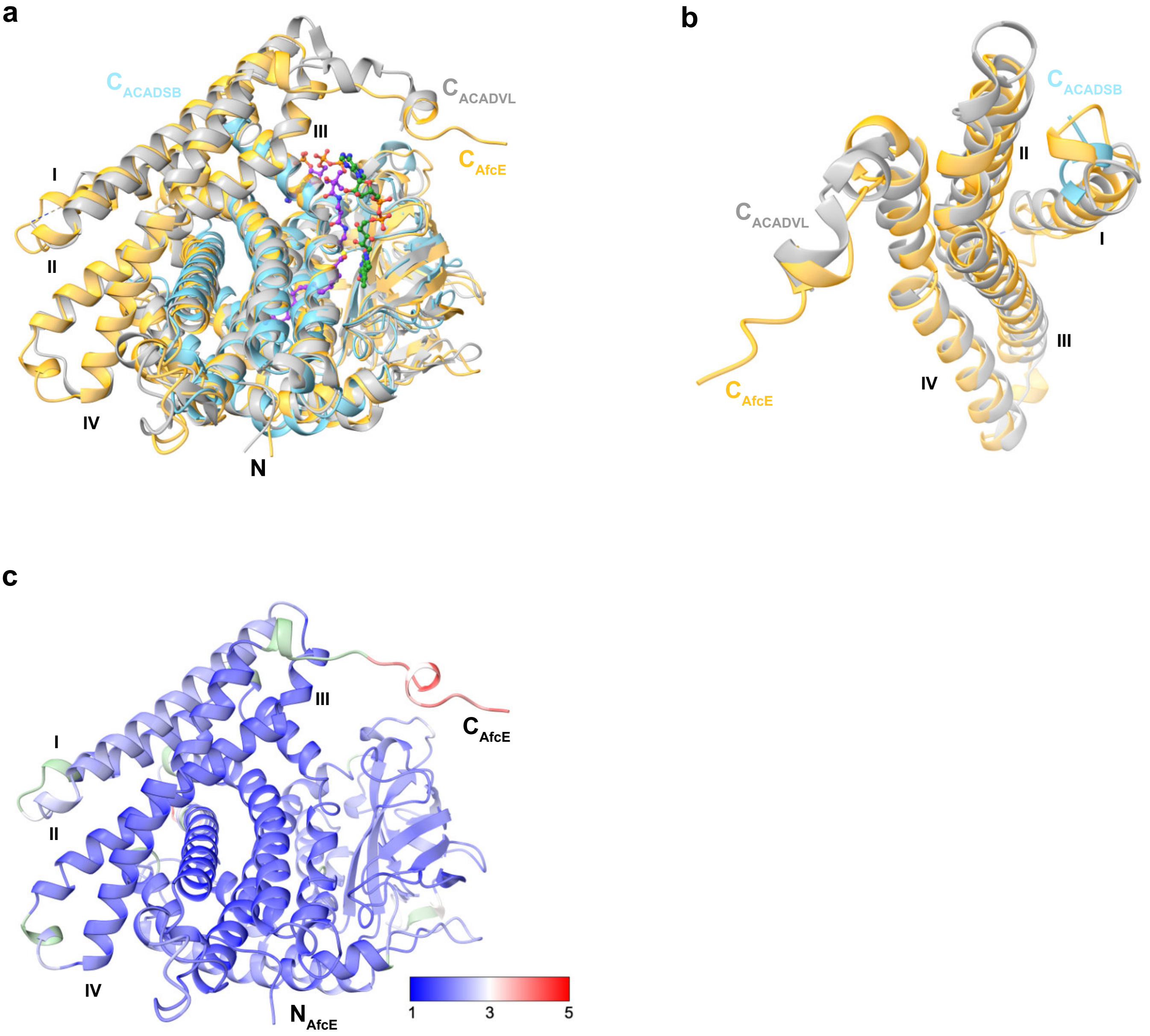

Supplementary Figure 66. Structural analysis of AfcE. **a**) Comparative alignment of AfcE (AlphaFold2, ***orange***)[16–20] with ACADVL (PDB: 6KSE, ***gray***),[128,129] and ACADSB (PDB: 2JIF, ***cyan***).[132] The FAD cofactor is illustrated as a ball-and-stick model (***green***), while the substrate is highlighted in purple. The additional C-terminal helixes in AfcE and ACADVL are labeled as I-IV and presented in **b**). **c**) The root mean square deviation (RMSD) map highlights the structural distinctions between AfcE and ACADVL.

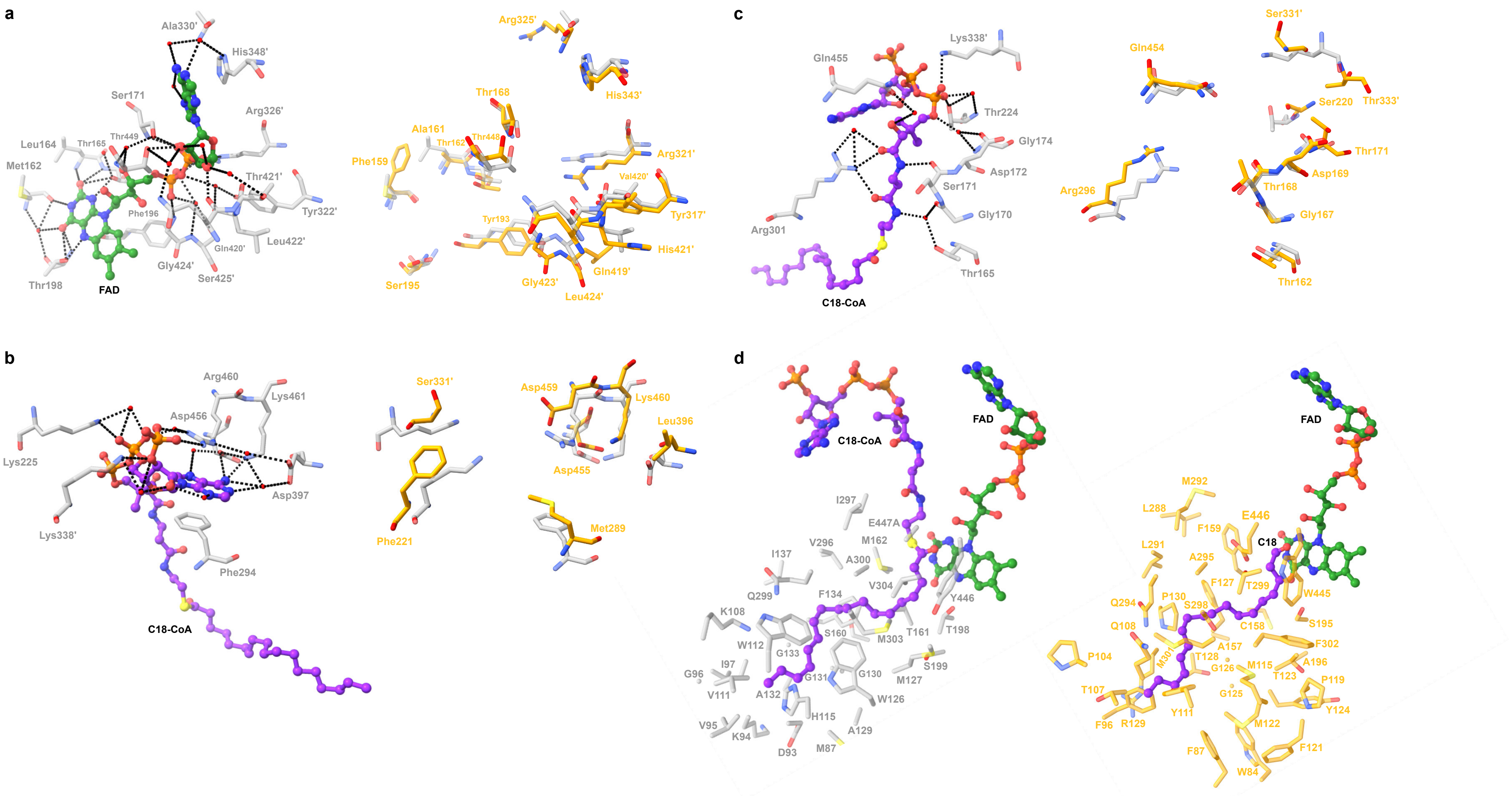

Supplementary Figure 67. Structural analysis of AlphaFold2-predicted AfcE model.[16–20] The binding sites of **a**) FAD cofactor (***ball stick***, ***green***), **b**) the adenosyl portion of CoA (***ball stick, purple***), **c**) the pantetheinyl portion (***ball stick***, ***purple***) and **d**) the hydrophobic pocket of the acyl chain (***ball stick, purple***) in ACADVL (PDB: 6KSE, ***stick***, ***gray***) are displayed on the left.[128,129] The comparative alignment of the binding pockets in AfcE model (***stick***, ***orange***) are shown on the right. This specific arrangement (K225, F294 and R460 in 6KSE) to recognize the 3'-phospho-adenosin moiety is missing in AfcE model, suggesting a CP/AfcK-loaded acyl-peptidyl chain as cognate substrate of AfcE.

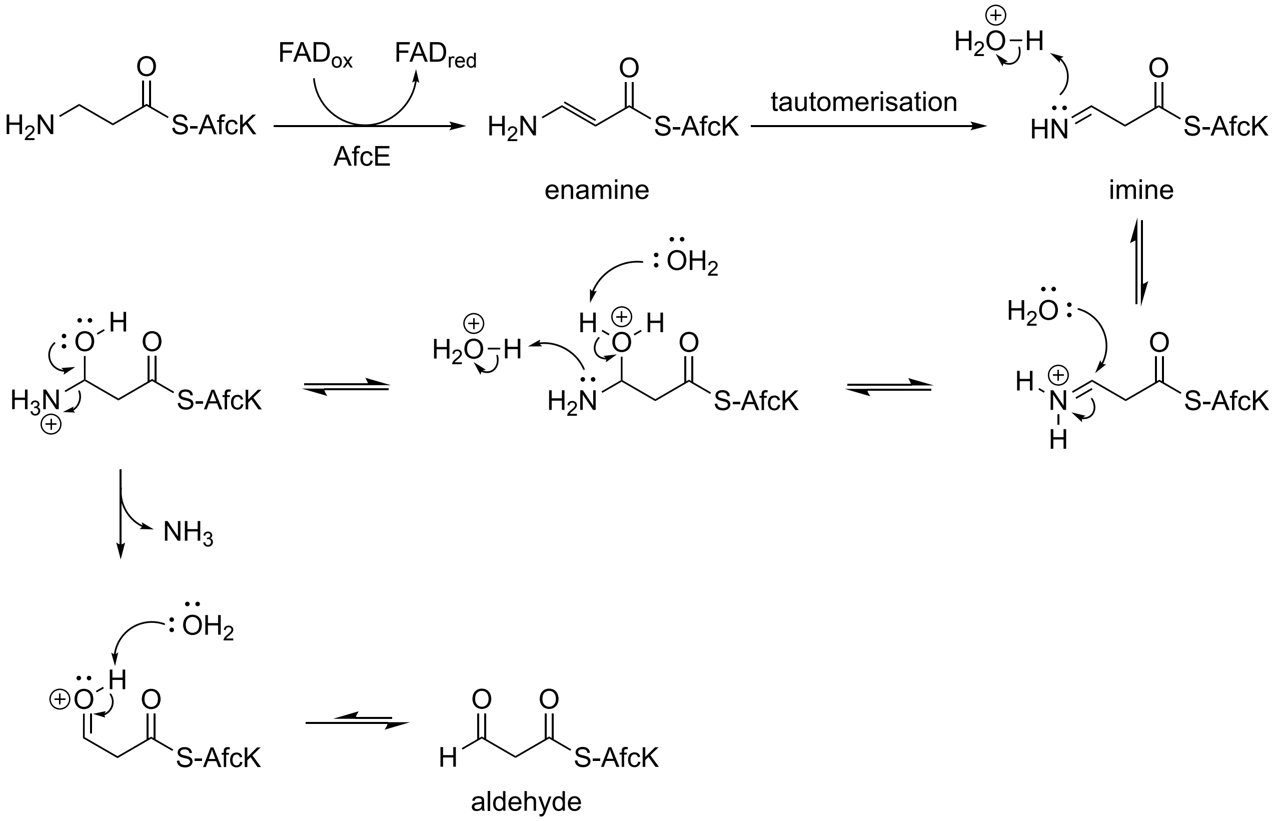

Supplementary Figure 68. Proposed enamine-imine tautomerization of the unprotected/capped terminal DBA.[135]

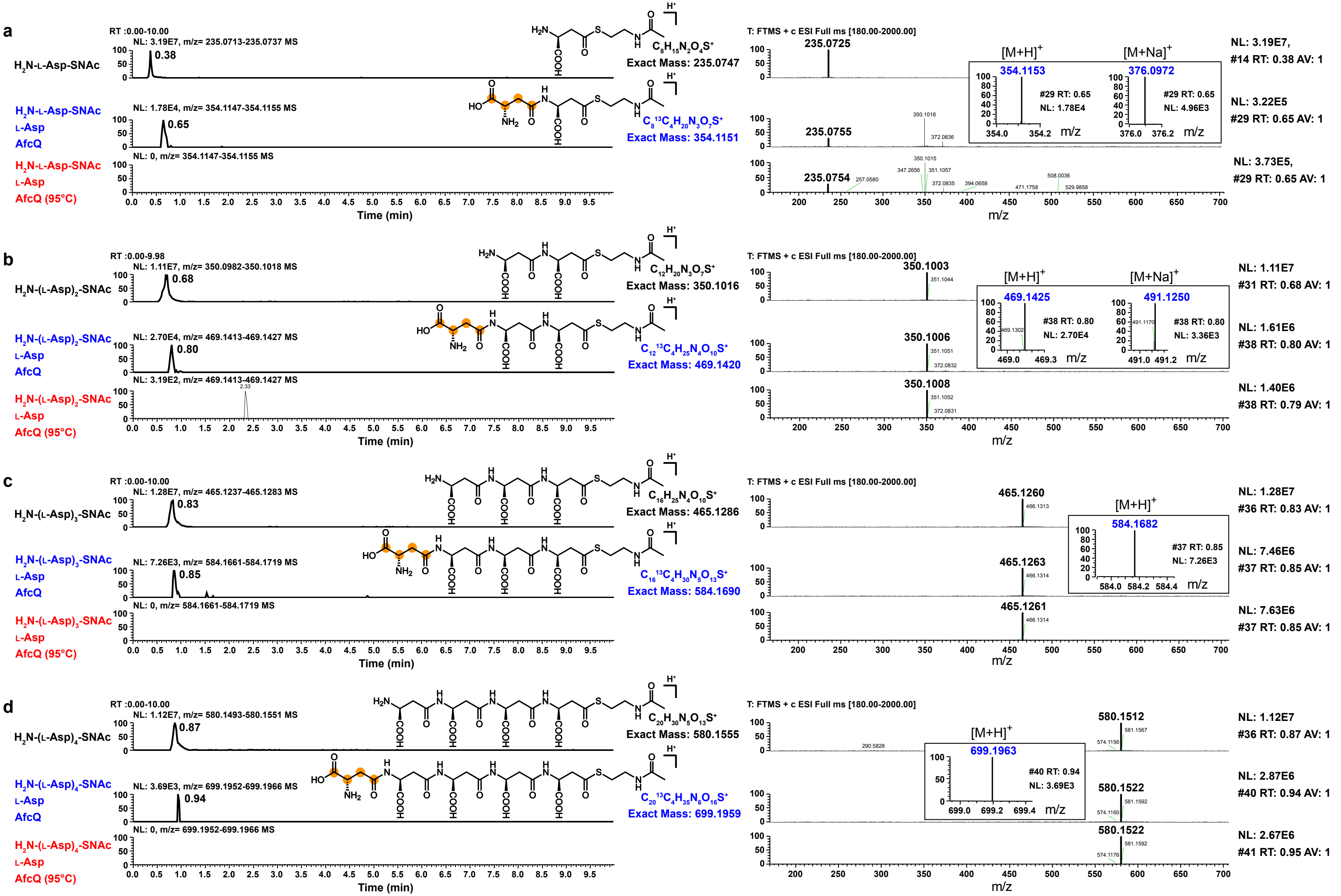

Supplementary Figure 69. EICs of AfcQ-catalyzed reactions with SNAc thioesters acquired by LTQ-Orbitrap XL: **a**) H2N-L-Asp-SNAc, **b**) H2N-(L-Asp)2-SNAc, **c**) H2N-(L-Asp)3-SNAc, and **d**) H2N-(L-Asp)4-SNAc. The zoomed-in images of the anticipated ions are highlighted in blue. L-Asp ($^{13}C_4$) was used for loading (***orange***) and AfcQ was deactivated at 95 °C for 5 min as negative controls.

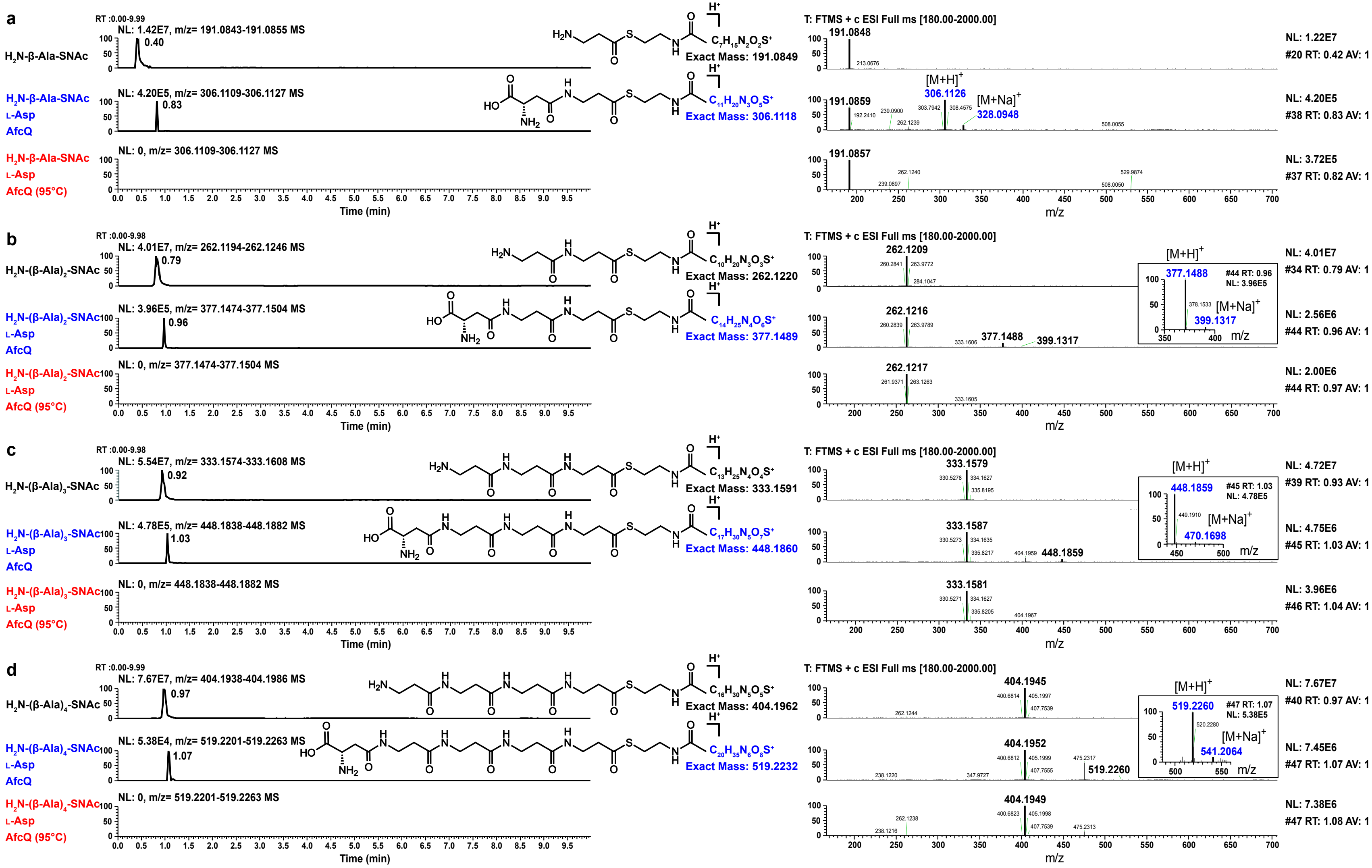

Supplementary Figure 70. EICs of AfcQ-catalyzed reactions with SNAc thioesters acquired by LTQ-Orbitrap XL: **a**) H2N-β-Ala-SNAc, **b**) H2N-(β-Ala)2-SNAc, **c**) H2N-(β-Ala)3-SNAc, and **d**) H2N-(β-Ala)4-SNAc. The zoomed-in images of the anticipated ions are highlighted in blue. L-Asp was used for loading and AfcQ was deactivated at 95 °C for 5 min as negative controls.

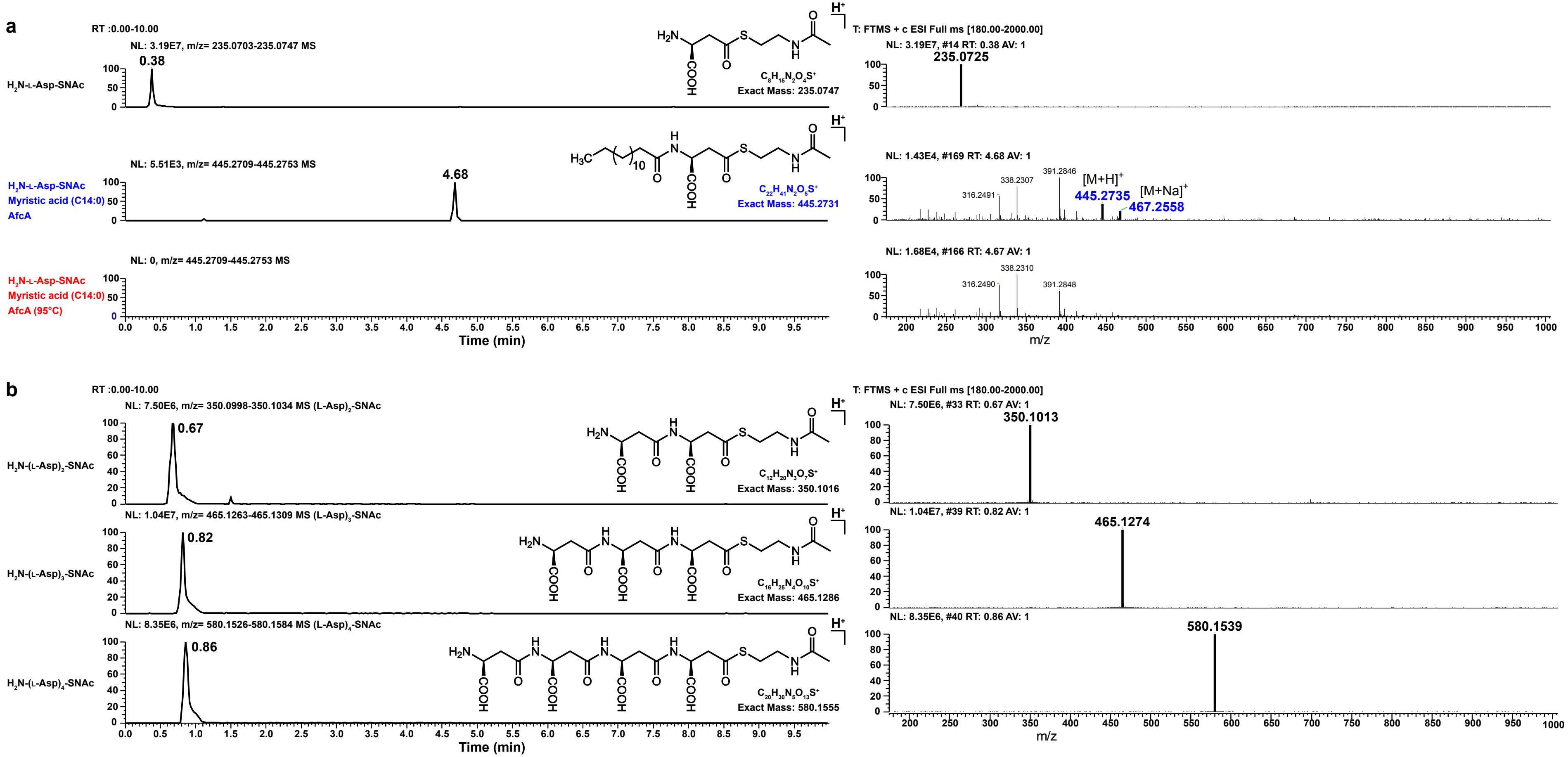

Supplementary Figure 71. EICs of AfcA-catalyzed reactions with SNAc thioesters acquired by LTQ-Orbitrap XL: **a**) H_2N-L-Asp-SNAc. The anticipated ions are highlighted in blue. Myristic acid (C14:0) was used for loading and AfcA was deactivated at 95 °C for 5 min as negative controls. The formation of expected ions was not observed when **b**) H_2N-(L-Asp)$_2$-SNAc, **c**) H_2N-(L-Asp)$_3$-SNAc, and **d**) H_2N-(L-Asp)$_4$-SNAc were tested.

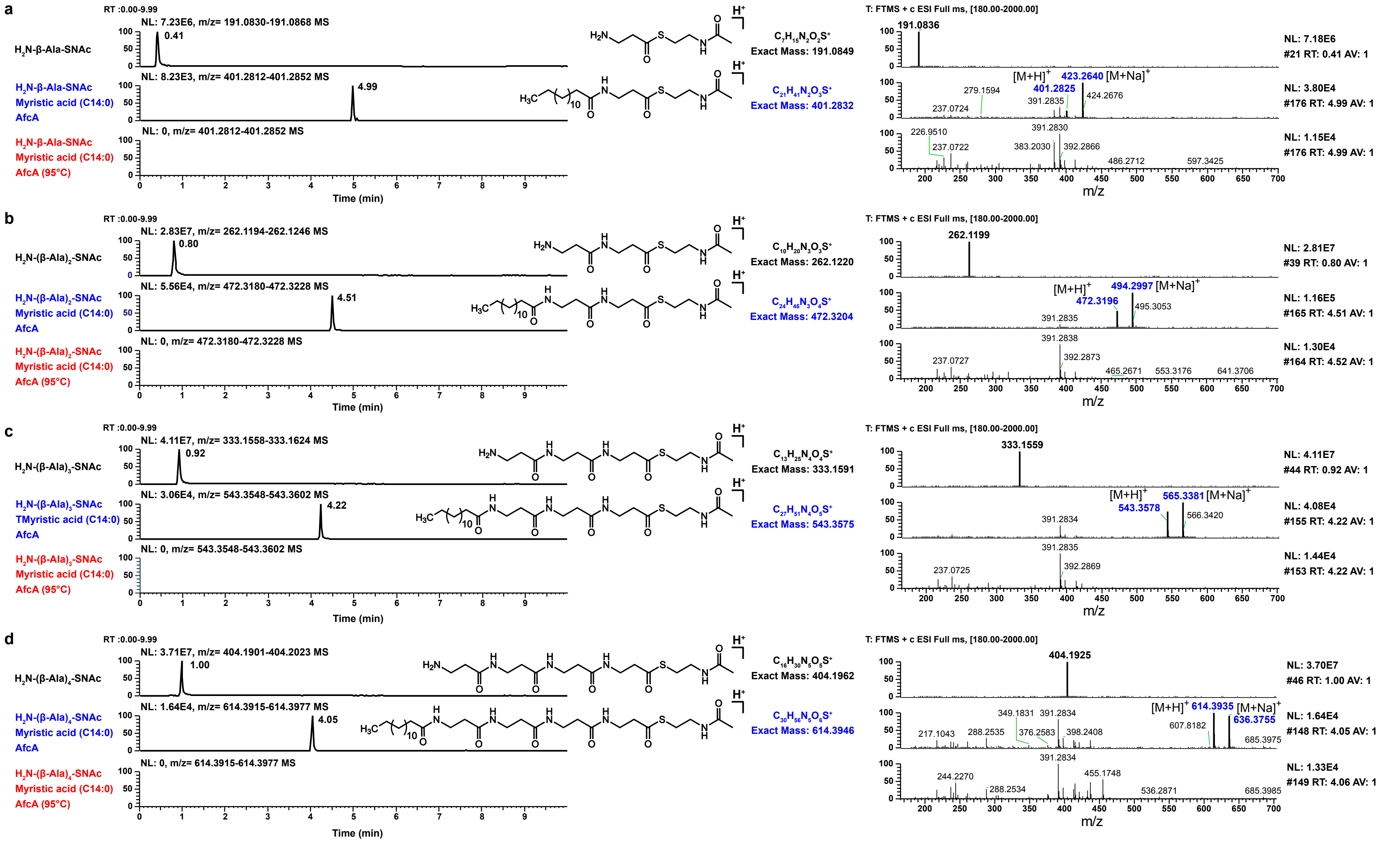

Supplementary Figure 72. EICs of AfcA-catalyzed reactions with SNAc thioesters acquired by LTQ-Orbitrap XL: **a**) H2N-β-Ala-SNAc, **b**) H2N-(β-Ala)2-SNAc, **c**) H2N-(β-Ala)3-SNAc, and **d**) H2N-(β-Ala)4-SNAc. The anticipated ions are highlighted in blue. Myristic acid (C14:0) was used for loading and AfcA was deactivated at 95 °C for 5 min as negative controls.

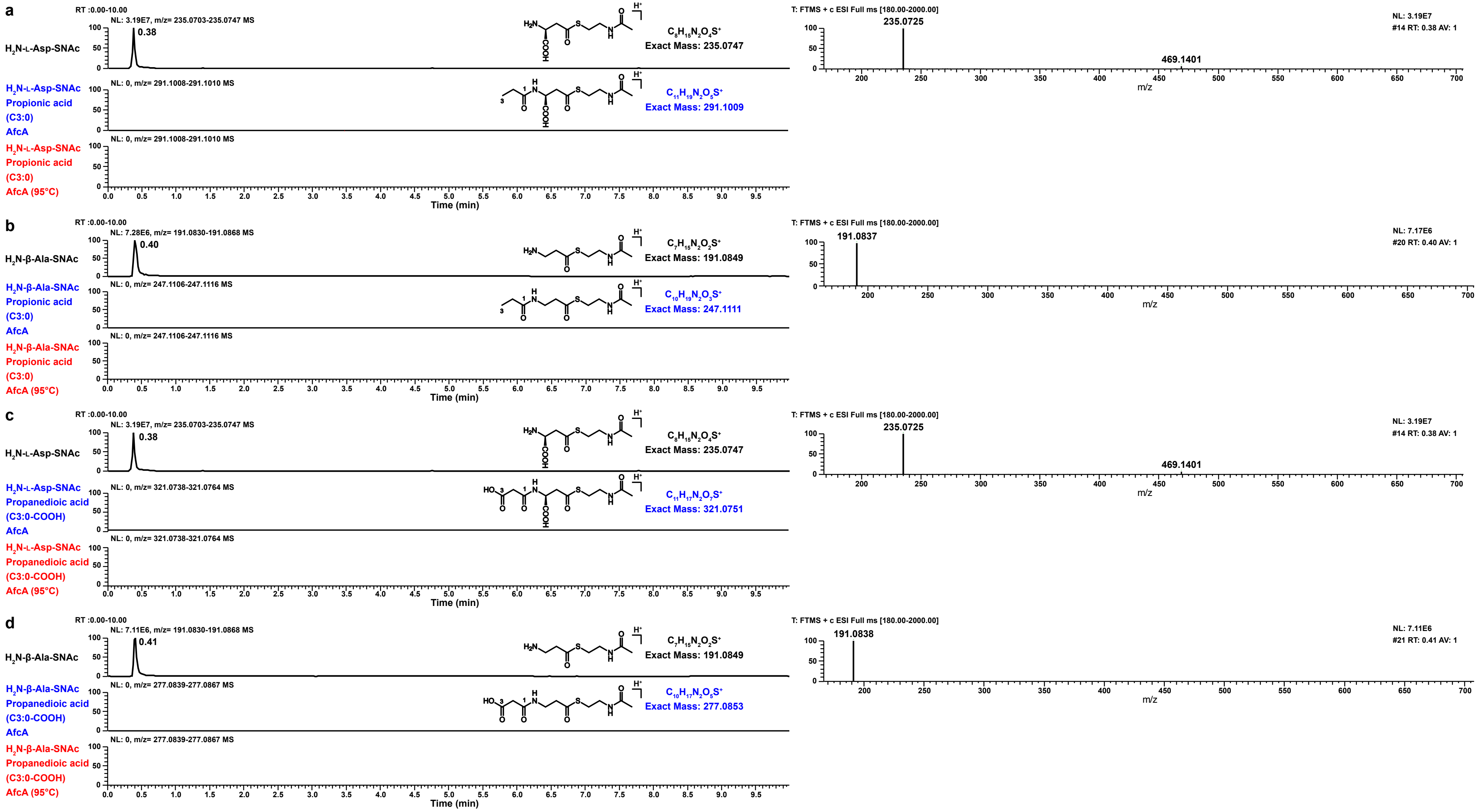

Supplementary Figure 73. EICs of AfcA-catalyzed reactions with SNAc thioesters acquired by LTQ-Orbitrap XL: **a**) propionic acid (C3:0) with H$_2$N-L-Asp-SNAc, **b**) propionic acid (C3:0) with H$_2$N-β-Ala-SNAc, **c**) propanedioic acid (C3:0-COOH) with H$_2$N-L-Asp-SNAc, and **d**) propanedioic acid (C3:0-COOH) with H$_2$N-β-Ala-SNAc. AfcA was deactivated at 95 °C for 5 min as negative controls. The formation of all expected ions was not observed.

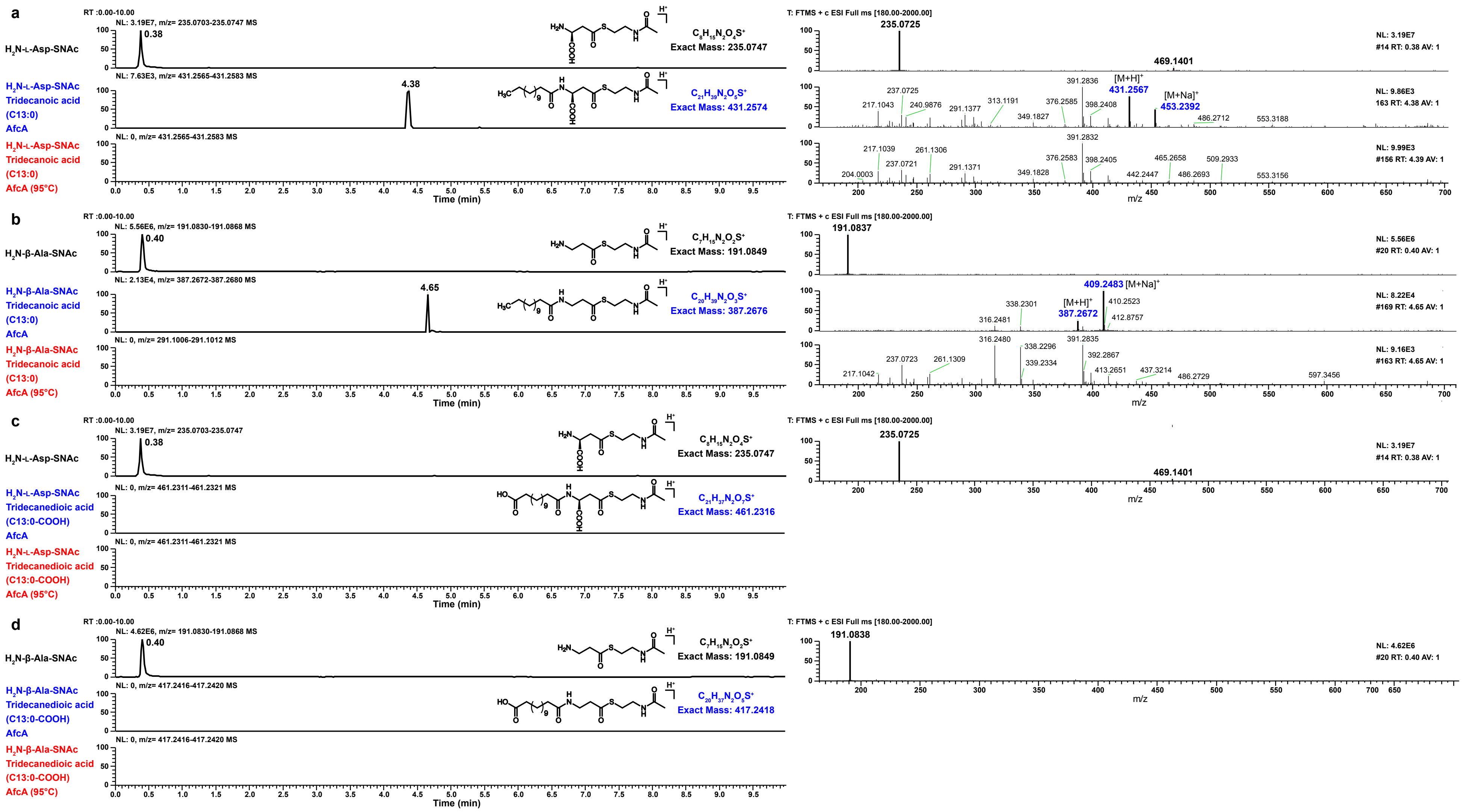

Supplementary Figure 74. EICs of AfcA-catalyzed reactions with SNAc thioesters acquired by LTQ-Orbitrap XL: **a**) tridecanoic acid (C13:0) with H2N-L-Asp-SNAc, **b**) tridecanoic acid (C13:0) with H2N-β-Ala-SNAc, **c**) tridecanedioic acid (C13:0-COOH) with H2N-L-Asp-SNAc, and **d**) tridecanedioic acid (C13:0-COOH) with H2N-β-Ala-SNAc. The anticipated ions are highlighted in blue. AfcA was deactivated at 95 °C for 5 min as negative controls. The formation of expected ions was not observed when tridecanedioic acid (C13) was tested.

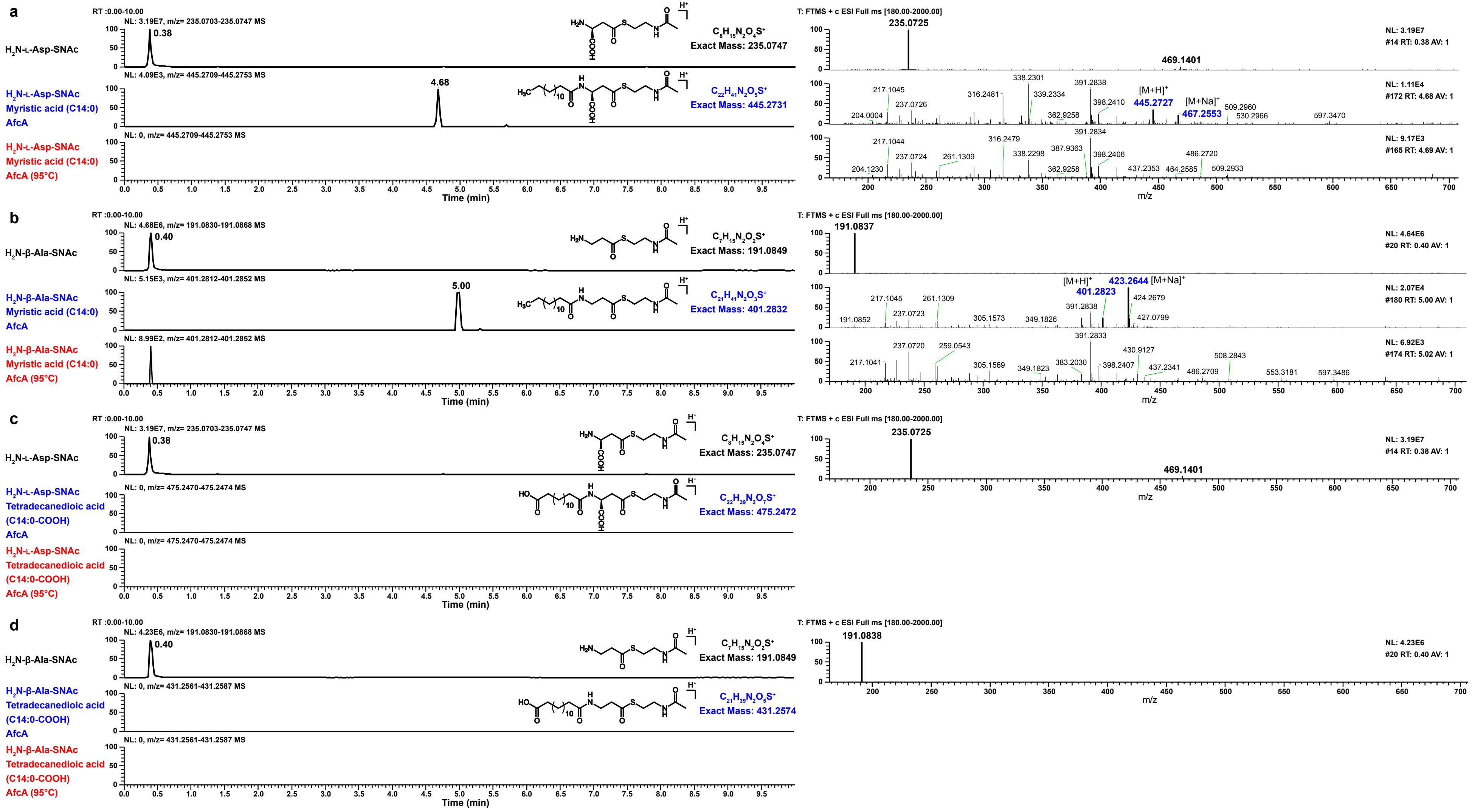

Supplementary Figure 75. EICs of AfcA-catalyzed reactions with SNAc thioesters acquired by LTQ-Orbitrap XL: **a**) myristic acid (C14:0) with H_2N-L-Asp-SNAc, **b**) myristic acid (C14:0) with H_2N-β-Ala-SNAc, **c**) tetradecanedioic acid (C14:0-COOH) with H_2N-L-Asp-SNAc, and **d**) tetradecanedioic acid (C14:0-COOH) with H_2N-β-Ala-SNAc. The anticipated ions are highlighted in blue. AfcA was deactivated at 95 °C for 5 min as negative controls. The formation of expected ions was not observed when tetradecanedioic acid (C14:0-COOH) was tested.

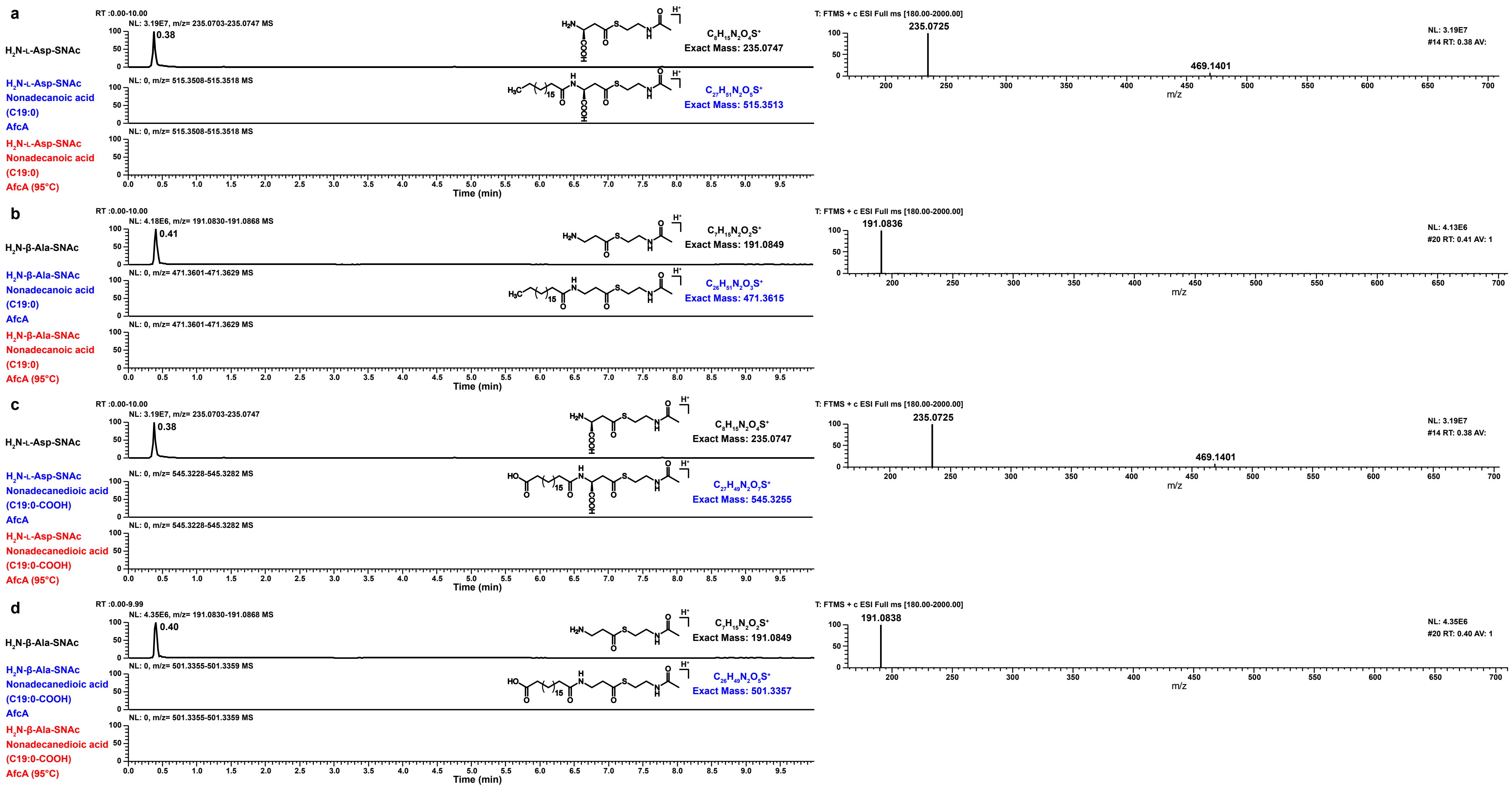

Supplementary Figure 76. EICs of AfcA-catalyzed reactions with SNAc thioesters acquired by LTQ-Orbitrap XL: **a**) nonadecanoic acid (C19:0) with H2N-L-Asp-SNAc, **b**) nonadecanoic acid (C19:0) with H2N-β-Ala-SNAc, **c**) nonadecanedioic acid (C19:0-COOH) with H2N-L-Asp-SNAc, and **d**) nonadecanedioic acid (C19:0-COOH) with H2N-β-Ala-SNAc. AfcA was deactivated at 95 °C for 5 min as negative controls. The formation of all expected ions was not observed.

Supplementary Table 10. Observed peptide bond formation catalyzed by AfcQ and AfcA when using SNAc thioesters as acceptor.

A domain	Substrate	Acceptor	Expected product	Calculated $[M+H]^+$	Observed $[M+H]^+$	Observed $[M+Na]^+$
AfcQ	L-Asp ($^{13}C_4$)	H_2N-L-Asp-SNAc	$C_8{}^{13}C_4H_{20}N_3O_7S^+$	354.1151	354.1153	376.0972
		H_2N-(L-Asp)$_2$-SNAc	$C_{12}{}^{13}C_4H_{25}N_4O_{10}S^+$	469.1420	469.1425	491.1250
		H_2N-(L-Asp)$_3$-SNAc	$C_{16}{}^{13}C_4H_{30}N_5O_{13}S^+$	584.1690	584.1682	N.D.
		H_2N-(L-Asp)$_4$-SNAc	$C_{20}{}^{13}C_4H_{35}N_6O_{16}S^+$	699.1959	699.1963	N.D.
	L-Asp	H_2N-β-Ala-SNAc	$C_{11}H_{20}N_3O_5S^+$	306.1118	306.1126	328.0948
		H_2N-(β-Ala)$_2$-SNAc	$C_{14}H_{25}N_4O_6S^+$	377.1489	377.1488	399.1317
		H_2N-(β-Ala)$_3$-SNAc	$C_{17}H_{30}N_5O_7S^+$	448.1860	448.1859	470.1698
		H_2N-(β-Ala)$_4$-SNAc	$C_{20}H_{35}N_6O_8S^+$	519.2232	519.2260	541.2064
AfcA	Propionic acid (C3:0)	H_2N-L-Asp-SNAc	$C_{11}H_{19}N_2O_5S^+$	291.1009	N.D.[a]	N.D.
		H_2N-β-Ala-SNAc	$C_{10}H_{19}N_2O_3S^+$	247.1111	N.D.	N.D.
	Propanedioic acid (C3:0-COOH)	H_2N-L-Asp-SNAc	$C_{11}H_{17}N_2O_7S^+$	321.0751	N.D.	N.D.
		H_2N-β-Ala-SNAc	$C_{10}H_{17}N_2O_5S^+$	277.0853	N.D.	N.D.
	Tridecanoic acid (C13:0)	H_2N-L-Asp-SNAc	$C_{21}H_{39}N_2O_5S^+$	431.2574	431.2567	453.2392
		H_2N-β-Ala-SNAc	$C_{20}H_{39}N_2O_3S_+$	387.2676	387.2672	409.2483
	Tridecanedioic acid (C13:0-COOH)	H_2N-L-Asp-SNAc	$C_{21}H_{37}N_2O_7S^+$	461.2316	N.D.	N.D.
		H_2N-β-Ala-SNAc	$C_{20}H_{37}N_2O_5S^+$	417.2418	N.D.	N.D.
	Tetradecanoic acid (C14:0, myristic acid)	H_2N-L-Asp-SNAc	$C_{22}H_{41}N_2O_5S^+$	445.2731	445.2735	467.2558
		H_2N-(L-Asp)$_2$-SNAc	$C_{26}H_{46}N_3O_8S^+$	560.3000	N.D.	N.D.
		H_2N-(L-Asp)$_3$-SNAc	$C_{30}H_{51}N_4O_{11}S^+$	675.3270	N.D.	N.D.
		H_2N-(L-Asp)$_4$-SNAc	$C_{34}H_{56}N_5O_{14}S^+$	790.3539	N.D.	N.D.
		H_2N-β-Ala-SNAc	$C_{21}H_{41}N_2O_3S^+$	401.2832	401.2825	423.2640
		H_2N-(β-Ala)$_2$-SNAc	$C_{24}H_{46}N_3O_4S^+$	472.3204	472.3196	494.2997
		H_2N-(β-Ala)$_3$-SNAc	$C_{27}H_{51}N_4O_5S^+$	543.3575	543.3578	565.3381
		H_2N-(β-Ala)$_4$-SNAc	$C_{30}H_{56}N_5O_6S^+$	614.3946	614.3935	636.3755
	Tetradecanedioic acid (C14:0-COOH)	H_2N-L-Asp-SNAc	$C_{22}H_{39}N_2O_7S^+$	475.2472	N.D.	N.D.
		H_2N-β-Ala-SNAc	$C_{21}H_{39}N_2O_5S^+$	431.2574	N.D.	N.D.
	Nonadecanoic acid (C19:0)	H_2N-L-Asp-SNAc	$C_{27}H_{51}N_2O_5S^+$	515.3513	N.D.	N.D.
		H_2N-β-Ala-SNAc	$C_{26}H_{51}N_2O_3S^+$	471.3615	N.D.	N.D.
	Nonadecanedioic acid (C19:0-COOH)	H_2N-L-Asp-SNAc	$C_{27}H_{49}N_2O_7S^+$	545.3255	N.D.	N.D.
		H_2N-β-Ala-SNAc	$C_{26}H_{49}N_2O_5S^+$	501.3357	N.D.	N.D.

[a] Not detected.

Supplementary Table 11. Microorganisms used in this study.

Strain	Origin	Nature of strain	Risk group[a]	Application in this study
		Bacteria		
Burkholderia orbicola Mc0-3	LMG 24308[b]	Gram-negative	2	Production of aimed compounds
Burkholderia puraquae DSM 103137	DSMZ[c]	Gram-negative	1	Production of aimed compounds
Burkholderia pyrrocinia DSM 10685	DSMZ	Gram-negative	1	WT and mutants
Escherichia coli DH5α[d]	Invitrogen	Gram-negative	1	Plasmid preparation
Escherichia coli BL21-Gold (DE3)[e]	Stratagene	Gram-negative	1	Heterologous expression
Escherichia coli DSM 113367	DSMZ	Gram-negative	1	Conjugation
Escherichia coli DSM 1116	DSMZ	Gram-negative	1	Antibacterial assays
Escherichia coli BW25113	DSMZ	Gram-negative	1	Antibacterial assays
Salmonella typhimurium TA100	DSMZ	Gram-negative	1	Antibacterial assays
Bacillus subtilis DSM 10	DSMZ	Gram-positive	1	Antibacterial assays
Micrococcus luteus DSM 1790	DSMZ	Gram-positive	1	Antibacterial assays
Mycobacterium phlei DSM 750	DSMZ	Gram-positive	1	Antibacterial assays
		Fungi		
Colletotrichum kahawae CM732	Cirad-UMR PHIM-Montpellier[136]	Coffee pathogenic	2	Antifungal assays
Pyrenophora teres f. *teres* Hun0005[f]	CBS 123931[g]	Barley pathogenic	2	Antifungal assays
Fusarium xylarioides CAB003	Cirad-UMR PHIM-Montpellier[137]	Coffee pathogenic	2	Antifungal assays
Pyricularia oryzae Guy11[h]	Cirad-UMR PHIM-Montpellier[138]	Rice pathogenic	2	Antifungal assays
Rhizoctonia solani CD9001	Cirad-UMR PHIM-Montpellier[139]	Rice pathogenic	2	Antifungal assays
Aspergillus fumigatus ATCC 205304	NRZMyk[i]	Human pathogenic	2	Antifungal assays
Candida albicans NRZ-2024-0602	NRZMyk	Human pathogenic	2	Antifungal assays
Candida glabrata NRZ-2024-0605[j]	NRZMyk	Human pathogenic	2	Antifungal assays
Candida krusei NRZ-2024-0601[k]	NRZMyk	Human pathogenic	1	Antifungal assays
Candida parapsilosis ATCC 22019	NRZMyk	Human pathogenic	2	Antifungal assays
Fusarium solani NRZ-2024-0590	NRZMyk	Human pathogenic	2	Antifungal assays
Lichtheimia corymbifera NRZ-2024-0649[l]	NRZMyk	Human pathogenic	2	Antifungal assays
Rhizomucor pusillus NRZ-2024-0631[l]	NRZMyk	Human pathogenic	1	Antifungal assays
Scedosporium apiospermum NRZ-2024-0680	NRZMyk	Human pathogenic	2	Antifungal assays

[a] Classification according to German TRBA (*Technische Regel für Biologische Arbeitsstoffe*). [b] LMG = collection of the Laboratory of Microbiology, Department of Biochemistry and Microbiology, Faculty of Sciences of Ghent University. [c] *Deutsche Sammlung von Mikroorganismen und Zellkulturen* (German Collection of Microorganisms and Cell Cultures). [d] Supplier: Thermo Fisher Scientific. [e] Supplier: Agilent Technologies. [f] Formerly *Helminthosporium teres*. [g] CBS = collection of the Westerdijk Fungal Biodiversity Institute. [h] Syn. *Magnaporthe oryzae* Guy11. [i] *Nationale Referenzzentrum für Invasive Pilzinfektionen* (National Reference Center for Invasive Fungal Infections), Leibniz Institute for Natural Product Research and Infection Biology, Hans-Knöll-Institute, Jena, Germany. [j] Syn. *Nakaseomyces glabratus*. [k] Syn. *Pichia kudriavzevii*. [l] Mucorales.

Supplementary Table 12. Media used in this study.

Medium	Ingredients (L^{-1})	Application in this study
PDA	39 g of potato dextrose agar (BD, Difco™), sterilization at 121 °C for 20 min	Cultivation of *Burkholderia* strains
PDB	24 g of potato dextrose broth (BD, Difco™), sterilization at 121 °C for 20 min	Cultivation of *Burkholderia* strains and antifungal assays
LB	10 g of tryptone (Roth), 5 g of yeast extract (BD, Bacto™), 5 g of NaCl, sterilization at 121 °C for 20 min	Preculture
LB Agar	LB medium + 15 g of Agar (Roth), sterilization at 121 °C for 20 min	Streaking/Spreading
TB	12 g of tryptone (Roth), 24 g of yeast extract (BD, Bacto™), 4 mL of glycerol, sterilization at 121 °C for 20 min, then 10 mL of 1 M $MgCl_2$ and 100 mL of sterile TB buffer 10x (170 mM KH_2PO_4, 720 mM K_2HPO_4)	Heterologous expression
MHBII	22 g of Mueller Hinton II Broth (cation-adjusted) (BD, BBL™), final pH 7.3, sterilization at 121 °C for 20 min	Antibacterial assays
M9	200 mL of sterile M9 stock solution 5x (64 g/L $Na_2HPO_4*2H_2O$, 15 g/L KH_2PO_4, 5.0 g/L NH_4Cl, 2.5 g/L NaCl), 100 µL of 1 M $CaCl_2$, 2 mL of 1 M $MgSO_4$, 100 µL of 1 mg/mL $FeSO_4$, 20 mL of glucose 20% (w/v), sterilization at 121 °C for 20 min	Conjugation

Supplementary Table 13. Buffers used in this study.

Buffer	Ingredients	Application in this study
Lysis buffer	500 mM NaCl, 50 mM Tris-HCl pH 8.0, 20 mM imidazole	Purification of Afc proteins (general)
Wash buffer	500 mM NaCl, 50 mM Tris-HCl pH 8.0, 20 mM imidazole	
Elution buffer	500 mM NaCl, 50 mM Tris-HCl pH 8.0, 250 mM imidazole	
Protein buffer	150 mM NaCl, 20 mM Tris-HCl pH 8.0	
Lysis buffer	300 mM NaCl, 50 mM Tris-HCl pH 8.0, 20 mM imidazole	Purification of 4'-phosphopantetheinyl transferase Sfp
Wash buffer	300 mM NaCl, 50 mM Tris-HCl pH 8.0, 20 mM imidazole	
Elution buffer	300 mM NaCl, 50 mM Tris-HCl pH 8.0, 500 mM imidazole	
Protein buffer	200 mM NaCl, 50 mM Tris-HCl pH 8.0	
HEPES buffer 1x	50 mM NaCl, 10 mM $MgCl_2$, 50 mM HEPES pH 7.0	*In vitro* enzymatic assays
Adenylation buffer 2x	300 mM NaCl, 10 mM $MgCl_2$, 40 mM Tris-HCl pH 8.0	
Adenylation buffer 1x	150 mM NaCl, 5 mM $MgCl_2$, 20 mM Tris-HCl pH 8.0	

Supplementary Table 14. Carboxylic acids used in this study for substrate specificity assays.

**Amino acids and analogues of citric acids (in total 28 compounds)**
L-Ala, D-Ala, β-Ala, L-Arg, L-Asn, D-Asn, L-Asp, D-Asp, L-Cys, L-Glu, L-Gln, Gly, L-His, L-Ile, L-Leu, L-Lys, D-Lys, L-Met, L-Phe, L-Pro, L-Ser, L-Thr, L-Trp, L-Tyr, L-Val, citric acid, oxalacetic acid, L/D-malic acid
**Fatty acids (in total 34 compounds)**
Propionic acid (C3:0)[a], Propanedioic acid (C3:0-COOH)[b], Butyric acid (C4:0), Succinic acid (C4:0-COOH), Pentanoic acid (C5:0), Pentanedioic acid (C5:0-COOH), Hexanoic acid (C6:0), Hexanedioic acid (C6:0-COOH), Heptanoic acid (C7:0), Heptanedioic acid (C7:0-COOH), Octanoic acid (C8:0), Octanedioic acid (C8:0-COOH), Nonanoic acid (C9:0), Nonanedioic acid (C9:0-COOH), Decanoic acid (C10:0), Decanedioic acid (C10:0-COOH), Undecanoic acid (C11:0), Undecanedioic acid (C11:0-COOH), Dodecanoic acid (C12:0), Dodecanedioic acid (C12:0-COOH), Tridecanoic acid (C13:0), Tridecanedioic acid (C13:0-COOH), Tetradecanoic acid (C14:0, myristic acid), Tetradecanedioic acid (C14:0-COOH), Pentadecanoic acid (C15:0), Pentadecanedioic acid (C15:0-COOH), Hexadecanoic acid (C16:0), Hexadecanedioic acid (C16:0-COOH), Heptadecanoic acid (C17:0), Heptadecanedioic acid (C17:0-COOH), Stearic acid (C18:0), Octadecanedioic acid (C18:0-COOH), Nonadecanoic acid (C19:0), Nonadecanedioic acid (C19:0-COOH)

[a] The monocarboxylic acids utilized for AfcA-related assays are fully-saturated unbranched fatty acids. [b] The dicarboxylic acids used in this study are fully-saturated fatty acids bearing a second carboxylic group at the ω-position.

Supplementary Table 15. Acquisition parameters for NMR measurements in this study.

Compound	Experiment	TD (F2/F1)	DS	NS	SW (ppm) (F2/F1)	O1P (ppm) (F2/F1)	Mixing time (sec)	Irradiation frequency (ppm)
**AFC-BC11**[a]	^{1}H	65536	2	256	13	6	/	/
	^{1}H-^{1}H COSY	4096/512	16	16	13/13	6/6	/	/
	^{1}H-^{1}H TOCSY	4096/512	16	16	13/13	6/6	0.1	/
	^{1}H-^{1}H NOESY	4096/512	16	64	13/13	6/6	0.6	/
	^{1}H-^{13}C HSQC	4096/256	16	16	13/236	6/100	/	/
	^{1}H-^{13}C HMBC	4096/512	16	32	13/236	6/100	/	/
	^{1}H-^{13}C HMBC	2048/256	16	1024	12/19	6/170	/	/
	^{1}H-^{15}N SOFAST-HMQC	2048/64	16	512	12/35	6/117	/	/
	^{1}H-^{15}N SOFAST-HMQC	2048/64	16	1024	12/35	6/120	/	/
	homo-decoupled ^{1}H	65536	2	128	13	6	/	2.50
	homo-decoupled ^{1}H	65536	2	128	13	6	/	3.43
**Photoisomer 5**[a]	^{1}H	48828	16	128	30	6	/	/
	^{1}H-^{1}H COSY	4096/256	16	8	12/12	6/6	/	/
	^{1}H-^{13}C HSQC	2048/256	16	72	14/150	6/75	/	/
	^{1}H-^{1}H NOESY	2048/512	16	32	12/12	6/6	0.6	/
**SNAcs (1-8, 12)**[b]	^{1}H	65536	2	8	20	6	/	/
**H_2N-(β-Ala)$_1$-*S*-CoA (9)**[b]	^{1}H	65536	2	64	20	6	/	/
**H_2N-(β-Ala)$_2$-*S*-CoA (10)**[c]	^{1}H	65536	2	64	20	6	/	/
**H_2N-(β-Ala)$_3$-*S*-CoA (11)**[c]	^{1}H	65536	2	64	12	6	/	/
**H_2N-L-Lys-*S*-CoA (13)**[c]	^{1}H	65536	2	64	20	6	/	/

[a] 700 MHz, DMSO-d_6, and 298 K. [b] 500 MHz, D_2O, and 298 K. [c] 700 MHz, D_2O, and 298 K.

Supplementary Table 16. PCR primers used to clone plasmids for heterologous expression.

Construct	Forward primer (5'- 3')	Reverse primer (5'- 3')
AfcQ	CTTGTATTTCCAGGGCCATATGAACGCATTGATCAGCATC	GCTTTGTTAGCAGCCGGATCTCATGCGGCGGTCTCCTCG
AfcA	CTTGTATTTCCAGGGCCATATGACGAAGTACGCATCCAC	GCTTTGTTAGCAGCCGGATCTCATGCGAACGCTCCCTGGAC
AfcK	CTTGTATTTCCAGGGCCATATGACGACCCAGAACGTTCC	GCTTTGTTAGCAGCCGGATCTCAGGCCGCGTGCTGCTGGC
AfcP	ACTTGTATTTCCAGGGCCATATGACGCCTGAGCGCCCGC	GCTTTGTTAGCAGCCGGATCCTAGGCGGGCCGCACGTACAC
AfcL	CTTGTATTTCCAGGGCCATATGCTCGCGCCCCTTGCCCC	GCTTTGTTAGCAGCCGGATCTCACGCCTCGCCGTCGAGAG
pET28a_TEV	ATGGCCCTGGAAATACAAGTTTTCG	GATCCGGCTGCTAACAAAGCC

Supplementary Table 17. PCR primers used to clone constructs for in frame deletion mutants (up = primer for upstream region of target gene; down = primer for downstream region of target gene).

Mutant	Forward primer (5'- 3')	Reverse primer (5'- 3')
Δ*afcC*	up: ACAGCTATGACCATGATTATGAAGAGCCGCCCGAGCGG<br>down: CCAGTTCGCGAAGAACGTCGGCACACGTGGTTCGTCGTG	up: CGACGTTCTTCGCGAACTGGATATAGTGCGCGGCCATCG<br>down: GATTAAGTTGGGTAACGCCTCATGCCGACTGCGTGGACG
Δ*afcD*	up: ACAGCTATGACCATGATTGTGACGACGATGCTCTATCC<br>down: GAAATGGTGCCGACCGAAGCGCACCGAGAAGCCGCTGCAC	up: ATTAAGTTGGGTAACGCCCTGGAAGCGCACCGCGTG<br>down: GATGTGCTGCAAGGCGATTATTACACGCCGTCCACCGCC
Δ*afcE*	up: GAAACAGCTATGACCATGATTATGAGCGCCTACAAGGTGAG<br>down: CCGCTCGAGGCGTACCGCTCGCGACGTGAAGATCCTGTCG	up: AGCGGTACGCCTCGAGCGGTGCAATCAGCGCTTTCTGG<br>down: CGATTAAGTTGGGTAACGCCTCATTCCACCTGCGCGAGTTC
Δ*afcF*	up: CAGCTATGACCATGATTATGAACGCGCTGCCCGAAC<br>down: CGTGATCGACACGCTCGCGATCGCGACCGGCATGCC	up: GGCATGCCGGTCGCGATCGCGAGCGTGTCGATCACG<br>down: GATTAAGTTGGGTAACGCCTCAATAGCGGACACGTCCG
Δ*afcI*	up: GAAACAGCTATGACCATGATTATGGCCAGTGACAACGAGGC<br>down: TCTACGATCGCTACGGCGACTACGACGCGGTCGATCGC	up: TCGCCGTAGCGATCGTAGATATCCGCCGTC<br>down: GATTAAGTTGGGTAACGCCTCAGAAGCGCACGCGCACGG
Δ*afcJ*	up: GAAACAGCTATGACCATGATTATGTCCACGCTTTCCGTCC<br>down: CTTCTCCGACGACCAGAACCTGCAGCTGGCGAACGTCG	up: TTCTGGTCGTCGGAGAAGAACAGCCCGAACATGATGC<br>down: CGATTAAGTTGGGTAACGCCTCAACTGCCGGCTTCGCAGC
Δ*afcL*	up: GAAACAGCTATGACCATGATTATGCTCGCGCCTCTTGCC<br>down: GGATGACCTGCTCGTACGAGCCGCGAACCGGCGCAGTC	up: TCGTACGAGCAGGTCATCCAGTCGGGCGTGTAGTCGTTCG<br>down: CGATTAAGTTGGGTAACGCCTCACGCCTCGCCTTCGAGAG
Δ*afcM*	up: GAAACAGCTATGACCATGATTATGGTGCGACCCGTTCAC<br>down: GCATTCCTCGCCGAAGCCACCCGTCGTGTGGATCGGCGAC	up: GGTGGCTTCGGCGAGGAATGCGAAATACTGGACGTGGTCG<br>down: CGATTAAGTTGGGTAACGCCTCAAGGTCTCCCCGGCCGC
Δ*afcN*	up: CAGCTATGACCATGATTATGACTCAAACGCTCACCG<br>down: GTCGAAACTCGCGTCCGCCGCATCAAGGACCTGTTCATGC	up: GCATGAACAGGTCCTTGATGCGGCGGACGCGAGTTTCGAC<br>down: GATTAAGTTGGGTAACGCCTCAGTTGAGCGGCATCGCCG
Δ*afcO*	up: CAGCTATGACCATGATTATGACGATGCCCTCCTCC<br>down: CTGCCGCACGAACTCGCCGCCGACCTGATCATGATGG	up: CCATCATGATCAGGTCGGCGGCGAGTTCGTGCGGCAG<br>down: GATTAAGTTGGGTAACGCCTCAGGCGTCATGGCGACC
Δ*afcP*	up: CAGCTATGACCATGATTATGACGCCTGAACGCCCGC<br>down: GTACTGCTGCGCCTGAACGCTATGCGACCGCGCTCGCG	up: CGCGAGCGCGGTCGCATAGCGTTCAGGCGCAGCAGTAC<br>down: GATTAAGTTGGGTAACGCCTCAGGCAGGCCGCACGTAC
Δ*afcS*	up: CAGCTATGACCATGATTATGGACACGACACTCGATTC<br>down: CTGTTGCGTGGTCTCGATGAGATCGATGCGGCGCTCGACG	up: GATTAAGTTGGGTAACGCCATGCGCGCCTGTACGTCATC<br>down: GATGTGCTGCAAGGCGATTATCACAGCCAGCCGACCGGC
Δ*afcT*	up: CAGCTATGACCATGATTATGCTGCTCAAGAACCTGCG<br>down: GACCTCGATCCGGCGATGCTCGCGCTCGTGCACGACATGC	up: CGATTAAGTTGGGTAACGCCGCCTTCGTCGAGCATCGCCG<br>down: GATTAAGTTGGGTAACGCCGTCAGGCCAGCGCCGGTTTCT
Δ*shvR*	up: CAGCTATGACCATGATTATGGCTAATGTGAGATTGGC<br>down: GTCGAGCGTGGGCCAGCGCCTGAAGGAGCTCGGCGTGC	up: GCACGCCGAGCTCCTTCAGGCGCTGGCCCACGCTCGAC<br>down: GATTAAGTTGGGTAACGCCCTATCCGACGCGATACAGCG
KmR	GCAATCAGCGCGACCTTGCTACGGCTACACTAGAAGGAC	GTCATCAGCGGTGGAGTGCATTAGAAAAACTCATCGAGCA
p18mobapra	TGCACTCCACCGCTGATGAC	CAAGGTCGCGCTGATTGCTG
TpR	GACTACCTTGGTGATCTCGCGGTCTGACGCTCAGTGGAAC	CGACTGATGTCATCAGCGGTGGCAGCACTGCATAATTCTC
p18mobKmR	ACCGCTGATGACATCAGTCG	GCGAGATCACCAAGGTAGTC

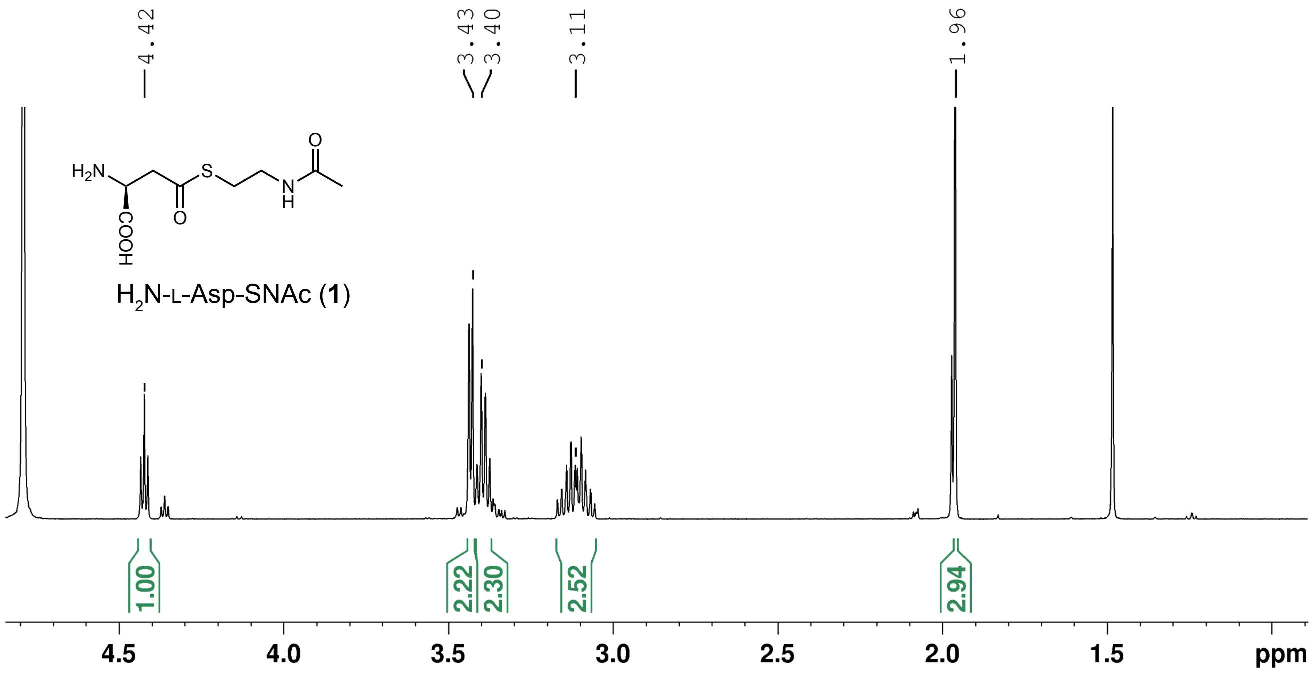

Supplementary Figure 77. ^{1}H-NMR spectrum of synthetic H_2N-L-Asp-SNAc (**1**).

Molecular formula: $C_8H_{14}N_2O_4S$
^{1}H-NMR (500 MHz, D_2O): δ = 4.42 (t, J_{HH} = 5.4 Hz, 1H), 3.43 (d, J_{HH} = 5.4 Hz, 2H), 3.40 (m, 2H), 3.11 (m, 2H), 1.96 ppm (s, 3H).
HRMS (ESI, *m/z*): calculated $[M+H]^+$ 235.0747, observed 235.0745.

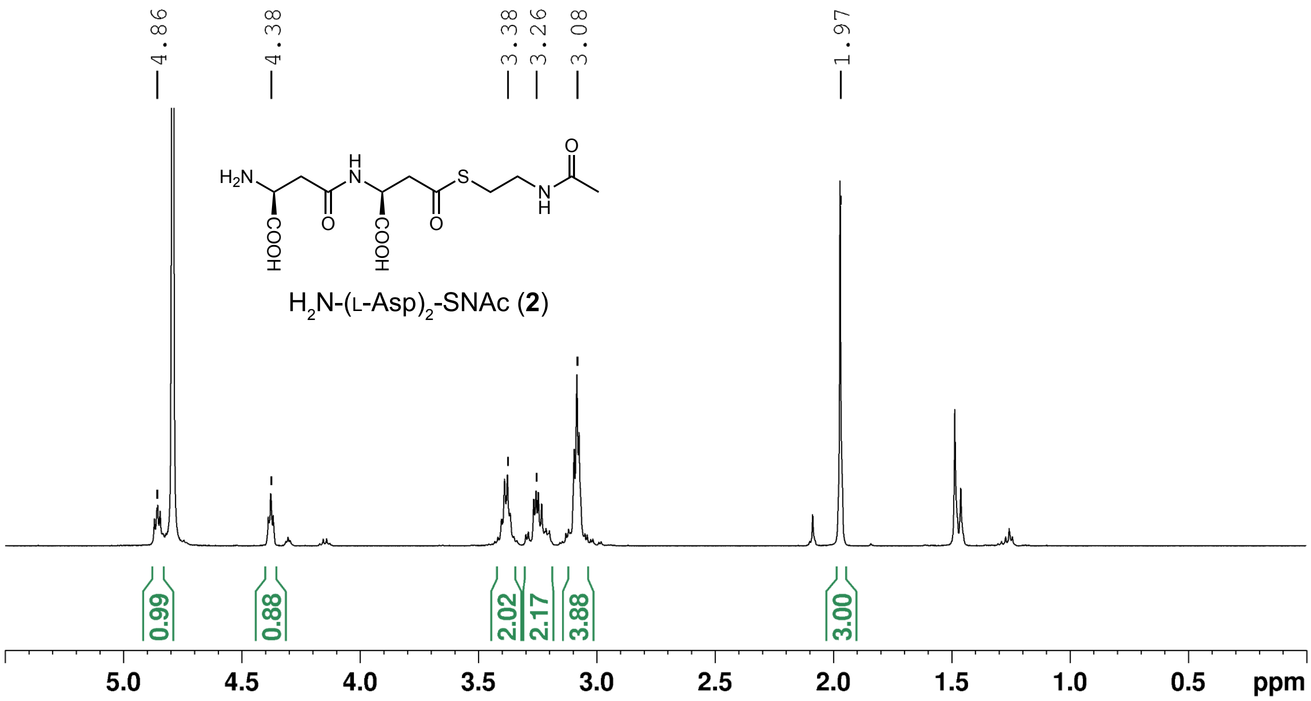

Supplementary Figure 78. ^{1}H-NMR spectrum of synthetic H_2N-(L-Asp)$_2$-SNAc (**2**).

Molecular formula: $C_{12}H_{19}N_3O_7S$
^{1}H-NMR (500 MHz, D_2O): δ = 4.86 (m, 1H), 4.38 (m, 1H), 3.38 (m, 2H), 3.26 (ol, 2H), 3.08 (ol, 4H), 1.97 ppm (s, 3H).
HRMS (ESI, *m/z*): calculated $[M+H]^+$ 350.1016, observed 350.1017.

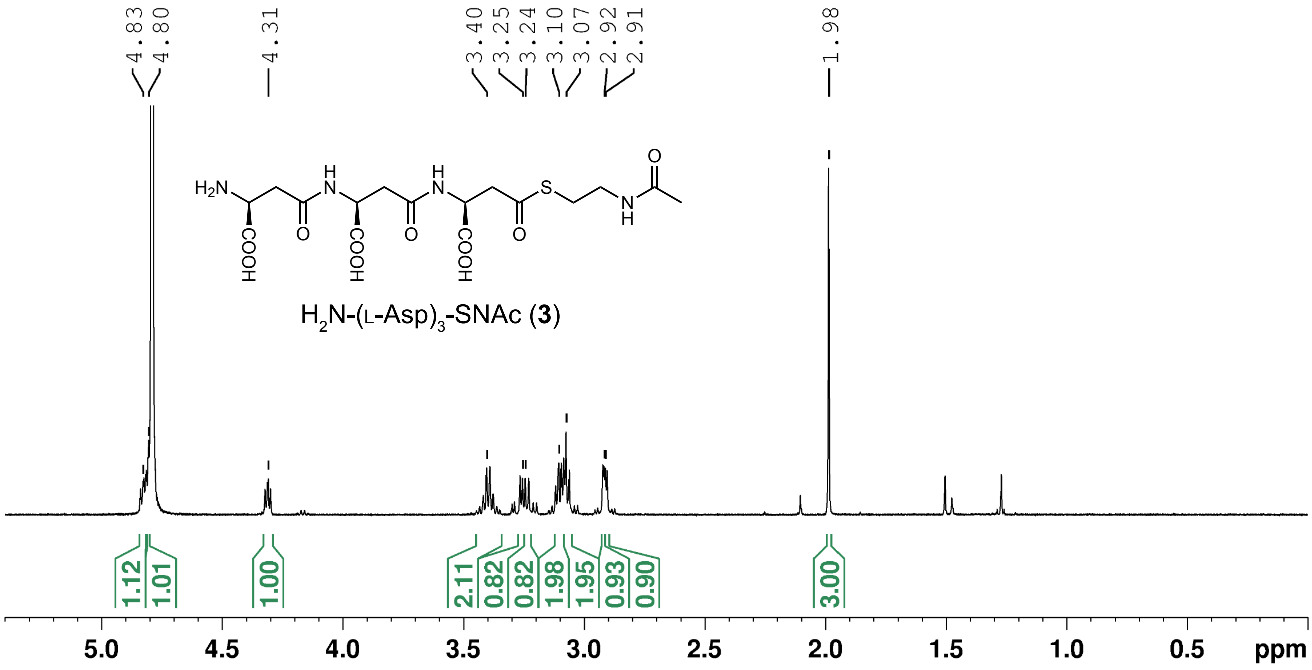

Supplementary Figure 79. ^{1}H-NMR spectrum of synthetic H_2N-(L-Asp)$_3$-SNAc (**3**).

Molecular formula: $C_{16}H_{24}N_4O_{10}S$
^{1}**H-NMR** (500 MHz, D_2O): δ = 4.83 (dd, J_{HH} = 7.2, 5.2 Hz, 1H), 4.80 (dd, J_{HH} = 7.2, 5.2 Hz, 1H), 4.31 (dd, J_{HH} = 6.4, 4.7 Hz, 1H), 3.40 (m, 2H), 3.25 (dd, J_{HH} = 11.2, 5.0 Hz, 1H), 3.24 (dd, J_{HH} = 9.0, 7.2 Hz, 1H), 3.10 (m, 2H), 3.07 (ol, 2H), 2.92 (dd, J_{HH} = 10.4, 5.4 Hz, 1H), 2.91 (dd, J_{HH} = 9.2, 6.4 Hz, 1H), 1.98 ppm (s, 3H).
HRMS (ESI, *m/z*): calculated $[M+H]^+$ 465.1286, observed 465.1284.

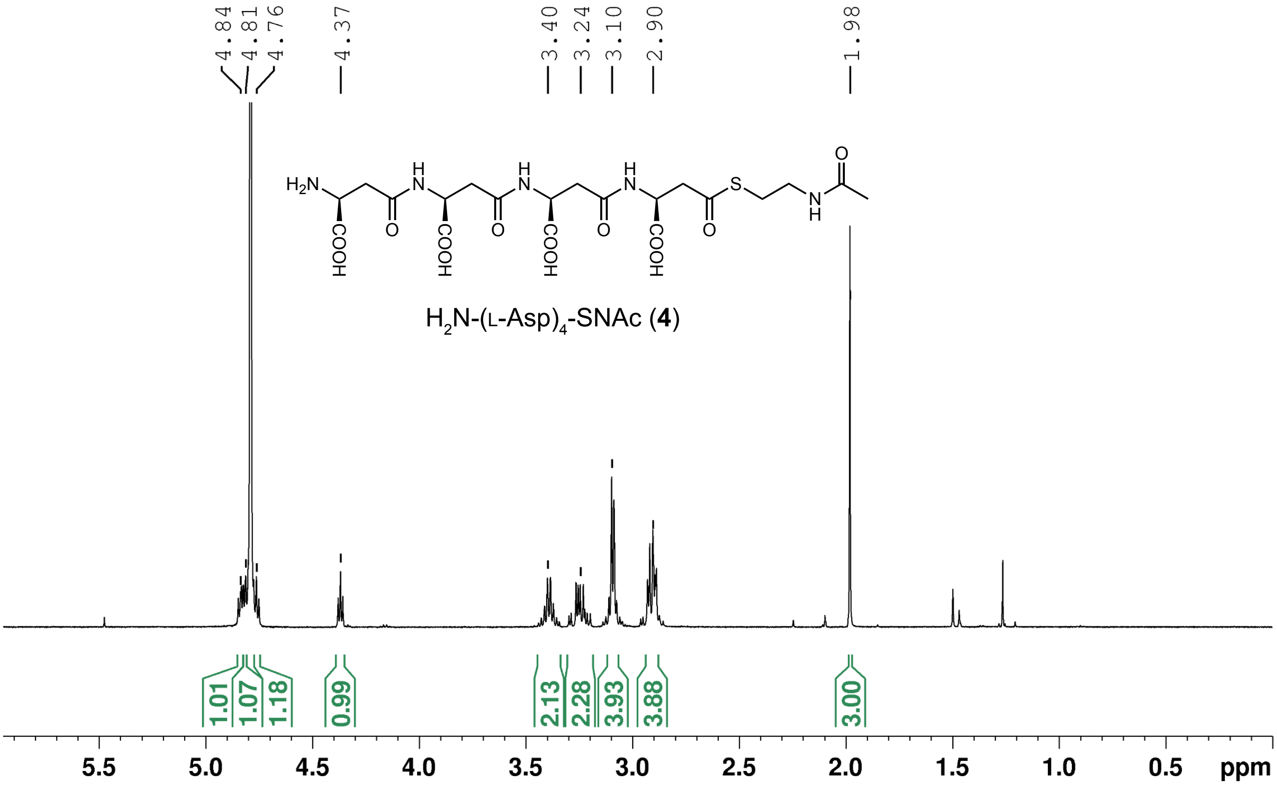

Supplementary Figure 80. ^{1}H-NMR spectrum of synthetic H_2N-(L-Asp)$_4$-SNAc (**4**).

Molecular formula: $C_{20}H_{29}N_5O_{13}S$
^{1}**H-NMR** (500 MHz, D_2O): δ = 4.84 (dd, J_{HH} = 7.1, 5.0 Hz, 1H), 4.81 (dd, J_{HH} = 6.7, 5.4 Hz, 1H), 4.76 (dd, J_{HH} = 6.6, 5.8 Hz, 1H), 4.37 (dd, J_{HH} = 5.9, 5.2 Hz, 1H), 3.40 (m, 2H), 3.24 (ol, 2H), 3.10 (ol, 4H), 2.90 (ol, 4H), 1.98 ppm (s, 3H).
HRMS (ESI, *m/z*): calculated $[M+H]^+$ 580.1555, observed 580.1550.

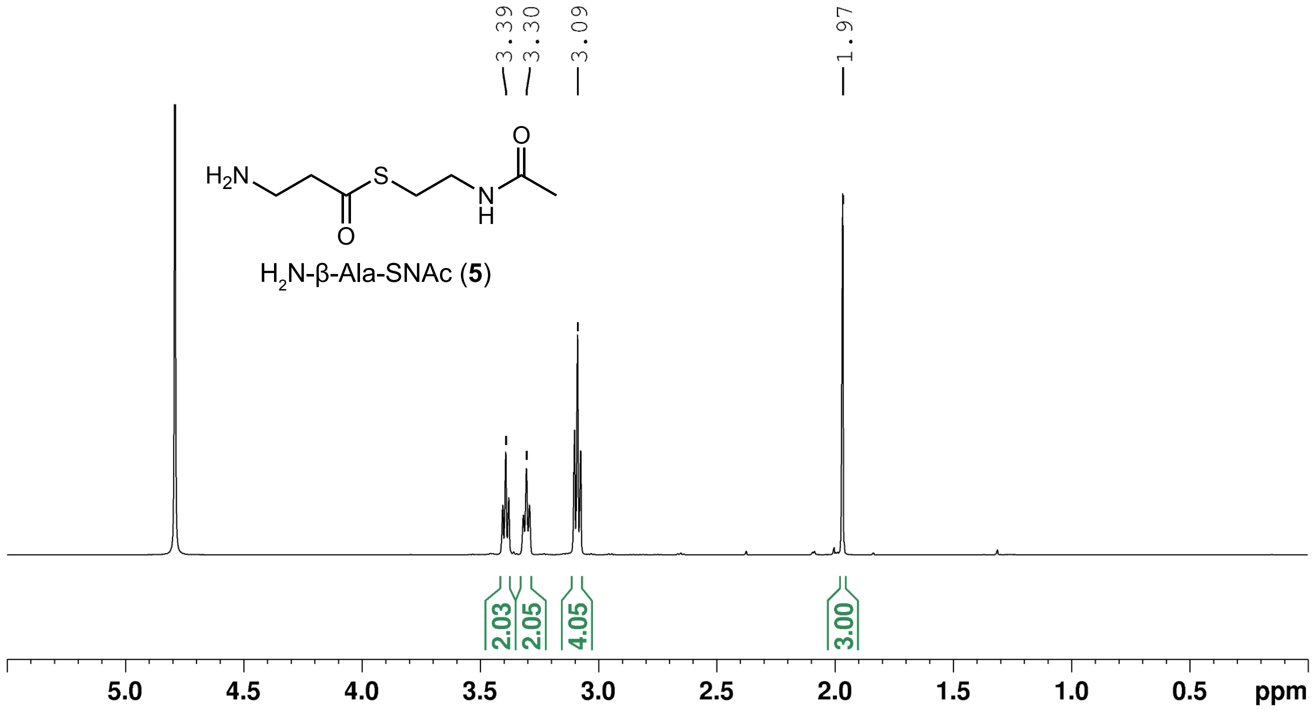

Supplementary Figure 81. ^{1}H-NMR spectrum of synthetic H_2N-β-Ala-SNAc (**5**).

Molecular formula: $C_7H_{14}N_2O_2S$
^{1}H-NMR (500 MHz, D_2O): δ = 3.39 (t, J_{HH} = 6.4 Hz, 2H), 3.30 (t, J_{HH} = 6.6 Hz, 2H), 3.09 (t, J_{HH} = 6.5 Hz, 4H), 1.97 ppm (s, 3H).
HRMS (ESI, *m/z*): calculated $[M+H]^+$ 191.0849, observed 191.0844.

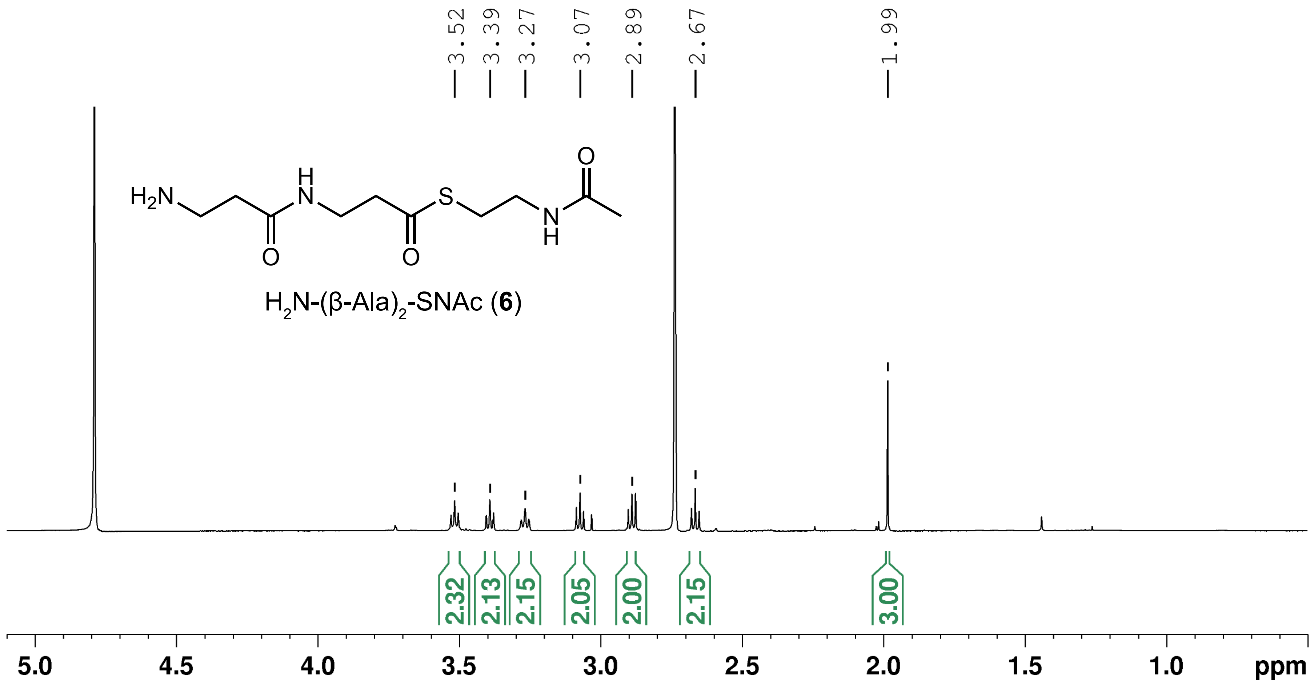

Supplementary Figure 82. ^{1}H-NMR spectrum of synthetic H_2N-(β-Ala)$_2$-SNAc (**6**).

Molecular formula: $C_{10}H_{19}N_3O_3S$
^{1}H-NMR (500 MHz, D_2O): δ = 3.52 (t, J_{HH} = 6.4 Hz, 2H), 3.39 (t, J_{HH} = 6.3 Hz, 2H), 3.27 (t, J_{HH} = 6.8 Hz, 2H), 3.07 (t, J_{HH} = 6.5 Hz, 2H), 2.89 (t, J_{HH} = 6.5 Hz, 2H), 2.67 (t, J_{HH} = 6.8 Hz, 2H), 1.99 ppm (s, 3H).
HRMS (ESI, *m/z*): calculated $[M+H]^+$ 262.1220, observed 262.1216.

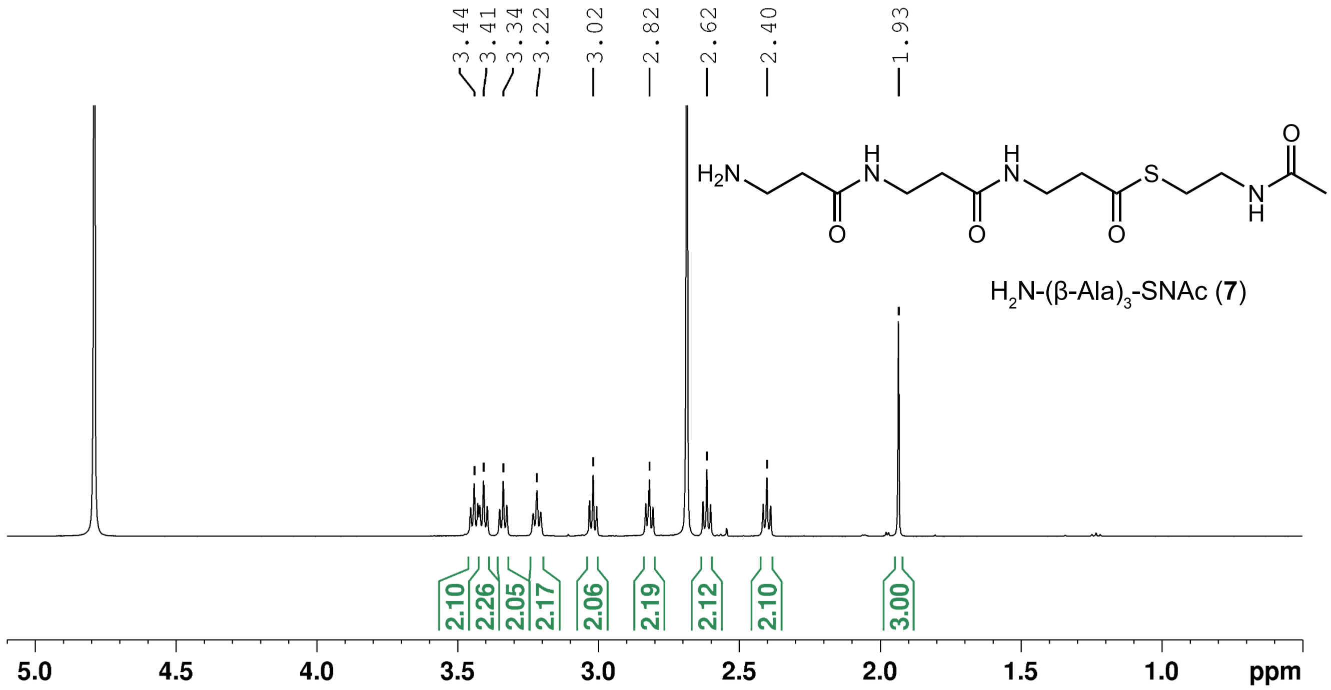

Supplementary Figure 83. ^{1}H-NMR spectrum of synthetic H_2N-(β-Ala)$_3$-SNAc (**7**).

Molecular formula: $C_{13}H_{24}N_4O_4S$
^{1}H-NMR (500 MHz, D_2O): δ = 3.44 (t, J_{HH} = 6.3 Hz, 2H), 3.41 (t, J_{HH} = 6.7 Hz, 2H), 3.34 (t, J_{HH} = 6.4 Hz, 2H), 3.22 (t, J_{HH} = 6.7 Hz, 2H), 3.02 (t, J_{HH} = 6.5 Hz, 2H), 2.82 (t, J_{HH} = 6.5 Hz, 2H), 2.62 (t, J_{HH} = 6.7 Hz, 2H), 2.40 (t, J_{HH} = 6.7 Hz, 2H), 1.93 ppm (s, 3H).
HRMS (ESI, *m/z*): calculated $[M+H]^+$ 333.1591, observed 333.1586.

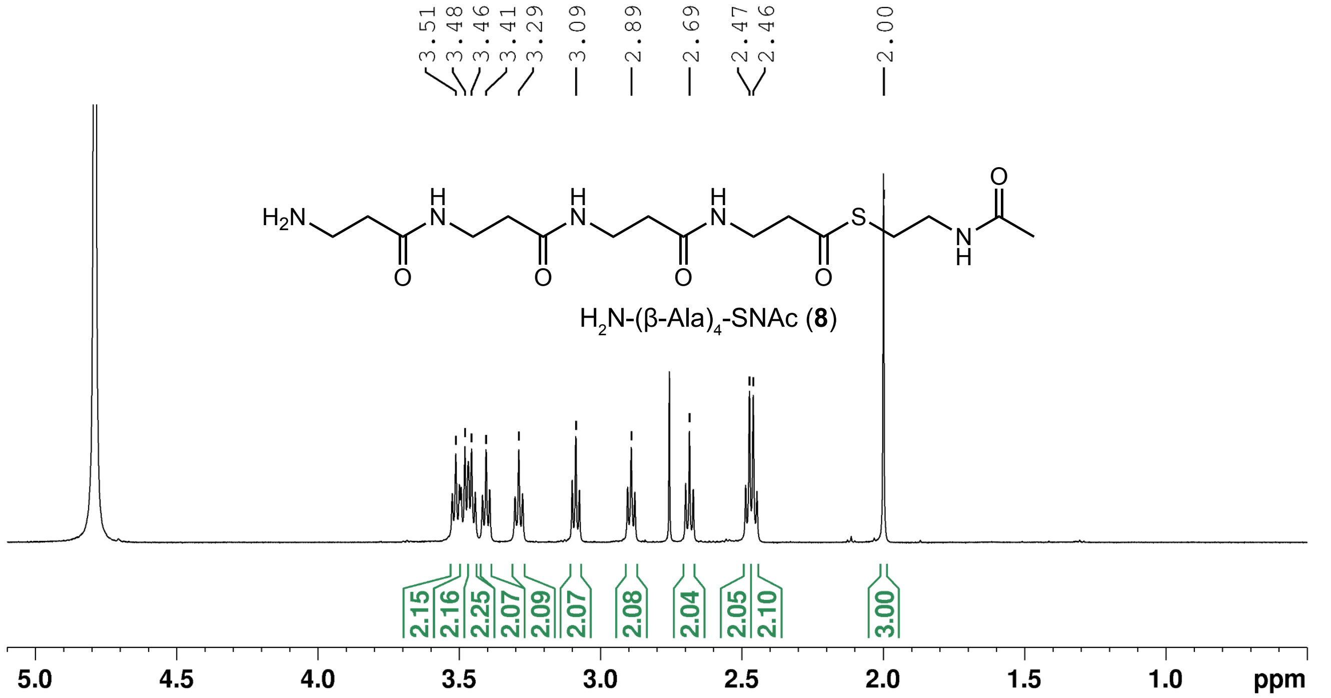

Supplementary Figure 84. ^{1}H-NMR spectrum of synthetic H_2N-(β-Ala)$_4$-SNAc (**8**).

Molecular formula: $C_{16}H_{29}N_5O_5S$
^{1}H-NMR (500 MHz, D_2O): δ = 3.51 (t, J_{HH} = 6.3 Hz, 2H), 3.48 (t, J_{HH} = 6.8 Hz, 2H), 3.46 (t, J_{HH} = 6.6 Hz, 2H), 3.41 (t, J_{HH} = 6.3 Hz, 2H), 3.29 (t, J_{HH} = 6.7 Hz, 2H), 3.09 (t, J_{HH} = 6.4 Hz, 2H), 2.89 (t, J_{HH} = 6.4 Hz, 2H), 2.69 (t, J_{HH} = 6.7 Hz, 2H), 2.47 (t, J_{HH} = 6.7 Hz, 2H), 2.46 (t, J_{HH} = 6.7 Hz, 2H), 2.00 ppm (s, 3H).
HRMS (ESI, *m/z*): calculated $[M+H]^+$ 404.1962, observed 404.1958.

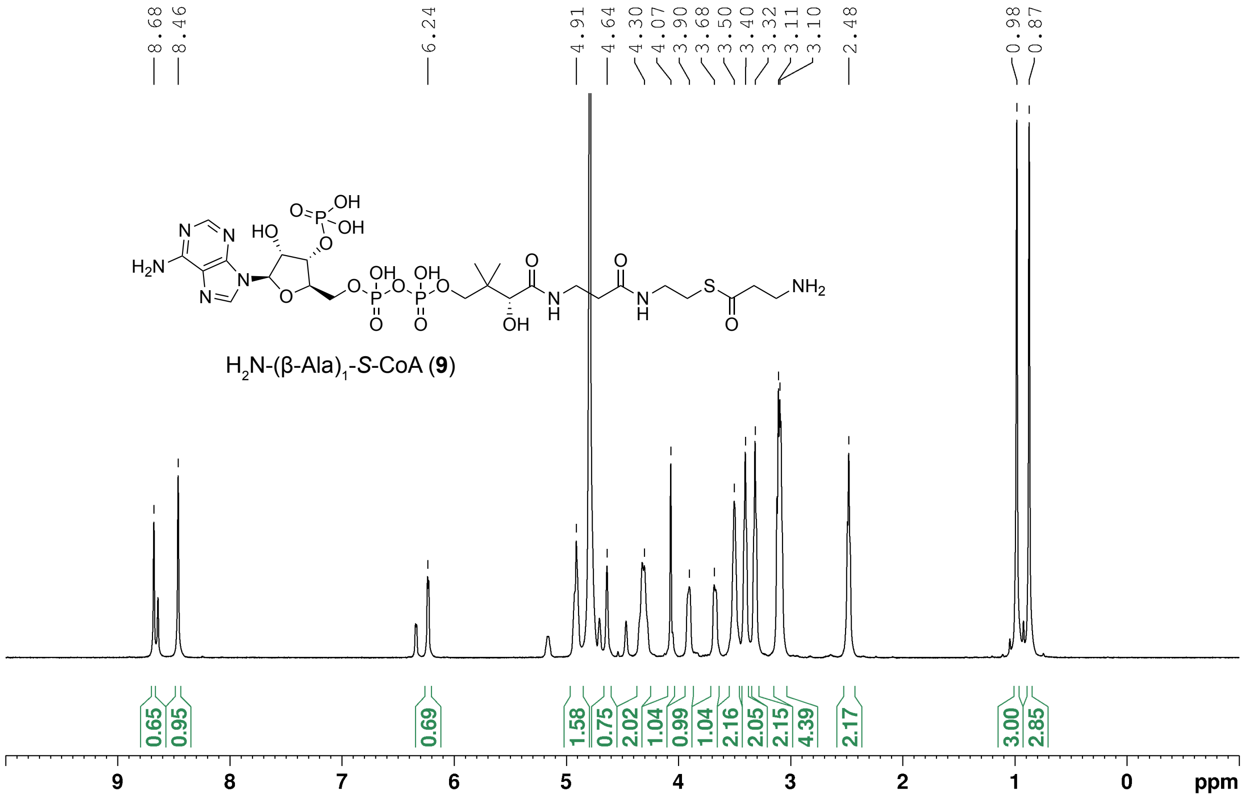

Supplementary Figure 85. ^{1}H-NMR spectrum of synthetic H_2N-(β-Ala)$_1$-*S*-CoA (**9**).

Molecular formula: $C_{24}H_{41}N_8O_{17}P_3S$
^{1}H-NMR (500 MHz, D_2O): δ = 8.68 (s, 1H), 8.46 (s, 1H), 6.24 (d, J_{HH} = 5.2 Hz, 1H), 4.91 (ol, 2H), 4.64 (br, 1H), 4.30 (m, 2H), 4.07 (s, 1H), 3.90 (m, 1H), 3.68 (d, J_{HH} = 9.5 Hz, 1H), 3.50 (m, 2H), 3.40 (m, 2H), 3.32 (m, 2H), 3.11 (ol, 2H), 3.10 (ol, 2H), 2.48 (m, 2H), 0.98 (s, 3H), 0.87 (s, 3H).
HRMS (ESI, *m/z*): calculated $[M+H]^+$ 839.1596, observed 839.1599 (420.0838 for $[M+2H]^{2+}$).

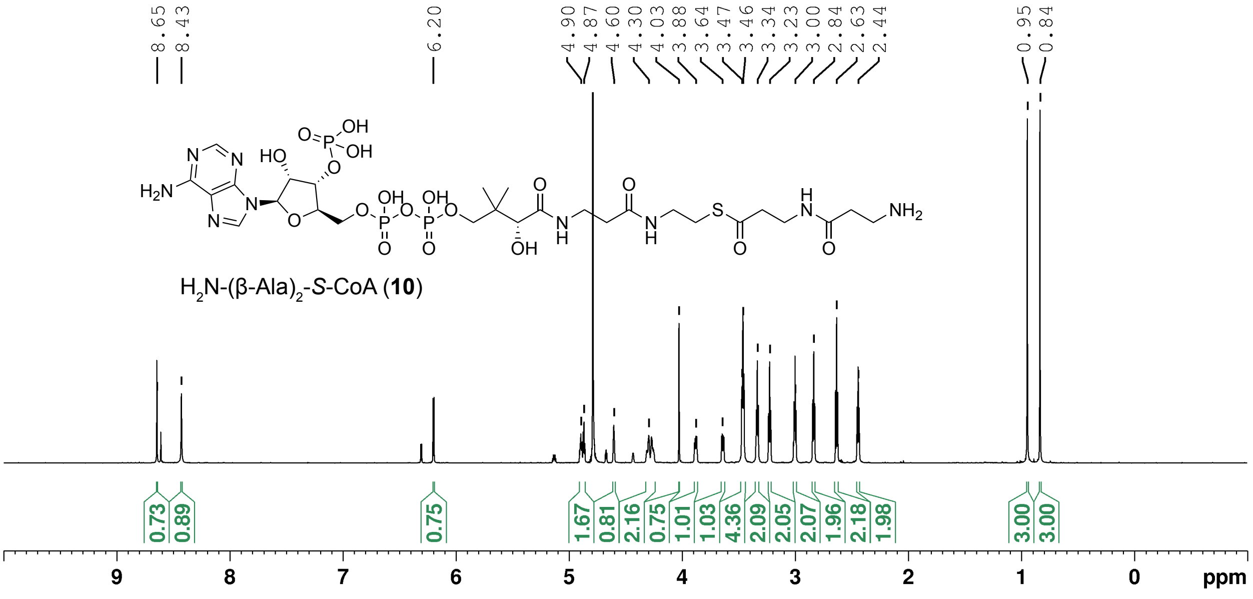

Supplementary Figure 86. ^{1}H-NMR spectrum of synthetic H_2N-(β-Ala)$_2$-*S*-CoA (**10**).

Molecular formula: $C_{27}H_{46}N_9O_{18}P_3S$
^{1}H-NMR (700 MHz, D_2O): δ = 8.65 (s, 1H), 8.43 (s, 1H), 6.20 (d, J_{HH} = 5.9 Hz, 1H), 4.90 (m, 1H), 4.87 (m, 1H), 4.60 (br, 1H), 4.30 (m, 2H), 4.03 (s, 1H), 3.88 (dd, J_{HH} = 9.7 Hz, J_{HH} = 4.5 Hz, 1H), 3.64 (dd, J_{HH} = 9.8 Hz, J_{HH} = 4.6 Hz, 1H), 3.47 (t, J_{HH} = 6.5 Hz, 2H), 3.46 (t, J_{HH} = 6.2 Hz, 2H), 3.34 (t, J_{HH} = 6.4 Hz, 2H), 3.23 (t, J_{HH} = 6.8 Hz, 2H), 3.00 (t, J_{HH} = 6.3 Hz, 2H), 2.84 (t, J_{HH} = 6.4 Hz, 2H), 2.63 (t, J_{HH} = 6.7 Hz, 2H), 2.44 (t, J_{HH} = 6.4 Hz, 2H), 0.95 (s, 3H), 0.84 (s, 3H).
HRMS (ESI, *m/z*): calculated $[M+H]^+$ 910.1967, observed 910.1968 (455.6021 for $[M+2H]^{2+}$).

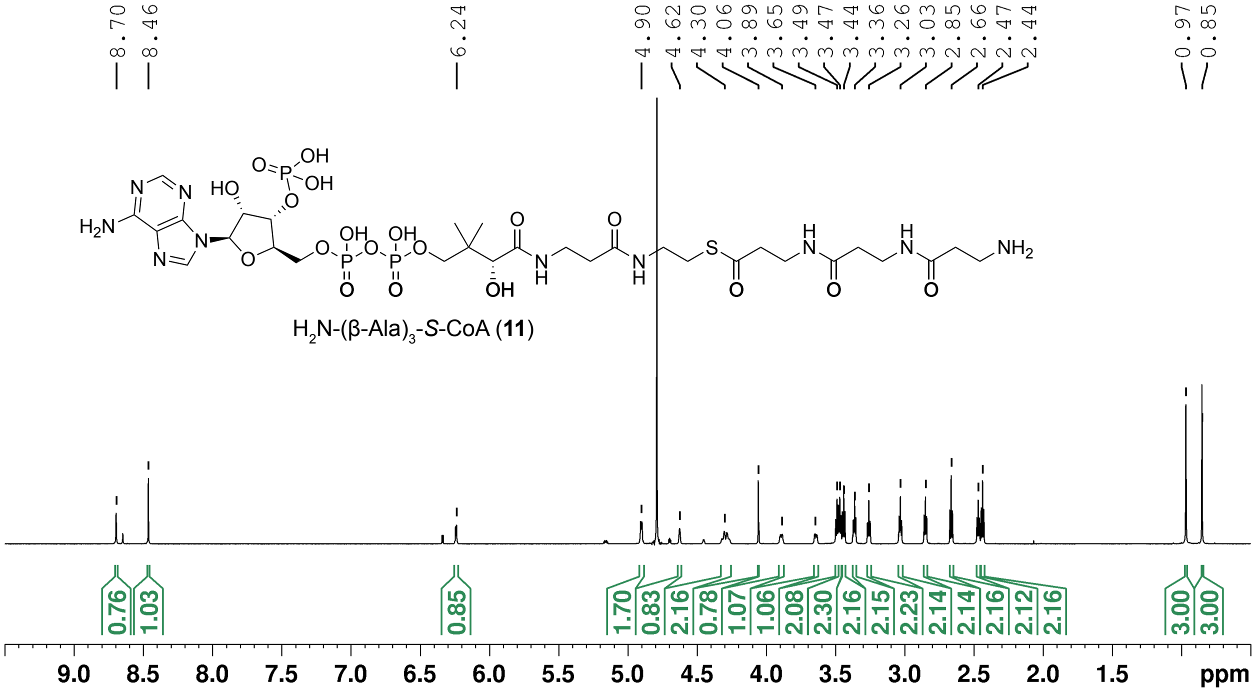

Supplementary Figure 87. ^{1}H-NMR spectrum of synthetic H_2N-(β-Ala)$_3$-*S*-CoA (**11**).

Molecular formula: $C_{30}H_{51}N_{10}O_{19}P_3S$

^{1}H-NMR (700 MHz, D_2O): δ = 8.70 (s, 1H), 8.46 (s, 1H), 6.24 (d, J_{HH} = 5.0 Hz, 1H) , 4.90 (ol, 2H), 4.62 (br, 1H), 4.30 (m, 2H), 4.06 (s, 1H), 3.89 (dd, J_{HH} = 9.7 Hz, J_{HH} = 4.0 Hz, 1H), 3.65 (dd, J_{HH} = 10.1 Hz, J_{HH} = 3.3 Hz, 1H), 3.49 (t, J_{HH} = 6.4 Hz, 2H), 3.47 (t, J_{HH} = 6.4 Hz, 2H), 3.44 (t, J_{HH} = 6.6 Hz, 2H), 3.36 (t, J_{HH} = 6.4 Hz, 2H), 3.26 (t, J_{HH} = 6.7 Hz, 2H), 3.03 (t, J_{HH} = 6.5 Hz, 2H), 2.85 (t, J_{HH} = 6.4 Hz, 2H), 2.66 (t, J_{HH} = 6.7 Hz, 2H), 2.47 (t, J_{HH} = 6.7 Hz, 2H), 2.44 (t, J_{HH} = 6.7 Hz, 2H), 0.97 (s, 3H), 0.85 (s, 3H).

HRMS (ESI, *m/z*): calculated $[M+H]^+$ 981.2338, observed 981.2194 (491.1184 for $[M+2H]^{2+}$).

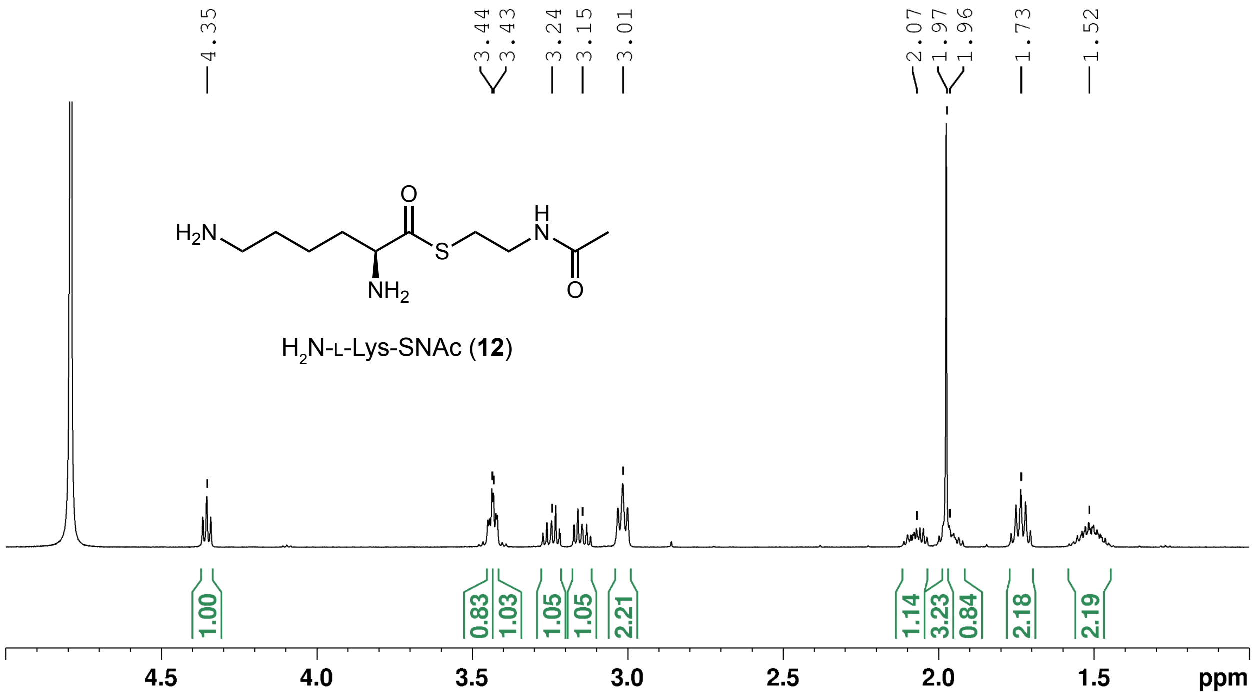

Supplementary Figure 88. ^{1}H-NMR spectrum of synthetic H_2N-L-Lys-SNAc (**12**).

Molecular formula: $C_{10}H_{21}N_3O_2S$

^{1}H-NMR (500 MHz, D_2O): δ = 4.35 (t, J_{HH} = 6.4 Hz, 1H), 3.44 (m, 1H), 3.43 (m, 1H), 3.24 (dt, J_{HH} = 14.1, 6.4 Hz, 1H), 3.15 (dt, J_{HH} = 14.1, 6.1 Hz, 1H), 3.01 (t, J_{HH} = 7.8 Hz, 2H), 2.07 (m, 1H), 1.97 (s, 3H), 1.96 (m, 1H), 1.73 (quintet, J_{HH} = 7.7 Hz, 2H), 1.52 ppm (m, 2H).

HRMS (ESI, *m/z*): calculated $[M+H]^+$ 248.1427, observed 248.1423.

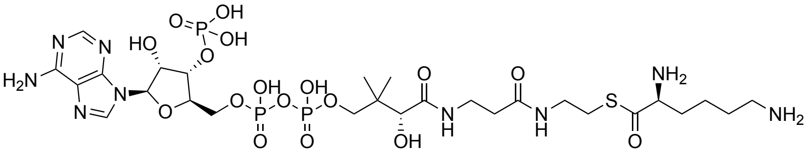

H_2N-L-Lys-*S*-CoA (**13**)

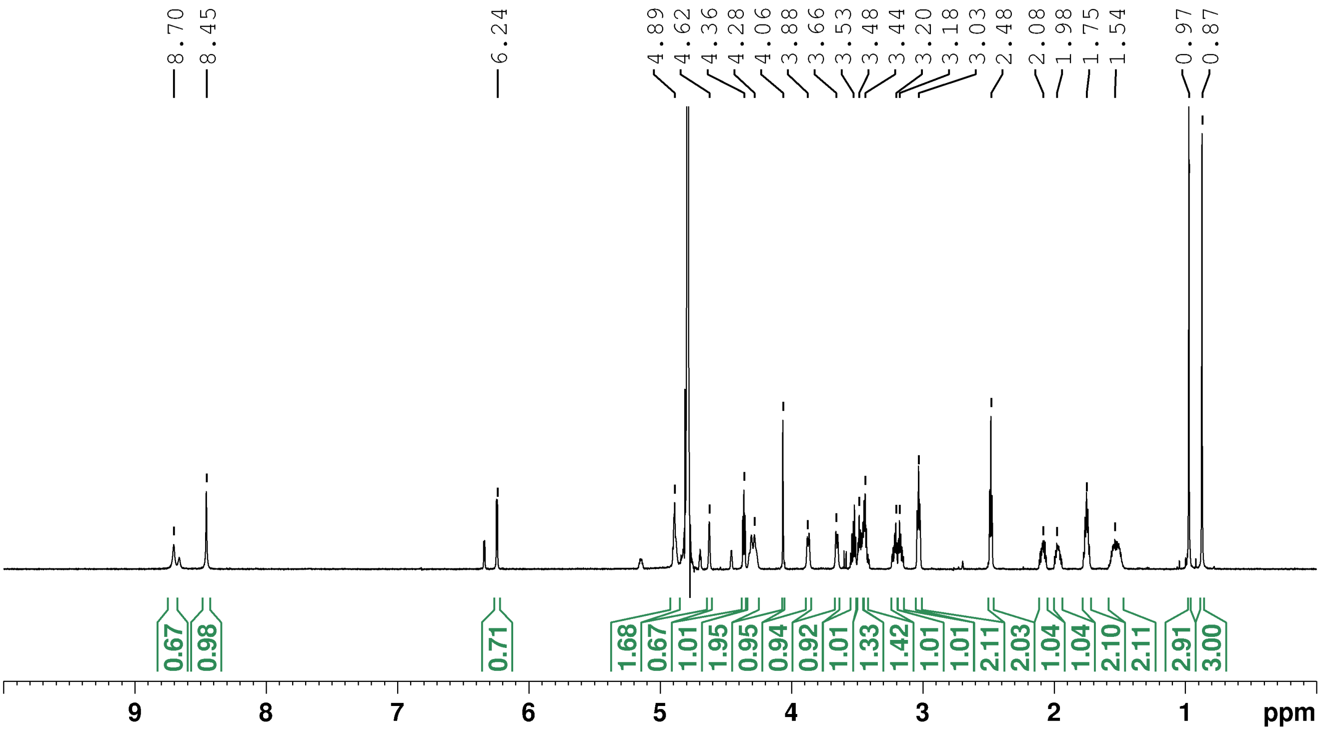

Supplementary Figure 89. ^{1}H-NMR spectrum of synthetic H_2N-L-Lys-*S*-CoA (**13**).

Molecular formula: $C_{27}H_{48}N_9O_{17}P_3S$

^{1}**H-NMR** (700 MHz, D_2O): δ = 8.70 (s, 1H), 8.45 (s, 1H), 6.24 (d, J_{HH} = 5.4 Hz, 1H), 4.89 (m, 2H), 4.62 (br, 1H), 4.36 (t, J_{HH} = 6.4 Hz, 1H), 4.28 (m, 2H), 4.06 (s, 1H), 3.88 (d, J_{HH} = 8.5 Hz, 1H), 3.66 (d, J_{HH} = 8.9 Hz, 1H), 3.53 (m, 1H), 3.48 (m, 1H), 3.44 (m, 2H), 3.20 (m, 1H), 3.18 (m, 1H), 3.03 (t, J_{HH} = 7.8 Hz, 2H), 2.48 (t, J_{HH} = 6.4 Hz, 2H), 2.08 (m, 1H), 1.98 (m, 1H), 1.75 (m, 2H), 1.54 (m, 2H), 0.97 (s, 3H), 0.87 (s, 3H).

HRMS (ESI, *m/z*): calculated $[M+H]^+$ 896.2174, observed 896.2176 (448.6115 for $[M+2H]^{2+}$).

References

(1) Wu, Y.; Liu, L.; Chen, H.-F.; Jiao, W.-H.; Sun, F.; Liu, L.-Y.; Zhu, H.-R.; Wang, S.-P.; Lin, H.-W. Fuscasins A–D, Cycloheptapeptides from the Marine Sponge Phakellia Fusca. *J. Nat. Prod.* **2019**, *82* (4), 970–979. https://doi.org/10.1021/acs.jnatprod.8b01033.

(2) Skinner, S. P.; Fogh, R. H.; Boucher, W.; Ragan, T. J.; Mureddu, L. G.; Vuister, G. W. CcpNmr AnalysisAssign: A Flexible Platform for Integrated NMR Analysis. *J. Biomol. NMR* **2016**, *66* (2), 111–124. https://doi.org/10.1007/s10858-016-0060-y.

(3) Arendrup, M. C.; Cuenca-Estrella, M.; Lass-Flörl, C.; Hope, W.; Eucast-Afst, T. EUCAST Technical Note on the EUCAST Definitive Document EDef 7.2: Method for the Determination of Broth Dilution Minimum Inhibitory Concentrations of Antifungal Agents for Yeasts EDef 7.2 (EUCAST-AFST). *Clin. Microbiol. Infect.* **2012**, *18* (7), 246–247. https://doi.org/10.1111/j.1469-0691.2012.03880.x.

(4) Tudela, J. L. R.; Donnelly, J. P.; Arendrup, M. C.; Arikan, S.; Barchiesi, F.; Bille, J.; Chryssanthou, E.; Cuenca-Estrella, M.; Dannaoui, E.; Denning, D.; Fegeler, W.; Gaustad, P.; Lass-Flörl, C.; Moore, C.; Richardson, M.; Schmalreck, A.; Velegraki, J. A.; Verweij, P. EUCAST Technical Note on the Method for the Determination of Broth Dilution Minimum Inhibitory Concentrations of Antifungal Agents for Conidia–Forming Moulds. *Clin. Microbiol. Infect.* **2008**, *14* (10), 982–984. https://doi.org/10.1111/j.1469-0691.2008.02086.x.

(5) Sullivan, M. J.; Petty, N. K.; Beatson, S. A. Easyfig: A Genome Comparison Visualizer. *Bioinformatics* **2011**, *27* (7), 1009–1010. https://doi.org/10.1093/bioinformatics/btr039.

(6) Saitou, N.; Nei, M. The Neighbor-Joining Method: A New Method for Reconstructing Phylogenetic Trees. *Molecular Biology and Evolution* **1987**, *4* (4), 406–425. https://doi.org/10.1093/oxfordjournals.molbev.a040454.

(7) Felsenstein, J. Confidence Limits on Phylogenies: An Approach Using the Bootstrap. *Evolution* **1985**, *39* (4), 783–791. https://doi.org/10.2307/2408678.

(8) Nei, M.; Kumar, S.; Nei, M.; Kumar, S. *Molecular Evolution and Phylogenetics*; Oxford University Press: Oxford, New York, 2000.

(9) Tamura, K.; Stecher, G.; Kumar, S. MEGA11: Molecular Evolutionary Genetics Analysis Version 11. *Molecular Biology and Evolution* **2021**, *38* (7), 3022–3027. https://doi.org/10.1093/molbev/msab120.

(10) Stecher, G.; Tamura, K.; Kumar, S. Molecular Evolutionary Genetics Analysis (MEGA) for macOS. *Molecular Biology and Evolution* **2020**, *37* (4), 1237–1239. https://doi.org/10.1093/molbev/msz312.

(11) Letunic, I.; Bork, P. Interactive Tree Of Life (iTOL) v5: An Online Tool for Phylogenetic Tree Display and Annotation. *Nucleic Acids Research* **2021**, *49* (W1), 293–296. https://doi.org/10.1093/nar/gkab301.

(12) Madeira, F.; Pearce, M.; Tivey, A. R. N.; Basutkar, P.; Lee, J.; Edbali, O.; Madhusoodanan, N.; Kolesnikov, A.; Lopez, R. Search and Sequence Analysis Tools Services from EMBL-EBI in 2022. *Nucleic Acids Research* **2022**, *50* (W1), 276–279. https://doi.org/10.1093/nar/gkac240.

(13) Waterhouse, A. M.; Procter, J. B.; Martin, D. M. A.; Clamp, M.; Barton, G. J. Jalview Version 2—a Multiple Sequence Alignment Editor and Analysis Workbench. *Bioinformatics* **2009**, *25* (9), 1189–1191. https://doi.org/10.1093/bioinformatics/btp033.

(14) Pei, J.; Kim, B.-H.; Grishin, N. V. PROMALS3D: A Tool for Multiple Protein Sequence and Structure Alignments. *Nucleic Acids Research* **2008**, *36* (7), 2295–2300. https://doi.org/10.1093/nar/gkn072.

(15) van Kempen, M.; Kim, S. S.; Tumescheit, C.; Mirdita, M.; Lee, J.; Gilchrist, C. L. M.; Söding, J.; Steinegger, M. Fast and Accurate Protein Structure Search with Foldseek. *Nat. Biotechnol.* **2024**, *42* (2), 243–246. https://doi.org/10.1038/s41587-023-01773-0.

(16) Jumper, J.; Evans, R.; Pritzel, A.; Green, T.; Figurnov, M.; Ronneberger, O.; Tunyasuvunakool, K.; Bates, R.; Žídek, A.; Potapenko, A.; Bridgland, A.; Meyer, C.; Kohl, S. A. A.; Ballard, A. J.; Cowie, A.; Romera-Paredes, B.; Nikolov, S.; Jain, R.; Adler, J.; Back, T.; Petersen, S.; Reiman, D.; Clancy, E.; Zielinski, M.; Steinegger, M.; Pacholska, M.; Berghammer, T.; Bodenstein, S.; Silver, D.; Vinyals, O.; Senior, A. W.; Kavukcuoglu, K.; Kohli, P.; Hassabis, D. Highly Accurate Protein Structure Prediction with AlphaFold. *Nature* **2021**, *596* (7873), 583–589. https://doi.org/10.1038/s41586-021-03819-2.

(17) Mirdita, M.; Schütze, K.; Moriwaki, Y.; Heo, L.; Ovchinnikov, S.; Steinegger, M. ColabFold: Making Protein Folding Accessible to All. *Nat. Methods.* **2022**, *19* (6), 679–682. https://doi.org/10.1038/s41592-022-01488-1.

(18) Mitchell, A. L.; Almeida, A.; Beracochea, M.; Boland, M.; Burgin, J.; Cochrane, G.; Crusoe, M. R.; Kale, V.; Potter, S. C.; Richardson, L. J.; Sakharova, E.; Scheremetjew, M.; Korobeynikov, A.; Shlemov, A.; Kunyavskaya, O.; Lapidus, A.; Finn, R. D. MGnify: The Microbiome Analysis Resource in 2020. *Nucleic Acids Research* **2019**, *48*, 570–578. https://doi.org/10.1093/nar/gkz1035.

(19) Mirdita, M.; Steinegger, M.; Söding, J. MMseqs2 Desktop and Local Web Server App for Fast, Interactive Sequence Searches. *Bioinformatics* **2019**, *35* (16), 2856–2858. https://doi.org/10.1093/bioinformatics/bty1057.

(20) Mirdita, M.; von den Driesch, L.; Galiez, C.; Martin, M. J.; Söding, J.; Steinegger, M. Uniclust Databases of Clustered and Deeply Annotated Protein Sequences and Alignments. *Nucleic Acids Research* **2017**, *45* (D1), 170–176. https://doi.org/10.1093/nar/gkw1081.

(21) Goddard, T. D.; Huang, C. C.; Meng, E. C.; Pettersen, E. F.; Couch, G. S.; Morris, J. H.; Ferrin, T. E. UCSF ChimeraX: Meeting Modern Challenges in Visualization and Analysis. *Protein Science* **2018**, *27* (1), 14–25. https://doi.org/10.1002/pro.3235.

(22) Pettersen, E. F.; Goddard, T. D.; Huang, C. C.; Meng, E. C.; Couch, G. S.; Croll, T. I.; Morris, J. H.; Ferrin, T. E. UCSF ChimeraX: Structure Visualization for Researchers, Educators, and Developers. *Protein Science* **2021**, *30* (1), 70–82. https://doi.org/10.1002/pro.3943.

(23) Sanner, M. F. Python: A Programming Language for Software Integration and Development.

(24) O'Boyle, N. M.; Banck, M.; James, C. A.; Morley, C.; Vandermeersch, T.; Hutchison, G. R. Open Babel: An Open Chemical Toolbox. *Journal of Cheminformatics* **2011**, *3* (1), 33–46. https://doi.org/10.1186/1758-2946-3-33.

(25) Eberhardt, J.; Santos-Martins, D.; Tillack, A. F.; Forli, S. AutoDock Vina 1.2.0: New Docking Methods, Expanded Force Field, and Python Bindings. *J. Chem. Inf. Model.* **2021**, *61* (8), 3891–3898. https://doi.org/10.1021/acs.jcim.1c00203.

(26) Gibson, D. G.; Young, L.; Chuang, R.-Y.; Venter, J. C.; Hutchison, C. A.; Smith, H. O. Enzymatic Assembly of DNA Molecules up to Several Hundred Kilobases. *Nat. Methods.* **2009**, *6* (5), 343–345. https://doi.org/10.1038/nmeth.1318.

(27) Gonsior, M.; Mühlenweg, A.; Tietzmann, M.; Rausch, S.; Poch, A.; Süssmuth, R. D. Biosynthesis of the Peptide Antibiotic Feglymycin by a Linear Nonribosomal Peptide Synthetase Mechanism. *ChemBioChem* **2015**, *16* (18), 2610–2614. https://doi.org/10.1002/cbic.201500432.

(28) Flannagan, R. S.; Linn, T.; Valvano, M. A. A System for the Construction of Targeted Unmarked Gene Deletions in the Genus Burkholderia. *Environmental Microbiology* **2008**, *10* (6), 1652–1660. https://doi.org/10.1111/j.1462-2920.2008.01576.x.

(29) Hogan, A. M.; Rahman, A. S. M. Z.; Lightly, T. J.; Cardona, S. T. A Broad-Host-Range CRISPRi Toolkit for Silencing Gene Expression in Burkholderia. *ACS Synth. Biol.* **2019**, *8* (10), 2372–2384. https://doi.org/10.1021/acssynbio.9b00232.

(30) Miller, J. H. *Experiments in Molecular Genetics*; Cold Spring Harbor Laboratory: Cold Spring Harbor, New York, 1972.

(31) Wilson, D. J.; Aldrich, C. C. A Continuous Kinetic Assay for Adenylation Enzyme Activity and Inhibition. *Analytical Biochemistry* **2010**, *404* (1), 56–63. https://doi.org/10.1016/j.ab.2010.04.033.

(32) Duckworth, B. P.; Wilson, D. J.; Aldrich, C. C. Measurement of Nonribosomal Peptide Synthetase Adenylation Domain Activity Using a Continuous Hydroxylamine Release Assay. In *Nonribosomal Peptide and Polyketide Biosynthesis*; Evans, B. S., Ed.; Methods in Molecular Biology; Springer New York: New York, NY, 2016; Vol. 1401, pp 53–61. https://doi.org/10.1007/978-1-4939-3375-4_3.

(33) Trottmann, F.; Fiedler, J.; Ishida, K.; Ishida-Ito, M.; Little, R. F.; Hertweck, C. Bacterial Pathogen Channels Medium-Sized Fatty Acids into Malleicyprol Biosynthesis. *ACS Chem. Biol.* **2023**, *18* (7), 1557–1563. https://doi.org/10.1021/acschembio.3c00188.

(34) Gaudelli, N. M.; Long, D. H.; Townsend, C. A. β-Lactam Formation by a Non-Ribosomal Peptide Synthetase during Antibiotic Biosynthesis. *Nature* **2015**, *520* (7547), 383–387. https://doi.org/10.1038/nature14100.

(35) Kaniusaite, M.; Tailhades, J.; A. Marschall, E.; A. Goode, R. J.; B. Schittenhelm, R.; J. Cryle, M. A Proof-Reading Mechanism for Non-Proteinogenic Amino Acid Incorporation into Glycopeptide Antibiotics. *Chemical Science* **2019**, *10* (41), 9466–9482. https://doi.org/10.1039/C9SC03678D.

(36) Prior, A. M.; Hori, T.; Fishman, A.; Sun, D. Recent Reports of Solid-Phase Cyclohexapeptide Synthesis and Applications. *Molecules* **2018**, *23* (6), 1475–1500. https://doi.org/10.3390/molecules23061475.

(37) *Fmoc Solid Phase Peptide Synthesis: A Practical Approach*; Chan, W., White, P., Eds.; Practical Approach Series; Oxford University Press: Oxford, New York, 1999.

(38) Jenner, M.; Frank, S.; Kampa, A.; Kohlhaas, C.; Pöplau, P.; Briggs, G. S.; Piel, J.; Oldham, N. J. Substrate Specificity in Ketosynthase Domains from Trans-AT Polyketide Synthases. *Angew. Chem. Int. Ed.* **2013**, *52* (4), 1143–1147. https://doi.org/10.1002/anie.201207690.

(39) Ehmann, D. E.; Trauger, J. W.; Stachelhaus, T.; Walsh, C. T. Aminoacyl-SNACs as Small-Molecule Substrates for the Condensation Domains of Nonribosomal Peptide Synthetases. *Chemistry & Biology* **2000**, *7* (10), 765–772. https://doi.org/10.1016/S1074-5521(00)00022-3.

(40) Li, B.; Berliner, M.; Buzon, R.; Chiu, C. K.-F.; Colgan, S. T.; Kaneko, T.; Keene, N.; Kissel, W.; Le, T.; Leeman, K. R.; Marquez, B.; Morris, R.; Newell, L.; Wunderwald, S.; Witt, M.; Weaver, J.; Zhang, Z.; Zhang, Z. Aqueous Phosphoric Acid as a Mild Reagent for Deprotection of Tert-Butyl Carbamates, Esters, and Ethers. *J. Org. Chem.* **2006**, *71* (24), 9045–9050. https://doi.org/10.1021/jo061377b.

(41) Dunbar, K. L.; Dell, M.; Gude, F.; Hertweck, C. Reconstitution of Polythioamide Antibiotic Backbone Formation Reveals Unusual Thiotemplated Assembly Strategy. *Proc. Natl. Acad. Sci.* **2020**, *117* (16), 8850–8858. https://doi.org/10.1073/pnas.1918759117.

(42) Dashti, Y.; Nakou, I. T.; Mullins, A. J.; Webster, G.; Jian, X.; Mahenthiralingam, E.; Challis, G. L. Discovery and Biosynthesis of Bolagladins: Unusual Lipodepsipeptides from Burkholderia Gladioli Clinical Isolates. *Angew. Chem. Int. Ed. Engl.* **2020**, *59* (48), 21553–21561. https://doi.org/10.1002/anie.202009110.

(43) Dose, B.; Ross, C.; Niehs, S. P.; Scherlach, K.; Bauer, J. P.; Hertweck, C. Food-Poisoning Bacteria Employ a Citrate Synthase and a Type II NRPS to Synthesize Bolaamphiphilic Lipopeptide Antibiotics. *Angew. Chem. Int. Ed. Engl.* **2020**, *59* (48), 21535–21540. https://doi.org/10.1002/anie.202009107.

(44) Ota, Y.; Tamegai, H.; Kudo, F.; Kuriki, H.; Koike-Takeshita, A.; Eouchi, T.; Kakinuma, K. Butirosin-Biosynthetic Gene Cluster from Bacillus Circulans. *J. Antibiot.* **2000**, *53* (10), 1158–1167. https://doi.org/10.7164/antibiotics.53.1158.

(45) Shinohara, Y.; Kudo, F.; Eguchi, T. A Natural Protecting Group Strategy to Carry an Amino Acid Starter Unit in the Biosynthesis of Macrolactam Polyketide Antibiotics. *J. Am. Chem. Soc.* **2011**, *133* (45), 18134–18137. https://doi.org/10.1021/ja208927r.

(46) Ogasawara, Y.; Katayama, K.; Minami, A.; Otsuka, M.; Eguchi, T.; Kakinuma, K. Cloning, Sequencing, and Functional Analysis of the Biosynthetic Gene Cluster of Macrolactam Antibiotic Vicenistatin in Streptomyces Halstedii. *Chemistry & Biology* **2004**, *11* (1), 79–86. https://doi.org/10.1016/j.chembiol.2003.12.010.

(47) Hwang, S.; Kim, E.; Lee, J.; Shin, J.; Yoon, Y. J.; Oh, D.-C. Structure Revision and the Biosynthetic Pathway of Tripartilactam. *J. Nat. Prod.* **2020**, *83* (3), 578–583. https://doi.org/10.1021/acs.jnatprod.9b00819.

(48) Low, Z. J.; Pang, L. M.; Ding, Y.; Cheang, Q. W.; Le Mai Hoang, K.; Thi Tran, H.; Li, J.; Liu, X.-W.; Kanagasundaram, Y.; Yang, L.; Liang, Z.-X. Identification of a Biosynthetic Gene Cluster for the Polyene Macrolactam Sceliphrolactam in a Streptomyces Strain Isolated from Mangrove Sediment. *Sci. Rep.* **2018**, *8* (1), 1594. https://doi.org/10.1038/s41598-018-20018-8.

(49) Shin, Y.-H.; Beom, J. Y.; Chung, B.; Shin, Y.; Byun, W. S.; Moon, K.; Bae, M.; Lee, S. K.; Oh, K.-B.; Shin, J.; Yoon, Y. J.; Oh, D.-C. Bombyxamycins A and B, Cytotoxic Macrocyclic Lactams from an Intestinal Bacterium of the Silkworm Bombyx Mori. *Org. Lett.* **2019**, *21* (6), 1804–1808. https://doi.org/10.1021/acs.orglett.9b00384.

(50) Beemelmanns, C.; Ramadhar, T. R.; Kim, K. H.; Klassen, J. L.; Cao, S.; Wyche, T. P.; Hou, Y.; Poulsen, M.; Bugni, T. S.; Currie, C. R.; Clardy, J. Macrotermycins A–D, Glycosylated Macrolactams from a Termite-Associated Amycolatopsis Sp. M39. *Org. Lett.* **2017**, *19* (5), 1000–1003. https://doi.org/10.1021/acs.orglett.6b03831.

(51) Derewacz, D. K.; Covington, B. C.; McLean, J. A.; Bachmann, B. O. Mapping Microbial Response Metabolomes for Induced Natural Product Discovery. *ACS Chem. Biol.* **2015**, *10* (9), 1998–2006. https://doi.org/10.1021/acschembio.5b00001.

(52) Barajas, J. F.; Zargar, A.; Pang, B.; Benites, V. T.; Gin, J.; Baidoo, E. E. K.; Petzold, C. J.; Hillson, N. J.; Keasling, J. D. Biochemical Characterization of β-Amino Acid Incorporation in Fluvirucin B2 Biosynthesis. *ChemBioChem* **2018**, *19* (13), 1391–1395. https://doi.org/10.1002/cbic.201800169.

(53) Miller, I. J.; Chevrette, M. G.; Kwan, J. C. Interpreting Microbial Biosynthesis in the Genomic Age: Biological and Practical Considerations. *Marine Drugs* **2017**, *15* (6), 165. https://doi.org/10.3390/md15060165.

(54) Skellam, E. J.; Stewart, A. K.; Strangman, W. K.; Wright, J. L. C. Identification of Micromonolactam, a New Polyene Macrocyclic Lactam from Two Marine Micromonospora Strains Using Chemical and Molecular Methods: Clarification of the Biosynthetic Pathway from a Glutamate Starter Unit. *J. Antibiot.* **2013**, *66* (7), 431–441. https://doi.org/10.1038/ja.2013.34.

(55) Schulze, C. J.; Donia, M. S.; Siqueira-Neto, J. L.; Ray, D.; Raskatov, J. A.; Green, R. E.; McKerrow, J. H.; Fischbach, M. A.; Linington, R. G. Genome-Directed Lead Discovery: Biosynthesis, Structure Elucidation, and Biological Evaluation of Two Families of Polyene Macrolactams against Trypanosoma Brucei. *ACS Chem. Biol.* **2015**, *10* (10), 2373–2381. https://doi.org/10.1021/acschembio.5b00308.

(56) Land, M.; Lapidus, A.; Mayilraj, S.; Chen, F.; Copeland, A.; Del Rio, T. G.; Nolan, M.; Lucas, S.; Tice, H.; Cheng, J.-F.; Chertkov, O.; Bruce, D.; Goodwin, L.; Pitluck, S.; Rohde, M.; Göker, M.; Pati, A.; Ivanova, N.; Mavromatis, K.; Chen, A.; Palaniappan, K.; Hauser, L.; Chang, Y.-J.; Jeffries, C. C.; Brettin, T.; Detter, J. C.; Han, C.; Chain, P.; Tindall, B. J.; Bristow, J.; Eisen, J. A.; Markowitz, V.; Hugenholtz, P.; Kyrpides, N. C.; Klenk, H.-P. Complete Genome Sequence of Actinosynnema Mirum Type Strain (101T). *Stand. Genomic Sci.* **2009**, *1* (1), 46–53. https://doi.org/10.4056/sigs.21137.

(57) Takaishi, M.; Kudo, F.; Eguchi, T. Identification of the Incednine Biosynthetic Gene Cluster: Characterization of Novel β-Glutamate-β-Decarboxylase IdnL3. *J. Antibiot.* **2013**, *66* (12), 691–699. https://doi.org/10.1038/ja.2013.76.

(58) Lim, Y. H.; Wong, F. T.; Yeo, W. L.; Ching, K. C.; Lim, Y. W.; Heng, E.; Chen, S.; Tsai, D.-J.; Lauderdale, T.-L.; Shia, K.-S.; Ho, Y. S.; Hoon, S.; Ang, E. L.; Zhang, M. M.; Zhao, H. Auroramycin: A Potent Antibiotic from Streptomyces Roseosporus by CRISPR-Cas9 Activation. *ChemBioChem* **2018**, *19* (16), 1716–1719. https://doi.org/10.1002/cbic.201800266.

(59) Conti, E.; Stachelhaus, T.; Marahiel, M. A.; Brick, P. Structural Basis for the Activation of Phenylalanine in the Non-Ribosomal Biosynthesis of Gramicidin S. *The EMBO Journal* **1997**, *16* (14), 4174–4183. https://doi.org/10.1093/emboj/16.14.4174.

(60) Stachelhaus, T.; Mootz, H. D.; Marahiel, M. A. The Specificity-Conferring Code of Adenylation Domains in Nonribosomal Peptide Synthetases. *Chemistry & Biology* **1999**, *6* (8), 493–505. https://doi.org/10.1016/S1074-5521(99)80082-9.

(61) Cieślak, J.; Miyanaga, A.; Takaku, R.; Takaishi, M.; Amagai, K.; Kudo, F.; Eguchi, T. Biochemical Characterization and Structural Insight into Aliphatic β-Amino Acid Adenylation Enzymes IdnL1 and CmiS6. *Proteins: Structure, Function, and Bioinformatics* **2017**, *85* (7), 1238–1247. https://doi.org/10.1002/prot.25284.

(62) Miyanaga, A.; Cieślak, J.; Shinohara, Y.; Kudo, F.; Eguchi, T. The Crystal Structure of the Adenylation Enzyme VinN Reveals a Unique β-Amino Acid Recognition Mechanism. *J. Biol. Chem.* **2014**, *289* (45), 31448–31457. https://doi.org/10.1074/jbc.M114.602326.

(63) Herbst, D. A.; Boll, B.; Zocher, G.; Stehle, T.; Heide, L. Structural Basis of the Interaction of MbtH-like Proteins, Putative Regulators of Nonribosomal Peptide Biosynthesis, with Adenylating Enzymes. *Journal of Biological Chemistry* **2013**, *288* (3), 1991–2003. https://doi.org/10.1074/jbc.M112.420182.

(64) Kudo, F.; Miyanaga, A.; Eguchi, T. Structural Basis of the Nonribosomal Codes for Nonproteinogenic Amino Acid Selective Adenylation Enzymes in the Biosynthesis of Natural Products. *JIMB* **2019**, *46* (3–4), 515–536. https://doi.org/10.1007/s10295-018-2084-7.

(65) Miyanaga, A.; Hayakawa, Y.; Numakura, M.; Hashimoto, J.; Teruya, K.; Hirano, T.; Shin-ya, K.; Kudo, F.; Eguchi, T. Identification of the Fluvirucin B2 (Sch 38518) Biosynthetic Gene Cluster from Actinomadura Fulva Subsp. Indica ATCC 53714: Substrate Specificity of the β-Amino Acid Selective Adenylating Enzyme FlvN. *Bioscience, Biotechnology, and Biochemistry* **2016**, *80* (5), 935–941. https://doi.org/10.1080/09168451.2015.1132155.

(66) Miao, V.; Coëffet-LeGal, M.-F.; Brian, P.; Brost, R.; Penn, J.; Whiting, A.; Martin, S.; Ford, R.; Parr, I.; Bouchard, M.; Silva, C. J.; Wrigley, S. K.; Baltz, R. H. Daptomycin Biosynthesis in Streptomyces Roseosporus: Cloning and Analysis of the Gene Cluster and Revision of Peptide Stereochemistry. *Microbiology* **2005**, *151* (5), 1507–1523. https://doi.org/10.1099/mic.0.27757-0.

(67) Weber, T.; Baumgartner, R.; Renner, C.; Marahiel, M. A.; Holak, T. A. Solution Structure of PCP, a Prototype for the Peptidyl Carrier Domains of Modular Peptide Synthetases. *Structure* **2000**, *8* (4). https://doi.org/10.1016/S0969-2126(00)00120-9.

(68) Malmierca, M. G.; Pérez-Victoria, I.; Martín, J.; Reyes, F.; Méndez, C.; Olano, C.; Salas, J. A. Cooperative Involvement of Glycosyltransferases in the Transfer of Amino Sugars during the Biosynthesis of the Macrolactam Sipanmycin by Streptomyces Sp. Strain CS149. *Applied and Environmental Microbiology* **2018**, *84* (18), e01462-18. https://doi.org/10.1128/AEM.01462-18.

(69) Gottardi, E. M.; Krawczyk, J. M.; von Suchodoletz, H.; Schadt, S.; Mühlenweg, A.; Uguru, G. C.; Pelzer, S.; Fiedler, H.-P.; Bibb, M. J.; Stach, J. E. M.; Süssmuth, R. D. Abyssomicin Biosynthesis: Formation of an Unusual Polyketide, Antibiotic-Feeding Studies and Genetic Analysis. *ChemBioChem* **2011**, *12* (9), 1401–1410. https://doi.org/10.1002/cbic.201100172.

(70) Dang, T.; Loll, B.; Müller, S.; Skobalj, R.; Ebeling, J.; Bulatov, T.; Gensel, S.; Göbel, J.; Wahl, M. C.; Genersch, E.; Mainz, A.; Süssmuth, R. D. Molecular Basis of Antibiotic Self-Resistance in a Bee Larvae Pathogen. *Nat Commun* **2022**, *13* (1), 2349. https://doi.org/10.1038/s41467-022-29829-w.

(71) Müller, S.; Garcia-Gonzalez, E.; Mainz, A.; Hertlein, G.; Heid, N. C.; Mösker, E.; Van Den Elst, H.; Overkleeft, H. S.; Genersch, E.; Süssmuth, R. D. Paenilamicin: Structure and Biosynthesis of a Hybrid Nonribosomal Peptide/Polyketide Antibiotic from the Bee Pathogen Paenibacillus Larvae. *Angew. Chem. Int. Ed. Engl.* **2014**, *53* (40), 10821–10825. https://doi.org/10.1002/anie.201404572.

(72) Cociancich, S.; Pesic, A.; Petras, D.; Uhlmann, S.; Kretz, J.; Schubert, V.; Vieweg, L.; Duplan, S.; Marguerettaz, M.; Noëll, J.; Pieretti, I.; Hügelland, M.; Kemper, S.; Mainz, A.; Rott, P.; Royer, M.; Süssmuth, R. D. The Gyrase Inhibitor Albicidin Consists of P-Aminobenzoic Acids and Cyanoalanine. *Nat Chem Biol* **2015**, *11* (3), 195–197. https://doi.org/10.1038/nchembio.1734.

(73) Mootz, H. D.; Marahiel, M. A. The Tyrocidine Biosynthesis Operon of Bacillus Brevis: Complete Nucleotide Sequence and Biochemical Characterization of Functional Internal Adenylation Domains. *Journal of Bacteriology* **1997**, *179* (21), 6843–6850. https://doi.org/10.1128/jb.179.21.6843-6850.1997.

(74) Koumoutsi, A.; Chen, X.-H.; Henne, A.; Liesegang, H.; Hitzeroth, G.; Franke, P.; Vater, J.; Borriss, R. Structural and Functional Characterization of Gene Clusters Directing Nonribosomal Synthesis of Bioactive Cyclic Lipopeptides in Bacillus Amyloliquefaciens Strain FZB42. *Journal of Bacteriology* **2004**, *186* (4), 1084–1096. https://doi.org/10.1128/jb.186.4.1084-1096.2004.

(75) E. Webb, M.; G. Smith, A.; Abell, C. Biosynthesis of Pantothenate. *Natural Product Reports* **2004**, *21* (6), 695–721. https://doi.org/10.1039/B316419P.

(76) Schmitzberger, F.; Kilkenny, M. L.; Lobley, C. M. C.; Webb, M. E.; Vinkovic, M.; Matak-Vinkovic, D.; Witty, M.; Chirgadze, D. Y.; Smith, A. G.; Abell, C.; Blundell, T. L. Structural Constraints on Protein Self-Processing in L-Aspartate-α-Decarboxylase. *The EMBO Journal* **2003**, *22* (23), 6193–6204. https://doi.org/10.1093/emboj/cdg575.

(77) Weber, T.; Laiple, K. J.; Pross, E. K.; Textor, A.; Grond, S.; Welzel, K.; Pelzer, S.; Vente, A.; Wohlleben, W. Molecular Analysis of the Kirromycin Biosynthetic Gene Cluster Revealed β-Alanine as Precursor of the Pyridone Moiety. *Chemistry & Biology* **2008**, *15* (2), 175–188. https://doi.org/10.1016/j.chembiol.2007.12.009.

(78) Wang, B.; Kang, Q.; Lu, Y.; Bai, L.; Wang, C. Unveiling the Biosynthetic Puzzle of Destruxins in Metarhizium Species. *Proceedings of the National Academy of Sciences* **2012**, *109* (4), 1287–1292. https://doi.org/10.1073/pnas.1115983109.

(79) Rivas Arenas, L. A.; de Paiva, F. C. R.; de O. Rossini, N.; Li, Y.; Spencer, J.; Leadlay, P.; Dias, M. V. B. Crystal Structure of BtrK, a Decarboxylase Involved in the (S)-4-Amino-2-Hydroxybutyrate (AHBA) Formation during Butirosin Biosynthesis. *J. Mol. Struct.* **2022**, *1267*, 133576. https://doi.org/10.1016/j.molstruc.2022.133576.

(80) Zhang, Z.; Zhou, R.; Sauder, J. M.; Tonge, P. J.; Burley, S. K.; Subramanyam Swaminathan. Structural and Functional Studies of Fatty Acyl Adenylate Ligases from E. Coli and L. Pneumophila. *J. Mol. Biol.* **2011**, *406* (2), 313–324. https://doi.org/10.1016/j.jmb.2010.12.011.

(81) Yan, M.; Ma, M.; Chen, R.; Cao, Y.; Zhang, W.; Liu, X. Structural Basis for the Development of Potential Inhibitors Targeting FadD23 from Mycobacterium Tuberculosis. *Acta Cryst F* **2023**, *79* (8), 208–216. https://doi.org/10.1107/S2053230X23005836.

(82) Siméone, R.; Léger, M.; Constant, P.; Malaga, W.; Marrakchi, H.; Daffé, M.; Guilhot, C.; Chalut, C. Delineation of the Roles of FadD22, FadD26 and FadD29 in the Biosynthesis of Phthiocerol Dimycocerosates and Related Compounds in Mycobacterium Tuberculosis. *The FEBS Journal* **2010**, *277* (12), 2715–2725. https://doi.org/10.1111/j.1742-4658.2010.07688.x.

(83) Nakamura, H.; Hamer, H. A.; Sirasani, G.; Balskus, E. P. Cylindrocyclophane Biosynthesis Involves Functionalization of an Unactivated Carbon Center. *J. Am. Chem. Soc.* **2012**, *134* (45), 18518–18521. https://doi.org/10.1021/ja308318p.

(84) Hayashi, T.; Kitamura, Y.; Funa, N.; Ohnishi, Y.; Horinouchi, S. Fatty Acyl-AMP Ligase Involvement in the Production of Alkylresorcylic Acid by a Myxococcus Xanthus Type III Polyketide Synthase. *ChemBioChem* **2011**, *12* (14), 2166–2176. https://doi.org/10.1002/cbic.201100344.

(85) Hansen, D. B.; Bumpus, S. B.; Aron, Z. D.; Kelleher, N. L.; Walsh, C. T. The Loading Module of Mycosubtilin: An Adenylation Domain with Fatty Acid Selectivity. *J. Am. Chem. Soc.* **2007**, *129* (20), 6366–6367. https://doi.org/10.1021/ja070890j.

(86) Mareš, J.; Hájek, J.; Urajová, P.; Kopecký, J.; Hrouzek, P. A Hybrid Non-Ribosomal Peptide/Polyketide Synthetase Containing Fatty-Acyl Ligase (FAAL) Synthesizes the β-Amino Fatty Acid Lipopeptides Puwainaphycins in the Cyanobacterium Cylindrospermum Alatosporum. *PLoS ONE* **2014**, *9* (11), e111904. https://doi.org/10.1371/journal.pone.0111904.

(87) Gulick, A. M.; Lu, X.; Dunaway-Mariano, D. Crystal Structure of 4-Chlorobenzoate:CoA Ligase/Synthetase in the Unliganded and Aryl Substrate-Bound States. *Biochemistry* **2004**, *43* (27), 8670–8679. https://doi.org/10.1021/bi049384m.

(88) Reger, A. S.; Wu, R.; Dunaway-Mariano, D.; Gulick, A. M. Structural Characterization of a 140° Domain Movement in the Two-Step Reaction Catalyzed by 4-Chlorobenzoate:CoA Ligase. *Biochemistry* **2008**, *47* (31), 8016–8025. https://doi.org/10.1021/bi800696y.

(89) Olsen, J. G.; Kadziola, A.; von Wettstein-Knowles, P.; Siggaard-Andersen, M.; Lindquist, Y.; Larsen, S. The X-Ray Crystal Structure of β-Ketoacyl Acyl Carrier Protein Synthase I. *FEBS Letters* **1999**, *460* (1), 46–52. https://doi.org/10.1016/S0014-5793(99)01303-4.

(90) Wang, J.; Soisson, S. M.; Young, K.; Shoop, W.; Kodali, S.; Galgoci, A.; Painter, R.; Parthasarathy, G.; Tang, Y. S.; Cummings, R.; Ha, S.; Dorso, K.; Motyl, M.; Jayasuriya, H.; Ondeyka, J.; Herath, K.; Zhang, C.; Hernandez, L.; Allocco, J.; Basilio, Á.; Tormo, J. R.; Genilloud, O.; Vicente, F.; Pelaez, F.; Colwell, L.; Lee, S. H.; Michael, B.; Felcetto, T.; Gill, C.; Silver, L. L.; Hermes, J. D.; Bartizal, K.; Barrett, J.; Schmatz, D.; Becker, J. W.; Cully, D.; Singh, S. B. Platensimycin Is a Selective FabF Inhibitor with Potent Antibiotic Properties. *Nature* **2006**, *441* (7091), 358–361. https://doi.org/10.1038/nature04784.

(91) Baum, B.; Lecker, L. S. M.; Zoltner, M.; Jaenicke, E.; Schnell, R.; Hunter, W. N.; Brenk, R. Structures of Pseudomonas Aeruginosa β-Ketoacyl-(Acyl-Carrier-Protein) Synthase II (FabF) and a C164Q Mutant Provide Templates for Antibacterial Drug Discovery and Identify a Buried Potassium Ion and a Ligand-

Binding Site That Is an Artefact of the Crystal Form. *Acta Crystallogr F Struct Biol Commun* **2015**, *71* (8), 1020–1026. https://doi.org/10.1107/S2053230X15010614.
(92) Musayev, F.; Sachdeva, S.; Neel Scarsdale, J.; Reynolds, K. A.; Wright, H. T. Crystal Structure of a Substrate Complex of Mycobacterium Tuberculosis β-Ketoacyl-Acyl Carrier Protein Synthase III (FabH) with Lauroyl-Coenzyme A. *Journal of Molecular Biology* **2005**, *346* (5), 1313–1321. https://doi.org/10.1016/j.jmb.2004.12.044.
(93) Pan, H.; Tsai, S.; Meadows, E. S.; Miercke, L. J. W.; Keatinge-Clay, A. T.; O'Connell, J.; Khosla, C.; Stroud, R. M. Crystal Structure of the Priming β-Ketosynthase from the R1128 Polyketide Biosynthetic Pathway.
(94) Ellis, B. D.; Milligan, J. C.; White, A. R.; Duong, V.; Altman, P. X.; Mohammed, L. Y.; Crump, M. P.; Crosby, J.; Luo, R.; Vanderwal, C. D.; Tsai, S.-C. An Oxetane-Based Polyketide Surrogate to Probe Substrate Binding in a Polyketide Synthase. *J. Am. Chem. Soc.* **2018**, *140* (15), 4961–4964. https://doi.org/10.1021/jacs.7b11793.
(95) Drake, E. J.; Miller, B. R.; Shi, C.; Tarrasch, J. T.; Sundlov, J. A.; Leigh Allen, C.; Skiniotis, G.; Aldrich, C. C.; Gulick, A. M. Structures of Two Distinct Conformations of Holo-Non-Ribosomal Peptide Synthetases. *Nature* **2016**, *529* (7585), 235–238. https://doi.org/10.1038/nature16163.
(96) Fortin, P. D.; Walsh, C. T.; Magarvey, N. A. A Transglutaminase Homologue as a Condensation Catalyst in Antibiotic Assembly Lines. *Nature* **2007**, *448* (7155), 824–827. https://doi.org/10.1038/nature06068.
(97) Llewellyn, N. M.; Li, Y.; Spencer, J. B. Biosynthesis of Butirosin: Transfer and Deprotection of the Unique Amino Acid Side Chain. *Chemistry & Biology* **2007**, *14* (4), 379–386. https://doi.org/10.1016/j.chembiol.2007.02.005.
(98) Cui, J. J.; Zhang, Y.; Ju, K.-S. Phosphonoalamides Reveal the Biosynthetic Origin of Phosphonoalanine Natural Products and a Convergent Pathway for Their Diversification. *Angew. Chem. Int. Ed. Engl.* **2024**, *63* (32), e202405052. https://doi.org/10.1002/anie.202405052.
(99) Zhang, H.; Zhang, C.; Li, Q.; Ma, J.; Ju, J. Metabolic Blockade-Based Genome Mining Reveals Lipochain-Linked Dihydro-β-Alanine Synthetases Involved in Autucedine Biosynthesis. *Org. Lett.* **2022**, *24* (30), 5535–5540. https://doi.org/10.1021/acs.orglett.2c01957.
(100) Saeed, A. U.; Rahman, M. U.; Chen, H.-F.; Zheng, J. Structural Insight of KSIII (β-Ketoacyl-ACP Synthase)-like Acyltransferase ChlB3 in the Biosynthesis of Chlorothricin. *Molecules* **2022**, *27* (19), 6405. https://doi.org/10.3390/molecules27196405.
(101) He, Q.-L.; Jia, X.-Y.; Tang, M.-C.; Tian, Z.-H.; Tang, G.-L.; Liu, W. Dissection of Two Acyl-Transfer Reactions Centered on Acyl-S-Carrier Protein Intermediates for Incorporating 5-Chloro-6-Methyl-O-Methylsalicyclic Acid into Chlorothricin. *ChemBioChem* **2009**, *10* (5), 813–819. https://doi.org/10.1002/cbic.200800714.
(102) Freitag, A.; Wemakor, E.; Li, S.-M.; Heide, L. Acyl Transfer in Clorobiocin Biosynthesis: Involvement of Several Proteins in the Transfer of the Pyrrole-2-Carboxyl Moiety to the Deoxysugar. *ChemBioChem* **2005**, *6* (12), 2316–2325. https://doi.org/10.1002/cbic.200500252.
(103) Strieter, E. R.; Vaillancourt, F. H.; Walsh, C. T. CmaE: A Transferase Shuttling Aminoacyl Groups between Carrier Protein Domains in the Coronamic Acid Biosynthetic Pathway. *Biochemistry* **2007**, *46* (25), 7549–7557. https://doi.org/10.1021/bi700243h.
(104) Tang, Z.; Pang, B.; Liu, C.; Guo, S.; Qu, X.; Liu, W. Formation and Loading of a (2S)-2-Ethylmalonamyl Starter Unit in the Assembly Line of Polyketide-Nonribosomal Peptide Hybrid Sanglifehrin A. *Angew. Chem. Int. Ed. Engl.* **2023**, *135* (23), e202217090. https://doi.org/10.1002/ange.202217090.
(105) Bretschneider, T.; Zocher, G.; Unger, M.; Scherlach, K.; Stehle, T.; Hertweck, C. A Ketosynthase Homolog Uses Malonyl Units to Form Esters in Cervimycin Biosynthesis. *Nat. Chem. Biol.* **2012**, *8* (2), 154–161. https://doi.org/10.1038/nchembio.746.
(106) Guo, X.; Zhang, J.; Han, L.; Lee, J.; Williams, S. C.; Forsberg, A.; Xu, Y.; Austin, R. N.; Feng, L. Structure and Mechanism of the Alkane-Oxidizing Enzyme AlkB. *Nat. Commun.* **2023**, *14* (1), 2180. https://doi.org/10.1038/s41467-023-37869-z.
(107) Chai, J.; Guo, G.; McSweeney, S. M.; Shanklin, J.; Liu, Q. Structural Basis for Enzymatic Terminal C–H Bond Functionalization of Alkanes. *Nat. Struct. Mol. Biol.* **2023**, *30* (4), 521–526. https://doi.org/10.1038/s41594-023-00958-0.
(108) Hallgren, J.; Tsirigos, K. D.; Pedersen, M. D.; Almagro Armenteros, J. J.; Marcatili, P.; Nielsen, H.; Krogh, A.; Winther, O. DeepTMHMM Predicts Alpha and Beta Transmembrane Proteins Using Deep Neural Networks. 2022. https://doi.org/10.1101/2022.04.08.487609.
(109) Russell, R. J.; Gerike, U.; Danson, M. J.; Hough, D. W.; Taylor, G. L. Structural Adaptations of the Cold-Active Citrate Synthase from an Antarctic Bacterium. *Structure* **1998**, *6* (3), 351–361. https://doi.org/10.1016/S0969-2126(98)00037-9.
(110) Kanamori, E.; Kawaguchi, S.; Kuramitsu, S.; Kouyama, T.; Murakami, M. Structural Comparison between the Open and Closed Forms of Citrate Synthase from *Thermus Thermophilus* HB8. *Biophysics and Physicobiology* **2015**, *12* (0), 47–56. https://doi.org/10.2142/biophysico.12.0_47.
(111) Bell, G. S.; Russell, R. J. M.; Connaris, H.; Hough, D. W.; Danson, M. J.; Taylor, G. L. Stepwise Adaptations of Citrate Synthase to Survival at Life's Extremes. *European Journal of Biochemistry* **2002**, *269* (24), 6250–6260. https://doi.org/10.1046/j.1432-1033.2002.03344.x.
(112) Francois, J. A.; Starks, C. M.; Sivanuntakorn, S.; Jiang, H.; Ransome, A. E.; Nam, J.-W.; Constantine, C. Z.; Kappock, T. J. Structure of a NADH-Insensitive Hexameric Citrate Synthase That Resists Acid Inactivation. *Biochemistry* **2006**, *45* (45), 13487–13499. https://doi.org/10.1021/bi061083k.
(113) Boutz, D. R.; Cascio, D.; Whitelegge, J.; Perry, L. J.; Yeates, T. O. Discovery of a Thermophilic Protein Complex Stabilized by Topologically Interlinked Chains. *Journal of Molecular Biology* **2007**, *368* (5), 1332–1344. https://doi.org/10.1016/j.jmb.2007.02.078.
(114) Ferraris, D. M.; Spallek, R.; Oehlmann, W.; Singh, M.; Rizzi, M. Structures of Citrate Synthase and Malate Dehydrogenase of Ycobacterium Tuberculosis. *Proteins: Structure, Function, and Bioinformatics* **2015**, *83* (2), 389–394. https://doi.org/10.1002/prot.24743.
(115) Schlachter, C. R.; Klapper, V.; Radford, T.; Chruszcz, M. Comparative Studies of Aspergillus Fumigatus 2-Methylcitrate Synthase and Human Citrate Synthase. *Biological Chemistry* **2019**, *400* (12), 1567–1581. https://doi.org/10.1515/hsz-2019-0106.
(116) Lee, S. H.; Kim, K.-J. Crystal Structure and Biochemical Properties of Msed_0281, the Citrate Synthase from Metallosphaera Sedula. *Biochemical and Biophysical Research Communications* **2019**, *509* (3), 722–727. https://doi.org/10.1016/j.bbrc.2018.12.172.
(117) Lee, S. H.; Son, H. F.; Kim, K.-J. Structural Insights into the Inhibition Properties of Archaeon Citrate Synthase from Metallosphaera Sedula. *PLoS ONE* **2019**, *14* (2), e0212807. https://doi.org/10.1371/journal.pone.0212807.
(118) Pathirage, R.; Favrot, L.; Petit, C.; Yamsek, M.; Singh, S.; Mallareddy, J. R.; Rana, S.; Natarajan, A.; Ronning, D. R. *Mycobacterium Tuberculosis* CitA Activity Is Modulated by Cysteine Oxidation and Pyruvate Binding. *RSC Med. Chem.* **2023**, *14* (5), 921–933. https://doi.org/10.1039/D3MD00058C.
(119) Sadler, J. C.; Chung, C. H.; Mosley, J. E.; Burley, G. A.; Humphreys, L. D. Structural and Functional Basis of C-Methylation of Coumarin Scaffolds by NovO. *ACS Chem. Biol.* **2017**, *12* (2), 374–379. https://doi.org/10.1021/acschembio.6b01053.
(120) Chai, A.-F.; Bulloch, E. M. M.; Evans, G. L.; Lott, J. S.; Baker, E. N.; Johnston, J. M. A Covalent Adduct of MbtN, an Acyl-ACP Dehydrogenase from Mycobacterium Tuberculosis, Reveals an Unusual Acyl-Binding Pocket. *Acta Crystallographica Section D: Biological Crystallography* **2015**, *71* (4), 862–872. https://doi.org/10.1107/S1399004715001650.
(121) Krithika, R.; Marathe, U.; Saxena, P.; Ansari, Mohd. Z.; Mohanty, D.; Gokhale, R. S. A Genetic Locus Required for Iron Acquisition in Mycobacterium Tuberculosis. *Proc. Natl. Acad. Sci.* **2006**, *103* (7), 2069–2074. https://doi.org/10.1073/pnas.0507924103.
(122) Watanabe, K.; Khosla, C.; Stroud, R. M.; Tsai, S.-C. Crystal Structure of an Acyl-ACP Dehydrogenase from the FK520 Polyketide Biosynthetic Pathway: Insights into Extender Unit Biosynthesis. *Journal of Molecular Biology* **2003**, *334* (3), 435–444. https://doi.org/10.1016/j.jmb.2003.10.021.
(123) Blake-Hedges, J. M.; Pereira, J. H.; Cruz-Morales, P.; Thompson, M. G.; Barajas, J. F.; Chen, J.; Krishna, R. N.; Chan, L. J. G.; Nimlos, D.; Alonso-Martinez, C.; Baidoo, E. E. K.; Chen, Y.; Gin, J. W.; Katz, L.; Petzold, C. J.; Adams, P. D.; Keasling, J. D. Structural Mechanism of Regioselectivity in an Unusual Bacterial Acyl-CoA Dehydrogenase. *J. Am. Chem. Soc.* **2020**, *142* (2), 835–846. https://doi.org/10.1021/jacs.9b09187.

(124) Battaile, K. P.; Molin-Case, J.; Paschke, R.; Wang, M.; Bennett, D.; Vockley, J.; Kim, J.-J. P. Crystal Structure of Rat Short Chain Acyl-CoA Dehydrogenase Complexed with Acetoacetyl-CoA. *Journal of Biological Chemistry* **2002**, *277* (14), 12200–12207. https://doi.org/10.1074/jbc.M111296200.

(125) Yang, M.; Lu, R.; Guja, K. E.; Wipperman, M. F.; St. Clair, J. R.; Bonds, A. C.; Garcia-Diaz, M.; Sampson, N. S. Unraveling Cholesterol Catabolism in Mycobacterium Tuberculosis: ChsE4-ChsE5 A2β2 Acyl-CoA Dehydrogenase Initiates β-Oxidation of 3-Oxo-Cholest-4-En-26-Oyl CoA. *ACS Infect. Dis.* **2015**, *1* (2), 110–125. https://doi.org/10.1021/id500033m.

(126) Kim, J. J.; Wang, M.; Paschke, R. Crystal Structures of Medium-Chain Acyl-CoA Dehydrogenase from Pig Liver Mitochondria with and without Substrate. *Proc. Natl. Acad. Sci.* **1993**, *90* (16), 7523–7527. https://doi.org/10.1073/pnas.90.16.7523.

(127) Satoh, A. Structure of the Transition State Analog of Medium-Chain Acyl-CoA Dehydrogenase. Crystallographic and Molecular Orbital Studies on the Charge-Transfer Complex of Medium-Chain Acyl-CoA Dehydrogenase with 3-Thiaoctanoyl-CoA. *J. Biochem.* **2003**, *134* (2), 297–304. https://doi.org/10.1093/jb/mvg143.

(128) McAndrew, R. P.; Wang, Y.; Mohsen, A.-W.; He, M.; Vockley, J.; Kim, J.-J. P. Structural Basis for Substrate Fatty Acyl Chain Specificity. *Journal of Biological Chemistry* **2008**, *283* (14), 9435–9443. https://doi.org/10.1074/jbc.M709135200.

(129) Chen, X.; Chen, J.; Yan, B.; Zhang, W.; Guddat, L. W.; Liu, X.; Rao, Z. Structural Basis for the Broad Substrate Specificity of Two Acyl-CoA Dehydrogenases FadE5 from Mycobacteria. *Proc. Natl. Acad. Sci.* **2020**, *117* (28), 16324–16332. https://doi.org/10.1073/pnas.2002835117.

(130) Battaile, K. P.; Nguyen, T. V.; Vockley, J.; Kim, J.-J. P. Structures of Isobutyryl-CoA Dehydrogenase and Enzyme-Product Complex. *Journal of Biological Chemistry* **2004**, *279* (16), 16526–16534. https://doi.org/10.1074/jbc.M400034200.

(131) Tiffany, K. A.; Roberts, D. L.; Wang, M.; Paschke, R.; Mohsen, A.-W. A.; Vockley, J.; Kim, J.-J. P. Structure of Human Isovaleryl-CoA Dehydrogenase at 2.6 Å Resolution: Structural Basis for Substrate Specificity. *Biochemistry* **1997**, *36* (28), 8455–8464. https://doi.org/10.1021/bi970422u.

(132) Berman, H.; Henrick, K.; Nakamura, H. Structure of Human Short-Branched Chain Acyl-CoA Dehydrogenase (ACADSB). *Nat. Struct. Mol. Biol.* **2003**, *10* (12), 980–980. https://doi.org/10.1038/nsb1203-980.

(133) Fu, Z.; Wang, M.; Paschke, R.; Rao, K. S.; Frerman, F. E.; Kim, J.-J. P. Crystal Structures of Human Glutaryl-CoA Dehydrogenase with and without an Alternate Substrate: Structural Bases of Dehydrogenation and Decarboxylation Reactions. *Biochemistry* **2004**, *43* (30), 9674–9684. https://doi.org/10.1021/bi049290c.

(134) Wischgoll, S.; Demmer, U.; Warkentin, E.; Günther, R.; Boll, M.; Ermler, U. Structural Basis for Promoting and Preventing Decarboxylation in Glutaryl-Coenzyme A Dehydrogenases. *Biochemistry* **2010**, *49* (25), 5350–5357. https://doi.org/10.1021/bi100317m.

(135) Carey, A. R. E.; Fukata, G.; O'Ferrall, R. A. M.; Murphy, M. G. The Mechanism of Irnine-Enamine Tautomerism of 2- and 4-Phenacylquinolines. *J. Chem. Soc., Perkin Trans. 2* **1985**, 1711–1722. https://doi.org/10.1039/P29850001711.

(136) Durand, N.; Bertrand, B.; Guyot, B.; Guiraud, J. P.; Tachon, A. F. Study on the Coffea Arabica/Colletotrichum Kahawae Pathosystem: Impact of a Biological Plant Protection Product. *J. Plant. Dis. Prot.* **2009**, *116* (2), 78–85. https://doi.org/10.1007/BF03356290.

(137) Musoli, C. P. Recherche de Sources de Résistance à La Trachéomycose Du Caféier Coffea Canephora Pierre, Due à Fusarium Xylarioides Steyaert En Ouganda. Thèse de doctorat, Ecole Nationale supérieure agronomique de Montpellier, 2007. https://agritrop.cirad.fr/538793/ (accessed 2024-08-20).

(138) Leung, H.; Borromeo, E. S.; Bernardo, M. A.; Nottéghem, J. L. Genetic Analysis of Virulence in the Rice Blast Fungus Magnaporthe Grisea. *Phytopathology* **1988**, *78* (9), 1227–1233.

(139) Kabore, K. H. Helminthosporiose du riz en Afrique de l'Ouest : identification des espèces responsables et diversité génétique de Bipolaris oryzae et Exserohilum rostratum. Thèse de doctorat, Université Paris-Saclay, 2022. https://theses.hal.science/tel-03722955 (accessed 2024-08-20).
